# Regiodivergent Ring-Expansion of Oxindoles to Quinolinones

**DOI:** 10.1021/jacs.3c12119

**Published:** 2024-02-09

**Authors:** Hendrik L. Schmitt, Den Martymianov, Ori Green, Tristan Delcaillau, Young Seo Park Kim, Bill Morandi

**Affiliations:** † Laboratorium für Organische Chemie, ETH Zürich, Vladimir-Prelog-Weg 3, HCI, 8093 Zürich, Switzerland

## Abstract

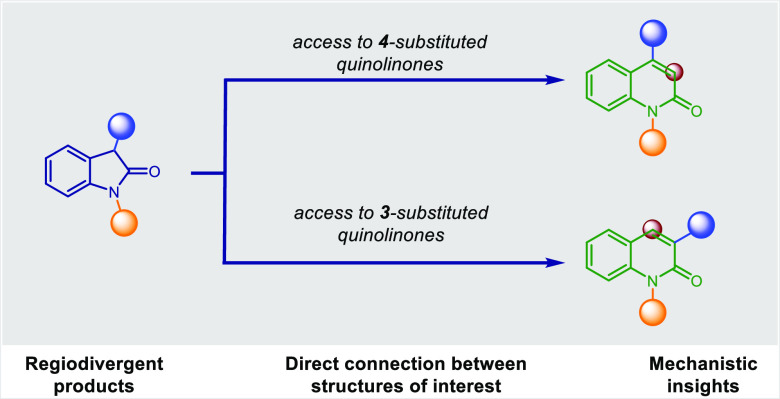

The development of divergent methods to expedite structure–activity relationship studies is crucial to streamline discovery processes. We developed a rare example of regiodivergent ring expansion to access two regioisomers from a common starting material. To enable this regiodivergence, we identified two distinct reaction conditions for transforming oxindoles into quinolinone isomers. The presented methods proved to be compatible with a variety of functional groups, which enabled the late-stage diversification of bioactive oxindoles as well as facilitated the synthesis of quinolinone drugs and their derivatives.

The diversification of molecular structures is key to the discovery of novel pharmaceuticals and other purpose-driven compounds.^1−4^ To achieve variations of the underlying core structures of lead compounds, de novo multistep syntheses are usually required, obstructing access to otherwise desirable analogues. Recently, new methods for the direct editing of core scaffolds, such as cut-and-sew, and skeletal editing strategies, have emerged,^1−11^ complementing established transformations such as e.g., the Beckmann rearrangement.^12−14^ These methods streamline access to new entities and potential drug candidates. However, numerous bioactive scaffolds are currently not amenable to such skeletal modifications, creating a strong demand for the development of new methodologies. Skeletal modifications that allow for the synthesis of multiple regioisomers are particularly rare, despite their potential to provide access to structural analogues for structure–activity relationship studies. The challenges of developing such reactions, namely, the need to activate strong bonds and identify complementary reaction conditions for regiodivergent conversion, have greatly limited progress in this field. Thus, besides classical nitrogen insertions,^15−18^ to the best of our knowledge, only few methods to selectively access skeletal regioisomers exist.^19−21^

**Figure 1:**
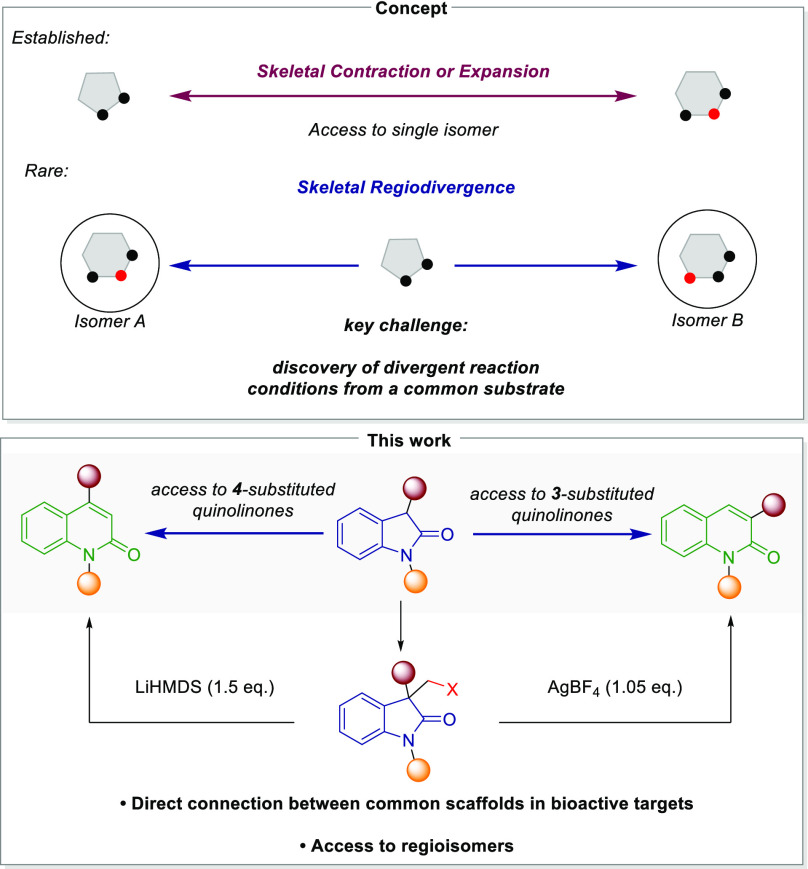
Context and importance of this work.

Two privileged pharmacologically relevant motifs are the oxindole and the quinolinone structure, for both of which a plethora of derivatives can be found in various therapeutical uses, ranging from treatment of bacterial infections to antiviral applications and cancer therapy.^22−29^ Consequently, establishing a straightforward synthetic pathway to convert oxindoles into quinolinones holds the potential for the identification of novel bioactive compounds. Such molecular diversification strategies would be particularly attractive if regioisomers of quinolinones could be accessed from a common oxindole starting material, thereby further streamlining structure–activity relationship studies.

Previous attempts to convert oxindoles into quinolinones are limited to substrates which often contain strongly electron-withdrawing groups, or other engineered starting materials, restricting the diversity of possible quinolinone products.^30−44^ Additionally, approaches to selectively access structurally different quinolinones from the same starting material through simple C1 insertion are currently lacking.

Herein we report a regiodivergent strategy to access quinolinones from oxindoles by using two distinct reaction conditions (Figure 1). We also report preliminary mechanistic experiments as well as synthetic applications to late diversification of bioactive compounds.

During our previous studies on thioether oxindoles,^45^ we serendipitously observed that upon subjecting oxindole **1aa** to a nickel-catalyzed transfer amination, a 4-substituted quinolinone **2a** was obtained rather than the free thiol (Figure 2A). Further investigation revealed that only lithium-bis(trimethylsilyl)amide (LiHMDS) and catalytic amounts of morpholine were required to enable this transition metal-free transformation. This observation drove us to design an analogous process using nucleofuges that are more established than thioethers, such as halogens. Indeed, identical reactivity could be observed without the need for added morpholine, further increasing the synthetic appeal of this process. To identify the ideal leaving group and gather some preliminary information about this novel reaction, we subjected the chloro-, bromo, and iodo-methylene substrates to the LiHMDS-mediated conditions and monitored their reaction profiles (Figure 2B). An increase in reactivity was observed for the heavier halogens, clearly indicating that the character of the leaving group plays a significant role in this transformation. Importantly, 3-substituted oxindoles could be directly converted into the corresponding iodomethylene derivatives through simple substitution with diiodomethane (see Supporting Information (SI), GP SM-A). Hence, we were now able to leverage this inexpensive and abundant material as a C1 building block for ring expansion. Further control reactions showed that HMDS bases were crucial to enable the reported ring expansion, with Li representing the superior counterion. Other lithium derived bases such as lithiumdiisopropylamide (LDA) or lithiumtetramethylpiperidide (LTMP) led to either no, or diminished, yields (Figure 2B). Upon further optimization of the reaction conditions (see SI, Table S1), 3-iodomethyl oxindole **1ab** could be converted to the 4-quinolinone **2a** with 1.5 equiv of LiHMDS in THF at 80 °C, yielding the product in 77% yield.

**Figure 2:**
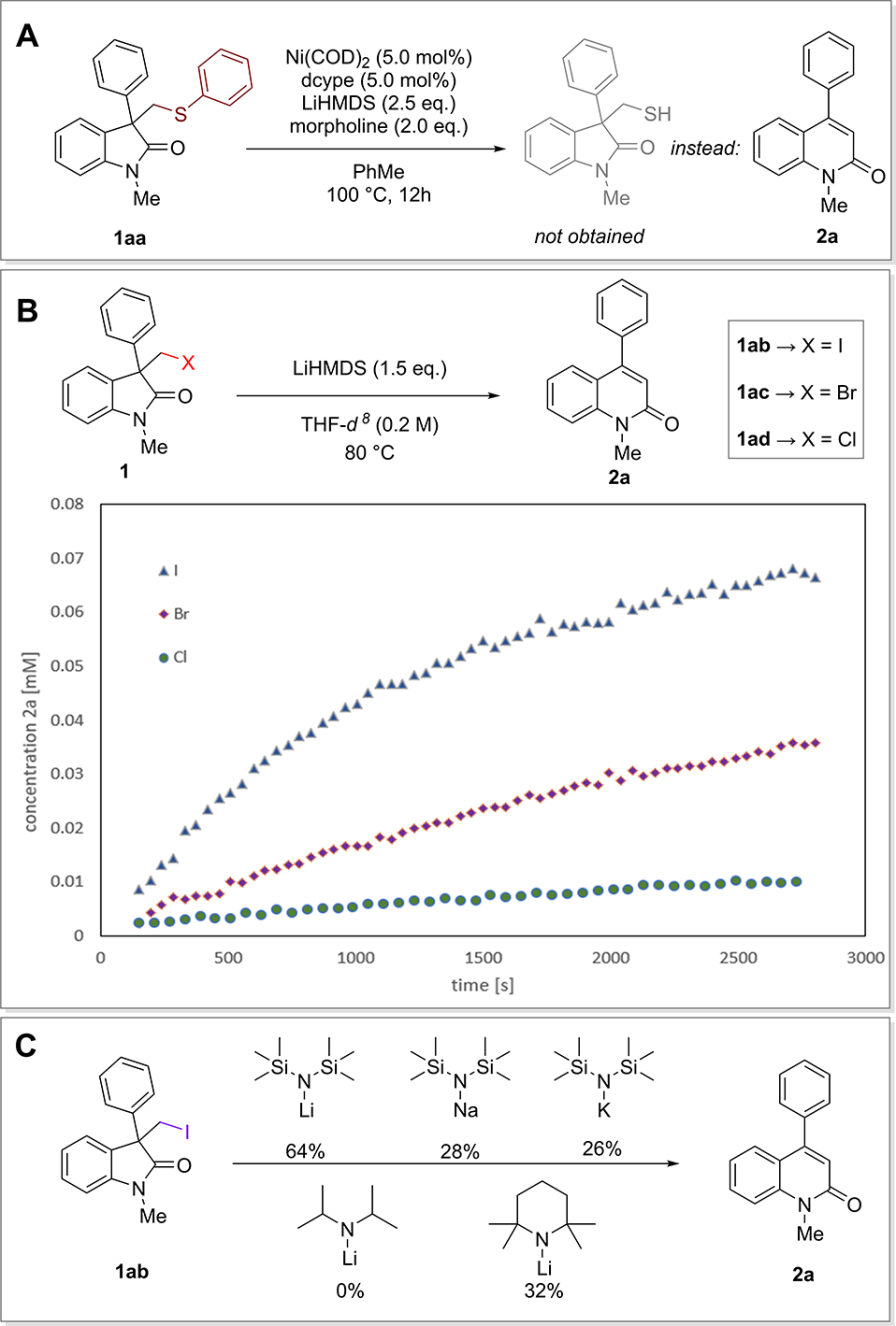
(A) Initial discovery; (B) Reaction profiles for different leaving groups X = I, Br, Cl; (C) Base dependence. For experimental details see SI.

With these optimized conditions in hand, we set out to evaluate the scope of the reaction. Initially, substituents on the aryl core of the oxindole were tested. A variety of functional groups of electron-donating (Me **2b**, OMe **2c**) or electron-withdrawing (F **2d** and **2e**, CF_3_**2f**) nature were tolerated, as well as the heterocycle containing derivatives **2g** and **2h**. Subsequently we tested several 3-substituted oxindoles as well as diverse substitution patterns on the amide nitrogen of the oxindole. Again, various functional groups were tolerated, yielding the desired products in good to excellent yields. 3-Alkyl substituted oxindoles, however, led to only deiodination of the starting materials. By exchanging the iodide with a thiomethyl functionality, the desired quinolinones **2s**, **2t**, and **2u** were obtained (Scheme 1C).

**Scheme 1:**
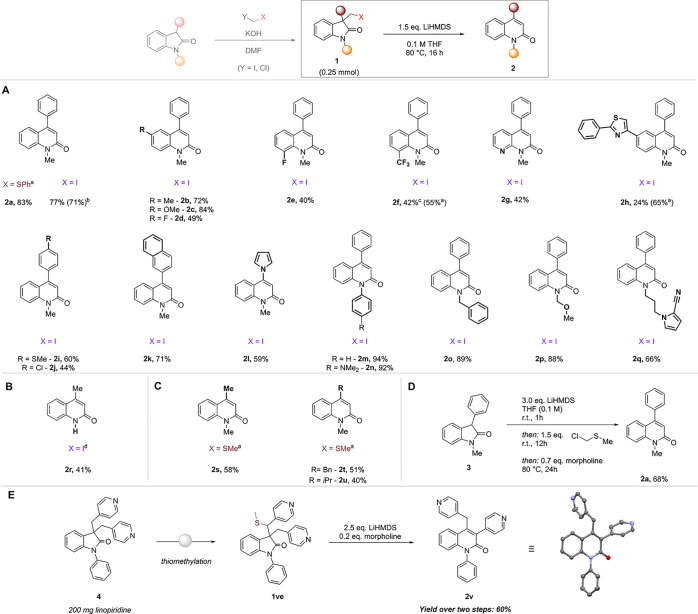
Scope of the LiHMDS Mediated Reaction of Oxindoles to 4-Quinolinones Addition of 0.2 equiv of morpholine. LiHMDS (1 M solution in THF). 24 h reaction time. Substrate obtained with different base than KOH. All yields are isolated yields after purification unless noted otherwise.

To further showcase the utility of the described reaction, we set out to probe a two-step, one-pot approach directly from a 3-substituted oxindole **3** (Scheme 1D). We were indeed able to isolate the desired product in only slightly reduced yield as compared to the stepwise procedure, highlighting the simplicity and applicability of our method for a straightforward late-stage skeletal transformation.

Finally, we probed the reaction conditions for the modification of a small molecule drug (Scheme 1E). We chose the cognitive enhancer linopiridine as a suitable target.^47^ Upon introduction of a thiomethyl group in the oxindole starting material, we were indeed able to obtain the disubstituted quinolinone (**2v**) in 60% yield over two steps, starting from 200 mg of linopiridine. The structure of the product was confirmed by X-ray analysis.

To investigate the mechanism of the reported reaction, we conducted several control experiments. Since a strong dependence of the reaction on the employed base was observed (Figure 2C), we chose to examine the intermolecular interaction of the latter with the substrate molecule. We were able to crystallize the corresponding substrate-reagent complex **5** (Figure 3A). The obtained dimer revealed a coordination of the employed base to the carbonyl oxygen of the substrate. Similar interactions of lithium bases and amides were prominently reported by the Szostak,^48−50^ Collum,^51,52^ and Hevia groups,^53^ which exploit this interplay for the activation of the amide moiety, enabling subsequent nucleophilic attacks or deprotonations.

Upon examination of the reaction mixture of the N–H oxindole **1rb** we were able to isolate the urea derivative **6** (Figure 3B). Mechanistically, the formation of this byproduct can be rationalized through the formation of an intermediary isocyanate following an intramolecular elimination cascade upon deprotonation, as previously proposed.^31,37,38^ Subsequent cyclization results in the formation of the desired product.^54−56^ Alternatively, in a side-reaction, the isocyanate intermediate can be nucleophilically attacked by LiHMDS, followed by hydrolysis in the work-up, explaining the formation of the observed urea.^57^

**Figure 3:**
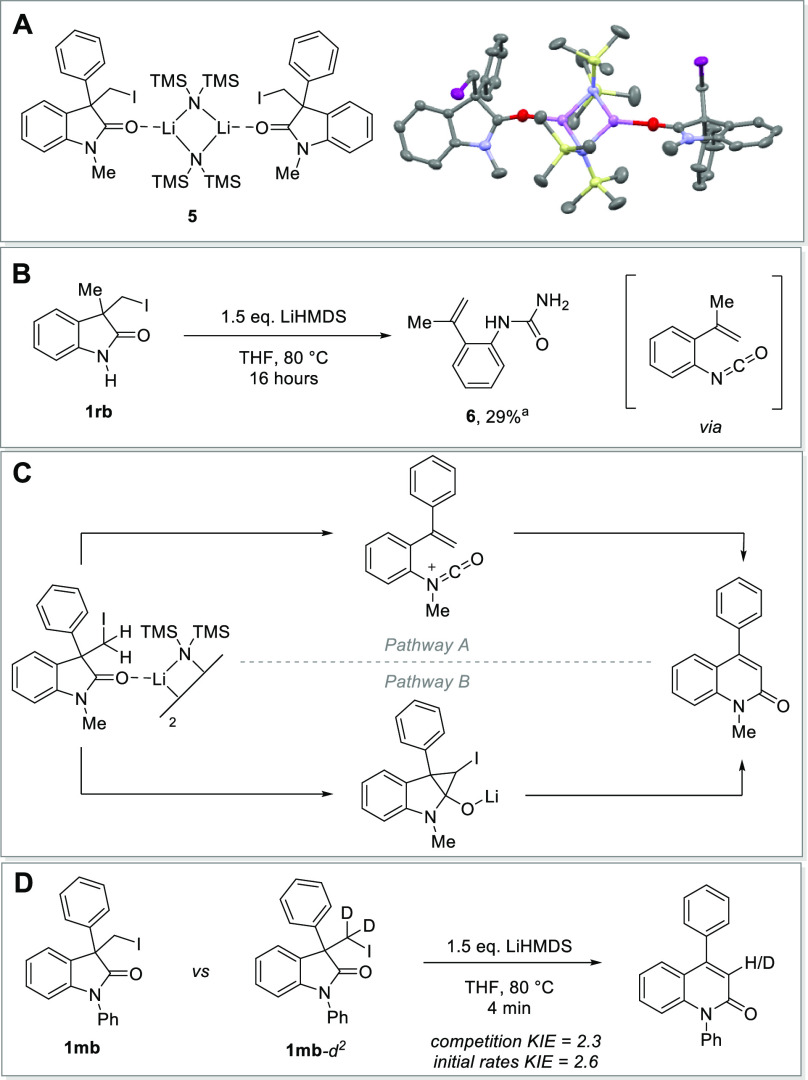
Mechanistic experiments. (A) structure of the substrate-reagent complex; (B) Isolated urea byproduct; (C) Proposed mechanistic pathways. (D) Competitive KIE experiment. ^*a*^Yield of the isolated product.

For the *N*-substituted oxindoles a similar mechanistic proposal would result in the formation of a highly energetic cationic isocyanate intermediate (Figure 3C, pathway A). The latter was previously proposed as a reactive intermediate in the reaction of carbamoyl chlorides and fluorides with olefins by the Lautens^58,59^ and Takemoto groups.^60^

Another mechanism involving deprotonation of the nonenolizable iodomethylene moiety followed by addition to the carbonyl forming a cyclopropyl alcoholate intermediate can alternatively be envisioned (Figure 3C, pathway B). Such cyclopropanol formation was previously observed upon lithium–halogen exchange of bromo oxindoles with *t*-BuLi.^61^ Subsequent fragmentation of the halogen-bearing cyclopropanol, in line with previous reports,^62−88^ could result in our case in the desired quinolinone. To distinguish between these two mechanistic pathways, we performed a series of experiments. In the case of the cationic isocyanate intermediate LiHMDS would have to engage the amide as a Lewis acid to enable the corresponding cleavage of the C_2_–C_3_-bond. However, since no other, more common Lewis acid could enable the desired reactivity (SI, Table S2) and Hammett analysis did not indicate a positive charge build-up on the nitrogen (see SI, Figure S2), such a mechanism seems less likely. Subsequently, we measured the KIE both in a competition experiment and by comparing initial rates (Figure 3D). The experimentally observed KIE (2.3 resp. 2.6) shows that C–H cleavage is rate-determining, an observation most consistent with an anionic mechanism when using *N*-substituted oxindoles as substrates. However, the fact that LiHMDS performed much better than comparatively stronger bases, which are commonly utilized in deprotonation reactions of alkyl halides, warrants additional future studies to understand the privileged role played by LiHMDS in our reaction, most notably with regard to potential complex-induced proximity effects (CIPE).^65,66^ This observation, along with the fact that deprotonative metalation of nonenolizable carbonyl moieties has not yet been utilized in ring-expansion reactions,^67−73^ is poised to stimulate new developments in this area.

We next questioned whether a different mechanistic approach might enable us to divert the reactivity toward the regioisomeric 3-substituted quinolinone starting from the same starting materials. This would complement the previous protocol and overall unlock a rare example of a regiodivergent skeletal editing process. We envisioned that the generation of a carbon centered cation on the methyl-group might lead to a rapid Friedel–Crafts type ring expansion (see Table 1).^74−76^ A similar mechanism was previously proposed by Huang et al. in their metathesis of *para*-quinone methides with 3-diazo oxindoles.^77^ Indeed, we observed that upon treatment of starting material **1ab** with AgBF_4_ as a halogen scavenger, the desired 3-substituted quinolinone **2a** could be obtained in excellent yield (Scheme 2). In order to accommodate for substrates that necessitate the incorporation of a thioether rather than a halogen as a leaving group in the LiHMDS mediated reaction, we tested copper-catalyzed conditions to generate the corresponding sulfonium, which should behave as a comparable nucleofuge. Indeed, the thioethers could be transformed into the desired quinolinones in only slightly reduced yield. A variety of electron-withdrawing and -donating functional groups on the oxindole core were tolerated. However, as was established by previous work on Friedel–Crafts type reactions,^78−80^ the efficiency of the reaction decreased with electron-poor oxindoles.

**Table 1:**
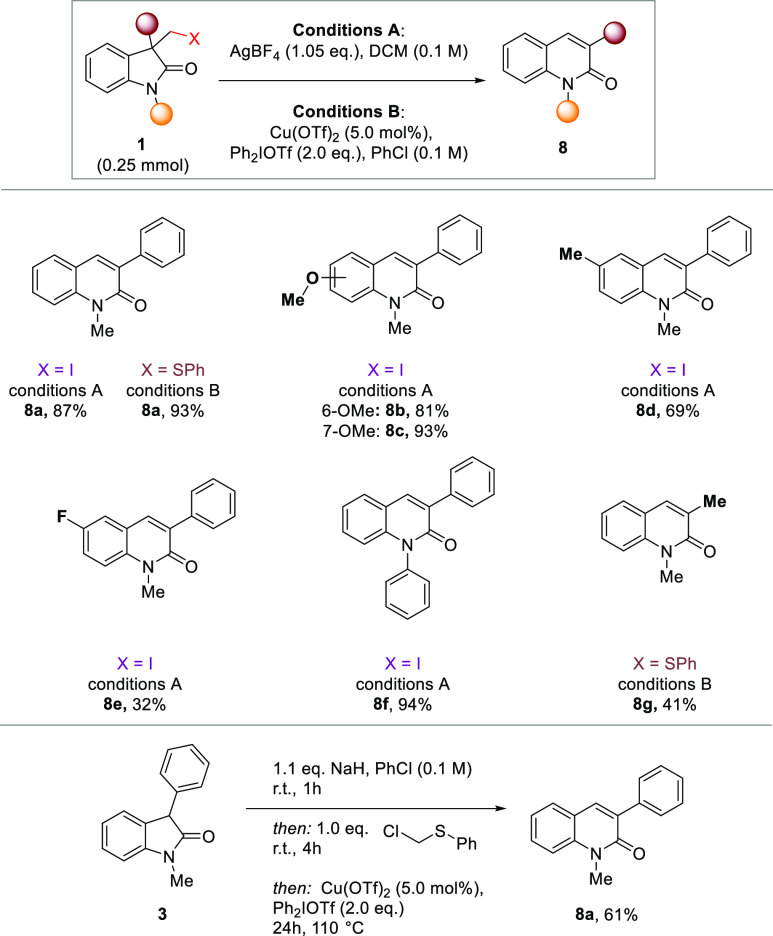
Scope of the Ring Expansion from Oxindoles to 3-Quinolinones^a^

a Yields of isolated product.

**Scheme 2:**
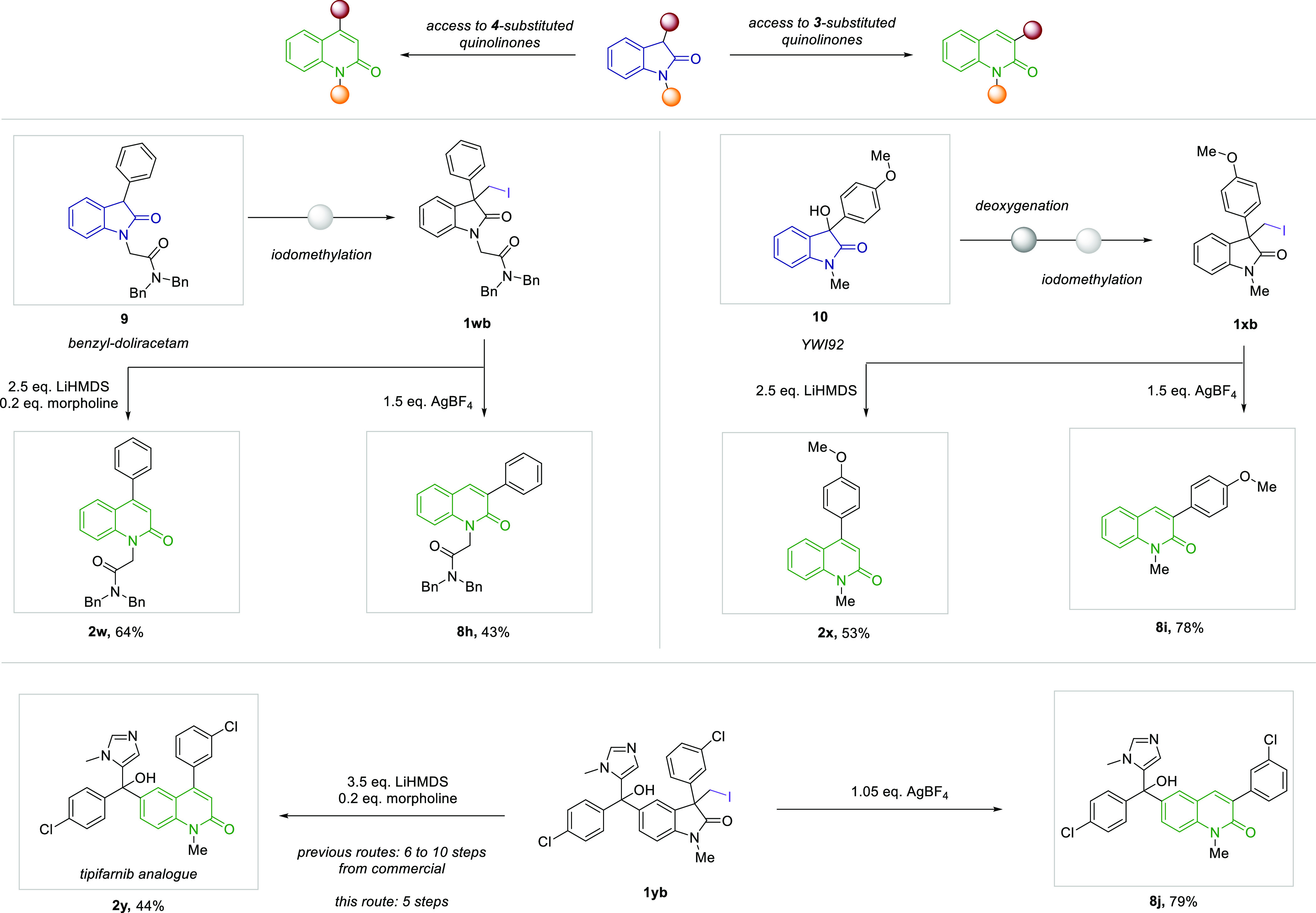
Scope of Late Stage Regiodivergent Quinolinone Synthesis from Bioactive Oxindoles, and Synthesis of a Quinolinone Drug and Its Regioisomer Yield of isolated product on a 0.25 mmol scale.

Finally, we aimed to synthesize regiodivergent quinolinones from biologically active oxindoles. Doliracetam, an oxindole drug used for the treatment of epilepsy,^81^ could be converted in its protected form through simple substitution with diiodomethane to the corresponding iodomethyl-oxindole **1vb**. Subjecting this derivative to both of our reaction conditions gave the desired quinolinones in good yields. This example demonstrates that our method can be used for the late-stage skeletal transformation of oxindole drugs and showcases the tolerance of amides and acidic α-protons for both of our presented methods. Next, YWI92 (**10**), a seizure suppressant,^82^ which consists of a 3-hydroxy-3-substituted oxindole, another prominent structural motif in the treatment of a variety of diseases,^27,83,84^ was transformed into the iodomethyl oxindole starting material **1wb** through straightforward deoxygenation and substitution. We again were able to obtain the desired regiodivergent quinolinones in excellent yields. Lastly, we aimed to showcase the applicability of our methods to the synthesis of quinolinone drugs. We chose the tipifarnib analogue **2y** as a suitable target. Upon synthesis of its oxindole derivative **11** (see SI) we were indeed able to obtain the desired derivative **2y**, as well as its isomer **8j**, further indicating the tolerance of both reaction conditions for unprotected alcohols and imidazole heterocycles. To the best of our knowledge this route offers access to the tipifarnib analogue with the least number of steps from commercial starting materials,^85−87^ while also granting the possibility to probe its regioisomer and the oxindole analogue in structure–relationship studies.

In conclusion, we have reported a rare example of regiodivergent skeletal editing of oxindoles to quinolinones. We were able to show the compatibility of our developed methods for the late-stage functionalization of bioactive oxindoles, as well as their potential in the synthesis of quinoline derivatives.

## Supporting Information Available

Experimental procedures and characterization data for all compounds (PDF)
