# Supplementary material for: Regiodivergent Ring-Expansion of Oxindoles to Quinolinones
Source: J Am Chem Soc. 2024 Feb 9;146(7):4301–8. doi: 10.1021/jacs.3c12119 (PMC10885155; doi:10.1021/jacs.3c12119)
Supplement: Supplementary file 1 — ja3c12119_si_001.pdf [file ja3c12119_si_001.pdf]

Regiodivergent Ring-Expansion of Oxindoles to Quinolinones

Hendrik L. Schmitt,[a] Den Martymianov,[a] Ori Green,[a] Tristan Delcaillau,[a] Young Seo Park Kim,[a] and Bill Morandi*[a]

AUTHOR ADDRESS [a] H. L. Schmitt, D. Martymianov, O. Green, T. Delcaillau, Y. S. Park Kim, Bill Morandi.
Laboratorium für Organische Chemie, ETH Zürich
Vladimir-Prelog-Weg 3, HCI, 8093 Zürich, Switzerland

E-mail: bill.morandi@org.chem.ethz.ch

Table of Contents

General Information 3
Optimization 4
Synthesis of starting materials 7
General Procedure SM-A 7
General Procedure SM-B 16
Substrates according to known procedures 19
Other substrates 20
Substrate scope 26
General procedure A 26
Two step one pot procedure 1 36
General procedure B 37
General procedure C 37
Two step one pot procedure 2 41
Drug derived molecules 42
Linopiridine 42
Doliracetam 44
YWI92 46
Tipifarnib 48
Mechanistic experiments 52
KIE 54
Hammett analysis 58
Reaction profiles 62
X-ray data 63
References 70
NMR Spectra 73
Starting materials 73
Scope reaction A 110
Scope reaction B 134
Linopiridine 142
Doliracetam 144
YWI92 148

Tipifarnib 151
Mechanistic experiments 155

General Information

Compound naming The compound name gives direct information about the employed leaving groups. The first letter for methylene-oxindoles (**1**) correspond to the order in which they appear in the main text of this work. The second letter refers to the employed leaving group with **a** = SPh, **b** = I, **c** = Br, **d** = Cl, **e** = SMe.

General Methods All cyclization reactions were performed in an argon atmosphere glovebox (LABmaster Pro SP, MBraun). The substrates and reagents for catalytic reactions were degassed and stored in the glovebox.

Chemicals Bis(dibenzylideneacetone)palladium and XPhos were purchased from Fluorochem. $AgBF_4$ was purchased from Apollo Scientific, LiHMDS was purchased from SigmaAldrich, diiodomethane was bought from ABCR GmBH, acetonitrile (99.9% extra dry over molecular sieve, AcroSeal®), THF (99.9% extra dry over molecular sieve, AcroSeal®), *N*,*N*-dimethylformamide (99.9% extra dry over molecular sieve, AcroSeal®), and DCM (99.9% extra dry over molecular sieve, AcroSeal®), were purchased from Acros Organics. They were used without further purification.

NMR Spectroscopy Proton nuclear magnetic resonance (^{1}H NMR) spectra were acquired on commercial instruments (400 MHz) at the NMR facility of ETH Zürich. Carbon-13 nuclear magnetic resonance (^{13}C NMR) spectra (1H-broadband decoupled) were acquired at 126 or 101 MHz. Fluorine-19 nuclear magnetic resonance (^{19}F NMR) spectra were acquired at 376 MHz. ^{1}H NMR spectra are reported as follows: chemical shift, multiplicity (s = singlet, d = doublet, dd = doublet of doublets, t = triplet, q = quadruplet, p = pentett and m = multiplet), coupling constant (J values) in Hz and integration. Chemical shifts (δ) were reported with respect to the corresponding solvent residual peak at (7.26 ppm for $CDCl_3$, 2.50 ppm for DMSO-d_6) for ^{1}H NMR spectra. ^{13}C NMR spectra are reported in ppm using the solvent peak of $CDCl_3$ (77.16 ppm) or the solvent peak of DMSO-d_6 (39.52 ppm) as reference.

High-resolution mass spectrometry (HRMS) HRMS data were provided by the mass spectrometry service in the Laboratorium für Organische Chemie at ETH Zürich. The molecular ion $[M]^+$, $[M+H]^+$, $[M+K]^+$ and $[M+Na]^+$ respectively or the anion are given in m/z units.

Purification Methods Analytical thin layer chromatography was performed on silica gel coated glass plates (0.25 mm) with fluorescence indicator UV254 (Merck, 0.25 mm silica gel Si 60, UV254). For detection of spots, irradiation with UV light at 254 nm or oxidative staining using potassium permanganate solution ($KMnO_4$) was used. column chromatography was conducted with silica gel 60 (particle size 40–63 µM, Silicycle) at room temperature and under elevated pressure.

Optimization

Table S1. Optimization studies

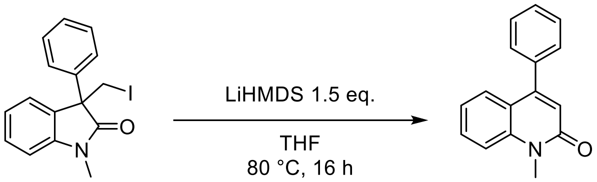

entry	**Derivation from standard conditions**	**yield (%)[a]**
1	none	64
2	KHMDS instead of LiHMDS	26
3	LiO*t*Bu instead of LiHMDS	0
4	Li_2CO_3 instead of LiHMDS	0
5	LDA instead of LiHMDS	0
6	Li-TMP instead of LiHMDS	32
7	NaHMDS instead of LiHMDS	28
8	toluene instead of THF	21
9	1,4-dioxane instead of THF	54
10	0.5 M instead of 0.2 M	64
11	1.0 M instead of 0.2 M	45
**12**	**0.1 M instead of 0.2 M**	**73**
13	2.5 eq. LiHMDS	62
14	1.0 eq. LiHMDS	55
15	0.5 eq. LiHMDS	23
16	LDA + 20 mol% morpholine instead of LiHMDS	12

Standard conditions: 0.05 mmol starting material, 1.5 eq. LiHMDS in 0.25 mL THF (0.2 M) [a] ^{1}H-NMR yield with 1,3,5-trimethoxybenzene as internal standard

Table S2. Optimization studies

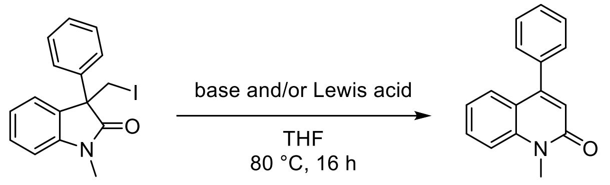

entry	**Additives**	**yield (%)**[a]
1	1.5 eq. KO*t*Bu	0
2	1.5 eq. K_2CO_3	0
3	1.0 eq. $FeCl_2$ + 1.5 eq. KO*t*Bu	0
4	1.0 eq. $FeCl_3$ +1.5 eq. KO*t*Bu	0
5	1.0 eq. LiOTf + 1.5 eq. KO*t*Bu	0
6	1.0 eq. BPh_3 + 1.5 eq. KO*t*Bu	0
7	8.0 eq. HFIP in toluene, 100 °C	0
8	1.0 eq. $MeSO_3H$	0
9	1.0 eq. $FeCl_2$	0
10	1.0 eq. $FeCl_3$	0
11	1.0 eq. $ScOTf_3$	0
12	1.0 eq. BPh_3	0
13	1.0 eq. LiO*t*Bu	0
14	1.0 eq. LiCl	0
15	$BF_3 \cdot OEt_2$	0

Standard conditions: 0.05 mmol starting material, in 0.25 mL THF (0.2 M) [a] ^{1}H-NMR yield with 1,3,5-trimethoxybenzene as internal standard

Table S3. Amine screens for thiomethyl substrates

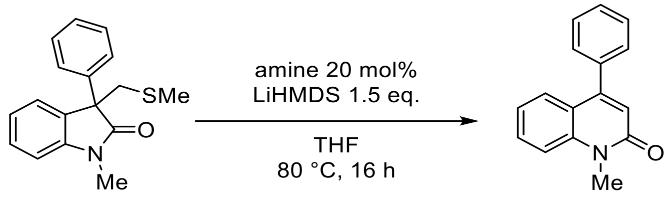

entry	**Amine**	**yield (%)[a]**
1	morpholine	74
2	dibenzylamine	60
3	diethylamine	11
4	aniline	11
5	triethylamine	trace

Standard conditions: 0.1 mmol starting material, 0.2 eq. amine, 1.5 eq. LiHMDS in 0.5 mL THF (0.1 M) [a] ^{1}H-NMR yield with 1,3,5-trimethoxybenzene as internal standard

Synthesis of starting materials

General Procedure SM-A

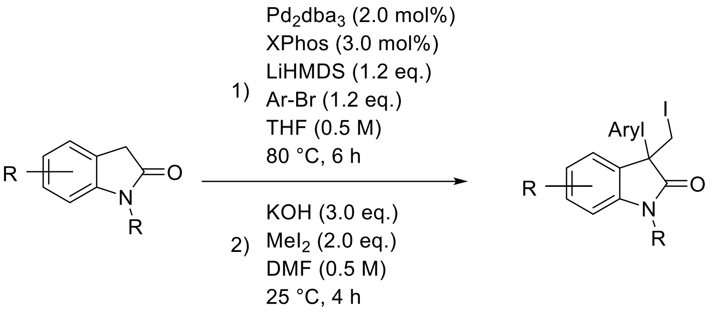

The starting materials were synthesized according to modified procedures from the literature.[1,2]

A flame-dried 16 mL drum vial in a glovebox was charged with oxindole (1.00 eq.), XPhos (0.03 eq.), $Pd(dba)_2$ (0.02 eq.), and a magnetic stir bar. The vial was sealed with a septum-cap and removed from the glovebox. Then, aryl bromide (1.20 eq.) and THF (0.5 M) were added through the septum. The mixture was heated to 70 °C for 5 min, then LiHMDS (1 M in THF, 1.20 eq.) was added. The resulting mixture was placed on a heated stirring plate at 70 °C and stirred for 6 h. Upon cooling to room temperature, the mixture was filtered through a plug of silica and washed with EtOAc. Volatiles were removed *in vacuo.* The resulting crude mixture was redissolved in DMF (0.5 M) and diiodomethane (2.00 eq.) was added. The reaction was degassed through continuous bubbling with nitrogen. Then, KOH (3.00 eq.) was added. The resulting mixture was stirred for 4 h at room temperature. Equivalent amounts of water and EtOAc were added, and the organic phase collected. The aqueous phase was washed with EtOAc (3 x). The combined organic residues were dried over $MgSO_4$ and volatiles removed *in vacuo.* The obtained crude residue was purified by column chromatography.

3-(Iodomethyl)-1-methyl-3-phenylindolin-2-one (1ab)

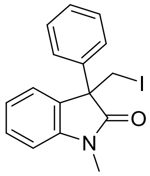

The title compound was obtained according to general procedure **SM-A** from 1-methyloxindole (442 mg, 3.00 mmol), and bromobenzene. Purification by column chromatography (SiO_2, 8% EtOAc in pentane) afforded **1ab** as a white solid (716 mg, 1.97 mmol, 66%).

^{1}H-NMR (400 MHz, $CDCl_3$) δ 7.49 – 7.36 (m, 4H), 7.35 – 7.28 (m, 3H), 7.20 (td, *J* = 7.6, 1.0 Hz, 1H), 6.94 (dd, *J* = 7.8, 0.8 Hz, 1H), 4.04 (d, *J* = 9.8 Hz, 1H), 3.77 (d, *J* = 9.8 Hz, 1H), 3.25 (s, 3H).

^{13}C{1H} NMR (101 MHz, $CDCl_3$) δ 176.3, 144.2, 137.8, 131.0, 129.3, 128.9, 128.2, 127.3, 125.1, 122.9, 108.8, 56.8, 26.7, 10.7.

The spectroscopic data matched those reported in the literature.[3]

3-(Iodomethyl)-1,5-dimethyl-3-phenylindolin-2-one (1bb)

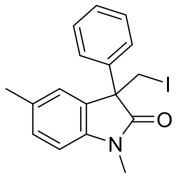

The title compound was obtained according to general procedure **SM-A** from 1,5-dimethylindolin-2-one (340 mg, 2.11 mmol), and bromobenzene. Purification by column chromatography (SiO_2, 0 to 10% EtOAc in hexane) afforded **1bb** as a white solid (387 mg, 1.03 mmol, 49%).

^{1}H-NMR (400 MHz, $CDCl_3$) δ 7.47 – 7.39 (m, 2H), 7.35 – 7.27 (m, 3H), 7.23 – 7.15 (m, 2H), 6.83 (d, *J* = 7.9 Hz, 1H), 4.03 (d, *J* = 9.7 Hz, 1H), 3.76 (d, *J* = 9.7 Hz, 1H), 3.23 (s, 3H), 2.41 (d, *J* = 0.8 Hz, 3H).

^{13}C{1H} NMR (101 MHz, $CDCl_3$) δ 176.2, 141.7, 137.9, 132.3, 130.9, 129.4, 128.8, 128.0, 127.1, 125.6, 108.3, 56.7, 26.6, 21.3, 10.7.

The spectroscopic data matched those reported in the literature.[3]

3-(Iodomethyl)-5-methoxy-1-methyl-3-phenylindolin-2-one (1cb)

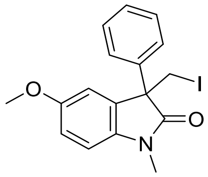

The title compound was obtained according to general procedure **SM-A** from 5-methoxy-1-methylindolin-2-one (444 mg, 2.51 mmol), and bromobenzene. Purification by column chromatography (SiO_2, 0 to 30% EtOAc in hexane) afforded **1cb** as an off-white solid (512 mg, 1.30 mmol, 52%).

^{1}H-NMR (400 MHz, $CDCl_3$) δ 7.46 – 7.40 (m, 2H), 7.35 – 7.27 (m, 3H), 7.00 (d, *J* = 2.5 Hz, 1H), 6.94 (dd, *J* = 8.5, 2.5 Hz, 1H), 6.84 (d, *J* = 8.4 Hz, 1H), 4.02 (d, *J* = 9.8 Hz, 1H), 3.84 (s, 3H), 3.75 (d, *J* = 9.8 Hz, 1H), 3.22 (s, 3H).

^{13}C{1H} NMR (101 MHz, $CDCl_3$) δ 176.0, 156.2, 137.8, 137.7, 132.3, 128.9, 128.2, 127.2, 113.4, 112.6, 109.0, 57.1, 56.0, 26.8, 10.6.

HRMS: m/z $[M+H]^+$ calcd. for: $C_{17}H_{17}INO_2$ 394.0299, found: 394.0297.

3-(iodomethyl)-6-methoxy-1-methyl-3-phenylindolin-2-one (1cb-isomer)

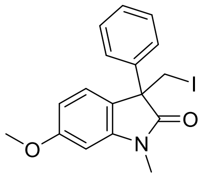

The title compound was obtained according to general procedure **SM-A** from 6-methoxy-1-methylindolin-2-one (200 mg, 1.13 mmol), and bromobenzene. Purification by column chromatography (SiO_2, 0 to 30% EtOAc in hexane) afforded **1cb** as a white foam (110 mg, 0.28 mmol, 25%).

^{1}H-NMR (400 MHz, $CDCl_3$) δ 7.52 – 7.40 (m, 2H), 7.34 – 7.27 (m, 4H), 6.69 (dd, *J* = 8.2, 2.3 Hz, 1H), 6.51 (d, *J* = 2.3 Hz, 1H), 4.02 (d, *J* = 9.7 Hz, 1H), 3.88 (s, 3H), 3.74 (d, *J* = 9.7 Hz, 1H), 3.22 (s, 3H).

^{13}C{1H} NMR (101 MHz, $CDCl_3$) δ 176.8, 161.0, 145.4, 138.1, 128.9, 128.2, 127.3, 125.8, 122.8, 106.7, 96.6, 56.3, 55.7, 26.7, 11.5.

HRMS: m/z $[M+Na]^+$ calcd. for: C17H16INNaO2 416.0118, found: 416.0111.

5-Fluoro-3-(iodomethyl)-1-methyl-3-phenylindolin-2-one (1db)

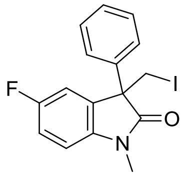

The title compound was obtained according to general procedure **SM-A** from 5-fluoro-1-methylindolin-2-one (425 mg, 2.58 mmol), and bromobenzene. Purification by column chromatography (SiO_2, 8% EtOAc in pentane) afforded **1db** as a off-white solid (520 mg, 1.37 mmol, 53%).

^{1}H-NMR (400 MHz, $CDCl_3$) δ 7.46 – 7.38 (m, 2H), 7.36 – 7.28 (m, 3H), 7.18 – 7.05 (m, 2H), 6.86 (m, 1H), 4.02 (d, *J* = 9.9 Hz, 1H), 3.74 (d, *J* = 9.9 Hz, 1H), 3.24 (s, 3H).

^{13}C{1H} NMR (101 MHz, $CDCl_3$) δ 176.1, 159.4 (d, *J* = 241.5 Hz), 140.2 (d, *J* = 2.1 Hz), 137.3, 132.6 (d, *J* = 8.0 Hz), 129.1, 128.4, 127.1, 115.6 (d, *J* = 23.5 Hz), 113.2 (d, *J* = 25.1 Hz), 109.3 (d, *J* = 8.1 Hz), 57.2 (d, *J* = 1.7 Hz), 26.9, 9.9.

^{19}F{1H} NMR (376 MHz, $CDCl_3$) δ -119.6

The spectroscopic data matched those reported in the literature.[3]

7-Fluoro-3-(iodomethyl)-1-methyl-3-phenylindolin-2-one (1eb)

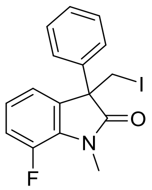

The title compound was obtained according to general procedure **SM-A** from 7-fluoro-1-methylindolin-2-one (584 mg, 3.54 mmol), and bromobenzene. Purification by column chromatography (SiO_2, 0 to 15% EtOAc in hexane) afforded **1eb** as an off-white solid (698 mg, 1.83 mmol, 52%).

^{1}H-NMR (400 MHz, $CDCl_3$) δ 7.44 – 7.39 (m, 2H), 7.36 – 7.29 (m, 3H), 7.17 – 7.10 (m, 3H), 4.05 (d, *J* = 9.8 Hz, 1H), 3.72 (d, *J* = 9.8 Hz, 1H), 3.46 (d, *J* = 2.8 Hz, 3H).

^{13}C{1H} NMR (101 MHz, $CDCl_3$) δ 176.0, 148.22 (d, *J* = 244.3 Hz), 137.4, 133.8 (d, *J* = 3.2 Hz), 131.0 (d, *J* = 8.5 Hz), 129.0, 128.4, 127.1, 123.4 (d, *J* = 6.4 Hz), 120.9 (d, *J* = 3.3 Hz), 117.2 (d, *J* = 19.3 Hz), 57.1, 29.2 (d, *J* = 6.0 Hz), 10.1.

^{19}F{1H} NMR (376 MHz, $CDCl_3$) δ -135.7

HRMS: m/z $[M+Na]^+$ calcd. for: $C_{16}H_{13}FINNaO$ 403.9918, found: 403.9911.

3-(Iodomethyl)-1-methyl-3-phenyl-1,3-dihydro-2H-pyrrolo[2,3-b]pyridin-2-one (1gb)

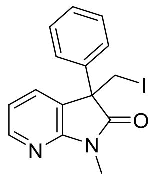

The title compound was obtained according to general procedure **SM-A** from 1-methyl-1,3-dihydro-*2H*-pyrrolo[2,3-b]pyridin-2-one (271 mg, 1.83 mmol), and bromobenzene. Purification by column chromatography (SiO_2, 0 to 10% EtOAc in hexane) afforded **1eb** as a off-white solid (164 mg, 0.45 mmol, 25%).

^{1}H-NMR (400 MHz, $CDCl_3$) δ 8.32 (ddd, *J* = 5.3, 1.6, 0.5 Hz, 1H), 7.68 (ddd, *J* = 7.3, 1.6, 0.5 Hz, 1H), 7.49 – 7.39 (m, 2H), 7.39 – 7.29 (m, 3H), 7.10 (ddd, *J* = 7.4, 5.3, 0.5 Hz, 1H), 3.98 (d, *J* = 10.0 Hz, 1H), 3.79 (d, *J* = 9.9 Hz, 1H), 3.33 (d, *J* = 0.5 Hz, 3H).

^{13}C{1H} NMR (101 MHz, $CDCl_3$) δ 169.3, 157.4, 148.2, 136.8, 132.7, 129.1, 128.6, 127.1, 125.7, 118.4, 56.5, 25.9, 9.8.

HRMS: m/z $[M+H]^+$ calcd. for: $C_{15}H_{14}IN_2O$ 365.0145, found: 365.0146.

3-(Iodomethyl)-1-methyl-3-phenyl-5-(2-phenylthiazol-4-yl)indolin-2-one (1hb)

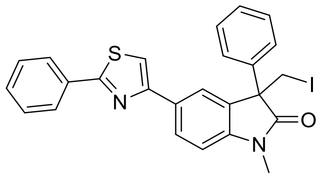

The title compound was obtained according to general procedure **SM-A** from 1-methyl-5-(2-phenylthiazol-4-yl)indolin-2-one (919 mg, 2.98 mmol), and bromobenzene. Purification by column chromatography (SiO_2, 0 to 15% EtOAc in hexane) afforded **1hb** as an off-white solid (444 mg, 0.85 mmol, 28%).

^{1}H-NMR (400 MHz, $CDCl_3$) δ 8.09 (dd, *J* = 8.1, 1.7 Hz, 1H), 8.07 – 8.00 (m, 3H), 7.53 – 7.41 (m, 6H), 7.39 – 7.29 (m, 3H), 7.02 (d, *J* = 8.2, 1H), 4.12 (d, *J* = 9.8 Hz, 1H), 3.85 (d, *J* = 9.7 Hz, 1H), 3.29 (s, 3H).

^{13}C{1H} NMR (101 MHz, $CDCl_3$) δ 176.5, 168.2, 156.0, 144.2, 137.7, 133.8, 131.5, 130.3, 130.0, 129.1, 129.0, 128.3, 127.6, 127.4, 126.8, 123.3, 111.8, 108.9, 57.0, 26.9, 10.5.

HRMS: m/z $[M+H]^+$ calcd. for: $C_{25}H_{20}IN_2OS$ 523.0336, found: 523.0329.

3-(Iodomethyl)-1,5-dimethyl-3-phenylindolin-2-one (1ib)

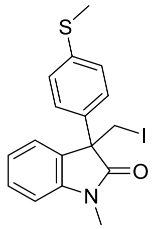

The title compound was obtained according to general procedure **SM-A** from 1-methylindolin-2-one (442 mg, 3.00 mmol), and 1-bromo-4-(methylthio)benzene. Purification by column chromatography (SiO_2, 0 to 10% EtOAc in hexane) afforded **1ib** as an off-white solid (583 mg, 1.42 mmol, 48%).

^{1}H-NMR (400 MHz, $CDCl_3$) δ 7.42 (td, *J* = 7.7, 1.3 Hz, 1H), 7.39 – 7.34 (m, 3H), 7.22 – 7.14 (m, 3H), 6.94 (ddd, *J* = 7.8, 0.9, 0.5 Hz, 1H), 3.99 (d, *J* = 9.8 Hz, 1H), 3.73 (d, *J* = 9.8 Hz, 1H), 3.23 (s, 3H), 2.44 (s, 3H).

^{13}C{1H} NMR (101 MHz, $CDCl_3$) δ 176.2, 144.1, 138.9, 134.4, 130.7, 129.3, 127.7, 126.7, 125.0, 122.9, 108.8, 56.3, 26.7, 15.7, 10.6.

HRMS: m/z for $C_{17}H_{16}INNaOS$ $[M+Na]^+$ calcd.: 431.989, found: 431.9888.

3-(4-Chlorophenyl)-3-(iodomethyl)-1-methylindolin-2-one (1jb)

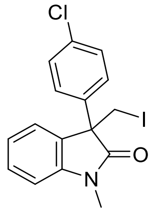

The title compound was obtained according to general procedure **SM-A** from 1-methylindolin-2-one (442 mg, 3.00 mmol), and 4-chlorobromobenzene. Purification by column chromatography (SiO_2, 0 to 10% EtOAc in hexane) afforded **1jb** as an off-white solid (535 mg, 1.35 mmol, 45%).

^{1}H-NMR (400 MHz, $CDCl_3$) δ 7.43 (td, *J* = 7.7, 1.3 Hz, 1H), 7.41 – 7.36 (m, 3H), 7.31 – 7.26 (m, 2H), 7.21 (td, *J* = 7.6, 1.0 Hz, 1H), 6.95 (dt, *J* = 7.8, 0.8 Hz, 1H), 3.96 (d, *J* = 9.8 Hz, 1H), 3.73 (d, *J* = 9.8 Hz, 1H), 3.24 (s, 3H).

^{13}C{1H} NMR (101 MHz, $CDCl_3$) δ 175.8, 144.0, 136.2, 134.2, 130.3, 129.4, 128.9, 128.6, 125.0, 122.9, 108.8, 56.0, 26.6, 10.2.

HRMS: m/z for C16H13ClINNaO $[M+Na]^+$ calcd.: 419.9623, found: 419.9618.

3-(Iodomethyl)-1-methyl-3-(naphthalen-2-yl)indolin-2-one (1kb)

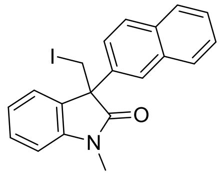

The title compound was obtained according to general procedure **SM-A** from 1-methylindolin-2-one (442 mg, 3.00 mmol), and 2-bromonaphthalene. Purification by column chromatography (SiO_2, 0 to 10% EtOAc in hexane) afforded **1kb** as a white-brownish solid (788 mg, 1.91 mmol, 64%).

^{1}H-NMR (400 MHz, $CDCl_3$) δ 7.87 – 7.74 (m, 4H), 7.62 (dd, *J* = 8.8, 2.0 Hz, 1H), 7.49 – 7.40 (m, 4H), 7.26 – 7.21 (m, 1H), 6.98 (d, *J* = 8.1, 1H), 4.14 (d, *J* = 9.8 Hz, 1H), 3.88 (d, *J* = 9.8 Hz, 1H), 3.28 (s, 3H).

^{13}C{1H} NMR (101 MHz, $CDCl_3$) δ 176.3, 144.2, 135.2, 133.2, 132.9, 131.1, 129.4, 128.8, 128.4, 127.6, 126.6, 126.5, 126.4, 125.2, 124.9, 123.0, 108.9, 56.9, 26.7, 10.5.

HRMS: m/z for $C_{20}H_{16}INNaO$ $[M+Na]^+$ calcd.: 436.0169, found: 436.0168.

3-(Iodomethyl)-1-methyl-3-(1H-pyrrol-1-yl)indolin-2-one (1lb)

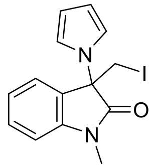

The title compound was obtained according to general procedure **SM-A**, however, instead of the alpha-arylation, **SM-2** was utilized directly in the diiodomethane substitution.

SM-2 (637 mg, 3.00 mmol) was subjected to the standard substation procedure of **SM-A**. Purification by column chromatography (SiO_2, 0 to 10% EtOAc in hexane) afforded **1lb** as a white solid (367 mg, 1.04 mmol, 35%).

^{1}H-NMR (400 MHz, $CDCl_3$) δ 7.79 – 7.56 (m, 1H), 7.49 (td, *J* = 7.8, 1.3 Hz, 1H), 7.23 (td, *J* = 7.6, 1.0 Hz, 1H), 6.95 (dt, *J* = 7.9, 0.8 Hz, 1H), 6.88 (t, *J* = 2.2 Hz, 2H), 6.19 (t, *J* = 2.2 Hz, 2H), 3.90 (d, *J* = 10.1 Hz, 1H), 3.84 (d, *J* = 10.1 Hz, 1H), 3.23 (s, 3H).

^{13}C{1H} NMR (101 MHz, $CDCl_3$) 172.4, 144.3, 131.0, 126.6, 125.6, 123.2, 119.3, 109.4, 109.2, 65.2, 26.7, 7.8.

HRMS: m/z for $C_{14}H_{13}IN_2NaO$ $[M+Na]^+$ calcd.: 374.9965, found: 374.9966.

3-(Iodomethyl)-1,3-diphenylindolin-2-one (1mb)

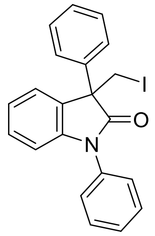

The title compound was obtained according to general procedure **SM-A** from 1-phenylindolin-2-one (628 mg, 3.00 mmol), and bromobenzene. Purification by column chromatography (SiO_2, 0 to 10% EtOAc in hexane) afforded **1mb** as a white solid (674 mg, 1.58 mmol, 53%).

^{1}H-NMR (400 MHz, $CDCl_3$) δ 7.59 – 7.50 (m, 4H), 7.49 – 7.45 (m, 2H), 7.45 – 7.40 (m, 2H), 7.39 – 7.30 (m, 4H), 7.23 (td, *J* = 7.5, 1.0 Hz, 1H), 6.91 (ddd, *J* = 7.9, 1.1, 0.6 Hz, 1H), 4.21 (d, *J* = 9.6 Hz, 1H), 3.84 (d, *J* = 9.7 Hz, 1H).

^{13}C{1H} NMR (101 MHz, CDCl$_3$) δ 175.9, 144.3, 137.7, 134.5, 130.8, 129.7, 129.1, 129.0, 128.4, 128.3, 127.3, 126.9, 125.2, 123.3, 110.1, 56.7, 11.1.

HRMS: m/z for $C_{21}H_{17}INO$ [M+H]$^+$ calcd.: 426.0349, found: 426.0338.

1-(4-(Dimethylamino)phenyl)-3-(iodomethyl)-3-phenylindolin-2-one (1nb)

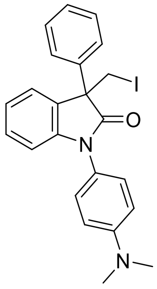

The title compound was obtained according to general procedure **SM-A** from 1-(4-(dimethylamino)phenyl)indolin-2-one (451 mg, 1.79 mmol), and bromobenzene. Purification by column chromatography (SiO_2, 10 to 25% EtOAc in hexane) afforded **1nb** as a white solid (520 mg, 1.11 mmol, 62%).

^{1}H-NMR (400 MHz, CDCl$_3$) δ 7.58 – 7.50 (m, 2H), 7.39 (dd, *J* = 7.5, 1.3 Hz, 1H), 7.37 – 7.24 (m, 6H), 7.18 (td, *J* = 7.5, 1.1 Hz, 1H), 6.84 (dt, *J* = 7.9, 0.8 Hz, 1H), 6.82 – 6.78 (m, 2H), 4.17 (d, *J* = 9.6 Hz, 1H), 3.83 (d, *J* = 9.6 Hz, 1H), 3.00 (s, 6H).

^{13}C{1H} NMR (101 MHz, CDCl$_3$) δ 176.2, 150.5, 145.3, 138.0, 130.8, 129.1, 129.0, 128.2, 127.8, 127.4, 125.0, 123.0, 122.9, 113.0, 110.1, 56.6, 40.7, 11.4.

HRMS: m/z for $C_{23}H_{22}IN_2O$ [M+H]+ calcd.: 469.0771, found: 469.0766.

1-Benzyl-3-(iodomethyl)-3-phenylindolin-2-one (1ob)

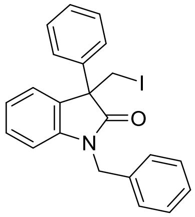

The title compound was obtained according to general procedure **SM-A** from 1-benzylindolin-2-one (670 mg, 3.00 mmol), and bromobenzene. Purification by column chromatography (SiO_2, 0 to 10% EtOAc in hexane) afforded **1ob** as a white-brownish solid (924 mg, 2.10 mmol, 70%).

^{1}H-NMR (400 MHz, CDCl$_3$) δ 7.52 – 7.42 (m, 2H), 7.39 – 7.27 (m, 10H), 7.15 (td, *J* = 7.5, 1.0 Hz, 1H), 6.80 (ddd, *J* = 7.9, 1.0, 0.5 Hz, 1H), 4.98 (d, *J* = 15.7 Hz, 1H), 4.91 (d, *J* = 15.7 Hz, 1H), 4.13 (d, *J* = 9.8 Hz, 1H), 3.82 (d, *J* = 9.8 Hz, 1H).

^{13}C{1H} NMR (101 MHz, CDCl$_3$) δ 176.5, 143.3, 138.1, 135.6, 131.1, 129.1, 129.0, 128.9, 128.3, 127.8, 127.6, 127.3, 125.1, 122.9, 109.9, 56.8, 44.4, 10.3.

The spectroscopic data matched those reported in the literature.[3]

3-(Iodomethyl)-1-(methoxymethyl)-3-phenylindolin-2-one (1pb)

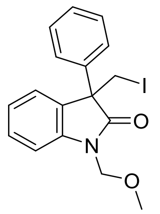

The title compound was obtained according to general procedure **SM-A** from 1-(methoxymethyl)indolin-2-one (440 mg, 2.48 mmol), and bromobenzene. Purification by column chromatography (SiO_2, 0 to 10% EtOAc in hexane) afforded **1pb** as an off-white solid (391 mg, 0.99 mmol, 40%).

^{1}H-NMR (400 MHz, $CDCl_3$) δ 7.46 – 7.40 (m, 3H), 7.38 (ddd, *J* = 7.5, 1.3, 0.6 Hz, 1H), 7.34 – 7.28 (m, 3H), 7.23 (td, *J* = 7.5, 1.0 Hz, 1H), 7.15 (dt, *J* = 7.9, 0.8 Hz, 1H), 5.17 (d, *J* = 11.0 Hz, 1H), 5.12 (d, *J* = 11.0 Hz, 1H), 4.11 (d, *J* = 9.9 Hz, 1H), 3.77 (d, *J* = 9.9 Hz, 1H), 3.36 (s, 3H).

^{13}C{1H} NMR (101 MHz, $CDCl_3$) δ 177.0, 142.5, 137.9, 130.5, 129.4, 129.0, 128.4, 127.2, 125.2, 123.4, 110.3, 72.0, 57.3, 56.9, 9.9.

HRMS: m/z for $C_{17}H_{16}INNaO_2$ $[M+Na]^+$ calcd.: 416.0118, found: 416.0109.

1-(3-(3-(Iodomethyl)-2-oxo-3-phenylindolin-1-yl)propyl)-1H-pyrrole-2-carbonitrile (1qb)

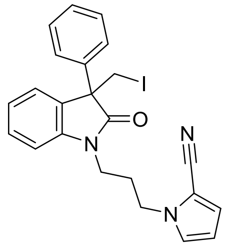

The title compound was obtained according to general procedure **SM-A** from 1-(3-(2-oxoindolin-1-yl)propyl)-1H-pyrrole-2-carbonitrile **SM-8** (327 mg, 1.23 mmol), and bromobenzene. Purification by column chromatography (SiO_2, 0 to 20% EtOAc in hexane) afforded **1qb** as an off-white solid (402 mg, 0.84 mmol, 68%).

^{1}H-NMR (400 MHz, $CDCl_3$) δ 7.45 – 7.38 (m, 3H), 7.37 – 7.28 (m, 4H), 7.22 (td, *J* = 7.5, 1.0 Hz, 1H), 7.03 (dd, *J* = 2.7, 1.6 Hz, 1H), 6.86 (dt, *J* = 7.9, 0.8 Hz, 1H), 6.81 (dd, *J* = 4.0, 1.6 Hz, 1H), 6.16 (dd, *J* = 4.0, 2.6 Hz, 1H), 4.32 – 4.08 (m, 3H), 3.86 (dt, *J* = 14.0, 6.9 Hz, 1H), 3.75 (d, *J* = 9.8 Hz, 1H), 3.64 (dt, *J* = 14.4, 6.3 Hz, 1H), 2.36 – 2.18 (m, 2H).

^{13}C{1H} NMR (101 MHz, $CDCl_3$) δ 176.7, 142.8, 137.6, 131.1, 129.5, 129.1, 128.4, 127.6, 127.2, 125.4, 123.2, 120.7, 114.0, 109.6, 108.8, 103.4, 56.8, 46.6, 37.5, 29.2, 10.4.

HRMS: m/z for $C_{23}H_{21}IN_3O$ $[M+H]^+$ calcd.: 482.0724, found: 482.0715.

1-Methyl-3-phenyl-3-((phenylthio)methyl)indolin-2-one (1aa)

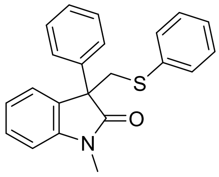

The title compound was obtained according to general procedure **SM-A** from 1-methyloxindole (442 mg, 3.00 mmol), and bromobenzene. Instead of diiodomethane, (chloromethyl)(phenyl)sulfane was used. The procedure remained identical. Purification by column chromatography (SiO_2, 0 to 10% EtOAc in hexane) afforded **1aa** as a white solid (676 mg, 1.96 mmol, 65%).

^{1}H-NMR (400 MHz, $CDCl_3$) δ 7.45 – 7.40 (m, 2H), 7.36 (td, *J* = 7.8, 1.2 Hz, 1H), 7.32 – 7.25 (m, 4H), 7.24 – 7.13 (m, 5H), 7.05 (td, *J* = 7.5, 1.0 Hz, 1H), 6.93 (dt, *J* = 7.7, 0.8 Hz, 1H), 3.92 – 3.82 (m, 2H), 3.23 (s, 3H).

^{13}C{1H} NMR (101 MHz, $CDCl_3$) δ 177.2, 144.4, 138.8, 136.2, 131.0, 130.3, 128.9, 128.9, 128.7, 127.9, 127.2, 126.7, 125.8, 122.7, 108.5, 56.9, 43.4, 26.7.

The spectroscopic data matched those reported in the literature.[4]

General Procedure SM-B

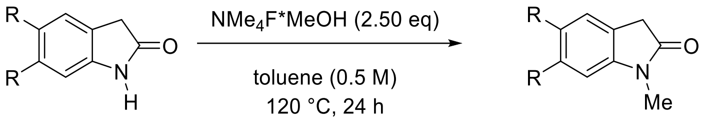

The selective *N*-methylation of oxindoles was adapted from seminal work by Schoenebeck and coworkers.[5] Instead of the reported NMe_4F, the corresponding methanol-adduct as reported by Sanford and coworkers was utilized.[6]

A 20 mL drum vial was charged with oxindole (1.0 eq.) and NMe_4F-methanol adduct (2.5 eq.), then toluene (0.5 M) was added and the vial sealed. The resulting suspension was placed on a heated stirring plate at 130 °C and stirred for 24 hours. Upon cooling to room temperature, the suspension was filtered through a plug of celite and washed with EtOAc. Volatiles were removed *in vacuo*. The obtained crude residue was purified by column chromatography.

5-Methoxy-1-methylindolin-2-one (SM-B-1)

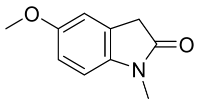

The title compound was obtained according to general procedure **SM-B** from 5-methoxy-1-methylindolin-2-one (1.00 g, 6.13 mmol). Purification by column chromatography (SiO_2, 0 to 30% EtOAc in hexane) afforded **SM-B-1** as an off-white solid (391 mg, 2.21 mmol, 36%).

^{1}H-NMR (400 MHz, $CDCl_3$) δ 6.88 (dt, *J* = 2.4, 1.2 Hz, 1H), 6.81 (ddt, *J* = 8.4, 2.5, 0.8 Hz, 1H), 6.71 (d, *J* = 8.5 Hz, 1H), 3.79 (s, 3H), 3.50 (s, 2H), 3.18 (s, 3H).

^{13}C{1H} NMR (101 MHz, $CDCl_3$) δ 174.8, 156.0, 139.0, 126.0, 112.3, 112.1, 108.4, 56.0, 36.3, 26.4.

The spectroscopic data matched those reported in the literature.[7]

5-Fluoro-1-methylindolin-2-one (SM-B-2)

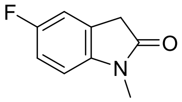

The title compound was obtained according to general procedure **SM-B** from 5-fluoroindolin-2-one (756 mg, 5.00 mmol). Purification by column chromatography (SiO_2, 10 to 20% EtOAc in hexane) afforded **SM-B-2** as an off-white solid (370 mg, 2.24 mmol, 45%).

^{1}H-NMR (400 MHz, $CDCl_3$) δ 6.99 (m, 2H), 6.75 – 6.70 (m, 1H), 3.52 (d, *J* = 1.0 Hz, 2H), 3.20 (s, 3H).

^{13}C{1H} NMR (101 MHz, $CDCl_3$) δ 174.7, 159.2 (d, *J* = 239.9 Hz), 141.4, 126.2, 114.2 (d, *J* = 23.4 Hz), 112.7 (d, *J* = 25.0 Hz), 108.5 (d, *J* = 8.3 Hz), 36.2 (d, *J* = 2.0 Hz), 26.5.

^{19}F{1H} NMR (376 MHz, $CDCl_3$) δ -121.17.

The spectroscopic data matched those reported in the literature.[8]

1-Methyl-1,3-dihydro-2H-pyrrolo[2,3-b]pyridin-2-one (SM-B-3)

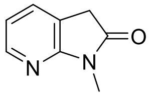

The title compound was obtained according to general procedure **SM-B** from 1,3-dihydro-2*H*-pyrrolo[2,3-b]pyridin-2-one (671 mg, 5.00 mmol). Purification by column chromatography (SiO_2, 50 to 70% EtOAc in hexane) afforded **SM-B-3** as a brownish solid (173 mg, 1.17 mmol, 23%).

^{1}H-NMR (400 MHz, $CDCl_3$) δ 8.19 (ddt, *J* = 5.3, 1.7, 0.9 Hz, 1H), 7.47 (dq, *J* = 7.2, 1.3 Hz, 1H), 6.94 (dd, *J* = 7.2, 5.3 Hz, 1H), 3.54 (s, 2H), 3.30 (s, 3H).

^{13}C{1H} NMR (101 MHz, $CDCl_3$) 174.5, 158.5, 147.0, 131.7, 119.4, 118.1, 35.0, 25.5.

The spectroscopic data matched those reported in the literature.[9]

7-Fluoro-1-methylindolin-2-one (SM-B-4)

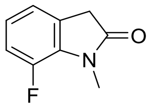

The title compound was obtained according to general procedure **SM-B** from 7-fluoroindolin-2-one (907 mg, 6.00 mmol). Purification by column chromatography (SiO_2, 0 to 20% EtOAc in hexane) afforded **SM-B-4** as an orange solid (601 mg, 3.64 mmol, 61%).

^{1}H-NMR (400 MHz, $CDCl_3$) δ 7.04 – 6.88 (m, 3H), 3.54 (s, 2H), 3.42 (d, *J* = 2.7 Hz, 3H).

^{13}C{1H} NMR (101 MHz, $CDCl_3$) δ 174.6, 147.7 (d, *J* = 243.0 Hz), 131.9 (d, *J* = 8.1 Hz), 127.3 (d, *J* = 3.6 Hz), 122.9 (d, *J* = 6.5 Hz), 120.3 (d, *J* = 3.3 Hz), 116.0 (d, *J* = 19.2 Hz), 36.1 (d, *J* = 2.2 Hz), 28.8 (d, *J* = 5.7 Hz).

^{19}F{1H} NMR (376 MHz, $CDCl_3$) δ -137.3

The spectroscopic data matched those reported in the literature.[10]

1-Methyl-5-(2-phenylthiazol-4-yl)indolin-2-one (SM-B-5)

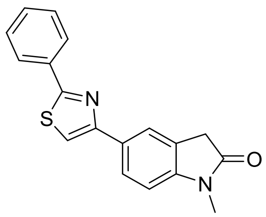

The title compound was obtained according to general procedure **SM-B** from 5-(2-phenylthiazol-4-yl)indolin-2-one hydrochloride (1.63 g, 4.96 mmol) with the addition of K_2CO_3 (685 mg, 4.96 mmol, 1.00 eq.) to deprotonate the hydrochloride salt. Purification by column chromatography (SiO_2, 30 to 50% EtOAc in hexane) afforded **SM-B-5** as an orange solid (1.07 g, 3.49 mmol, 70%).

^{1}H-NMR (400 MHz, $CDCl_3$) δ 8.07 – 7.99 (m, 2H), 7.97 – 7.90 (m, 2H), 7.55 – 7.42 (m, 3H), 7.38 (s, 1H), 6.95 – 6.83 (m, 1H), 3.60 (d, *J* = 1.1 Hz, 2H), 3.26 (s, 3H).

$^{13}C\{1H\}$ NMR (101 MHz, $CDCl_3$) δ 175.3, 168.1, 156.2, 145.4, 133.9, 130.2, 129.5, 129.1, 126.7, 126.4, 125.1, 122.9, 111.4, 108.3, 36.0, 26.5.

Substrates according to known procedures

5-(2-Phenylthiazol-4-yl)indolin-2-one (SM-1)

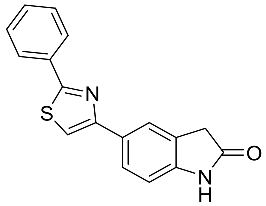

The title compound was synthesized according to the literature.[11]

1-Methyl-3-(1H-pyrrol-1-yl)indolin-2-one (SM-2)

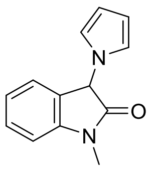

The title compound was synthesized according to the literature.[12]

3-Benzylindolin-2-one (SM-3)

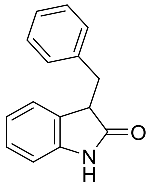

The title compound was synthesized according to the literature.[13]

1-Benzylindolin-2-one (SM-4)

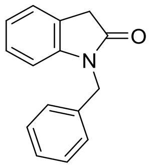

The title compound was synthesized according to the literature.[14]

1-(Methoxymethyl)indolin-2-one (SM-5)

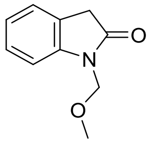

The title compound was synthesized according to the literature.[15]

Other substrates

1,5-Dimethylindolin-2-one (SM-6)

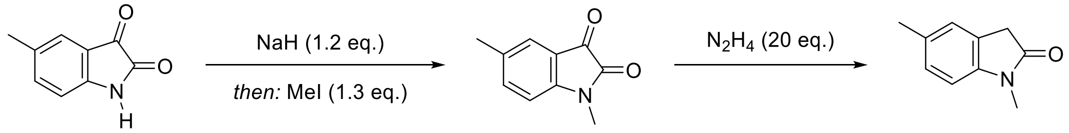

The title compound was synthesized according to the following modified procedure.[14]

To a solution of 5-methyl-2,3-dihydro-1H-indole-2,3-dione (1.61 g, 10.0 mmol, 1.00 eq.) in THF (33 mL) at 0 °C was added sodium hydride (60% in mineral oil; 480 mg, 12.0 mmol, 1.20 eq.) in portions. The resulting suspension was stirred for 30 min. Then, iodomethane (813 µL, 13.0 mmol, 1.30 eq.) was added and the reaction mixture stirred at room temperature for 12 h. Subsequently, NH_4Cl (aq.) and water were added and the organic phase extracted with EtOAc (3 × 30 mL). The combined organic phases were dried over $MgSO_4$ and volatiles were removed *in vacuo*. The crude mixture was used directly in the subsequent step.

To the crude mixture was added hydrazine hydrate (9.74 mL, 200 mmol, 20.0 eq.) and the resulting suspension was heated to 100 °C for 4 h. Upon completion, the mixture was cooled to room temperature, water was added and the aqueous phase was extracted with EtOAc (3 × 30 mL). The combined organic phases were dried over $MgSO_4$ and volatiles were removed *in vacuo*. The title compound was obtained upon purification by column chromatography (SiO_2, 0 to 15% EtOAc in hexane) as an off-white solid (1.24 g, 7.69 mmol, 77%).

^{1}H-NMR (400 MHz, $CDCl_3$) δ 7.14 – 7.00 (m, 2H), 6.78 – 6.46 (m, 1H), 3.48 (s, 2H), 3.19 (s, 3H), 2.33 (s, 3H).

^{13}C{1H} NMR (101 MHz, $CDCl_3$) δ 175.2, 143.0, 132.0, 128.2, 125.4, 124.7, 107.9, 35.9, 26.3, 21.2.

The spectroscopic data matched those reported in the literature.[16]

3-(Iodomethyl)-1-methyl-3-phenyl-7-(trifluoromethyl)indolin-2-one (1fb)

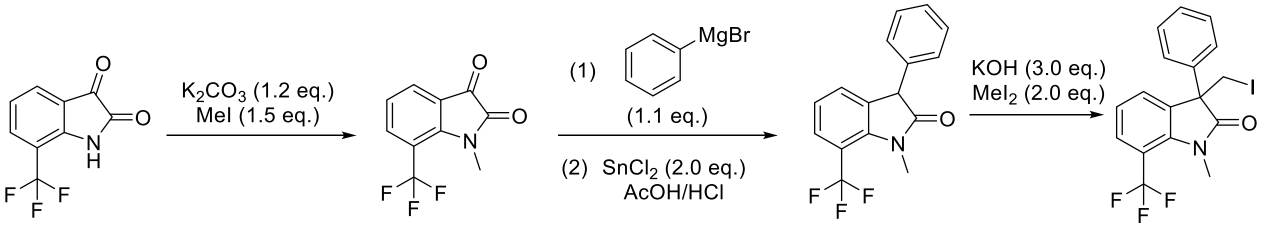

1-methyl-7-(trifluoromethyl)indoline-2,3-dione was synthesized according to the literature.[17]

The title compound was synthesized according to the following modified procedure.[18]

To a solution of 1-methyl-7-(trifluoromethyl)indoline-2,3-dione (980 mg, 4.30 mmol, 1.00 eq.) in anhydrous THF (22 mL) at 0 °C was added PhMgBr (1.60 mL of 3 M solution, 4.56 mmol, 1.10 eq.) dropwise. The mixture was allowed to warm to room temperature. After one hour, the reaction mixture was quenched with sat. NH_4Cl (30 mL) and extracted with ether (3 × 30 mL). The combined extracts were washed with brine and dried over sodium sulfate, and then concentrated *in vacuo*.

The obtained crude intermediate was redissolved in AcOH/HCl (25 mL/1.7 mL), $SnCl_2$ (1.60 g, 8.46 mmol, 2.00 eq.) was added at room temperature. The mixture was heated to reflux for 4 h. The mixture was diluted with H_2O (20 mL) and extracted with ethyl acetate (100 mL). The organic phase was washed with sat. Na_2CO_3 solution and brine, dried over sodium sulfate, and concentrated *in vacuo* and purified by column chromatography (SiO_2, 0 to 10% EtOAc in hexane) to sufficient purity to proceed.

The resulting mixture was redissolved in DMF (10 mL) and diiodomethane (690 µL, 8.60 mmol, 2.00 eq.) was added. The reaction was degassed through continuous bubbling with nitrogen. Then, KOH (724 mg, 12.9 mmol, 3.00 eq.) was added. The resulting mixture was stirred for 4 h at room temperature. Equivalent amounts of water and EtOAc (total 20 mL) were added, and the organic phase collected. The aqueous phase was washed with EtOAc (3 x 20 mL). The combined organic residues were dried over $MgSO_4$ and volatiles removed *in vacuo.* The obtained crude residue was purified by column chromatography (SiO_2, 0 to 5% EtOAc in hexane) giving the title product as a yellow solid (206 mg, 0.48 mmol, 11% over 4 steps).

^{1}H-NMR (400 MHz, $CDCl_3$) δ 7.76 – 7.66 (m, 1H), 7.53 – 7.45 (m, 1H), 7.38 – 7.27 (m, 5H), 7.25 – 7.22 (m, 1H), 4.09 (d, *J* = 9.8 Hz, 1H), 3.74 (d, *J* = 9.8 Hz, 1H), 3.45 (q, *J* = 2.4 Hz, 3H).

^{13}C{1H} NMR (101 MHz, $CDCl_3$) δ 177.3, 142.2, 137.2, 133.7, 129.1, 128.6, 128.4, 127.3 (q, *J* = 6.1 Hz), 127.1, 123.6 (q, *J* = 271.6 Hz) 122.2, 113.2 (dd, *J* = 33.0 Hz), 55.3, 29.4 (q, *J* = 6.5 Hz), 9.7.

^{19}F{1H} NMR (376 MHz, $CDCl_3$) -53.1.

HRMS: m/z [M+H]$^+$ calcd. for: $C_{17}H_{14}F_3INO$ 432.0067, found: 432.0067.

3-(Iodomethyl)-3-methylindolin-2-one (1rb)

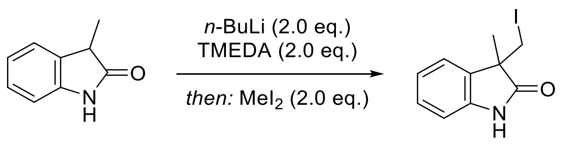

To *n*-BuLi (6.25 mL of 1.6 M solution in hexanes, 10.0 mmol, 2.00 eq.) in THF (10 mL) at -78 °C were added slowly TMEDA (1.50 mL, 10.0 mmol, 2.00 eq.) and 3-methylindolin-2-one (736 mg, 5.00 mmol, 1.00 eq.). The resulting mixture was allowed to warm to -20 °C and was stirred for 1 h, then slowly to room temperature over 1 h. Then diiodomethane (807 µL, 10.0 mmol, 2.00 eq.) was added at once, and the reaction stirred for 2 h. The mixture was quenched through the addition of sat. NH_4Cl (20 mL) and the aqueous phase extracted with EtOAc (3 × 20 mL) The combined organic residues were dried over $MgSO_4$ and volatiles removed *in vacuo.* The obtained crude residue was purified by column chromatography (SiO_2, 15 to 30% EtOAc in hexane), giving the title product as a white solid (743 mg, 2.59 mmol, 52%).

^{1}H-NMR (400 MHz, $CDCl_3$) δ 9.03 (s, 1H), 7.36 – 7.23 (m, 2H), 7.16 – 7.07 (m, 1H), 7.05 – 6.89 (m, 1H), 3.54 (d, *J* = 9.9 Hz, 1H), 3.45 (d, *J* = 9.9 Hz, 1H), 1.56 (s, 3H).

^{13}C{1H} NMR (101 MHz, $CDCl_3$) δ 180.6, 140.5, 133.2, 128.8, 123.1, 122.8, 110.4, 49.3, 23.4, 10.3.

HRMS: m/z [M+H]$^+$ calcd. for: $C_{10}H_{11}INO$ 287.988, found: 287.988.

1-(4-(Dimethylamino)phenyl)indolin-2-one (SM-7)

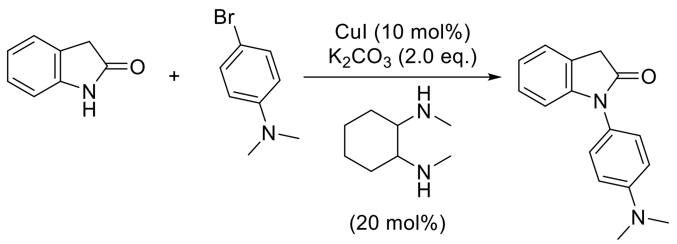

The title compound was synthesized according to the following modified procedure.[1]

In a glovebox, *N,N*-dimethyl-*p*-bromoaniline (1.20 g, 6.00 mmol, 1.00 eq.), indolin-2-one (959 mg, 7.20 mmol, 1.20 eq.), CuI (114 mg, 0.60 mmol, 0.10 eq.), K_2CO_3 (1.66 g, 12.0 mmol, 2.00 eq.), rac- *N,N*-dimethylcyclohexane-1,2-diamine (378 µL, 1.20 mmol, 0.20 eq.) and dioxane (3 mL) were combined. The reaction flask was sealed with a rubber septum and removed from the glovebox. Outside, *N,N*-dimethyl-4-bromoaniline was added and the flask heated to 80 °C for 24 h. Subsequently, the reaction mixture was cooled to room temperature diluted with ethyl acetate (15 mL), and filtered through a plug of silica, eluting with additional ethyl acetate (50 mL). The filtrate was concentrated, and the resulting residue was purified by column chromatography (SiO_2, 10 to 30% EtOAc in hexane). The title compound was obtained as an orange solid (451 mg, 1.79 mmol, 30%).

^{1}H-NMR (400 MHz, $CDCl_3$) δ 7.30 – 7.27 (m, 1H), 7.24 – 7.21 (m, 2H), 7.21 – 7.15 (m, 1H), 7.04 (td, *J* = 7.5, 1.1 Hz, 1H), 6.87 – 6.79 (m, 2H), 6.73 (ddd, *J* = 7.9, 1.1, 0.6 Hz, 1H), 3.68 (s, 1H), 3.01 (s, 6H).

^{13}C{1H} NMR (101 MHz, $CDCl_3$) δ 175.1, 150.4, 146.4, 127.9, 127.7, 124.5, 124.5, 122.5, 113.2, 109.5, 40.7, 36.2.

The spectroscopic data matched those reported in the literature.[1]

1-(3-(2-Oxoindolin-1-yl)propyl)-1H-pyrrole-2-carbonitrile (SM-8)

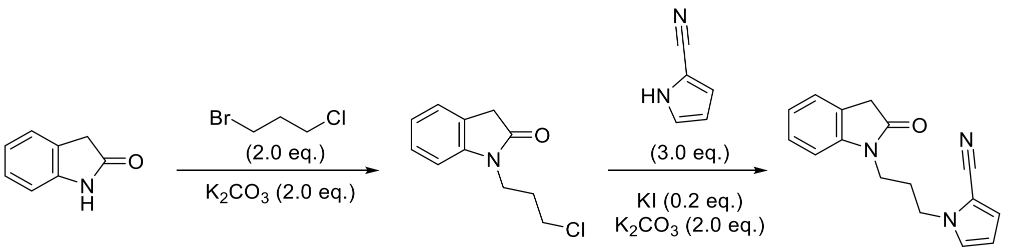

1-(3-chloropropyl)indolin-2-one was synthesized according to the literature.[19]

To 1-(3-chloropropyl)indolin-2-one (400 mg, 1.91 mmol, 1.00 eq.) in MeCN (4.8 mL) was added K_2CO_3 (527 mg, 3.82 mmol, 2.00 eq.), KI (63.3 mg, 382 µmol, 0.20 eq.), and 2-cyano-1*H*-pyrrole (486 µL, 5.72 mmol, 3.00 eq.). The resulting reaction mixture was heated to 80 °C for 16 hours. Upon cooling to room temperature water (10 mL) was added and the aqueous phase extracted with EtOAc (3 × 10 mL). The combined organic residues were dried over $MgSO_4$ and volatiles removed *in vacuo.* The obtained crude residue was purified by column chromatography (SiO_2, 50% EtOAc in hexane), giving the title product as an off-white solid (342 mg, 1.29 mmol, 68%).

^{1}H-NMR (400 MHz, $CDCl_3$) δ 7.33 – 7.22 (m, 2H), 7.11 – 6.96 (m, 2H), 6.81 (m, 1H), 6.73 (d, *J* = 7.7, 1H), 6.27 – 6.11 (m, 1H), 4.13 (td, *J* = 6.9, 2.2 Hz, 2H), 3.75 (td, *J* = 6.9, 2.1 Hz, 2H), 3.55 (d, *J* = 2.3 Hz, 2H), 2.53 – 2.08 (m, 2H).

$^{13}C\{^1H\}$ NMR (101 MHz, $CDCl_3$) δ 175.4, 144.0, 128.2, 127.2, 124.8, 124.7, 122.7, 120.6, 113.9, 109.7, 108.2, 103.5, 46.5, 37.1, 35.9, 29.1.

1,3-Dimethyl-3-((methylthio)methyl)indolin-2-one (1se)

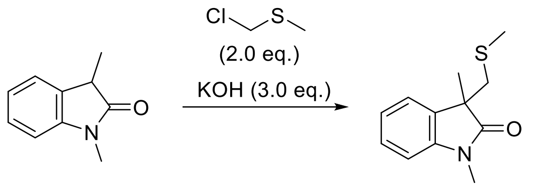

1,3-Dimethylindolin-2-one (806 mg, 5.00 mmol, 1.00 eq.) was dissolved in DMF (10 mL) and (chloromethyl)(methyl)sulfane (832 µL, 10.0 mmol, 2.00 eq.) was added. The reaction was degassed through continuous bubbling with nitrogen. Then, KOH (842 mg, 15.0 mmol, 3.00 eq.) was added. The resulting mixture was stirred for 4 h at room temperature. Equivalent amounts of water and EtOAc (total 20 mL) were added, and the organic phase collected. The aqueous phase was washed with EtOAc (3 x 20 mL). The combined organic residues were dried over $MgSO_4$ and volatiles removed *in vacuo.* The obtained crude residue was purified by column chromatography (SiO_2, 0 to 10% EtOAc in hexane), giving the title product as a yellow oil that solidified over time (769 mg, 3.47 mmol, 70%).

^1H-NMR (400 MHz, $CDCl_3$) δ 7.34 – 7.27 (m, 2H), 7.07 (td, *J* = 7.5, 1.0 Hz, 1H), 6.87 (d, *J* = 7.6, 1H), 3.24 (s, 3H), 3.00 (d, *J* = 13.2 Hz, 1H), 2.94 (d, *J* = 13.2 Hz, 1H), 1.93 (s, 3H), 1.43 (s, 3H).

$^{13}C\{^1H\}$ NMR (101 MHz, $CDCl_3$) δ 179.6, 143.7, 133.1, 128.4, 123.2, 122.6, 108.2, 49.5, 42.7, 26.4, 23.1, 17.6.

The spectroscopic data matched those reported in the literature.[4]

Compound **1sa** was synthesized analogously with (chloromethyl)(phenyl)sulfane instead.

3-Benzyl-1-methyl-3-((methylthio)methyl)indolin-2-one (1te)

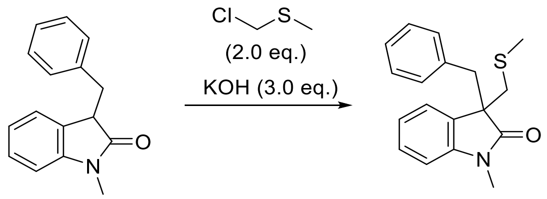

3-benzyl-1-methylindolin-2-one (1.19 g, 5.00 mmol, 1.00 eq.) was dissolved in DMF (10 mL) and (chloromethyl)(methyl)sulfane (832 µL, 10.0 mmol, 2.00 eq.) was added. The reaction was degassed through continuous bubbling with nitrogen. Then, KOH (842 mg, 15.0 mmol, 3.00 eq.) was added. The resulting mixture was stirred for 4 h at room temperature. Equivalent amounts of water and EtOAc (total 20 mL) were added, and the organic phase collected. The aqueous phase was washed with EtOAc (3 x 20 mL). The combined organic residues were dried over $MgSO_4$ and volatiles removed *in vacuo.* The obtained crude residue was purified by column chromatography (SiO_2, 0 to 10% EtOAc in hexane), giving the title product as a yellow oil that solidified over time (699 mg, 2.35 mmol, 47%).

^1H-NMR (400 MHz, $CDCl_3$) δ 7.25 – 7.16 (m, 2H), 7.11 – 6.96 (m, 4H), 6.90 – 6.80 (m, 2H), 6.62 (d, *J* = 7.7, 1H), 3.16 – 3.08 (m, 4H), 3.00 (s, 3H), 1.96 (s, 3H).

^{13}C{1H} NMR (101 MHz, CDCl$_3$) δ 178.1, 144.1, 135.4, 130.1, 129.9, 128.3, 127.6, 126.6, 124.0, 122.0, 107.9, 55.5, 43.3, 41.1, 25.9, 17.5.

HRMS: m/z $[M+H]^+$ calcd. for: $C_{18}H_{20}NOS$ 298.126, found: 298.1255.

3-isopropyl-1-methyl-3-((methylthio)methyl)indolin-2-one (1ue)

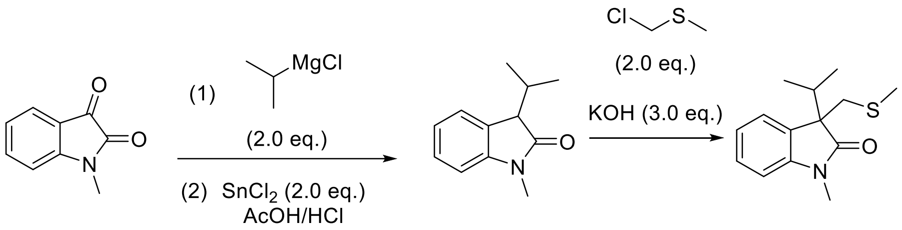

To a solution of 1-methylindoline-2,3-dione (650 mg, 4.03 mmol, 1.00 eq.) in anhydrous THF (4 mL) at 0 °C was added isopropylmagnesiumchloride (4.03 mL of 2 M solution, 8.07 mmol, 2.00 eq.) dropwise. The mixture was allowed to warm to room temperature. After one hour, the reaction mixture was quenched with sat. NH_4Cl (30 mL) and extracted with ether (3 × 30 mL). The combined extracts were washed with brine and dried over sodium sulfate, and then concentrated *in vacuo*.

The obtained crude intermediate was redissolved in AcOH/HCl (25 mL/1.7 mL), $SnCl_2$ (1.53 g, 8.07 mmol, 2.00 eq.) was added at room temperature. The mixture was heated to reflux for 4 h. The mixture was diluted with H_2O (20 mL) and extracted with ethyl acetate (100 mL). The organic phase was washed with sat. Na_2CO_3 solution and brine, dried over sodium sulfate, and concentrated *in vacuo*.

The resulting mixture was redissolved in DMF (3.3 mL) and (chloromethyl)(methyl)sulfane (277 µL, 3.32 mmol, 2.00 eq.) was added. The reaction was degassed through continuous bubbling with nitrogen. Then, KOH (279 mg, 4.98 mmol, 3.00 eq.) was added. The resulting mixture was stirred for 4 h at room temperature. Equivalent amounts of water and EtOAc (total 20 mL) were added, and the organic phase collected. The aqueous phase was washed with EtOAc (3 x 20 mL). The combined organic residues were dried over $MgSO_4$ and volatiles removed *in vacuo.* The obtained crude residue was purified by column chromatography (SiO_2, 0 to 10% EtOAc in hexane) giving the title product as a yellow oil (371 mg, 1.49 mmol, 37% over 3 steps).

^{1}H-NMR (400 MHz, CDCl$_3$) δ 7.31 (td, J = 7.7, 1.2 Hz, 1H), 7.23 (ddd, J = 7.4, 1.2, 0.6 Hz, 1H), 7.06 (td, J = 7.5, 1.0 Hz, 1H), 6.85 (dt, J = 7.8, 0.8 Hz, 1H), 3.23 (s, 3H), 3.17 (d, J = 12.7 Hz, 1H), 2.98 (d, J = 12.7 Hz, 1H), 2.22 (p, J = 6.8 Hz, 1H), 1.89 (s, 3H), 0.96 (d, J = 6.9 Hz, 3H), 0.76 (d, J = 6.8 Hz, 3H).

^{13}C{1H} NMR (101 MHz, CDCl$_3$) δ 179.0, 144.8, 130.4, 128.3, 124.0, 122.2, 107.9, 57.3, 40.1, 35.4, 26.1, 17.5, 17.5, 17.5.

HRMS: m/z $[M+Na]^+$ calcd. for: $C_{14}H_{19}NNaOS$ 272.108, found: 272.1075.

3-(Bromomethyl)-1-methyl-3-phenylindolin-2-one (1ac)

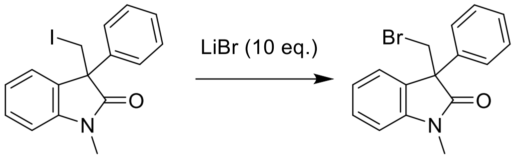

To 3-(iodomethyl)-1-methyl-3-phenylindolin-2-one (218 mg, 600 µmol, 1.00 eq.) in DMSO (4.2 mL), was added LiBr (717 mg, 8.26 mmol, 10.0 eq.). The resulting mixture was heated to 100 °C for 16 hours, then water (20 mL) was added and the aqueous phase extracted with EtOAc (3 x 20 mL). The combined organic residues were dried over $MgSO_4$ and volatiles removed *in vacuo.* The title compound was directly obtained in sufficient purity (175 mg, 553 µmol, 92%).

^{1}H-NMR (400 MHz, $CDCl_3$) δ 7.49 – 7.39 (m, 4H), 7.37 – 7.28 (m, 3H), 7.20 (ddd, *J* = 7.7, 7.4, 1.0 Hz, 1H), 6.94 (dt, *J* = 7.7, 0.9 Hz, 1H), 4.18 (d, *J* = 10.0 Hz, 1H), 3.98 (d, *J* = 10.0 Hz, 1H), 3.24 (s, 3H).

^{13}C{1H} NMR (101 MHz, $CDCl_3$) δ 170.7, 144.4, 137.4, 129.9, 129.3, 128.9, 128.3, 127.3, 125.3, 122.9, 108.7, 57.2, 36.6, 26.7.

The spectroscopic data matched those reported in the literature.[3]

3-(Chloromethyl)-1-methyl-3-phenylindolin-2-one (1ad)

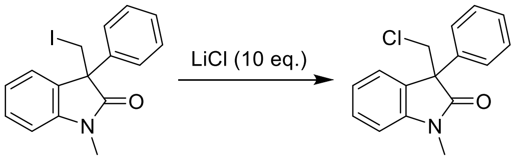

To 3-(iodomethyl)-1-methyl-3-phenylindolin-2-one (159 mg, 438 µmol, 1.00 eq.) in DMSO (4.2 mL), was added LiCl (186 mg, 4.38 mmol, 10.0 eq.). The resulting mixture was heated to 100 °C for 16 hours, then water (20 mL) was added and the aqueous phase extracted with EtOAc (3 x 20 mL). The combined organic residues were dried over $MgSO_4$ and volatiles removed *in vacuo.* The title compound was directly obtained in sufficient purity (116 mg, 427 µmol, 98%).

^{1}H-NMR (400 MHz, $CDCl_3$) δ 7.46 – 7.38 (m, 4H), 7.37 – 7.28 (m, 3H), 7.22 – 7.15 (m, 1H), 6.94 (dt, *J* = 7.7, 0.8 Hz, 1H), 4.29 (d, *J* = 10.8 Hz, 1H), 4.15 (d, *J* = 10.8 Hz, 1H), 3.24 (s, 3H).

^{13}C{1H} NMR (101 MHz, $CDCl_3$) δ 175.9, 144.5, 136.9, 129.4, 129.2, 128.9, 128.3, 127.3, 125.5, 122.9, 108.7, 57.8, 48.5, 26.7.

HRMS: m/z $[M+H]^+$ calcd. for: $C_{16}H_{15}ClNO$ 272.0837, found: 272.0835.

.

Substrate scope

General procedure A

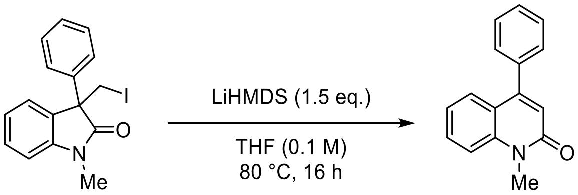

A flame-dried 4 mL drum vial in a glovebox was charged with oxindole (1.0 eq.) and LiHMDS (1.5 eq.) and a magnetic stir bar. The vial was sealed with a septum-cap and removed from the glovebox. Then, THF (0.1 M) was added through the septum. The resulting mixture was placed on a heated stirring plate at 80 °C for 16 h. Upon completion, volatiles were removed *in vacuo*. Purification by column chromatography or preparative TLC afforded the product.

Alternatively, the oxindole can be added to a flame-dried vial with a septum. Upon changing the atmosphere to nitrogen, THF (0.1 M), and 1 M LiHMDS in THF (1.5 eq.) solution were added. This procedure bypasses the necessity to utilize a glovebox (see compound 2a for yield comparison).

1-Methyl-4-phenylquinolin-2(1*H*)-one (2a)

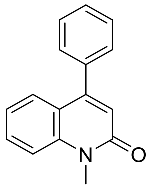

The title compound was obtained according to general procedure **A** from **1ab** (90.8 mg, 0.25 mmol). Purification by column chromatography (SiO_2, 25% EtOAc in pentane) afforded **2a** as a white-yellow solid (45.0 mg, 0.19 mmol, 77%).

When commercially available 1 M LiHMDS solution in THF was used instead of the LiHMDS from the glovebox, the title compound was afforded in comparable yield (41.5 mg, 0.18 mmol, 71%)

^{1}H-NMR (400 MHz, $CDCl_3$) δ 7.61 – 7.54 (m, 2H), 7.52 – 7.46 (m, 3H), 7.46 – 7.40 (m, 3H), 7.17 (ddt, *J* = 8.1, 7.1, 1.0 Hz, 1H), 6.68 (s, 1H), 3.78 (s, 3H).

^{13}C{1H} NMR (101 MHz, $CDCl_3$) δ 162.1, 151.0, 140.5, 137.2, 130.8, 129.1, 128.8, 128.7, 127.9, 122.0, 121.4, 120.6, 114.6, 29.6.

The spectroscopic data matched those reported in the literature.[20]

1,6-Dimethyl-4-phenylquinolin-2(1*H*)-one (2b)

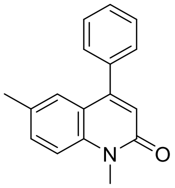

The title compound was obtained according to general procedure **A** from **1bb** (94.3 mg, 0.25 mmol). Purification by preparative TLC (SiO_2, 45% EtOAc in pentane) afforded **2b** as a yellow solid (45.0 mg, 0.18 mmol, 72%).

^{1}H-NMR (400 MHz, $CDCl_3$) δ 7.49 (m, 3H), 7.41 (m, 3H), 7.36 – 7.30 (m, 2H), 6.66 (s, 1H), 3.77 (s, 3H), 2.33 (s, 3H).

^{13}C{1H} NMR (101 MHz, $CDCl_3$) 162.0, 150.8, 138.5, 137.4, 132.0, 131.6, 129.0, 128.7, 128.7, 127.5, 121.5, 120.5, 114.5, 29.6, 20.9.

The spectroscopic data matched those reported in the literature.[21]

6-Methoxy-1-methyl-4-phenylquinolin-2(1*H*)-one (2c)

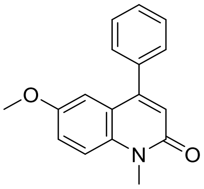

The title compound was obtained according to general procedure **A** from **1cb** (98.3 mg, 0.25 mmol). Purification by preparative TLC (SiO_2, 70% EtOAc in pentane) afforded **2c** as a white-yellow solid (56.0 mg, 0.21 mmol, 84%).

^{1}H-NMR (400 MHz, $CDCl_3$) δ 7.56 – 7.41 (m, 5H), 7.38 (d, *J* = 9.2 Hz, 1H), 7.20 (dd, *J* = 9.2, 2.9 Hz, 1H), 7.03 (d, *J* = 2.9 Hz, 1H), 6.69 (s, 1H), 3.77 (s, 3H), 3.73 (s, 3H).

^{13}C{1H} NMR (101 MHz, $CDCl_3$) 161.7, 154.7, 150.4, 137.3, 135.1, 129.0, 128.9, 128.8, 122.1, 121.4, 118.8, 115.8, 110.5, 55.8, 29.7.

HRMS: m/z for $C_{17}H_{16}NO_2$ $[M+H]^+$ calcd.: 266.1176, found: 266.1175.

6-Fluoro-1-methyl-4-phenylquinolin-2(1*H*)-one (2d)

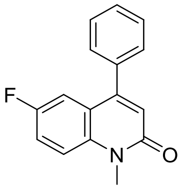

The title compound was obtained according to general procedure **A** from **1db** (95.3 mg, 0.25 mmol). Purification by preparative TLC (SiO_2, 40% EtOAc in pentane) afforded **2d** as a white-yellow solid (31.1 mg, 0.12 mmol, 49%).

^{1}H-NMR (400 MHz, $CDCl_3$) δ 7.53 – 7.46 (m, 3H), 7.44 – 7.36 (m, 3H), 7.31 (ddd, *J* = 9.2, 7.6, 2.9 Hz, 1H), 7.24 (dd, *J* = 9.5, 2.9 Hz, 1H), 6.72 (d, *J* = 0.7 Hz, 1H), 3.77 (s, 3H).

^{13}C{1H} NMR (101 MHz, $CDCl_3$) 161.6, 157.8 (d, *J* = 241.7 Hz), 150.1 (d, *J* = 3.1 Hz), 136.9, 136.5, 129.0, 128.9, 128.9, 122.5, 121.6 (d, *J* = 8.0 Hz), 118.4 (d, *J* = 23.8 Hz), 116.0 (d, *J* = 8.1 Hz), 113.0 (d, *J* = 23.8 Hz), 29.8.

^{19}F{1H} NMR (376 MHz, $CDCl_3$) δ -120.68.

HRMS: m/z for $C_{16}H_{13}FNO$ $[M+H]^+$ calcd.: 254.0976, found: 254.0973.

8-Fluoro-1-methyl-4-phenylquinolin-2(1*H*)-one (2e)

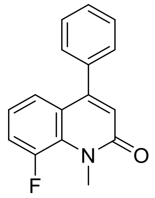

The title compound was obtained according to general procedure **A** from **1eb** (95.3 mg, 0.25 mmol). Purification by preparative TLC (SiO_2, 20% EtOAc in pentane) afforded **2f** as an off-white-solid (25.5 mg, 0.10 mmol, 40%).

^{1}H-NMR (400 MHz, $CDCl_3$) δ 7.51 – 7.46 (m, 3H), 7.41 – 7.34 (m, 2H), 7.35 – 7.27 (m, 2H), 7.10 – 7.02 (m, 1H), 6.69 (s, 1H), 3.97 (d, *J* = 9.0 Hz, 3H).

^{13}C{1H} NMR (101 MHz, $CDCl_3$) δ 162.3, 150.8 (d, *J* = 246.5 Hz), 150.8 (d, *J* = 2.4 Hz), 137.1, 129.8 (d, *J* = 6.5 Hz), 128.9, 128.9, 128.7, 123.8 (d, *J* = 3.5 Hz), 123.6 (d, *J* = 2.4 Hz), 122.3, 122.2, 118.0 (d, *J* = 23.8 Hz), 33.7 (d, *J* = 16.3 Hz).

^{19}F{1H} NMR (376 MHz, $CDCl_3$) δ -120.6

HRMS: m/z for $C_{16}H_{13}FNO$ $[M+H]^+$ calcd.: 254.0976, found: 254.0978.

1-Methyl-4-phenyl-8-(trifluoromethyl)quinolin-2(1*H*)-one (2f)

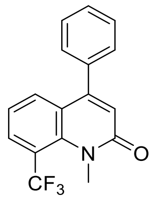

The title compound was obtained according to general procedure **A** with the addition of 20 mol% of morpholine (4.31 µL, 0.05 mmol) from **1fb** (100 mg, 0.24 mmol). Purification by preparative TLC (SiO_2, 10% EtOAc in pentane) afforded **2g** as an yellow solid (38.0 mg, 0.13 mmol, 55%).

^{1}H-NMR (400 MHz, $CDCl_3$) δ 7.92 (dd, *J* = 7.8, 1.6 Hz, 1H), 7.68 (dd, *J* = 7.9, 1.6 Hz, 1H), 7.53 – 7.45 (m, 3H), 7.40 – 7.34 (m, 2H), 7.21 (td, *J* = 7.8, 0.8 Hz, 1H), 6.70 (s, 1H), 3.78 (q, *J* = 2.3 Hz, 3H).

^{13}C{1H} NMR (101 MHz, $CDCl_3$) δ 163.7, 151.1, 140.6, 136.8, 131.8, 131.1 (q, *J* = 6.3 Hz), 129.1, 129.0, 128.9, 125.4 (q, *J* = 271 Hz), 123.4, 121.9, 121.5, 117.9 (q, *J* = 31.8 Hz), 38.0 (q, *J* = 7.7 Hz).

^{19}F{1H} NMR (376 MHz, $CDCl_3$) δ -52.1.

HRMS: m/z for $C_{17}H_{13}F_3NO$ $[M+H]^+$ calcd.: 304.0944, found: 304.0945.

1-Methyl-4-phenyl-1,8-naphthyridin-2(1*H*)-one (2g)

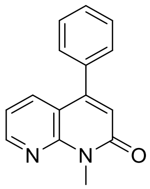

The title compound was obtained according to general procedure **A** from **1eb** (91.0 mg, 0.25 mmol). Purification by preparative TLC (SiO_2, 55% EtOAc in pentane) afforded **2e** as a white-solid (24.7 mg, 0.11 mmol, 42%).

^{1}H-NMR (400 MHz, $CDCl_3$) δ 8.62 (dd, *J* = 4.6, 1.8 Hz, 1H), 7.88 (dd, *J* = 7.9, 1.8 Hz, 1H), 7.55 – 7.46 (m, 3H), 7.45 – 7.33 (m, 2H), 7.13 (dd, *J* = 7.9, 4.6 Hz, 1H), 6.74 (s, 1H), 3.90 (s, 3H).

^{13}C{1H} NMR (101 MHz, $CDCl_3$) 162.8, 150.3, 149.8, 149.3, 136.0, 135.5, 129.1, 128.8, 122.1, 117.8, 115.7, 28.4.

The spectroscopic data matched those reported in the literature.[22]

1-Methyl-4-phenyl-6-(2-phenylthiazol-4-yl)quinolin-2(1*H*)-one (2h)

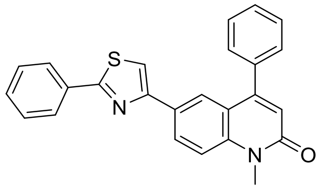

The title compound was obtained according to general procedure **A** with the addition of 20 mol% of morpholine (4.31 µL, 0.05 mmol) from **1hb** (131 mg, 0.25 mmol). Purification by preparative TLC (SiO_2, 65% EtOAc in pentane) afforded **2h** as an brownish solid (63.8 mg, 0.16 mmol, 65%).

^{1}H-NMR (400 MHz, $CDCl_3$) δ 8.27 (dd, *J* = 8.8, 2.1 Hz, 1H), 8.16 (d, *J* = 2.0 Hz, 1H), 8.05 – 7.94 (m, 2H), 7.58 – 7.48 (m, 6H), 7.48 – 7.41 (m, 3H), 7.33 (s, 1H), 6.73 (s, 1H), 3.83 (s, 3H).

^{13}C{1H} NMR (101 MHz, $CDCl_3$) δ 168.3, 162.0, 155.2, 151.1, 140.3, 137.2, 133.7, 130.3, 129.3, 129.2, 129.1, 129.0, 128.8, 128.8, 126.7, 125.4, 121.8, 120.8, 115.0, 112.5, 29.8.

HRMS: m/z for $C_{25}H_{19}N_2OS$ $[M+H]^+$ calcd.: 395.1213, found: 395.1207.

1-Methyl-4-(4-(methylthio)phenyl)quinolin-2(1*H*)-one (2i)

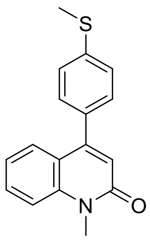

The title compound was obtained according to general procedure **A** from **1ib** (102 mg, 0.25 mmol). Purification by preparative TLC (SiO_2, 65% EtOAc in pentane) afforded **2i** as a white-yellow solid (42.4 mg, 0.15 mmol, 60%).

^{1}H-NMR (400 MHz, $CDCl_3$) δ 7.59 (m, 2H), 7.43 (dd, *J* = 9.0, 1.1 Hz, 1H), 7.40 – 7.28 (m, 4H), 7.23 – 7.11 (m, 1H), 6.66 (s, 1H), 3.77 (s, 3H), 2.55 (s, 3H).

^{13}C{1H} NMR (101 MHz, $CDCl_3$) 162.0, 150.5, 140.5, 139.8, 133.7, 130.8, 129.5, 127.7, 126.3, 122.1, 121.3, 120.5, 114.6, 29.6, 15.6.

HRMS: m/z for $C_{17}H_{16}NOS$ $[M+H]^+$ calcd.: 282.0947, found: 282.0946.

4-(4-Chlorophenyl)-1-methylquinolin-2(1*H*)-one (2j)

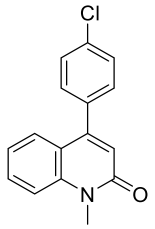

The title compound was obtained according to general procedure **A** from **1jb** (99.4 mg, 0.25 mmol). Purification by preparative TLC (SiO_2, 50% EtOAc in pentane) afforded **2j** as a white-brown solid (30.1 mg, 0.11 mmol, 44%).

^{1}H-NMR (400 MHz, $CDCl_3$) δ 7.60 (ddd, *J* = 8.6, 7.2, 1.5 Hz, 1H), 7.54 – 7.46 (m, 3H), 7.46 – 7.42 (m, 1H), 7.39 – 7.34 (m, 2H), 7.18 (ddd, *J* = 8.2, 7.1, 1.1 Hz, 1H), 6.66 (s, 1H) 3.78 (s, 3H).

^{13}C{1H} NMR (101 MHz, $CDCl_3$) 161.9, 149.8, 140.5, 135.6, 135.0, 131.0, 130.4, 129.0, 127.5, 122.2, 121.6, 120.3, 114.7, 29.7.

The spectroscopic data matched those reported in the literature.[21]

1-Methyl-4-(naphthalen-2-yl)quinolin-2(1*H*)-one (2k)

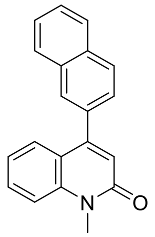

The title compound was obtained according to general procedure **A** from **1kb** (103 mg, 0.25 mmol). Purification by preparative TLC (SiO_2, 50% EtOAc in pentane) afforded **2k** as a white-yellow solid (51.3 mg, 0.18 mmol, 72%).

^{1}H-NMR (400 MHz, $CDCl_3$) δ 7.96 (d, *J* = 8.3, 1H), 7.94 – 7.87 (m, 3H), 7.64 – 7.55 (m, 4H), 7.53 (dd, *J* = 8.4, 1.7 Hz, 1H), 7.47 (dd, *J* = 8.9, 1.1 Hz, 1H), 7.17 (ddd, *J* = 8.1, 7.2, 1.1 Hz, 1H), 6.79 (s, 1H), 3.82 (s, 3H).

^{13}C{1H} NMR (101 MHz, $CDCl_3$) 162.1, 151.1, 140.5, 134.7, 133.3, 133.3, 130.8, 128.4, 128.3, 128.3, 128.0, 127.9, 126.9, 126.9, 126.8, 122.1, 121.7, 120.8, 114.6, 29.7.

HRMS: m/z for $C_{20}H_{16}NO$ $[M+H]^+$ calcd.: 286.1226, found: 286.1222.

1-Methyl-4-(1*H*-pyrrol-1-yl)quinolin-2(1*H*)-one (2l)

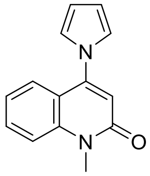

The title compound was obtained according to general procedure **A** from **1lb** (88.0 mg, 0.25 mmol). Purification by preparative TLC (SiO_2, 60% EtOAc in pentane) afforded **2l** as a brown solid (33.0 mg, 0.15 mmol, 59%).

^{1}H-NMR (400 MHz, $CDCl_3$) δ 7.79 – 7.68 (m, 1H), 7.64 (ddd, *J* = 8.6, 7.2, 1.5 Hz, 1H), 7.49 – 7.38 (m, 1H), 7.27 – 7.21 (m, 1H), 7.02 – 6.93 (m, 2H), 6.66 (s, 1H), 6.49 – 6.25 (m, 2H), 3.77 (s, 3H).

^{13}C{1H} NMR (101 MHz, $CDCl_3$) 162.3, 147.6, 140.7, 131.7, 125.6, 122.5, 122.3, 117.8, 116.0, 114.8, 110.8, 29.7.

HRMS: m/z for $C_{14}H_{13}N_2O$ $[M+H]^+$ calcd.: 225.1022, found: 225.1022.

1,4-Diphenylquinolin-2(1*H*)-one (2m)

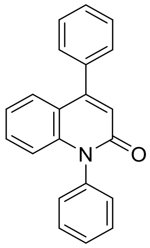

The title compound was obtained according to general procedure **A** from **1mb** (106 mg, 0.25 mmol). Purification by preparative TLC (SiO_2, 40% EtOAc in pentane) afforded **2m** as a white-yellow solid (70.0 mg, 0.24 mmol, 94%).

^{1}H-NMR (400 MHz, $CDCl_3$) δ 7.69 – 7.47 (m, 9H), 7.42 – 7.32 (m, 3H), 7.14 (ddd, *J* = 8.1, 7.1, 1.1 Hz, 1H), 6.77 (s, 1H), 6.74 (dd, *J* = 8.5, 1.1 Hz, 1H).

^{13}C{1H} NMR (101 MHz, $CDCl_3$) 162.0, 151.9, 141.6, 137.9, 137.2, 130.4, 130.3, 129.0, 129.0, 129.0, 128.9, 128.7, 127.4, 122.2, 121.8, 120.3, 116.5.

The spectroscopic data matched those reported in the literature.[23]

1-(4-(Dimethylamino)phenyl)-4-phenylquinolin-2(1*H*)-one (2n)

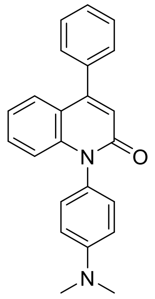

The title compound was obtained according to general procedure **A** from **1nb** (117 mg, 0.25 mmol). Purification by preparative TLC (SiO_2, 60% EtOAc in pentane) afforded **2n** as a yellow solid (78.8 mg, 0.23 mmol,93%).

^{1}H-NMR (400 MHz, $CDCl_3$) δ 7.58 (dd, *J* = 8.1, 1.5 Hz, 1H), 7.56 – 7.49 (m, 5H), 7.36 (ddd, *J* = 8.6, 7.1, 1.5 Hz, 1H), 7.22 – 7.17 (m, 2H), 7.13 (ddd, *J* = 8.2, 7.1, 1.1 Hz, 1H), 6.96 – 6.87 (m, 3H), 6.79 (s, 1H), 3.08 (s, 6H).

^{13}C{1H} NMR (101 MHz, $CDCl_3$) 162.6, 151.5, 150.6, 142.4, 137.4, 130.2, 129.3, 129.0, 128.8, 128.7, 127.2, 126.2, 122.0, 121.9, 120.3, 116.8, 113.5, 40.7.

HRMS: m/z for $C_{23}H_{21}N_2O$ $[M+H]^+$ calcd.: 341.1648, found: 341.1647.

1-Benzyl-4-phenylquinolin-2(1*H*)-one (2o)

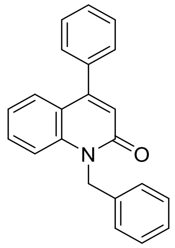

The title compound was obtained according to general procedure **A** from **1ob** (110 mg, 0.25 mmol). Purification by preparative TLC (SiO_2, 30% EtOAc in pentane) afforded **2o** as a yellow solid (69.6 mg, 0.22 mmol, 89%).

^{1}H-NMR (400 MHz, $CDCl_3$) δ 7.62 – 7.41 (m, 1H), 7.37 – 7.23 (m, 12H), 7.12 (ddd, *J* = 8.1, 7.1, 1.2 Hz, 1H), 6.79 (s, 1H), 5.64 (s, 2H).

^{13}C{1H} NMR (101 MHz, $CDCl_3$) 162.2, 151.6, 139.8, 137.2, 136.5, 130.7, 129.0, 128.9, 128.9, 128.7, 127.9, 127.4, 126.8, 122.1, 121.3, 120.9, 115.5, 46.1.

The spectroscopic data matched those reported in the literature.[24]

1-(Methoxymethyl)-4-phenylquinolin-2(1*H*)-one (2p)

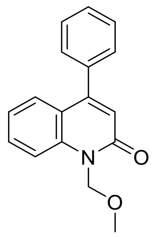

The title compound was obtained according to general procedure **A** from **1pb** (98.3 mg, 0.25 mmol). Purification by preparative TLC (SiO_2, 30% EtOAc in pentane) afforded **2p** as a yellow solid (58.7 mg, 0.22 mmol, 86%).

^{1}H-NMR (400 MHz, $CDCl_3$) δ 7.65 (d, *J* = 8.7, 1H), 7.60 – 7.47 (m, 5H), 7.47 – 7.40 (m, 2H), 7.18 (ddd, *J* = 8.2, 7.1, 1.2 Hz, 1H), 6.63 (s, 1H), 5.81 (s, 2H), 3.49 (s, 3H).

^{13}C{1H} NMR (101 MHz, $CDCl_3$) 162.6, 152.3, 139.6, 137.1, 130.9, 129.0, 128.9, 128.7, 127.8, 122.6, 121.1, 120.7, 115.7, 73.3, 56.8.

HRMS: m/z for $C_{17}H_{15}NNaO_2$ $[M+Na]^+$ calcd.: 288.0995, found: 288.0996.

1-(3-(2-Oxo-4-phenylquinolin-1(2*H*)-yl)propyl)-1*H*-pyrrole-2-carbonitrile (2q)

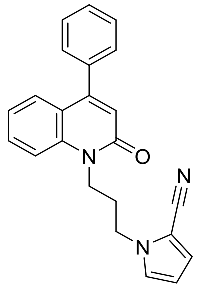

The title compound was obtained according to general procedure **A** from **1qb** (120 mg, 0.25 mmol). Purification by preparative TLC (SiO_2, 35% EtOAc in pentane) afforded **2q** as a brown solid (58.2 mg, 0.17 mmol, 66%).

^{1}H-NMR (400 MHz, $CDCl_3$) δ 7.60 – 7.46 (m, 5H), 7.44 – 7.37 (m, 2H), 7.23 – 7.13 (m, 2H), 7.07 (dd, *J* = 2.6, 1.6 Hz, 1H), 6.84 (dd, *J* = 4.0, 1.6 Hz, 1H), 6.22 (dd, *J* = 4.0, 2.7 Hz, 1H), 4.49 – 4.33 (m, 2H), 4.27 (t, *J* = 7.0 Hz, 2H), 2.35 (p, *J* = 7.1 Hz, 2H).

^{13}C{1H} NMR (101 MHz, $CDCl_3$) 161.9, 151.6, 139.2, 137.1, 131.1, 129.0, 128.9, 128.7, 128.3, 127.3, 122.2, 121.1, 121.0, 120.6, 114.0, 109.7, 103.6, 53.6, 46.8, 39.4, 29.3.

HRMS: m/z for $C_{23}H_{20}N_3O$ $[M+H]^+$ calcd.: 354.1601, found: 354.159.

4-Methylquinolin-2(1*H*)-one (2r)

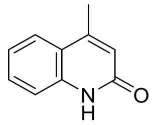

The title compound was obtained according to general procedure **A** from **1rb** (71.8 mg, 0.25 mmol). Purification by preparative TLC (SiO_2, 100% EtOAc) afforded **2r** as a yellow solid (16.1 mg, 0.10 mmol, 41%).

^{1}H-NMR (400 MHz, $CDCl_3$) δ 12.25 (s, 1H), 7.63 (dd, *J* = 8.0, 1.2 Hz, 1H), 7.52 – 7.42 (m, 2H), 7.21 (ddd, *J* = 8.2, 6.4, 1.9 Hz, 1H), 6.60 (d, *J* = 1.3 Hz, 1H), 2.46 (s, 3H).

^{13}C{1H} NMR (101 MHz, $CDCl_3$) 164.4, 149.4, 138.3, 130.6, 124.5, 122.6, 120.7, 120.7, 116.7, 19.2.

The spectroscopic data matched those reported in the literature.[20]

1,4-Dimethylquinolin-2(1*H*)-one (2s)

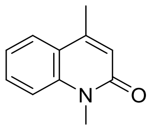

The title compound was obtained according to general procedure **A** with the addition of 20 mol% of morpholine (4.31 µL, 0.05 mmol) from **1se** (55.3 mg, 0.25 mmol). Purification by preparative TLC (SiO_2, 50% EtOAc in pentane) afforded **2s** as a yellow solid (25.2 mg, 0.15 mmol, 58%).

^{1}H-NMR (400 MHz, $CDCl_3$) δ 7.70 (dd, *J* = 8.0, 1.5 Hz, 1H), 7.57 (ddd, *J* = 8.6, 7.2, 1.5 Hz, 1H), 7.37 (d, *J* = 8.6, 1H), 7.32 – 7.21 (m, 1H), 6.60 (q, *J* = 1.2 Hz, 1H), 3.70 (s, 3H), 2.46 (d, *J* = 1.2 Hz, 3H).

^{13}C{1H} NMR (101 MHz, $CDCl_3$) 162.2, 146.5, 140.0, 130.6, 125.3, 122.0, 121.6, 121.3, 114.5, 29.3, 19.1.

The spectroscopic data matched those reported in the literature.[20]

4-Benzyl-1-methylquinolin-2(1*H*)-one (2t)

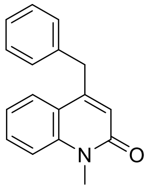

The title compound was obtained according to general procedure **A** from **1te** (74.4 mg, 0.25 mmol) with the addition of 20 mol% of morpholine (4.31 µL, 0.05 mmol). Purification by preparative TLC (SiO_2, 50% EtOAc in pentane) afforded **2t** as a yellow solid (32.1 mg, 0.13 mmol, 51%).

^{1}H-NMR (400 MHz, $CDCl_3$) δ 7.75 (dd, *J* = 8.1, 1.5 Hz, 1H), 7.55 (ddd, *J* = 8.6, 7.2, 1.5 Hz, 1H), 7.39 (dd, *J* = 8.5, 1.1 Hz, 1H), 7.35 – 7.29 (m, 2H), 7.26 – 7.17 (m, 4H), 6.51 (s, 1H), 4.17 (s, 2H), 3.72 (s, 3H).

^{13}C{1H} NMR (101 MHz, $CDCl_3$) 162.2, 148.6, 140.1, 137.5, 130.5, 128.9, 128.8, 126.8, 125.4, 122.0, 121.9, 120.6, 114.6, 38.4, 29.3.

The spectroscopic data matched those reported in the literature.[25]

4-Benzyl-1-methylquinolin-2(1*H*)-one (2u)

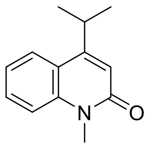

The title compound was obtained according to general procedure **A** from **1ue** (62.3 mg, 0.25 mmol) with the addition of 20 mol% of morpholine (4.31 µL, 0.05 mmol). Purification by preparative TLC (SiO_2, 50% EtOAc in pentane) afforded **2u** as a colorless oil (19.9 mg, 0.01 mmol, 40%).

^{1}H-NMR (400 MHz, $CDCl_3$) δ 7.82 (dd, *J* = 8.2, 1.5 Hz, 1H), 7.56 (ddd, *J* = 8.6, 7.1, 1.5 Hz, 1H), 7.39 (dd, *J* = 8.5, 1.2 Hz, 1H), 7.26 (ddd, *J* = 8.2, 7.3, 1.2 Hz, 1H), 6.66 (s, 1H), 3.71 (s, 3H), 3.40 (p, *J* = 6.7 Hz, 1H), 1.33 (d, *J* = 6.8 Hz, 6H).

^{13}C{1H} NMR (101 MHz, $CDCl_3$) 162.7, 156.2, 140.3, 130.3, 124.6, 122.0, 120.4, 117.2, 114.9, 29.4, 28.4, 22.5.

The spectroscopic data matched those reported in the literature.[26]

Two step one pot procedure 1

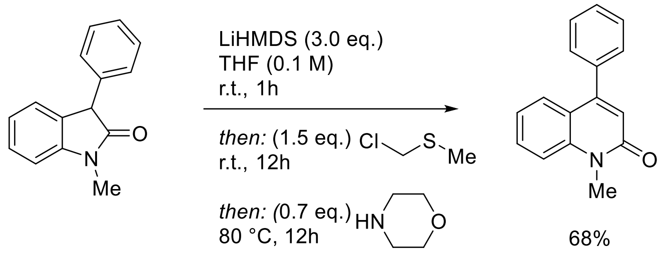

To 1-methyl-3-phenylindolin-2-one (55.8 mmol, 0.25 mmol, 1.00 eq.) in THF (2.5 mL) was added LiHMDS (125 mg, 0.75 mmol, 3.00 eq.). The resulting mixture was stirred for 1 h. Then, (chloromethyl)(methyl)sulfane (31.3 µL, 375 µmol, 1.50 eq.) was added and the mixture stirred for 12 h. Subsequently, morpholine (15.1 µL, 175 µmol, 0.70 eq.) was added and the mixture heated to 80 °C for 24 h. Work-up and purification of the resulting mixture was conducted as previously indicated for **2a**. The target compound was obtained in only reduced yield, compared to the single-step approach (40.0 mg, 0.17 mmol, 68%).

General procedure B

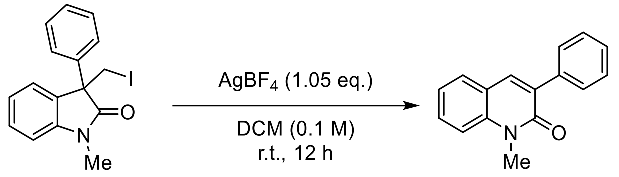

A flame-dried 4 mL drum vial in a glovebox was charged with oxindole (1.0 eq.) and $AgBF_4$ (1.05 eq.) and a magnetic stir bar. The vial was sealed with a septum-cap and removed from the glovebox. Then, DCM (0.1 M) was added through the septum. The resulting mixture was placed on a stirring plate at room temperature and stirred for 12 h. Upon completion, the mixture was filtered through a plug of silica, washed with EtOAc and subsequently volatiles were removed *in vacuo*. Purification by column chromatography or preparative TLC afforded the product.

General procedure C

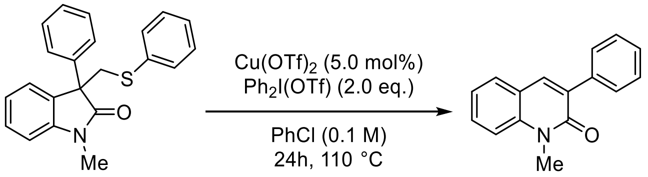

A flame-dried 4 mL drum vial in a glovebox was charged with oxindole (1.0 eq.), $Cu(OTf)_2$ (5.00 mol%), diphenyliodonium triflate (2.00 eq.) and chlorobenzene (0.1 M) and a magnetic stir bar. The vial was sealed with a septum-cap and removed from the glovebox. The resulting mixture was placed on a heated stirring plate at 110 °C and stirred for 24 h. Upon completion, the mixture was filtered through a plug of silica, residues of chlorobenzene were removed by washing with pentane, then washing with EtOAc released the product. Volatiles were removed *in vacuo*. Purification by column chromatography or preparative TLC afforded the product.

1-Methyl-3-phenylquinolin-2(1*H*)-one (8a)

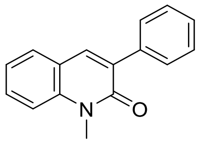

The title compound was obtained according to general procedure **B** from **1ab** (90.8 mg, 0.25 mmol). Purification by preparative TLC (SiO_2, 30% EtOAc in pentane) afforded **8a** as a white-yellow solid (51.0 mg, 0.22 mmol, 87%).

Alternatively, the title compound was also obtained according to general procedure **C** from **1aa** (86.4 mg, 0.25 mmol). Purification by preparative TLC (SiO_2, 20% EtOAc in pentane) afforded **8a** as a white-yellow solid (54.6 mg, 0.23 mmol, 93%).

^{1}H-NMR (400 MHz, $CDCl_3$) δ 7.80 (s, 1H), 7.73 (d, *J* = 7.4 Hz, 2H), 7.61 (d, *J* = 7.7, 1H), 7.57 (dd, *J* = 8.6, 7.2 Hz, 1H), 7.44 (t, *J* = 7.1 Hz, 2H), 7.42 – 7.35 (m, 2H), 7.29 – 7.20 (m, 1H), 3.81 (s, 3H).

^{13}C{1H} NMR (101 MHz, $CDCl_3$) 161.7, 139.8, 136.9 (overlap of two peaks), 132.6, 130.4, 129.1, 129.0, 128.3, 128.2, 122.3, 120.9, 114.1, 30.1.

The spectroscopic data matched those reported in the literature.[27]

6-Methoxy-1-methyl-3-phenylquinolin-2(1*H*)-one (8b)

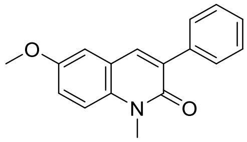

The title compound was obtained according to general procedure **B** from **1cb** (98.3 mg, 0.25 mmol). Purification by preparative TLC (SiO_2, 40% EtOAc in pentane) afforded **8b** as a white-yellow solid (54.0 mg, 0.20 mmol, 81%).

^{1}H-NMR (400 MHz, $CDCl_3$) δ 7.79 – 7.63 (m, 3H), 7.43 (tt, *J* = 6.6, 1.0 Hz, 2H), 7.40 – 7.34 (m, 1H), 7.30 (d, *J* = 9.2 Hz, 1H), 7.18 (dd, *J* = 9.2, 2.8 Hz, 1H), 7.05 (d, *J* = 2.9 Hz, 1H), 3.87 (s, 3H), 3.78 (s, 3H).

^{13}C{1H} NMR (101 MHz, $CDCl_3$) δ 161.2, 154.8, 137.0, 136.4, 134.4, 133.1, 129.1, 128.2, 128.2, 121.5, 119.1, 115.4, 110.5, 55.8, 30.2.

The spectroscopic data matched those reported in the literature.[28]

7-methoxy-1-methyl-3-phenylquinolin-2(1*H*)-one (8c)

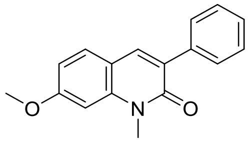

The title compound was obtained according to general procedure **B** from **1cb-isomer** (98.3 mg, 0.25 mmol). Purification by preparative TLC (SiO_2, 40% EtOAc in pentane) afforded **8c** as a white-yellow solid (62.0 mg, 0.23 mmol, 94%).

^{1}H-NMR (400 MHz, $CDCl_3$) δ 7.73 (s, 1H), 7.72 – 7.64 (m, 2H), 7.51 (d, *J* = 8.6 Hz, 1H), 7.45 – 7.38 (m, 2H), 7.38 – 7.32 (m, 1H), 6.84 (dd, *J* = 8.6, 2.3 Hz, 1H), 6.80 (d, *J* = 2.3 Hz, 1H), 3.93 (s, 3H), 3.76 (s, 3H).

^{13}C{1H} NMR (101 MHz, $CDCl_3$) δ 162.0, 161.7, 141.4, 137.2, 136.8, 130.4, 129.4, 129.0, 128.2, 127.8, 115.1, 109.9, 98.6, 55.7, 30.1.

The spectroscopic data matched those reported in the literature.[29]

1,6-Dimethyl-3-phenylquinolin-2(1*H*)-one (8d)

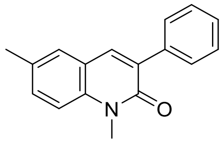

The title compound was obtained according to general procedure **B** from **1bb** (94.3 mg, 0.25 mmol). Purification by preparative TLC (SiO_2, 30% EtOAc in pentane) afforded **8d** as a white-yellow solid (43.2 mg, 0.17 mmol, 69%).

^{1}H-NMR (400 MHz, $CDCl_3$) δ 7.75 – 7.67 (m, 3H), 7.49 – 7.41 (m, 2H), 7.41 – 7.34 (m, 3H), 7.26 (d, *J* = 8.3 Hz, 1H), 3.77 (s, 3H), 2.43 (s, 3H).

$^{13}C\{1H\}$ **NMR (101 MHz, CDCl₃)** δ 161.5, 137.8, 137.1, 136.7, 132.5, 131.8, 131.6, 129.1, 128.7, 128.2, 128.1, 120.8, 114.0, 30.0, 20.7.

The spectroscopic data matched those reported in the literature.[28]

6-Fluoro-1-methyl-3-phenylquinolin-2(1*H*)-one (8e)

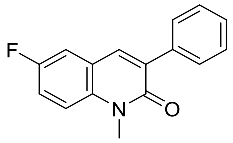

The title compound was obtained according to general procedure **B** from **1db** (95.3 mg, 0.25 mmol). Purification by preparative TLC (SiO_2, 30% EtOAc in pentane) afforded **8e** as a white-yellow solid (20.1 mg, 0.08 mmol, 32%).

^{1}H**-NMR (400 MHz, $CDCl_3$)** δ 7.73 (s, 1H), 7.71 – 7.67 (m, 2H), 7.48 – 7.27 (m, 6H), 3.79 (s, 3H).

$^{13}C\{1H\}$ **NMR (101 MHz, $CDCl_3$)** δ 161.3, 158.0 (d, *J* = 242.2 Hz), 136.6, 136.3 (d, *J* = 1.5 Hz), 135.8 (d, *J* = 3.2 Hz), 134.0, 129.1, 128.5, 128.3, 121.7 (d, *J* = 8.6 Hz), 118.1 (d, *J* = 23.8 Hz), 115.7 (d, *J* = 8.0 Hz), 113.8 (d, *J* = 22.5 Hz), 30.4.

$^{19}F\{1H\}$ **NMR (376 MHz, $CDCl_3$)** δ -121.2.

The spectroscopic data matched those reported in the literature.[30]

1,3-Diphenylquinolin-2(1*H*)-one (8f)

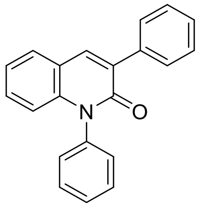

The title compound was obtained according to general procedure **B** from **1mb** (106 mg, 0.25 mmol). Purification by preparative TLC (SiO_2, 20% EtOAc in pentane) afforded **8f** as a white-yellow solid (70.1 mg, 0.24 mmol, 94%).

^{1}H**-NMR (400 MHz, $CDCl_3$)** δ 7.97 (s, 1H), 7.89 – 7.78 (m, 2H), 7.67 (dd, *J* = 7.7, 1.5 Hz, 1H), 7.64 – 7.59 (m, 2H), 7.57 – 7.50 (m, 1H), 7.47 – 7.32 (m, 6H), 7.25 – 7.17 (m, 1H), 6.74 – 6.62 (m, 1H).

$^{13}C\{1H\}$ **NMR (101 MHz, $CDCl_3$)** δ 161.7, 140.8, 138.2, 137.5, 136.3, 132.7, 130.2, 129.9, 129.2, 129.0, 128.9, 128.5, 128.3, 128.2, 122.5, 120.7, 115.9.

The spectroscopic data matched those reported in the literature.[31]

1,3-Dimethylquinolin-2(1*H*)-one (8g)

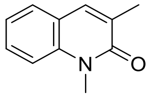

The title compound was obtained according to general procedure **C** from **1sa** (70.8 mg, 0.25 mmol). Purification by preparative TLC (SiO_2, 30% EtOAc in pentane) afforded **8g** as a white solid (17.8mg, 0.10 mmol, 41%).

^{1}H-NMR (400 MHz, $CDCl_3$) δ 7.55 (s, 1H), 7.53 – 7.47 (m, 2H), 7.33 (d, *J* = 9.0 Hz, 1H), 7.23 – 7.17 (m, 1H), 3.74 (s, 3H), 2.26 (d, *J* = 1.3 Hz, 3H).

^{13}C{1H} NMR (101 MHz, $CDCl_3$) δ 163.1, 139.2, 135.8, 130.2, 129.4, 127.9, 122.1, 120.9, 114.0, 29.8, 17.9.

The spectroscopic data matched those reported in the literature. [32]

Two step one pot procedure 2

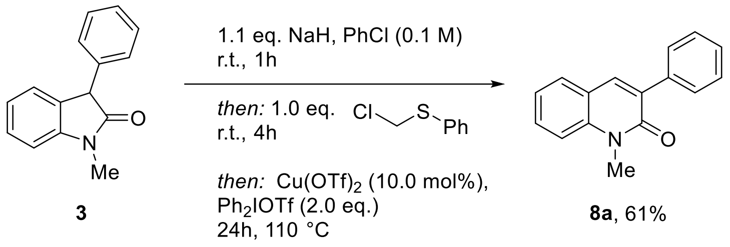

To 1-methyl-3-phenylindolin-2-one (55.8 mmol, 0.25 mmol, 1.00 eq.) in PhCl (2.5 mL) was added NaH (60% in mineral oil, 11.0 mg, 0.28 mmol, 1.10 eq.). The resulting mixture was stirred for 1 h. Then, [(Chloromethyl)sulfanyl]benzene (32.7 µL, 250 µmol, 1.00 eq.) was added and the mixture stirred for 4 h. Subsequently, $Cu(OTf)_2$ (9.04 mg, 25.0 µmol , 10.0 mol%) and diphenyliodonium triflate (215 mg, 0.50 mmol, 2.00 eq.) were added and the mixture heated to 110 °C for 24 h. Work-up and purification of the resulting mixture was conducted as previously indicated for **8a**. The target compound was obtained in only reduced yield, compared to the single-step approach (35.7 mg, 0.15 mmol, 61%).

Drug derived molecules

Linopiridine

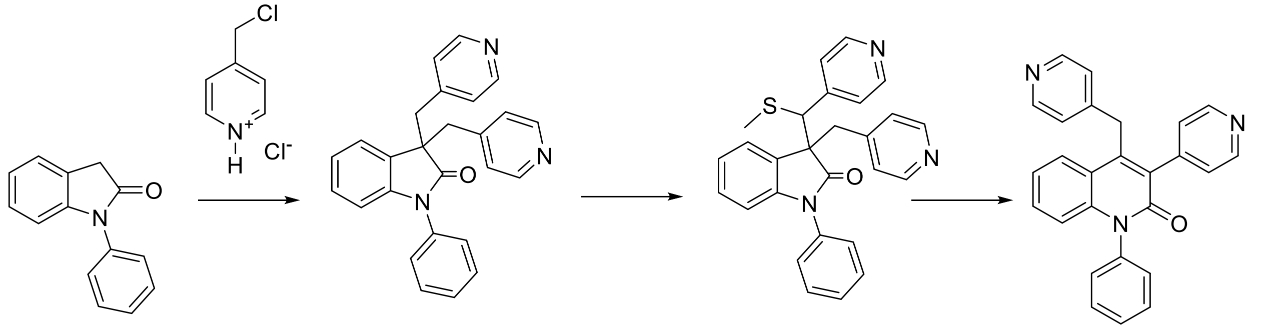

linopiridine **1ve** **2v**

Linopiridine was synthesized according to the following procedure:

To 1-phenylindolin-2-one (1.53 g, 7.32 mmol, 1.00 eq.) in DMF (24 mL) at 0 °C was added NaH (1.46 g, 60% in mineral oil, 36.6 mmol, 5.00 eq.) portion wise. The resulting mixture was stirred for 30 min, then 4-(chloromethyl)pyridin-1-ium chloride (2.64 g, 16.1 mmol, 2.20 eq.) was added slowly. The resulting mixture was allowed to warm to room temperature overnight. To the mixture was added water and sat. NH_4Cl until the residue was slightly acidic. The aqueous phase was extracted with DCM (3 × 50 mL), and the combined organic residues dried over $MgSO_4$. Volatiles were removed *in vacuo*. Upon filtration through a plug of silica (4% MeOH in DCM as eluent), and repeated removal of the solvent, the crude mixture was dissolved in 1:1 *i*PrOH:H_2O. Then, water was added, and the resulting precipitate was collected as a brownish solid of linopiridine (1.56 g, 3.98 mmol, 54%).

^{1}H-NMR (400 MHz, $CDCl_3$) δ 8.65 – 8.17 (m, 4H), 7.41 (dd, *J* = 7.4, 1.3 Hz, 1H), 7.38 – 7.30 (m, 3H), 7.13 (td, *J* = 7.4, 0.9 Hz, 1H), 7.09 – 7.00 (m, 1H), 6.91 – 6.83 (m, 4H), 6.64 – 6.51 (m, 2H), 6.31 – 6.22 (m, 1H), 3.48 (d, *J* = 12.7 Hz, 2H), 3.23 (d, *J* = 12.7 Hz, 2H).

^{13}C{1H} NMR (101 MHz, $CDCl_3$) 176.6, 149.5, 144.5, 143.9, 133.7, 129.8, 128.9, 128.5, 128.5, 126.4, 125.3, 123.9, 122.9, 109.7, 55.6, 43.2.

The spectroscopic data matched those reported in the literature.[33]

1-phenyl-3-(pyridin-4-yl)-4-(pyridin-4-ylmethyl)quinolin-2(1H)-one (2v)

The thiomethylation and rearrangement was achieved through the following procedure:

To linopiridine (200 mg, 0.51 mmol, 1.00 eq.) in THF (5.1 mL) at -78 °C was added *n*-BuLi (351 µL of 1.6 M solution in hexanes, 562 µmol, 1.10 eq.). After stirring for 20 min, dimethyldisulfide (54.5 µL, 613 µL, 1.20 eq.) was added and the reaction mixture allowed to warm to room temperature and stir overnight. Then, water was added, the aqueous phase was extracted with EtOAc (3 × 30 mL), and the combined organic residues dried over $MgSO_4$. Volatiles were removed *in vacuo* and the resulting solid **1ue** directly used in the next step.

To the crude mixture was added LiHMDS (214 mg, 1.28 mmol, 2.50 eq.), morpholine (8.82 µL, 102 µmol, 0.20 eq.), and THF (5.1 mL) inside a glovebox. Upon removal, the reaction was heated to 80 °C for 16 h. To the reaction mixture was added MeOH and volatiles were removed *in vacuo*. The title compound was obtained upon purification by preparative TLC (SiO_2, 6% MeOH in DCM) as brownish crystals (119 mg, 305 mmol, 60% over two steps).

Crystals for X-ray diffractometry were obtained through dissolving in DCM and slow diffusion with pentane.

^{1}H-NMR (400 MHz, $CDCl_3$) δ 8.65 – 8.47 (m, 4H), 7.65 – 7.57 (m, 2H), 7.57 – 7.50 (m, 2H), 7.40 – 7.32 (m, 3H), 7.23 – 7.19 (m, 2H), 7.14 (ddd, *J* = 8.2, 7.2, 1.2 Hz, 1H), 7.10 – 7.04 (m, 2H), 6.80 (dd, *J* = 8.8, 1.1 Hz, 1H), 4.19 (s, 2H).

^{13}C{1H} NMR (101 MHz, $CDCl_3$) 160.8, 150.4, 150.0, 147.7, 143.8, 143.4, 141.0, 137.6, 132.5, 130.8, 130.4, 129.2, 128.9, 126.3, 124.9, 123.3, 122.9, 119.7, 116.9, 35.6.

Chrystalographic data is provided below.

Doliracetam

***N,N*-dibenzyl-2-(3-(iodomethyl)-2-oxo-3-phenylindolin-1-yl)acetamide**

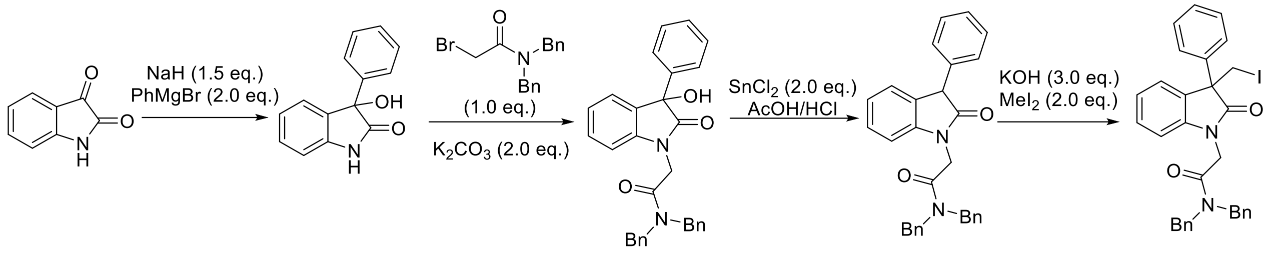

Benzyl protected doliracetam was synthesized according to the following procedure, which was adapted from the literature.[18]

To a solution of indoline-2,3-dione (1.47 g, 10.0 mmol, 1.00 eq.) in anhydrous THF (20 mL) was added NaH (600 mg 60% in mineral oil, 15.0 mmol, 1.50 eq.) at -15 °C and the resulting mixture was stirred for 30 min. PhMgBr (6.67 mL 3 M PhMgBr in THF, 20.0 mmol, 2.00 eq.) was then added dropwise to the mixture and the reaction mixture was allowed to warm to room temperature. After 1 , the reaction mixture was quenched with NH_4Cl (30 mL) and extracted with Et_2O (3 × 50 mL). The combined extracts were washed with brine and dried over Na_2SO_4, and then concentrated *in vacuo*.

The resulting crude mixture was redissolved in MeCN (20 mL) and *N,N*-dibenzyl-2-bromoacetamide (3.18 g, 10.0 mmol, 1.00 eq.) and K_2CO_3 (2.76 g, 20.0 mmol, 2.00 eq.) were added. Upon heating to 80 °C for 16 h, equal amounts of water and EtOAc (total 60 mL) were added, and the aqueous phase extracted with EtOAc (3 × 50 mL). The combined extracts were dried over $MgSO_4$, and then concentrated *in vacuo*.

The crude mixture was redissolved in AcOH/HCl (60 mL/4.0 mL) and $SnCl_2$ (3.79 g, 20.0 mmol, 2.00 eq.) was added at room temperature. Then, the mixture was heated to reflux for 4 h. The mixture was diluted with H_2O (20 mL) and extracted with EtOAc (100 mL). The organic phase was washed with sat. Na_2CO_3 solution and brine, dried over Na_2SO_4, and concentrated *in vacuo*. The resulting benzyl-doliracetam was purified by column chromatography (SiO_2, 0 to 30% EtOAc in hexane) to sufficient purity to proceed (3.20 g, 7.17 mmol, 72% over three steps).

Benzyl doliracetam (3.20 g, 7.17 mmol, 1.00 eq.) was redissolved in DMF (14 mL) and diiodomethane (1.16 mL, 14.3 mmol, 2.00 eq.) was added. The reaction was degassed through continuous bubbling with nitrogen. Then, KOH (483 mg, 8.60 mmol, 3.00 eq.) was added. The resulting mixture was stirred for 6 h at room temperature. Equivalent amounts of water and EtOAc (total 40 mL) were added, and the organic phase collected. The aqueous phase was washed with EtOAc (3 x 30 mL). The combined organic residues were dried over $MgSO_4$ and volatiles removed *in vacuo.* Purification by column chromatography (SiO_2, 0 to 18% EtOAc in pentane) afforded the title product **1vb** as a brownish solid (2.29 g, 3.90 mmol, 55%)

^{1}H-NMR (400 MHz, CDCl$_3$) δ 7.48 – 7.27 (m, 13H), 7.22 – 7.12 (m, 5H), 7.03 – 6.87 (m, 1H), 4.73 (d, *J* = 16.1 Hz, 1H), 4.69 – 4.45 (m, 5H), 3.94 (d, *J* = 9.9 Hz, 1H), 3.85 (d, *J* = 9.9 Hz, 1H).

^{13}C{1H} NMR (101 MHz, CDCl$_3$) 176.4, 166.7, 143.3, 138.0, 136.6, 135.9, 131.2, 129.3, 129.3, 129.0, 128.8, 128.6, 128.1, 128.1, 127.8, 127.2, 126.5, 125.0, 123.1, 109.7, 56.6, 49.7, 49.0, 42.5, 10.3.

HRMS: m/z for $C_{31}H_{28}IN_2O_2$ $[M+H]^+$ calcd.: 587.119, found: 587.1181.

N-benzoyl-N-(2-(2-oxo-4-phenylquinolin-1(2H)-yl)acetyl)benzamide (2w)

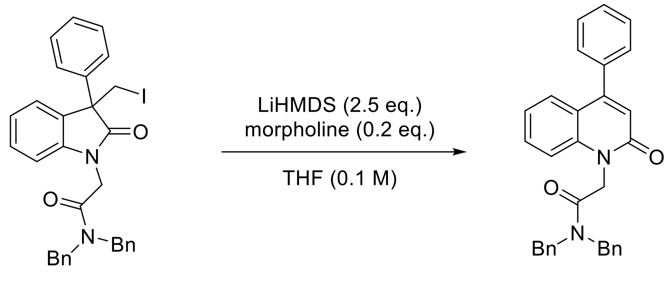

The title compound was obtained according to general procedure **A** with the addition of 20 mol% of morpholine (4.31 µL, 0.05 mmol) from **1wb** (147 mg, 0.25 mmol). Purification by column chromatography (SiO_2, 30% EtOAc in pentane) afforded **2w** as white needles (71.1 mg, 0.16 mmol, 62%).

^{1}H-NMR (400 MHz, $CDCl_3$) δ 7.57 – 7.45 (m, 5H), 7.42 – 7.27 (m, 12H), 7.20 – 7.11 (m, 2H), 6.62 (s, 1H), 5.27 (s, 2H), 4.70 (s, 2H), 4.68 (s, 2H)..

^{13}C{1H} NMR (101 MHz, $CDCl_3$) 167.7, 161.7, 151.8, 140.3, 137.3, 136.9, 136.2, 130.8, 129.3, 129.1, 128.9, 128.8, 128.7, 128.7, 128.1, 128.0, 127.8, 126.5, 122.2, 120.8, 120.8, 114.7, 49.7, 44.5.

HRMS: m/z for $C_{31}H_{27}N_2O_2$ $[M+H]^+$ calcd.: 459.2067, found: 459.2068.

N,N-dibenzyl-2-(2-oxo-3-phenylquinolin-1(2H)-yl)acetamide (8h)

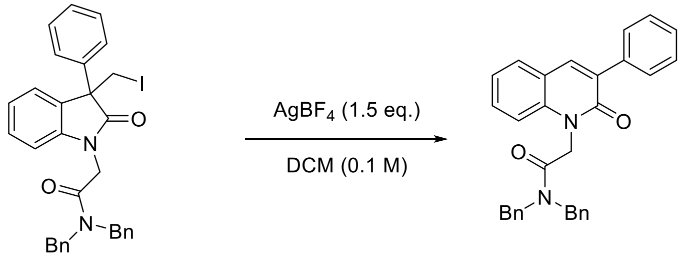

The title compound was obtained according to general procedure **B** from **1wb** (147 mg, 0.25 mmol). Purification by preparative TLC (SiO_2, 30% EtOAc in pentane) afforded **8h** as a white-yellow solid (49.0 mg, 0.11 mmol, 43%).

^{1}H-NMR (400 MHz, $CDCl_3$) δ 7.79 (s, 1H), 7.72 – 7.64 (m, 2H), 7.60 (dd, J = 7.8, 1.5 Hz, 1H), 7.51 (ddd, J = 8.7, 7.3, 1.5 Hz, 1H), 7.45 – 7.41 (m, 2H), 7.41 – 7.27 (m, 10H), 7.25 – 7.21 (m, 2H), 7.13 (d, J = 8.4 Hz, 1H), 5.26 (s, 2H), 4.70 (s, 2H), 4.68 (s, 2H).

^{13}C{1H} NMR (101 MHz, $CDCl_3$) 167.7, 161.4, 139.6, 137.7, 136.9, 136.7, 136.2, 132.1, 130.4, 129.3, 129.2, 129.1, 128.9, 128.6, 128.3, 128.2, 128.0, 127.8, 126.5, 122.5, 121.1, 114.3, 49.9, 49.6, 44.9.

HRMS: m/z for $C_{31}H_{26}N_2NaO_2$ $[M+Na]^+$ calcd.: 481.1886, found: 481.1886.

YWI92

YWI 91 was prepared according to the literature.[34]

3-(Iodomethyl)-3-(4-methoxyphenyl)-1-methylindolin-2-one (1xb)

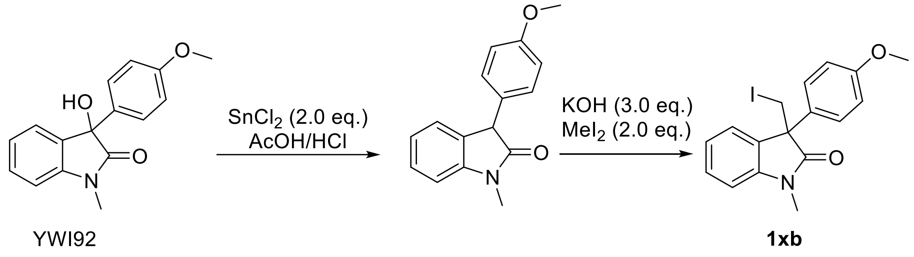

The title compound was synthesized according to the following procedure. The deoxygenation was adapted from the literature.[18]

To a solution of the YW192 (1.42 g, 5.27 mmol, 1.00 eq.) in AcOH/HCl (32 mL/2.2 mL), $SnCl_2$ (2.00 g, 10.5 mmol, 2.00 eq.) was added at room temperature. Then the mixture was heated to reflux for 4 h. Then, the mixture was diluted with H_2O (20 mL) and extracted with EtOAc (100 mL). The organic phase was washed with sat. Na_2CO_3 solution and brine, dried over Na_2SO_4, and concentrated *in vacuo*. Purification by column chromatography (SiO_2, 10 to 20% EtOAc in hexane) afforded the intermediate as a yellowish solid (1.30 g, 5.10 mmol, 97%)

The intermediate (1.30 g, 5.10 mmol) was redissolve in DMF (10 mL) and diiodomethane (828 µL, 10.30 mmol, 2.00 eq.) was added. The reaction was degassed through continuous bubbling with nitrogen. Then, KOH (1.01 g, 18.0 mmol, 3.00 eq.) was added. The resulting mixture was stirred for 4 h at room temperature. Equivalent amounts of water and EtOAc (total 20 mL) were added, and the organic phase collected. The aqueous phase was washed with EtOAc (3 x 20 mL). The combined organic residues were dried over $MgSO_4$ and volatiles removed *in vacuo.* Purification by column chromatography (SiO_2, 5 to 15% EtOAc in hexane) afforded the title product **1xb** as a yellow solid (1.68 g, 4.27 mmol, 83%).

^{1}H-NMR (400 MHz, $CDCl_3$) δ 7.44 – 7.34 (m, 4H), 7.19 (td, *J* = 7.6, 1.0 Hz, 1H), 6.93 (ddd, *J* = 7.8, 1.0, 0.6 Hz, 1H), 6.85 – 6.81 (m, 2H), 4.00 (d, *J* = 9.8 Hz, 1H), 3.77 (s, 3H), 3.72 (d, *J* = 9.8 Hz, 1H), 3.23 (s, 3H).

^{13}C{1H} NMR (101 MHz, $CDCl_3$) 176.6, 159.5, 144.2, 131.1, 129.8, 129.2, 128.5, 125.1, 122.8, 114.2, 108.8, 56.1, 55.4, 26.7, 11.0.

HRMS: m/z for $C_{17}H_{16}INNaO_2$ $[M+Na]^+$ calcd.: 416.0118, found: 416.011.

4-(4-Methoxyphenyl)-1-methylquinolin-2(1H)-one (2x)

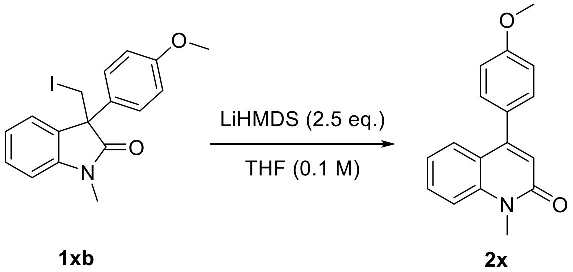

The title compound was obtained according to general procedure **A** from **1xb** (98.3 mg, 0.25 mmol). Purification by preparative TLC (SiO_2, 20% EtOAc in hexane) afforded **2x** as white needles (35.0 mg, 0.13 mmol, 53%).

^{1}H-NMR (400 MHz, $CDCl_3$) δ 7.65 – 7.55 (m, 2H), 7.43 (d, *J* = 8.9, 1H), 7.39 – 7.33 (m, 2H), 7.18 (ddd, *J* = 8.2, 7.2, 1.1 Hz, 1H), 7.06 – 6.99 (m, 2H), 6.67 (s, 1H), 3.89 (s, 3H), 3.78 (s, 3H).

^{13}C{1H} NMR (101 MHz, $CDCl_3$) 162.2, 160.2, 150.8, 140.5, 130.7, 130.4, 129.5, 127.9, 122.0, 121.2, 120.9, 114.6, 114.2, 55.5, 29.6.

The spectroscopic data matched those reported in the literature.[21]

3-(4-Methoxyphenyl)-1-methylquinolin-2(1H)-one (8i)

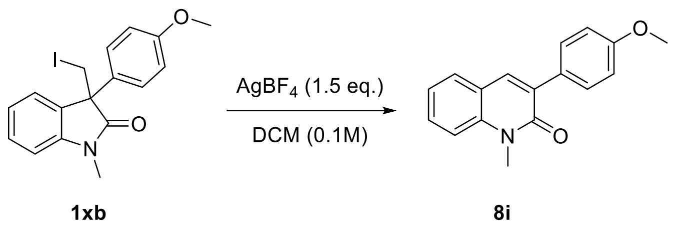

The title compound was obtained according to general procedure **B** from **1xb** (98.3 mg, 0.25 mmol). Purification by preparative TLC (SiO_2, 20% EtOAc in pentane) afforded **8i** as a white-yellow solid (52.0 mg, 0.20 mmol, 78%).

^{1}H-NMR (400 MHz, $CDCl_3$) δ 7.77 (s, 1H), 7.71 – 7.66 (m, 2H), 7.60 (dd, *J* = 7.8, 1.5 Hz, 1H), 7.55 (ddd, *J* = 8.6, 7.2, 1.5 Hz, 1H), 7.40 – 7.36 (m, 1H), 7.25 – 7.21 (m, 1H), 7.02 – 6.94 (m, 2H), 3.86 (s, 3H), 3.80 (s, 3H).

^{13}C{1H} NMR (101 MHz, $CDCl_3$) 161.9, 159.7, 139.6, 135.9, 132.2, 130.3, 130.1, 129.4, 128.8, 122.3, 121.1, 114.1, 113.8, 55.5, 30.1.

The spectroscopic data matched those reported in the literature.[28]

Tipifarnib

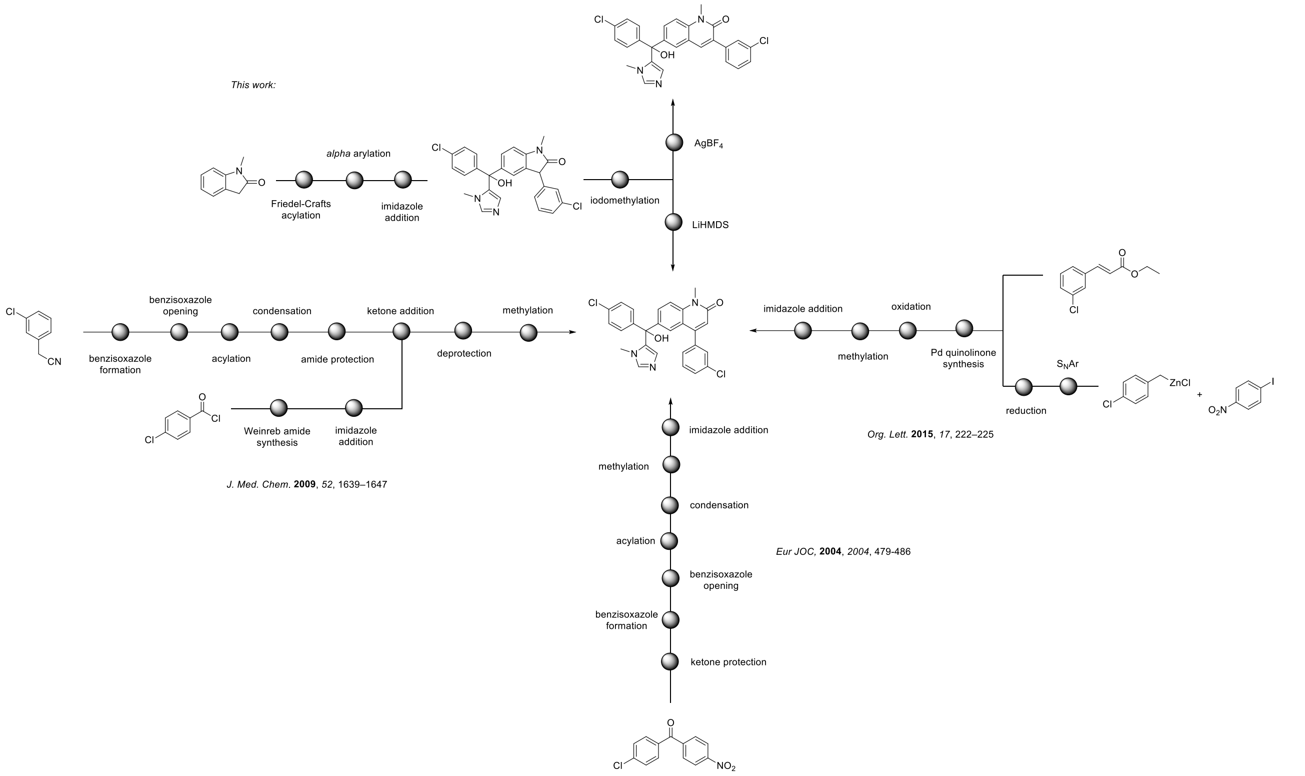

3-(3-chlorophenyl)-5-((4-chlorophenyl)(hydroxy)(1-methyl-1*H*-imidazol-5-yl)methyl)-3-(iodomethyl)-1-methylindolin-2-one (1xb)

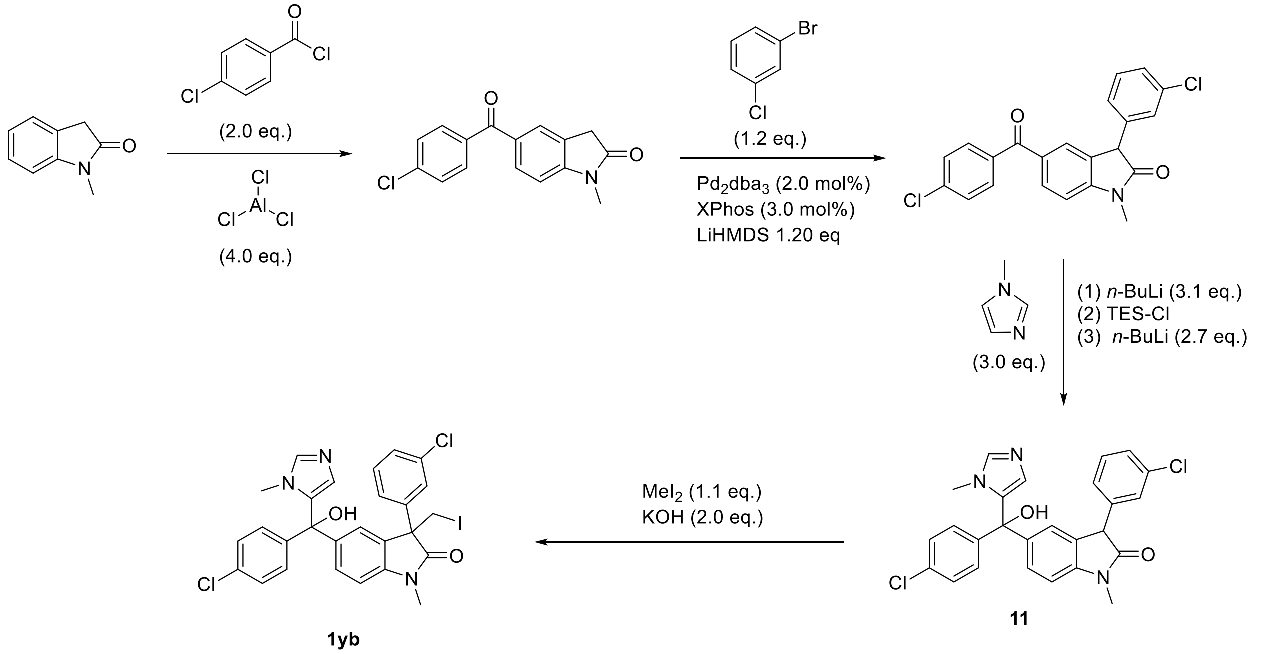

3-(3-chlorophenyl)-5-((4-chlorophenyl)(hydroxy)(1-methyl-1H-imidazol-5-yl)methyl)-1-methylindolin-2-one (11)

To $AlCl_3$ (2.67 g, 20.0 mmol, 4.00 eq.) in a Schlenk tube under nitrogen was added 4-chlorobenzoyl chloride (1.28 ml, 10.0 mmol, 2.00 eq.) and DCE (10 mL), the resulting mixture was stirred for 15 min. Then, 1-methylindolin-2-one (736 mg, 5.00 mmol, 1.00 eq.) was added in portions. Subsequently, the reaction was heated at 60 °C and stirred for 14 hours. Upon cooling to room temperature the mixture was poured into ice-water and 15% NaOH (aq.) (5 mL) was added, the biphasic mixture was stirred for 15 minutes to obtain visible phase separation. The organic phase was collected and the aqueous phase washed with DCM (4 × 30 mL). The combined organic residues were dried over $MgSO_4$ and volatiles removed *in vacuo*. Purification by column chromatography (SiO_2, 10 to 30% EtOAc in hexane) afforded the oxindole intermediate (741 mg, 2.59 mmol, 52%).

This intermediate (407 mg, 1.42 mmol, 1.00 eq.) was transferred into a glovebox and Pd_2dba_3 (16.4 mg, 28.5 µmol, 0.02 eq.) and XPhos (20.4 mg, 42.7 µmol, 0.03 eq.) were added. Upon removal from the glovebox, THF (2.9 mL) and 1-bromo-3-chloro-benzene (201 µL, 1.71 mmol, 1.20 eq.) were added and the mixture was heated to 70 °C. After 5 min, LiHMDS (1 M solution, 1.71 mL, 1.71 mmol, 1.20 eq.) was added and the reaction stirred for 12 h. Upon cooling to room temperature, the mixture was filtered through a plug of celite and washed with EtOAc. Volatiles were removed *in vacuo* and the crude mixture purified by column chromatography (SiO_2, 10 to 30% EtOAc in hexane) to afford the *alpha*-arylated intermediate (173 mg, 0.44 mmol, 31%).

The imidazole addition procedure was adapted from the literature.[35]

A flame-dried Schlenk tube was charged with a stir bar and over pressurized with dry nitrogen gas and *N*-methylimidazole (384 µL, 4.85 mmol, 3.00 eq.) was added. The flask was sealed with a rubber septum, and THF (2.15 mL) was added. The solution was stirred for about 10 min, and then the temperature was lowered to −78 °C and stirred for an additional 10 min. Then, *n*-BuLi (1.6 M in hexane, 3.13 mL, 5.01 mmol, 3.10 eq.) was added dropwise through the septum over a period of 10 min. A slight color change to pale yellow was observed. This was allowed to stir for 45 min, and then TESCl (829 µL, 4.93 mmol, 3.05 eq.) was added dropwise over 5 min. The reaction was allowed to stir for 1 h at −78 °C, at which point *n*-BuLi (1.6 M in hexane, 2.73 mL, 4.36 mmol, 2.70 eq.) was added dropwise through the septum over a period of 10 min and allowed to stir at −78 °C for an additional 45 min. Then, the previously obtained *alpha*-arylated intermediate (640 mg, 1.62 mmol, 1.00 eq.) in THF (1.1 mL) was added. The mixture was left to stir for 12 h. The reaction was quenched by the addition of 1 M HCl until the pH of the aqueous phase was no longer basic, as indicated by litmus paper, and then allowed to stir for 1 h. The pH of the aqueous phase was adjusted to above 8 with 1.5 M NaOH, and the mixture was partitioned between DCM and water (total 50 mL). The organic phase was washed with brine and dried with anhydrous MgSO4. Volatiles were removed *in vacuo*. Purification by column chromatography (SiO_2, 0 to 4% MeOH in DCM) afforded the oxindole **11** as a 1:1 mixture of diastereomers (677 mg, 1.42 mmol, 88%). Due to the presence of a 1:1 mixture of diastereomers, and the overlay of many aromatic signals, distinguishing different splitting patterns from the presence of diastereomers proved difficult. In the 13C, the apparent doublets of the diastereomers are indicated by an average shift, followed by the individual chemical shifts for each peak. The provided data was, however, sufficient to determine the purity and presence of the postulated product.

^{1}H-NMR (400 MHz, $CDCl_3$) δ 7.40 – 6.71 (m, 13H), 6.27 (apparent d, 6.28, 6.25, 1H), 4.53 (apparent d, 4.54, 4.52, 1H), 3.35 (apparent d, 3.35, 3.35, 3H), 3.23 (apparent d, 3.23, 3.22, 3H).

^{13}C{1H} NMR (101 MHz, $CDCl_3$) 175.4, 144.2 (144.2, 144.2), 143.5 (143.6, 143.5), 139.6 (139.6, 139.6), 138.3 (138.3, 138.3), 134.7 (134.8, 134.7), 133.7 (133.8, 133.7), 130.3, 128.5 (128.5, 128.5), 128.4, 128.3, 128.3, 128.2, 128.2, 128.2, 128.0, 127.4, 127.0 (127.1, 127.0), 123.8 (123.9, 123.6), 108.0 (108.0, 107.9), 76.4 (76.5, 76.3), 51.6, 33.8 (33.8, 33.8), 26.8.

HRMS: m/z for $C_{26}H_{22}Cl_2N_3O_2$ $[M+H]^+$ calcd.: 478.1084, found: 478.1080.

3-(3-chlorophenyl)-5-((4-chlorophenyl)(hydroxy)(1-methyl-1H-imidazol-5-yl)methyl)-3-(iodomethyl)-1-methylindolin-2-one (1yb)

The obtained oxindole **11** (677 mg, 1.42 mmol, 1.00 eq.) was redissolved in DMF (2.8 mL) and diiodomethane (126 µL, 1.56 mmol, 1.10 eq.) was added. The reaction was degassed through continuous bubbling with nitrogen. Then, KOH (159 mg, 2.83 mmol, 3.00 eq.) was added. The resulting mixture was stirred for 2 h at room temperature. Equivalent amounts of water and EtOAc (15 mL each) were added, and the organic phase collected. The aqueous phase was washed with EtOAc (3 x 15 mL). The combined organic residues were dried over $MgSO_4$ and volatiles removed *in vacuo.* The obtained crude residue was purified by column chromatography (SiO_2, 0 to 3% MeOH in DCM) to obtain **1yb** as a yellow solid (604 mg, 0.98 mmol, 69%). Again a 1:1 mixture of diastereomers was obtained. The changes observed in the NMR correlate to the expected signals.

^{1}H-NMR (400 MHz, $CDCl_3$) δ 7.53 – 7.11 (m, 12H), 6.87 (apparent dd, 6.88 (d, *J* = 8.1 Hz, H^a), 6.86 (d, *J* = 8.3 Hz, H^b) 1H), 6.28 (apparent d, 6.30, 6.27, 1H), 3.93 (apparent dd, 3.94 (d, *J* = 8.4 Hz, H^a), 3.92 (d, *J* = 8.6 Hz, H^b), 1H), 3.66 – 3.53 (apparent dd, 3.62 (d, *J* = 9.8 Hz, H^a), 3.56 (d, *J* = 9.8 Hz, H^b), 1H), 3.38 (apparent d, 3.38, 3.37, 3H), 3.21 (apparent d, 3.21, 3.21, 3H).

^{13}C{1H} NMR (101 MHz, $CDCl_3$) 175.6 (175.7, 175.6), 143.9 (143.9, 143.8), 143.4 (143.4, 143.3), 140.0, 139.8 (139.8, 139.8), 139.2 (139.2, 139.1), 136.1 (136.2, 136.0), 134.6 (134.6, 134.5), 133.5, 130.2 (130.2, 130.2), 130.2 (130.2, 130.1), 130.0, 128.4 (128.4, 128.4), 128.3 (128.4, 128.3), 128.1 (128.2, 128.1), 127.6, 127.3 (127.4, 127.3), 125.4 (125.4, 125.4), 123.7 (123.8, 123.6), 108.5 (108.5, 108.5), 76.1 (76.1, 76.0), 56.3 (56.4, 56.3), 33.7 (33.8, 33.6), 26.8, 10.0.

HRMS: m/z for $C_{27}H_{22}Cl_2IN_3NaO_2$ $[M+Na]^+$ calcd.: 640.0026, found: 640.0024.

4-(3-Chlorophenyl)-6-((4-chlorophenyl)(hydroxy)(1-methyl-1H-imidazol-5-yl)methyl)-1-methylquinolin-2(1*H*)-one (2y)

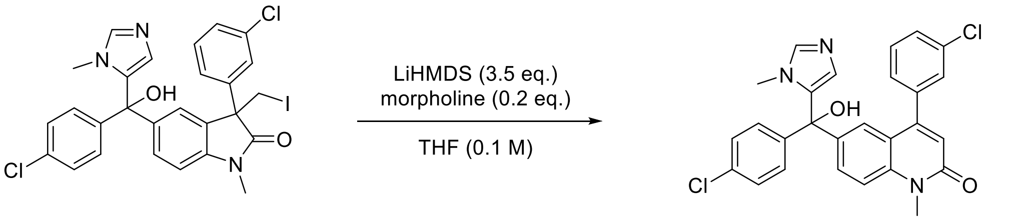

1yb **2y**

The title compound was obtained according to general procedure **A** with the addition of 20 mol% of morpholine (4.31 µL, 0.05 mmol) from **1yb** (155 mg, 0.25 mmol). Purification by column chromatography (SiO_2, 2% to 5% MeOH in DCM) afforded **2y** as a brownish solid (53.6 mg, 0.11 mmol, 44%).

^{1}H-NMR (400 MHz, $CDCl_3$) δ 7.63 (dd, *J* = 8.8, 2.2 Hz, 1H), 7.43 – 7.26 (m, 7H), 7.22 – 7.13 (m, 3H), 7.05 (dt, *J* = 7.5, 1.4 Hz, 1H), 6.57 (s, 1H), 6.23 (s, 1H), 3.70 (s, 3H), 3.37 (s, 3H).

^{13}C{1H} NMR (101 MHz, $CDCl_3$) 161.7, 149.4, 142.8, 140.1, 139.6, 139.1, 138.1, 135.9, 134.6, 133.8, 130.2, 130.1, 129.9, 129.1, 128.8, 128.5, 128.3, 127.0, 125.7, 121.7, 119.2, 114.6, 76.1, 33.7, 29.8.

HRMS: m/z for $C_{27}H_{22}Cl_2N_3O_2$ $[M+H]^+$ calcd.: 490.1084, found: 490.108.

3-(3-Chlorophenyl)-6-((4-chlorophenyl)(hydroxy)(1-methyl-1H-imidazol-5-yl)methyl)-1-methylquinolin-2(1*H*)-one (8j)

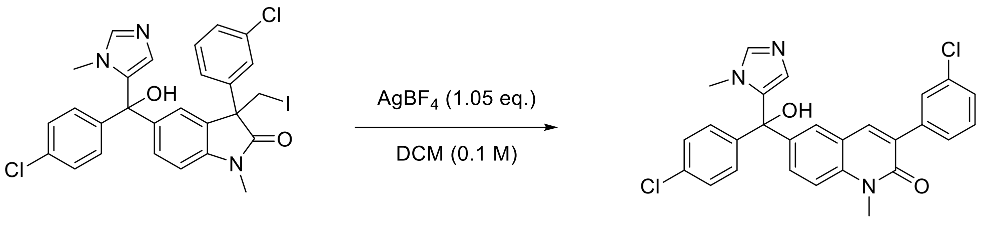

1yb **8j**

The title compound was obtained according to general procedure **B** from **1xb** (155 mg, 0.25 mmol). Purification by preparative TLC (6% MeOH in DCM) afforded **8j** as a white solid (97.4 mg, 0.20 mmol, 79%).

^{1}H-NMR (400 MHz, CDCl$_3$) δ 7.65 (s, 2H), 7.57 – 7.50 (m, 2H), 7.44 (d, *J* = 2.2 Hz, 1H), 7.38 – 7.33 (m, 2H), 7.29 – 7.23 (m, 5H), 7.21 (s, 1H), 6.25 (t, *J* = 1.2 Hz, 1H), 4.00 – 3.66 (m, 3H), 3.35 (s, 3H).

^{13}C{1H} NMR (101 MHz, CDCl$_3$) 161.3, 142.9, 140.4, 139.1, 139.0, 138.2, 137.4, 135.8, 134.2, 133.8, 131.5, 130.7, 129.8, 129.6, 129.0, 128.5, 128.4, 128.3, 127.2, 127.0, 120.0, 114.1, 76.1, 33.6, 30.3.

HRMS: m/z for $C_{27}H_{22}Cl_2N_3O_2$ $[M+H]^+$ calcd.: 490.1084, found: 490.1075.

Mechanistic experiments

1-(2-(Prop-1-en-2-yl)phenyl)urea (6)

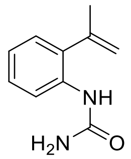

A flame-dried 4 mL drum vial in a glovebox was charged with 3-(iodomethyl)-3-methylindolin-2-one (71.8 mg, 0.25 mmol, 1.00 eq.) and LiHMDS (62.7mg, 375 µmol, 1.50 eq.) and a magnetic stir bar. The vial was sealed with a septum-cap and removed from the glovebox. Then, THF (0.1 M) was added through the septum. The resulting mixture was placed on a heated stirring plate at 80 °C. The mixture was then stirred for 16 hours. Upon completion, MeOH and water were added, and the aqueous phase extracted with EtOAc (3 × 10 mL). The combined organic phases were dried over $MgSO_4$ and volatiles were removed *in vacuo*. The title compound was obtained upon purification by preparative TLC (100% EtOAc) as white crystals (12.6 mg, 71.5 µmol, 29%).

^{1}H-NMR (400 MHz, DMSO-d^6) δ 7.86 (dd, *J* = 8.3, 1.2 Hz, 1H), 7.47 (s, 1H), 7.15 (ddd, *J* = 8.5, 7.3, 1.7 Hz, 1H), 7.06 (dd, *J* = 7.6, 1.7 Hz, 1H), 6.93 (td, *J* = 7.5, 1.3 Hz, 1H), 6.12 (s, 2H), 5.28 (dt, *J* = 3.1, 1.5 Hz, 1H), 4.94 (dq, *J* = 1.9, 0.9 Hz, 1H), 2.00 (dd, *J* = 1.6, 0.9 Hz, 3H).

^{13}C{1H} NMR (101 MHz, DMSO-d^6) δ 156.1, 142.9, 136.2, 133.5, 127.9, 127.2, 121.8, 121.3, 116.7, 23.6.

The spectroscopic data matched those reported in the literature.[36]

Crystallographic data is provided below.

***N*-methyl-*N*-(2-(1-phenylvinyl)phenyl)morpholine-4-carboxamide (7)**

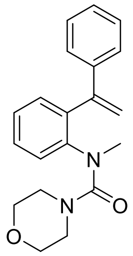

A flame-dried 4 mL drum vial in a glovebox was charged with 3-(chloromethyl)-1-methyl-3-phenylindolin-2-one (67.9 mg, 0.25 mmol, 1.00 eq.) and LiHMDS (62.7mg, 375 µmol, 1.50 eq.) and a magnetic stir bar. The vial was sealed with a septum-cap and removed from the glovebox. Then, THF (0.1 M) was added through the septum. The resulting mixture was placed on a heated stirring plate at 80 °C. Subsequently, morpholine (21.6 µL, 0.25 mmol, 1.00 eq.) was added dropwise to the reaction mixture over 10 minutes. The mixture was then stirred for 16 hours. Upon completion, water was added, and the aqueous phase extracted with DCM (3 × 10 mL). The combined organic phases were dried over $MgSO_4$ and volatiles were removed *in vacuo*. The title compound was obtained upon purification by preparative TLC (100% EtOAc) as a white solid (14.5 mg, 45.0 µmol, 18%).

^{1}H-NMR (400 MHz, $CDCl_3$) δ 7.40 – 7.27 (m, 5H), 7.23 – 7.12 (m, 4H), 5.64 (s, 1H), 5.30 (s, 1H), 3.36 (t, *J* = 4.8 Hz, 4H), 2.93 (t, *J* = 4.9 Hz, 4H), 2.90 (s, 3H).

^{13}C{1H} NMR (101 MHz, $CDCl_3$) δ 160.7, 148.0, 144.1, 140.7, 138.3, 132.3, 129.2, 128.3, 128.1, 127.3, 127.0, 126.2, 116.6, 66.5, 45.9, 39.6.

HRMS: m/z [M+H]$^+$ calcd. for: $C_{20}H_{23}N_2O_2$ 323.1754, found: 323.175.

KIE

3-(iodomethyl-d2)-1,3-diphenylindolin-2-one (1mb-d2)

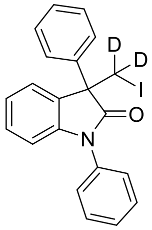

The title compound was synthesized analogous to **1mb**.

^{1}H-NMR (400 MHz, $CDCl_3$) δ 7.59 – 7.51 (m, 4H), 7.51 – 7.42 (m, 4H), 7.40 – 7.33 (m, 4H), 7.25 (td, *J* = 7.5, 1.1 Hz, 1H), 6.92 (dt, *J* = 8.0, 0.8 Hz, 1H).

^{13}C{1H} NMR (101 MHz, $CDCl_3$) δ 175.9, 144.4, 137.8, 134.5, 130.8, 129.8, 129.1, 129.0, 128.5, 128.3, 127.3, 127.0, 125.3, 123.4, 110.1, 56.6.

HRMS: m/z $[M+H]^+$ calcd. for: $C_{21}H_{15}D_2INO$ 428.0475, found: 428.0481.

Determination of KIE through competition experiments

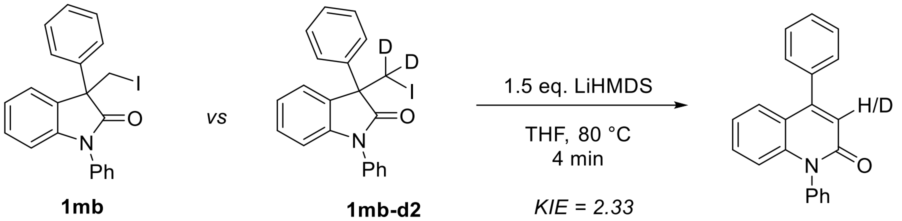

1mb **1mb-d2** *KIE = 2.33*

Three flame-dried 4 mL drum vial in a glovebox were charged with **1mb** (21.3 mg, 0.05 mmol, 0.5 eq.) and **1mb-d2** (21.4 mg, 0.05 mmol, 0.5 eq.) and a magnetic stir bar. To each of the vials was added 0.5 mL of a stock solution of THF-d^8 (2 mL), 1,3,5-trimethoxybenzene (22.2 mg, 0.13 mmol, 1.33 eq.) and LiHMDS (25.1 mg, 0.20 mmol, 6.00 eq.). The vials were sealed, removed from the glovebox and subsequently cooled to 0 °C in an ice-bath to ensure identical starting conditions. They were subsequently placed into a preheated heating block at 80 °C on a stir plate and allowed to stir for 4 min, before they were removed and immediately cooled in an ice-bath to 0 °C. Water was added and the aqueous phase extracted with EtOAc (3 × 1 mL). Upon removal of volatiles *in vacuo*, the samples were taken up in THF-d^8 and the yields determined by NMR.

To identify the relative abundance of deuterium in the sample, the internal standard was defined as 100 for all three reactions. The peak at 6.73 ppm was identified to be a common signal for both the deuterated and the non-deuterated version of the starting material, whereas the singlet at 6.60 ppm only occurs in the product of the non-deuterated substrate. Therefore, the amount of deuterated product could be calculated through:

$$amount\ (D - product) = amount\ (common\ peak) - amount\ (H - product)$$

	run 1	run 2	run 3
Ref integral	100	100	100
Common peak integral:	5.84	6.66	6.04
H-integral	4.1	4.66	4.21
D -amount	1.74	2	1.83
k_H/k_D:	2.356	2.330	2.301
Average KIE:	2.329		

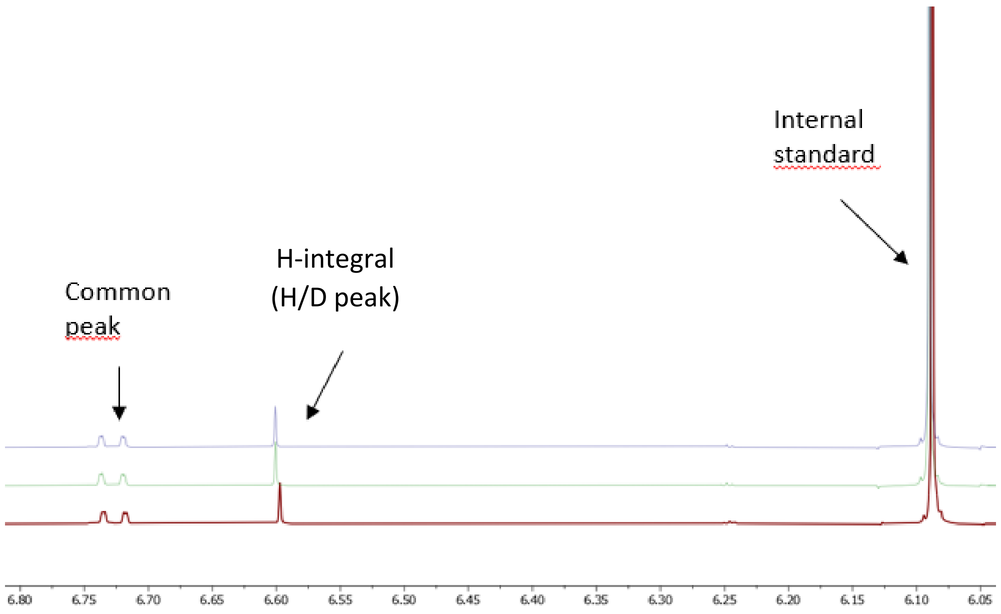

Figure S1: Rates of the LiHMDS mediated ring expansion of oxindoles with varying leaving groups.

Determination of KIE through initial rates

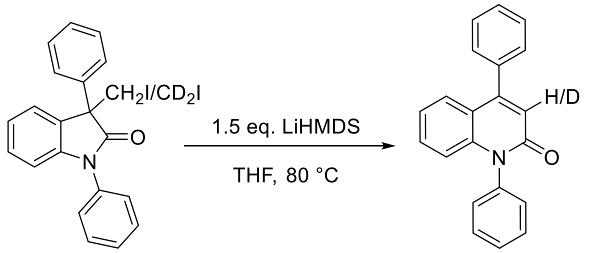

A flame-dried 4 mL drum vial in a glovebox was charged with a stir bar and 0.5 mL of a stock solution of THF-d^8 (5 mL), the corresponding starting material (0.50 mmol, 1.00 eq.), LiHMDS (125 mg, 0.75 mmol, 1.50 eq.) and 1,3,5-trimethoxybenzene (27.8 mg, 0.165 mmol, 0.33 eq.) The vials were sealed, removed from the glovebox and subsequently cooled to 0 °C in an ice-bath to ensure identical starting conditions. They were subsequently placed into a preheated heating block at 80 °C on a stir plate and allowed to stir for 2.5, 5 and 10 min, before they were removed and immediately cooled in an ice-bath to 0 °C. A drop of water was added to quench the remaining

LiHMDS and samples were transferred through a plug of $MgSO_4$ into a NMR tube to determine the corresponding yields.

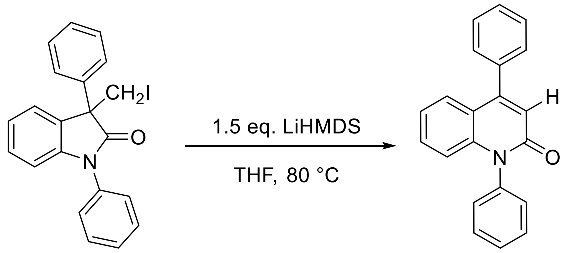

time [s]	*int1*	*int2*	*int 3*	*Conc. 1*	*Conc. 2*	*Conc. 3*	*mean*
150	6.27	5.76	5.65	0.00627	0.00576	0.00565	0.0058933
300	11.98	11.64	12.36	0.01198	0.01164	0.01236	0.0119933
600	23.21	23.22	22.88	0.02321	0.02322	0.02288	0.0231033

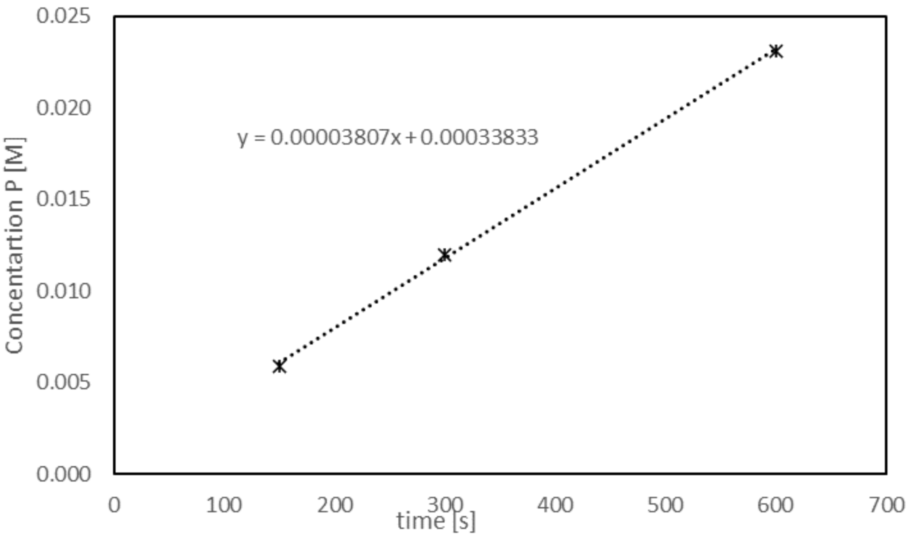

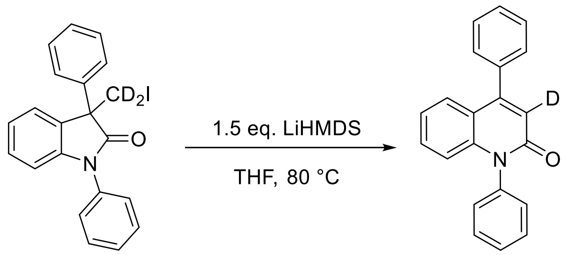

time [s]	*int1*	*int2*	*int 3*	*Conc. 1*	*Conc. 2*	*Conc. 3*	*mean*
150	1.37	1.73	1.62	0.00137	0.00173	0.00162	0.0015733
300	3.93	3.95	3.57	0.00393	0.00395	0.00357	0.0038167
600	8.18	8.1	7.95	0.00818	0.0081	0.00795	0.0080767

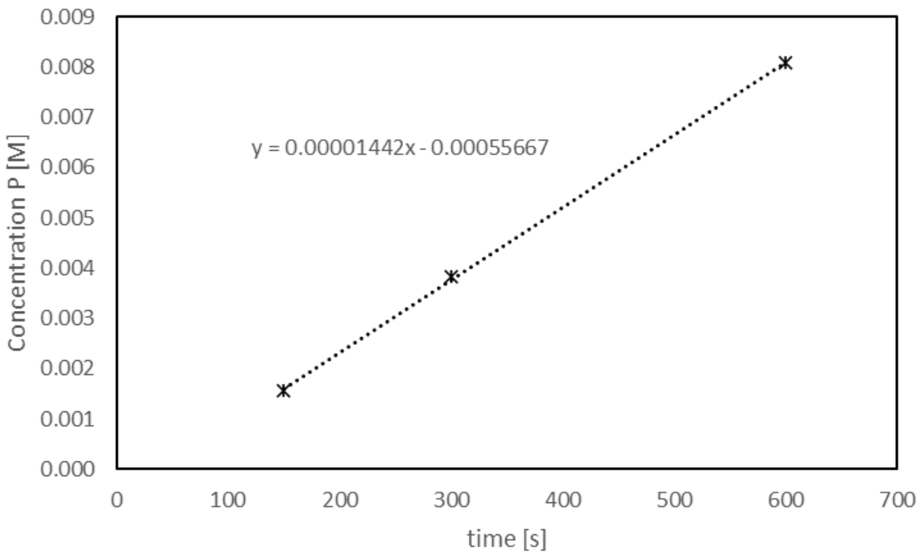

To calculate the KIE from the initial rates experiment the inclination of the linear graphs was used as an approximation to the corresponding rates.

$$KIE = \frac{inclination\,(H)}{inclination\;(D)}$$

$$KIE = 2.640$$

Hammett analysis

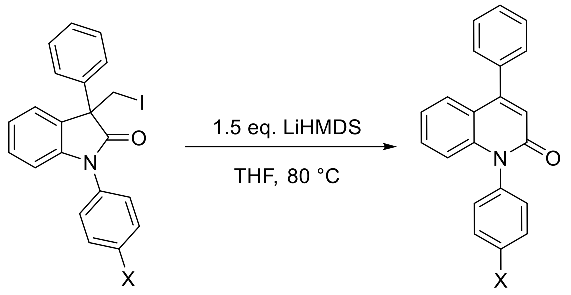

A flame-dried 4 mL drum vial in a glovebox was charged with a stir bar and 0.5 mL of a stock solution of THF-*d*[8] (3 mL), the corresponding starting material (0.30 mmol, 1.00 eq.), LiHMDS (75.0 mg, 0.45 mmol, 1.50 eq.) and 1,3,5-trimethoxybenzene (16.7 mg, 0.10 mmol, 0.33 eq.) The vials were sealed, removed from the glovebox and subsequently cooled to 0 °C in an ice-bath to ensure identical starting conditions. They were subsequently placed into a preheated heating block at 80 °C on a stir plate and allowed to stir for 2.5, 5 and 10 min, before they were removed and immediately cooled in an ice-bath to 0 °C. A drop of water was added to quench the remaining LiHMDS and samples were transferred through a plug of $MgSO_4$ into a NMR tube to determine the corresponding yields.

X =	σ	log(kR/kH)
**H**	0	1
NMe_2	-0.83	-0.1611372
OMe	-0.268	-0.1044341
CF_3	0.54	-0.0446013

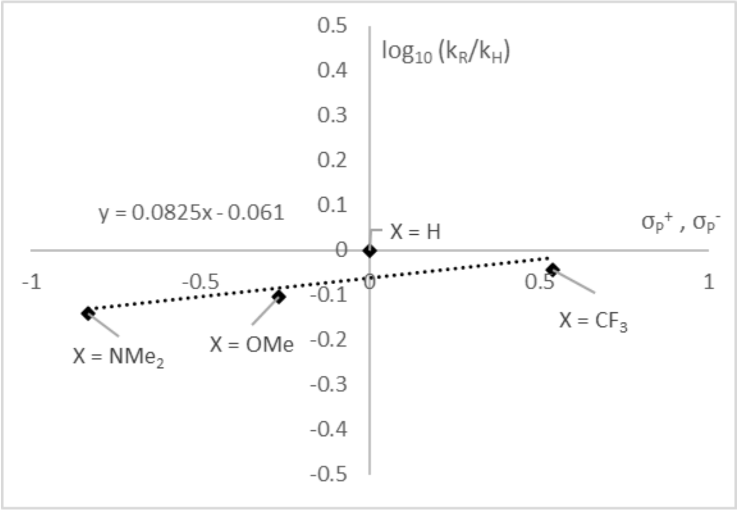

Figure S2: Hammett plot.

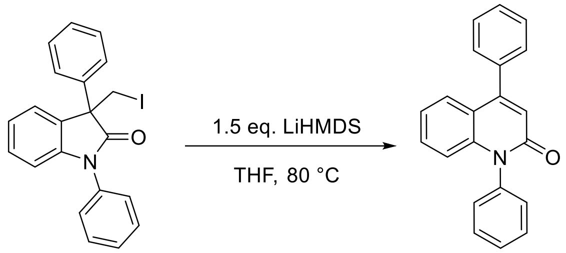

time [s]	*integral run 1*	*integral run 2*	*Conc. 1*	*Conc. 2*	*mean*
150	3.35	4.72	0.00335	0.00472	0.0040350
300	10.01	10.39	0.01001	0.01039	0.0102000
600	20.58	20.77	0.02058	0.02077	0.0206750

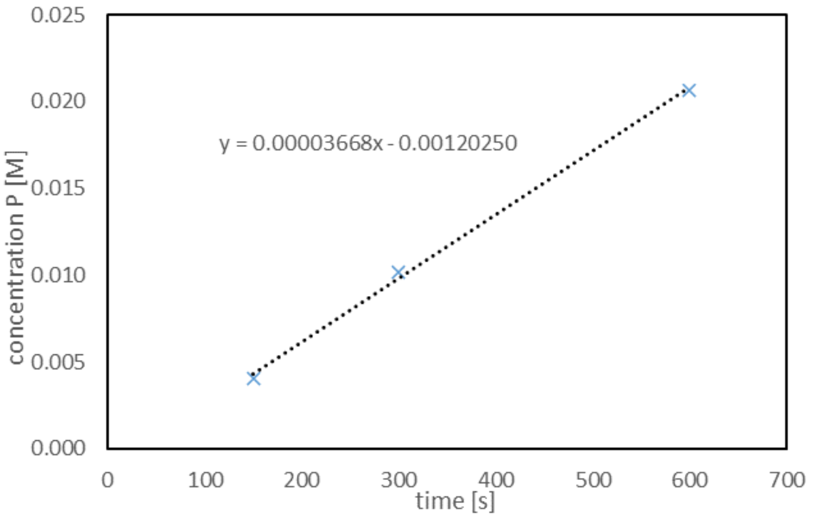

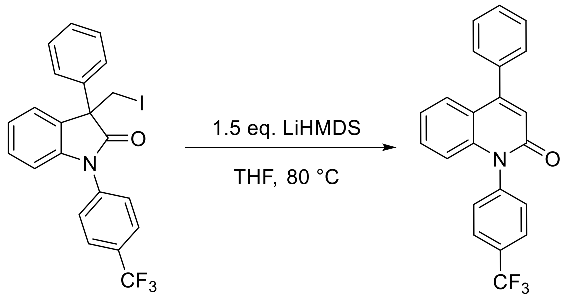

time [s]	*integral run 1*	*integral run 2*	*Conc. 1*	*Conc. 2*	*mean*
150	6.59	6.62	0.00659	0.00662	0.0066050
300	11.88	11.17	0.01188	0.01117	0.0115250
600	21.66	21.32	0.02166	0.02132	0.0214900

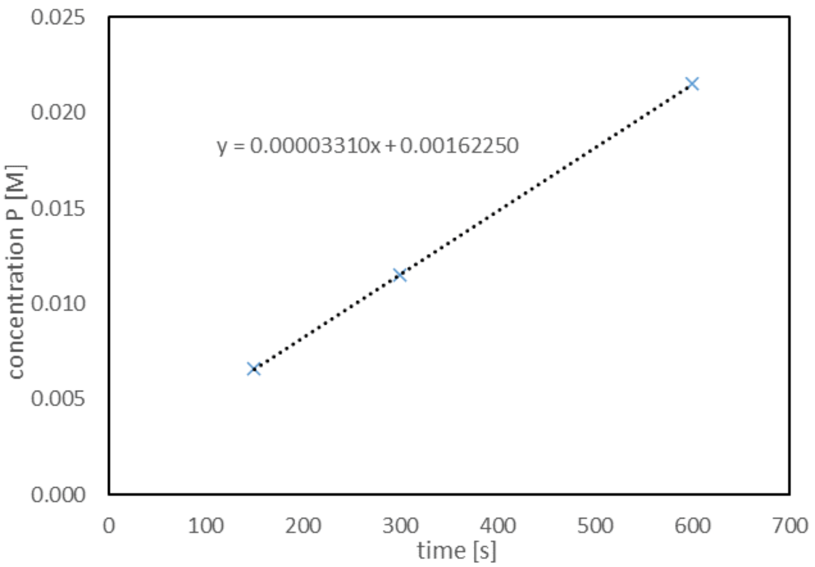

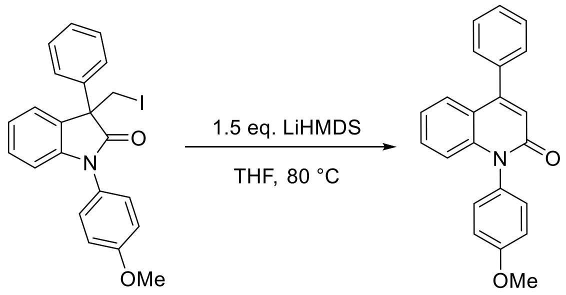

time [s]	*integral run 1*	*integral run 2*	*Conc. 1*	*Conc. 2*	*mean*
150	7.02	6.82	0.00702	0.00682	0.0069200
300	11.88	11.02	0.01188	0.01102	0.0114500
600	20.3	19.58	0.0203	0.01958	0.0199400

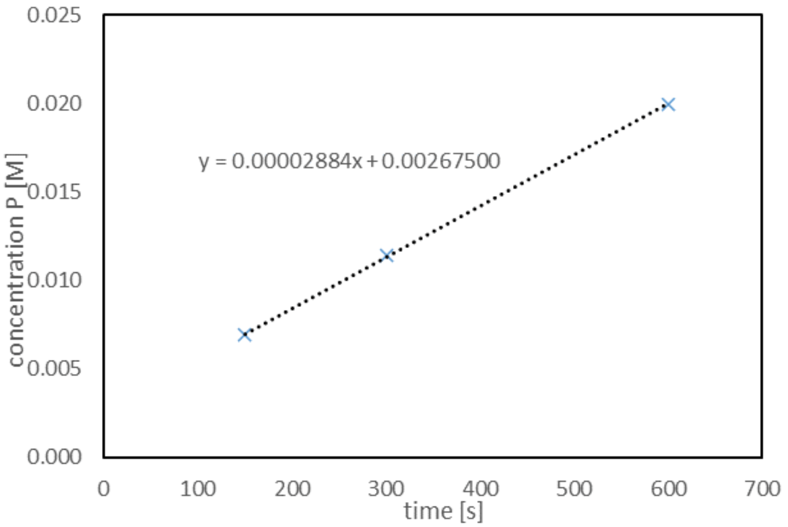

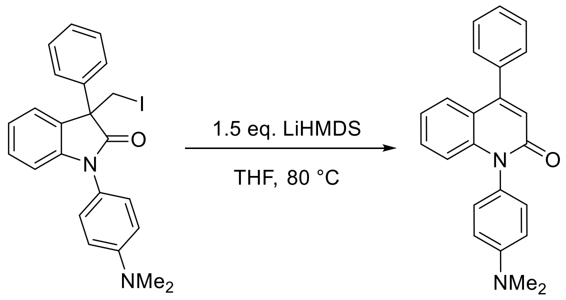

time [s]	*integral run 1*	*integral run 2*	*Conc. 1*	*Conc. 2*	*mean*
150	4.38	4.92	0.00438	0.00492	0.0046500
300	9.12	8.61	0.00912	0.00861	0.0088650
600	16.97	16.29	0.01697	0.01629	0.0166300

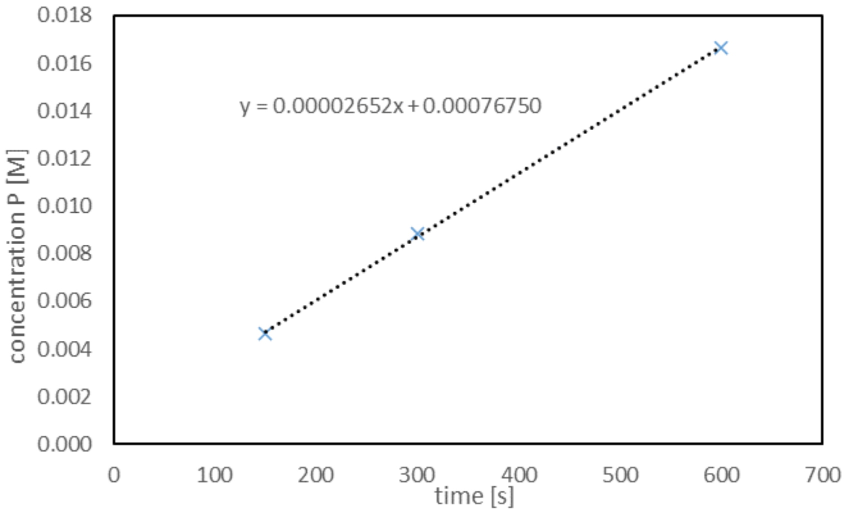

Reaction profiles

Procedure:

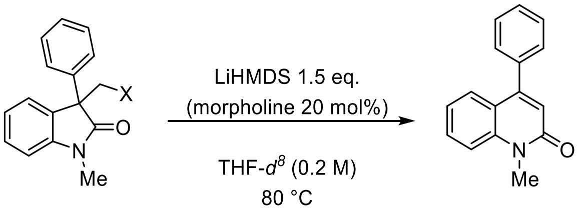

A flame-dried 4 mL drum vial in a glovebox was charged with oxindole (0.15 mmol, 1.00 eq.), LiHMDS (37.6 mg, 225 µmol, 1.50 eq.), trimethoxybenzene (8.33 mg, 49.5 µmol, 0.33 eq.), THF-d^8 (0.75 mL) and a magnetic stir bar. Where required, morpholine (2.59 µL, 30.0 µmol, 0.20 eq.) was added. The mixture was stirred at room temperature until homogeneous and then transferred into a flame-dried NMR tube. The tube was sealed with a rubber septum, removed from the glovebox and inserted into an NMR spectrometer preheated to 80 °C. At defined time intervals, a ^{1}H NMR spectrum of the reaction mixture was measured. The concentration was determined by integration of the product relative to the internal standard.

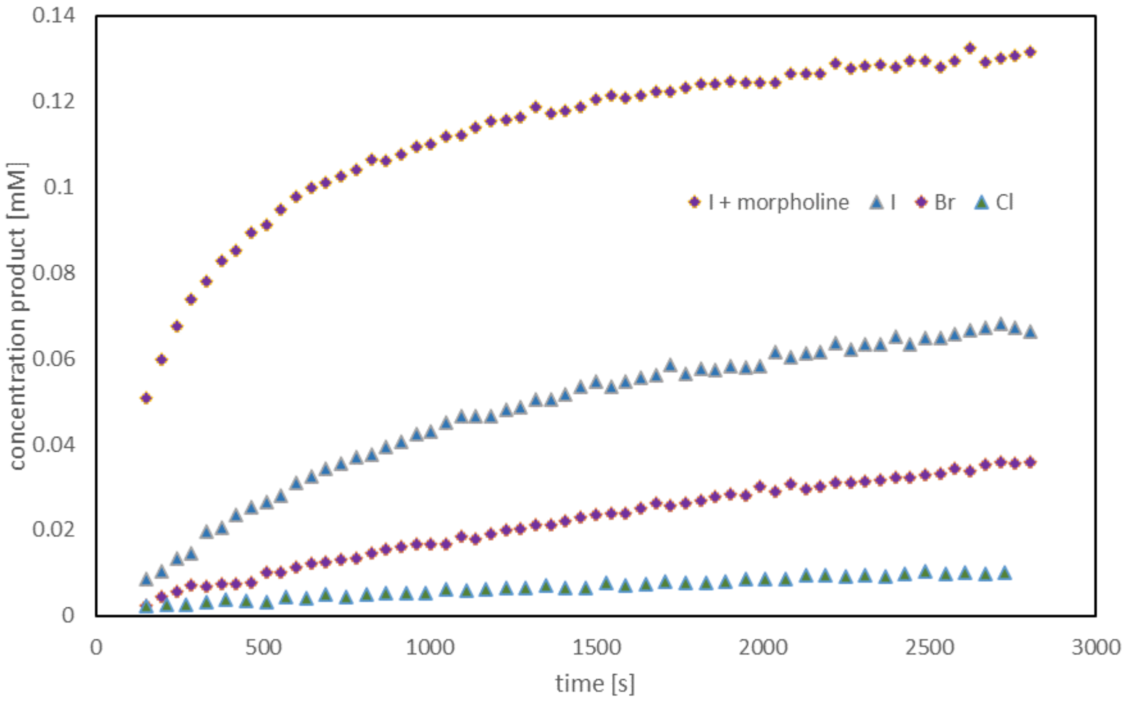

Figure S3: Rates of the LiHMDS mediated ring expansion of oxindoles with varying leaving groups.

The addition of morpholine led to a drastic increase in the reaction rate for the iodide derivative. Comparison with other amines on the reaction can be found in table **S3**. Preceding reports have indicated comparable effects on reactivity with regards to the introduction of amines into LiHMDS-carbonyl complexes.[37-39]

X-ray data

General information

Single crystalline samples were measured on the following instrument:

- Rigaku Oxford Diffraction XtaLAB Synergy-S Dualflex kappa diffractometer equipped with a Dectris Pilatus 300 HPAD detector and using microfocus sealed tube Cu-Kα radiation with mirror optics (λ = 1.54178 Å).

All measurements were carried out at 100 K using an Oxford Cryosystems Cryostream 800 sample cryostat. Data collected on the Bruker instrument were integrated using SAINT from the Bruker APEX3 program suite and corrected for absorption effects using the multi-scan method (SADABS).[40] Data collected on the Rigaku instrument were integrated using CrysAlisPro and corrected for absorption effects using a combination of empirical (ABSPACK) and numerical corrections.[41] The structures were solved using SHELXS[42] or SHELXT[43] and refined by full-matrix least-squares analysis (SHELXL)[43,44] using the program package OLEX2.[45] Unless otherwise indicated below, all non-hydrogen atoms were refined anisotropically and hydrogen atoms were constrained to ideal geometries and refined with fixed isotropic displacement parameters (in terms of a riding model). These data can be obtained free of charge from The Cambridge Crystallographic Data Centre, 12 Union Road, Cambridge CB2 1EZ, UK (fax: +44(1223)-336-033; e-mail: deposit@ccdc.cam.ac.uk), or via https://www.ccdc.cam.ac.uk/structures..

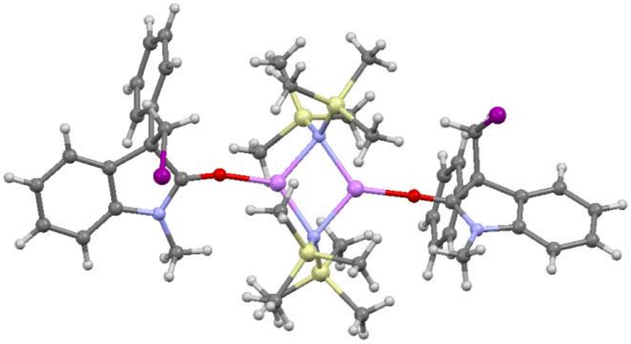

CCDC number	2301771
Empirical formula	$C_{44}H_{64}I_2Li_2N_4O_2Si_4$
Formula weight	1061.03
Temperature [K]	100.0(1)
Crystal system	triclinic
Space group (number)	$P\overline{1}$ (2)
a [Å]	9.2441(2)
b [Å]	14.7153(3)
c [Å]	19.0057(2)
α [°]	86.491(2)
β [°]	89.0120(10)
γ [°]	80.156(2)
Volume [Å^3]	2542.44(8)
Z	2
ρ_{calc} [gcm^{-3}]	1.386
μ [mm^{-1}]	10.902
F(000)	1080
Crystal size [mm^3]	0.19×0.162×0.032
Crystal colour	clear colourless
Crystal shape	plate
Radiation	Cu K_α (λ=1.54184 Å)
2θ range [°]	4.66 to 159.77 (0.78 Å)
Index ranges	−11 ≤ h ≤ 11<br>−15 ≤ k ≤ 18<br>−24 ≤ l ≤ 24
Reflections collected	45433
Independent reflections	10766<br>R_{int} = 0.0802<br>R_{sigma} = 0.0553
Completeness to θ = 67.684°	99.9 %

Data / Restraints / Parameters	10766/354/595
Goodness-of-fit on F^2	1.095
Final R indexes [I≥2σ(I)]	R1 = 0.0494 wR2 = 0.1292
Final R indexes [all data]	R1 = 0.0579 wR2 = 0.1345
Largest peak/hole [eÅ−3]	1.66/-1.39

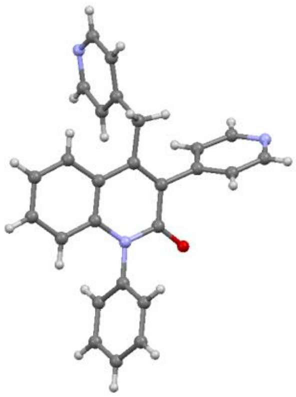

CCDC number	2301770
Empirical formula	$C_{26}H_{19}N_3O$
Formula weight	389.44
Temperature [K]	100.0(1)
Crystal system	monoclinic
Space group (number)	$P2_1/c$ (14)
a [Å]	9.84900(10)
b [Å]	19.0323(2)
c [Å]	11.36930(10)
α [°]	90
β [°]	110.8640(10)
γ [°]	90
Volume [Å^3]	1991.42(4)
Z	4
ρ_{calc} [gcm^{-3}]	1.299
μ [mm^{-1}]	0.637
F(000)	816
Crystal size [mm^3]	0.147×0.091×0.041
Crystal colour	clear yellow
Crystal shape	plate
Radiation	Cu K_α (λ=1.54184 Å)
2θ range [°]	9.29 to 159.07 (0.78 Å)
Index ranges	−11 ≤ h ≤ 12<br>−23 ≤ k ≤ 23<br>−14 ≤ l ≤ 12
Reflections collected	64860
Independent reflections	4171<br>R_{int} = 0.0392<br>R_{sigma} = 0.0157
Completeness to	100.0 %

θ = 67.684°	
Data / Restraints / Parameters	4171/0/271
Goodness-of-fit on F^2	1.042
Final *R* indexes [$I \geq 2\sigma(I)$]	R_1 = 0.0385<br>wR_2 = 0.1023
Final *R* indexes [all data]	R_1 = 0.0430<br>wR_2 = 0.1059
Largest peak/hole [eÅ^{-3}]	0.27/-0.22

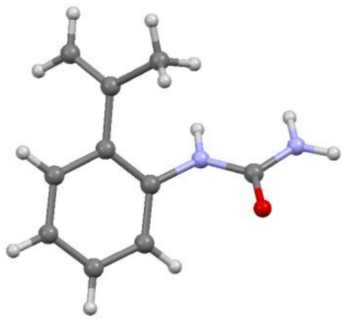

CCDC number	2301769
Empirical formula	$C_{10}H_{12}N_2O$
Formula weight	176.22
Temperature [K]	100.0(1)
Crystal system	monoclinic
Space group (number)	$P2_1/c$ (14)
a [Å]	14.8794(6)
b [Å]	7.7222(3)
c [Å]	8.2727(3)
α [°]	90
β [°]	104.667(4)
γ [°]	90
Volume [Å^3]	919.57(6)
Z	4
ρ_{calc} [gcm^{-3}]	1.273
μ [mm^{-1}]	0.677
F(000)	376
Crystal size [mm^3]	0.156×0.083×0.016
Crystal colour	clear colourless
Crystal shape	plate
Radiation	Cu K_α (λ=1.54184 Å)
2θ range [°]	12.30 to 158.20 (0.79 Å)
Index ranges	−17 ≤ h ≤ 18<br>−9 ≤ k ≤ 9<br>−10 ≤ l ≤ 6
Reflections collected	4405
Independent reflections	1714<br>R_{int} = 0.0356<br>R_{sigma} = 0.0436
Completeness to θ = 67.684°	96.7 %
Data / Restraints / Parameters	1714/4/128

Goodness-of-fit on F^2	1.046
Final R indexes [I≥2σ(I)]	R1 = 0.0415<br>wR2 = 0.1026
Final R indexes [all data]	R1 = 0.0557<br>wR2 = 0.1117
Largest peak/hole [eÅ−3]	0.19/-0.24

References

(1) Altman, R. A.; Hyde, A. M.; Huang, X.; Buchwald, S. L. Orthogonal Pd- and Cu-Based Catalyst Systems for C- and N-Arylation of Oxindoles. *J. Am. Chem. Soc.* **2008**, *130* (29), 9613–9620. https://doi.org/10.1021/ja803179s.

(2) El Bouakher, A.; Massip, S.; Jarry, C.; Troin, Y.; Abrunhosa-Thomas, I.; Guillaumet, G. A General and Efficient Method to Access Tetracyclic Spirooxindole Derivatives. *European J. Org. Chem.* **2015**, *2015* (3), 556–559. https://doi.org/10.1002/ejoc.201403292.

(3) Yoon, H.; Marchese, A. D.; Lautens, M. Carboiodination Catalyzed by Nickel. *J. Am. Chem. Soc.* **2018**, *140* (35), 10950–10954. https://doi.org/10.1021/jacs.8b06966.

(4) Delcaillau, T.; Schmitt, H. L.; Boehm, P.; Falk, E.; Morandi, B. Palladium-Catalyzed Carbothiolation of Alkenes and Alkynes for the Synthesis of Heterocycles. *ACS Catal.* **2022**, *12* (10), 6081–6091. https://doi.org/10.1021/acscatal.2c01178.

(5) Cheng, H.-G.; Pu, M.; Kundu, G.; Schoenebeck, F. Selective Methylation of Amides, N -Heterocycles, Thiols, and Alcohols with Tetramethylammonium Fluoride. *Org. Lett.* **2020**, *22* (1), 331–334. https://doi.org/10.1021/acs.orglett.9b04400.

(6) Morales-Colón, M. T.; See, Y. Y.; Lee, S. J.; Scott, P. J. H.; Bland, D. C.; Sanford, M. S. Tetramethylammonium Fluoride Alcohol Adducts for S N Ar Fluorination. *Org. Lett.* **2021**, *23* (11), 4493–4498. https://doi.org/10.1021/acs.orglett.1c01490.

(7) Kang, Q. K.; Lin, Y.; Li, Y.; Xu, L.; Li, K.; Shi, H. Catalytic SNAr Hydroxylation and Alkoxylation of Aryl Fluorides. *Angew. Chemie - Int. Ed.* **2021**, *60* (37), 20391–20399. https://doi.org/10.1002/anie.202106440.

(8) Lian, Z.; Friis, S. D.; Skrydstrup, T. Palladium-Catalyzed Carbonylative α-Arylation of 2-Oxindoles with (Hetero)Aryl Bromides: Efficient and Complementary Approach to 3-Acyl-2-Oxindoles. *Angew. Chemie - Int. Ed.* **2014**, *53* (36), 9582–9586. https://doi.org/10.1002/anie.201404217.

(9) Luise, N.; Wyatt, P. G. Diversity-Oriented Synthesis of Bicyclic Fragments Containing Privileged Azines. *Bioorganic Med. Chem. Lett.* **2019**, *29* (2), 248–251. https://doi.org/10.1016/j.bmcl.2018.11.046.

(10) Zhang, Q. B.; Jia, W. L.; Ban, Y. L.; Zheng, Y.; Liu, Q.; Wu, L. Z. Autoxidation/Aldol Tandem Reaction of 2-Oxindoles with Ketones: A Green Approach for the Synthesis of 3-Hydroxy-2-Oxindoles. *Chem. - A Eur. J.* **2016**, *22* (8), 2595–2598. https://doi.org/10.1002/chem.201504282.

(11) Jia; L. C.; Ji-Feng L.; Wie, L.; Amanda G.; Rogoff, H. Inhibitors of kinases and cancer stem cells, and methods of preparation and use thereof. US9187454B2, 2014.

(12) Cui, B.-D.; You, Y.; Zhao, J.-Q.; Zuo, J.; Wu, Z.-J.; Xu, X.-Y.; Zhang, X.-M.; Yuan, W.-C. 3-Pyrrolyl-Oxindoles as Efficient Nucleophiles for Organocatalytic Asymmetric Synthesis of Structurally Diverse 3,3′-Disubstituted Oxindole Derivatives. *Chem. Commun.* **2015**, *51* (4), 757–760. https://doi.org/10.1039/C4CC08364D.

(13) Kende, A. S.; Hodges, J. C. Regioselective C-3 Alkylations of Oxindole Dianion. *Synth. Commun.* **1982**, *12* (1), 1–10. https://doi.org/10.1080/00397918208080058.

(14) Ghosh, S.; Chaudhuri, S.; Bisai, A. Oxidative Dimerization of 2-Oxindoles Promoted by KO t Bu-I 2 : Total Synthesis of (±)-Folicanthine. *Org. Lett.* **2015**, *17* (6), 1373–1376. https://doi.org/10.1021/acs.orglett.5b00032.

(15) Li, H.; Cheng, B.; Boonnak, N.; Padwa, A. An Approach toward the Alkaloid (±)-Mersicarpine Using a Rhodium(II) Carbenoid Cyclization–Cycloaddition Cascade of an α-Diazo Dihydroindolinone. *Tetrahedron* **2011**, *67* (51), 9829–9836. https://doi.org/10.1016/j.tet.2011.09.118.

(16) Tsukano, C.; Okuno, M.; Takemoto, Y. Palladium-Catalyzed Amidation by Chemoselective C(Sp 3)-H Activation: Concise Route to Oxindoles Using a Carbamoyl Chloride Precursor. *Angew. Chemie - Int. Ed.* **2012**, *51* (11), 2763–2766. https://doi.org/10.1002/anie.201108889.

(17) Zetschok, D.; Heieck, L.; Wennemers, H. Decarboxylative Organocatalyzed Addition Reactions of Fluoroacetate Surrogates for the Synthesis of Fluorinated Oxindoles. *Org. Lett.* **2021**, *23* (5), 1753–1757. https://doi.org/10.1021/acs.orglett.1c00172.

(18) Xu, C.; Xie, W.; Xu, J. Metal-Free and Regiospecific Synthesis of 3-Arylindoles. *Org. Biomol. Chem.* **2020**, *18* (14), 2661–2671. https://doi.org/10.1039/D0OB00317D.

(19) Luehr, G.W.;Sundaram A.; Jaishankar, P.; Payne P. W.; Druzgala P. HUE031556T2, 2011.

(20) Ferguson, J.; Zeng, F.; Alwis, N.; Alper, H. Synthesis of 2(1 H)-Quinolinones via Pd-Catalyzed Oxidative Cyclocarbonylation of 2-Vinylanilines. *Org. Lett.* **2013**, *15* (8), 1998–2001. https://doi.org/10.1021/ol4006739.

(21) Sun, B.; Mai, W.-P.; Yang, L.-R.; Mao, P.; Yuan, J.-W.; Xiao, Y.-M. A Novel and Facile Synthesis of 4-Arylquinolin-2(1H)-Ones under Metal-Free Conditions. *Chinese Chem. Lett.* **2015**, *26* (8), 977–979. https://doi.org/10.1016/j.cclet.2015.05.008.

(22) Rodríguez, J. F.; Zhang, A.; Bajohr, J.; Whyte, A.; Mirabi, B.; Lautens, M. Cycloisomerization of Carbamoyl Chlorides in Hexafluoroisopropanol: Stereoselective Synthesis of Chlorinated Methylene Oxindoles and Quinolinones. *Angew. Chemie Int. Ed.* **2021**, *60* (34), 18478–18483. https://doi.org/10.1002/anie.202103323.

(23) Moon, Y.; Jang, E.; Choi, S.; Hong, S. Visible-Light-Photocatalyzed Synthesis of Phenanthridinones and Quinolinones via Direct Oxidative C–H Amidation. *Org. Lett.* **2018**, *20* (1), 240–243. https://doi.org/10.1021/acs.orglett.7b03600.

(24) Lee, S. H.; Chi, H. M. HFIP-Empowered One-Pot Synthesis of C4-Aryl-Substituted Tetrahydroquinolines with Propargylic Chlorides and Anilines. *Org. Lett.* **2023**, *25* (7), 1083–1087. https://doi.org/10.1021/acs.orglett.2c04299.

(25) Himbert, G.; Diehl, K.; Schlindwein, H. Cycloadditionen, 16: Einfluß von Alkyl- Und Phenylgruppen in Der Allenischen Ω-Position Auf Die Intramolekulare Diels-Alder-Reaktion Bei Allencarboxamiden. *Chem. Ber.* **1989**, *122* (9), 1691–1699. https://doi.org/10.1002/cber.19891220915.

(26) Hu, T.; Lückemeier, L.; Daniliuc, C.; Glorius, F. Ru-NHC-Catalyzed Asymmetric Hydrogenation of 2-Quinolones toChiral 3,4-Dihydro-2-Quinolones *Angew. Chem. Int. Ed.* **2021**, *43*, 23193-23196. https://doi.org/10.1002/anie.202108503

(27) Yin, K.; Zhang, R. Transition-Metal-Free Direct C–H Arylation of Quinoxalin-2(1 H)-Ones with Diaryliodonium Salts at Room Temperature. *Org. Lett.* **2017**, *19* (7), 1530–1533. https://doi.org/10.1021/acs.orglett.7b00310.

(28) Liu, L.; Lu, H.; Wang, H.; Yang, C.; Zhang, X.; Zhang-Negrerie, D.; Du, Y.; Zhao, K. PhI(OCOCF 3) 2 -Mediated C–C Bond Formation Concomitant with a 1,2-Aryl Shift in a Metal-Free Synthesis of 3-Arylquinolin-2-Ones. *Org. Lett.* **2013**, *15* (12), 2906–2909. https://doi.org/10.1021/ol400743r.

(29) Yuan, J.-W.; Yang, L.-R.; Yin, Q.-Y.; Mao, P.; Qu, L.-B. KMnO4/AcOH-mediated C3-selective direct arylation of coumarins with arylboronic acids *RSC Adv.*, **2016**, *6*, 35936-35944. https://doi.org/10.1039/C6RA04787D

(30) Cao, Y.; Zhao, H.; Zhang-Negrerie, D.; Du, Y.; Zhao, K. Metal-Free Synthesis of 3-Arylquinolin-2-Ones from N ,2-Diaryl- Acrylamides via Phenyliodine(III) Bis(2,2-Dimethylpropanoate)- Mediated Direct Oxidative C−C Bond Formation. *Adv. Synth. Catal.* **2016**, *358* (22), 3610–3615. https://doi.org/10.1002/adsc.201600512.

(31) Manley, P. J.; Bilodeau, M. T. A New Synthesis of Naphthyridinones and Quinolinones: Palladium-Catalyzed

Amidation of o-Carbonyl-Substituted Aryl Halides. *Org. Lett.* **2004**, *6* (14), 2433–2435. https://doi.org/10.1021/ol049165t.

(32) Mazodze, C. M.; Petersen, W. F. Silver-Catalysed Double Decarboxylative Addition–Cyclisation–Elimination Cascade Sequence for the Synthesis of Quinolin-2-Ones. *Org. Biomol. Chem.* **2022**, *20* (17), 3469–3474. https://doi.org/10.1039/D2OB00521B.

(33) Nykaza, T. V.; Li, G.; Yang, J.; Luzung, M. R.; Radosevich, A. T. PIII/PV=O Catalyzed Cascade Synthesis of N-Functionalized Azaheterocycles. *Angew. Chemie - Int. Ed.* **2020**, *59* (11), 4505–4510. https://doi.org/10.1002/anie.201914851.

(34) Jones, P. J.; Merrick, E. C.; Batts, T. W.; Hargus, N. J.; Wang, Y.; Stables, J. P.; Bertram, E. H.; Brown, M. L.; Patel, M. K. Modulation of Sodium Channel Inactivation Gating by a Novel Lactam: Implications for Seizure Suppression in Chronic Limbic Epilepsy. *J. Pharmacol. Exp. Ther.* **2009**, *328* (1), 201–212. https://doi.org/10.1124/jpet.108.144709.

(35) Kraus, J. M.; Verlinde, C. L. M. J.; Karimi, M.; Lepesheva, G. I.; Gelb, M. H.; Buckner, F. S. Rational Modification of a Candidate Cancer Drug for Use against Chagas Disease (Journal of Medicinal Chemistry (2009) 52, (1639) DOI: 10.1021/Jm801313t). *J. Med. Chem.* **2009**, *52* (15), 4979. https://doi.org/10.1021/jm900929t.

(36) Babu, S. S.; Varma, A. A.; Gopinath, P. Photoredox Catalyzed Cascade CF3addition/Chemodivergent Annulations of: Ortho -Alkenyl Aryl Ureas. *Chem. Commun.* **2022**, *58* (12), 1990–1993. https://doi.org/10.1039/d1cc06289a.

(37) Mack, K. A.; McClory, A.; Zhang, H.; Gosselin, F.; Collum, D. B. Lithium Hexamethyldisilazide-Mediated Enolization of Highly Substituted Aryl Ketones: Structural and Mechanistic Basis of the E/Z Selectivities. J. Am. Chem. Soc. 2017, 139 (35), 12182–12189. https://doi.org/10.1021/jacs.7b05057.

(38) Zhao, P.; Collum, D. B. Lithium Hexamethyldisilazide/Triethylamine-Mediated Ketone Enolization: Remarkable Rate Accelerations Stemming from a Dimer-Based Mechanism. J. Am. Chem. Soc. 2003, 125 (14), 4008–4009. https://doi.org/10.1021/ja021284l.

(39) Godenschwager, P. F.; Collum, D. B. Lithium Hexamethyldisilazide-Mediated Enolizations: Influence of Triethylamine on E/Z Selectivities and Enolate Reactivities. J. Am. Chem. Soc. 2008, 130 (27), 8726–8732. https://doi.org/10.1021/ja800250q.

(40) Sheldrick, G. M. SADABS. Program for Empirical Absorption Correction of Area Detector Data. Univ. of Göttingen: Göttingen, Germany, 1996.

(41) CrysAlisPro and ABSPACK. Rigaku Oxford Diffraction, 2016.

(42) Sheldrick, G. M. A short history of SHELX, Acta Cryst. 2008, A64, 112–122. https://doi.org/10.1107/S0108767307043930.

(43) Sheldrick, G. M. SHELXT - Integrated space-group and crystal-structure determination, Acta Cryst. 2015, A71, 3–8. https://doi.org/10.1107/S2053273314026370.

(44) Sheldrick, G. M. Crystal structure refinement with SHELXL, Acta Cryst. 2015, C71, 3–8. https://doi.org/10.1107/S2053229614024218.

(45) Dolomanov, O. V.; Bourhis, L. J.; Gildea, R. J.; Howard, J. A. K.; Puschmann, H. OLEX2: a complete structure solution, refinement and analysis program, J. Appl. Cryst. 2009, 42, 339–341. https://doi.org/10.1107/S0021889808042726

NMR Spectra

Starting materials

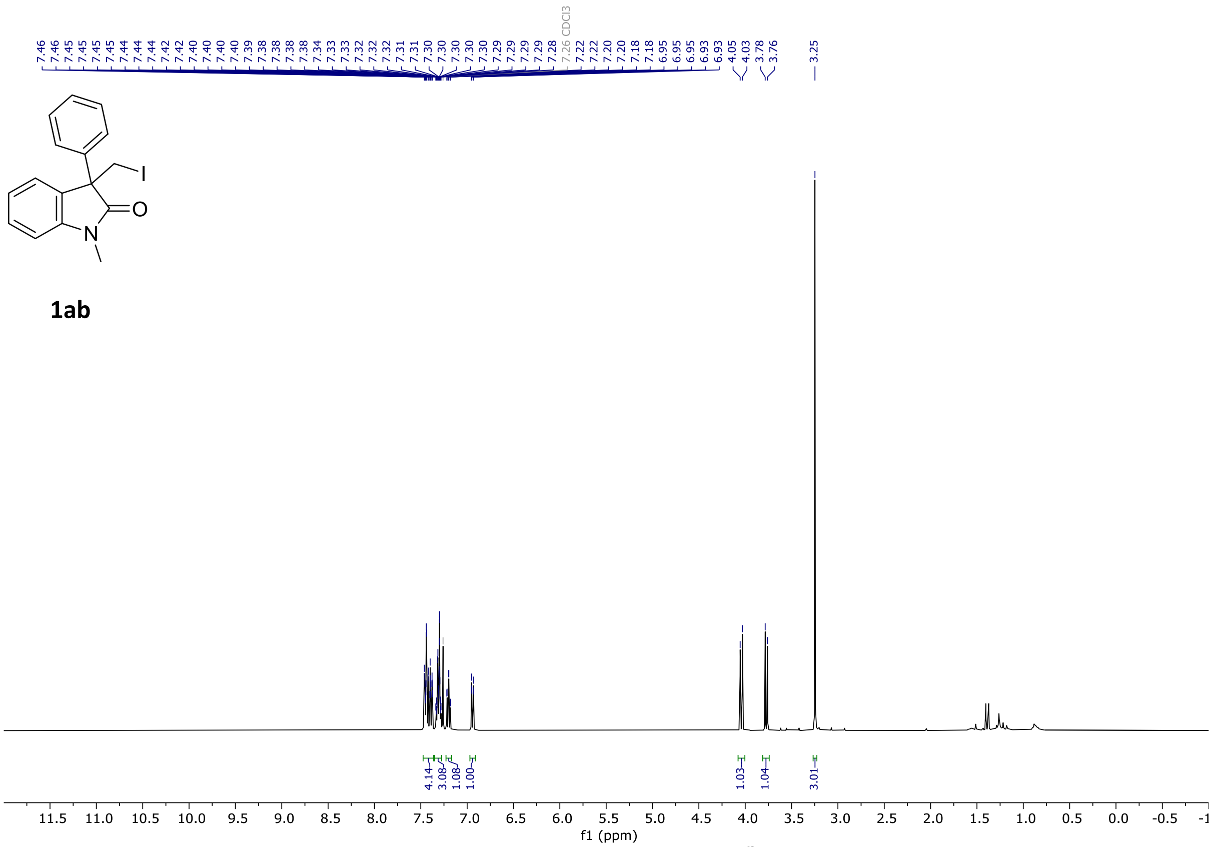

7.46 7.46 7.45 7.45 7.45 7.45 7.44 7.44 7.44 7.42 7.42 7.40 7.40 7.40 7.40 7.39 7.38 7.38 7.38 7.38 7.34 7.33 7.33 7.32 7.32 7.32 7.31 7.31 7.30 7.30 7.30 7.30 7.30 7.29 7.29 7.29 7.29 7.28
7.26 CDCl3
7.22 7.22 7.20 7.20 7.18 6.95 6.95 6.95 6.93 4.05 4.03 3.78 3.76
3.25
I
O
N
1ab
4.14
3.08
1.08
1.00
1.03
1.04
3.01
11.5 11.0 10.5 10.0 9.5 9.0 8.5 8.0 7.5 7.0 6.5 6.0 5.5 5.0 4.5 4.0 3.5 3.0 2.5 2.0 1.5 1.0 0.5 0.0 -0.5
f1 (ppm)

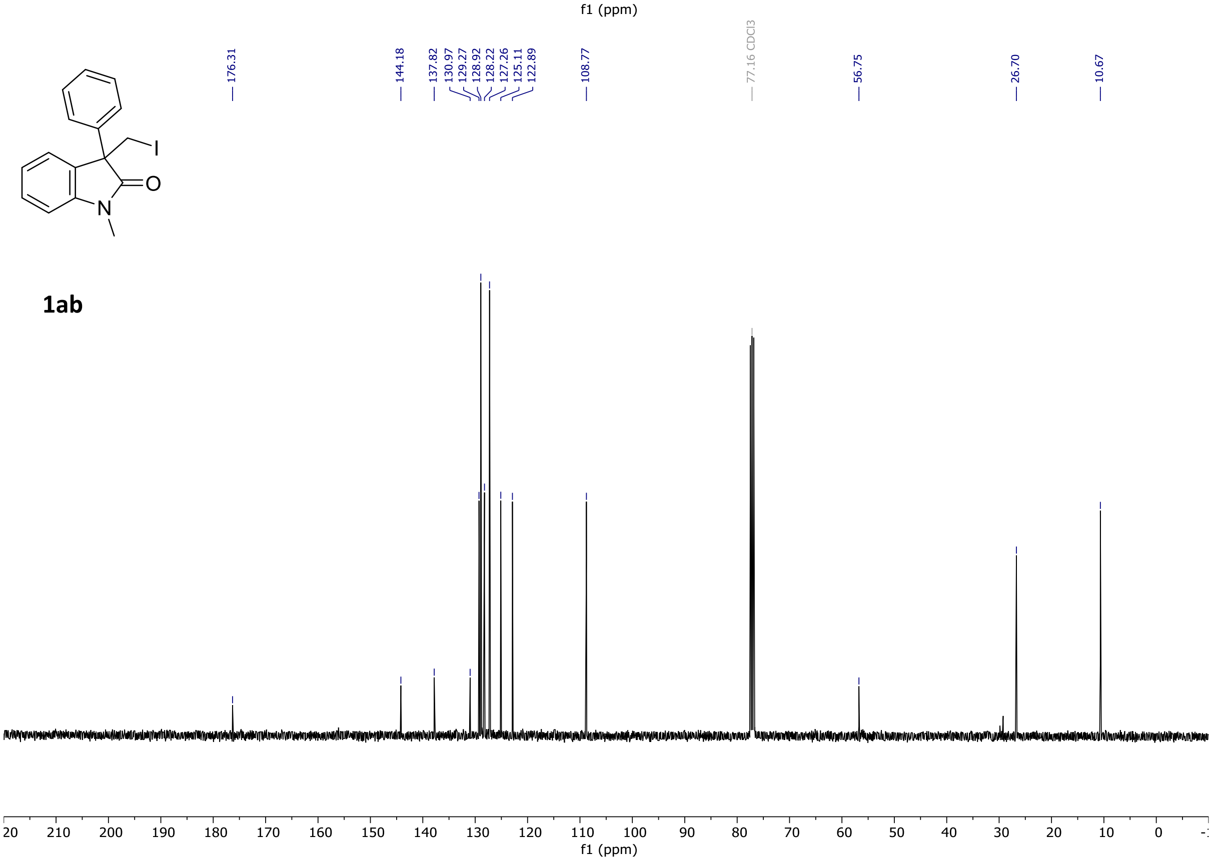

176.31
144.18
137.82 130.97 129.27 128.92 128.22 127.26 125.11 122.89
108.77
77.16 CDCl3
56.75
26.70
10.67
I
O
N
1ab
210 200 190 180 170 160 150 140 130 120 110 100 90 80 70 60 50 40 30 20 10 0
f1 (ppm)

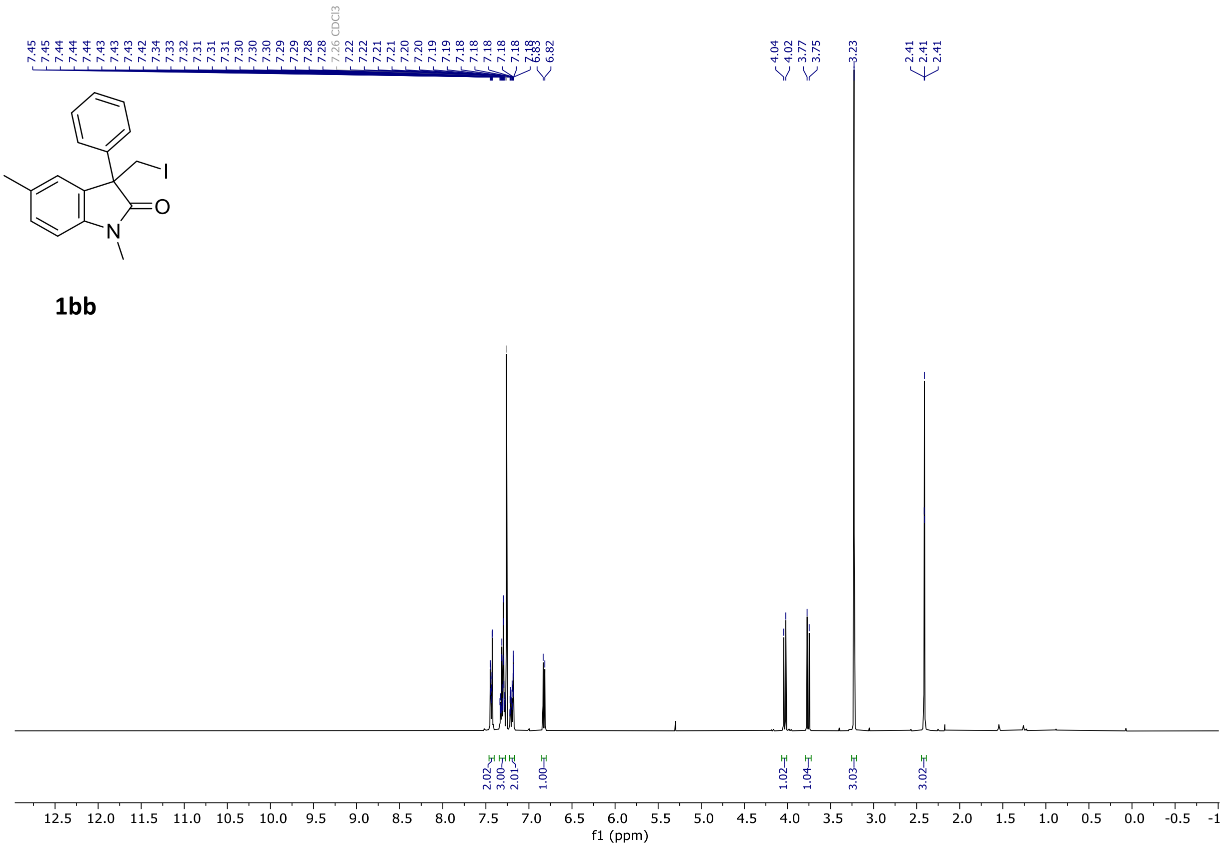
7.45 7.45 7.44 7.44 7.43 7.43 7.43 7.42 7.34 7.33 7.32 7.31 7.31 7.30 7.30 7.29 7.29 7.28 7.28 7.26 CDCl3 7.22 7.22 7.21 7.21 7.20 7.20 7.19 7.19 7.18 7.18 7.18 7.18 7.18 6.83 6.82
4.04 4.02 3.77 3.75
3.23
2.41 2.41 2.41
I
O
N
1bb
2.02 3.00 2.01 1.00 1.02 1.04 3.03 3.02
f1 (ppm)

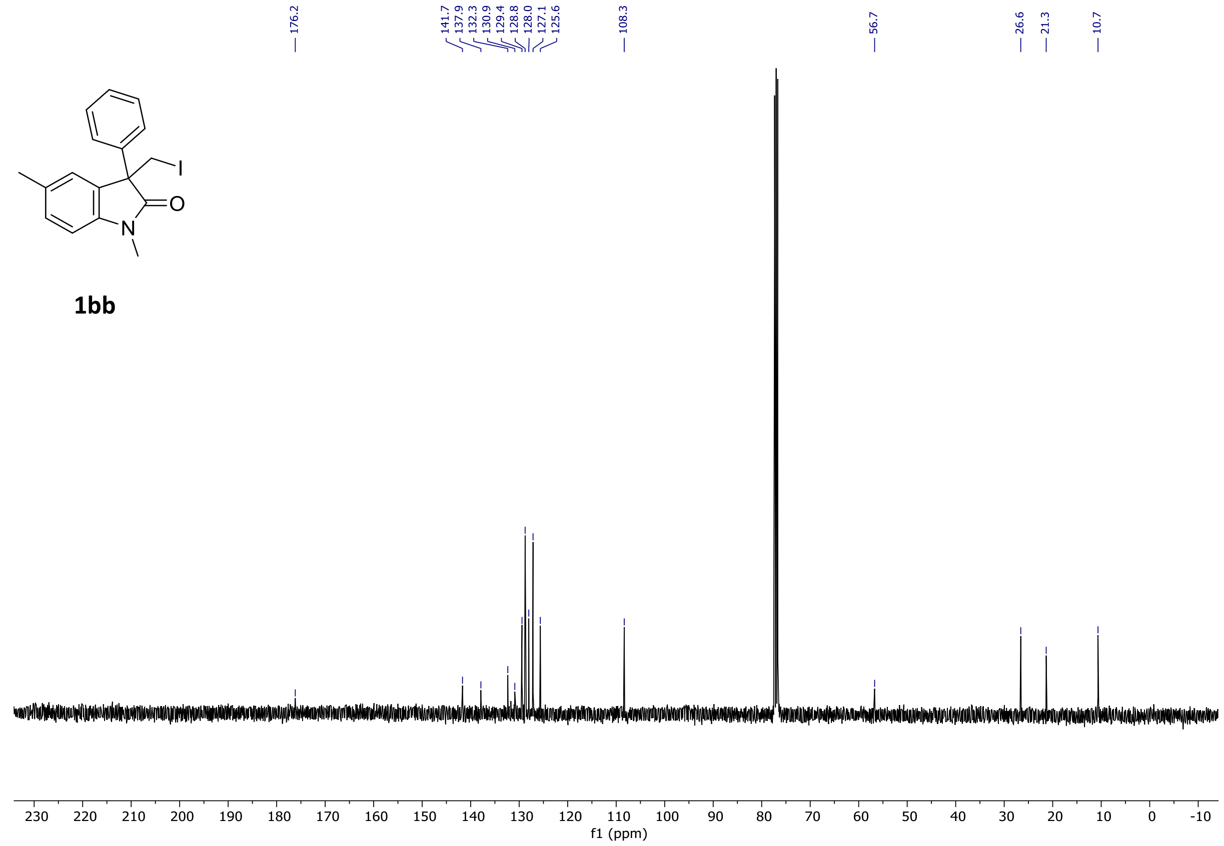
176.2
141.7 137.9 132.3 130.9 129.4 128.8 128.0 127.1 125.6
108.3
56.7
26.6 21.3 10.7
I
O
N
1bb
f1 (ppm)

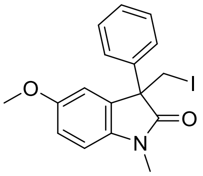
O
I
O
N

1cb

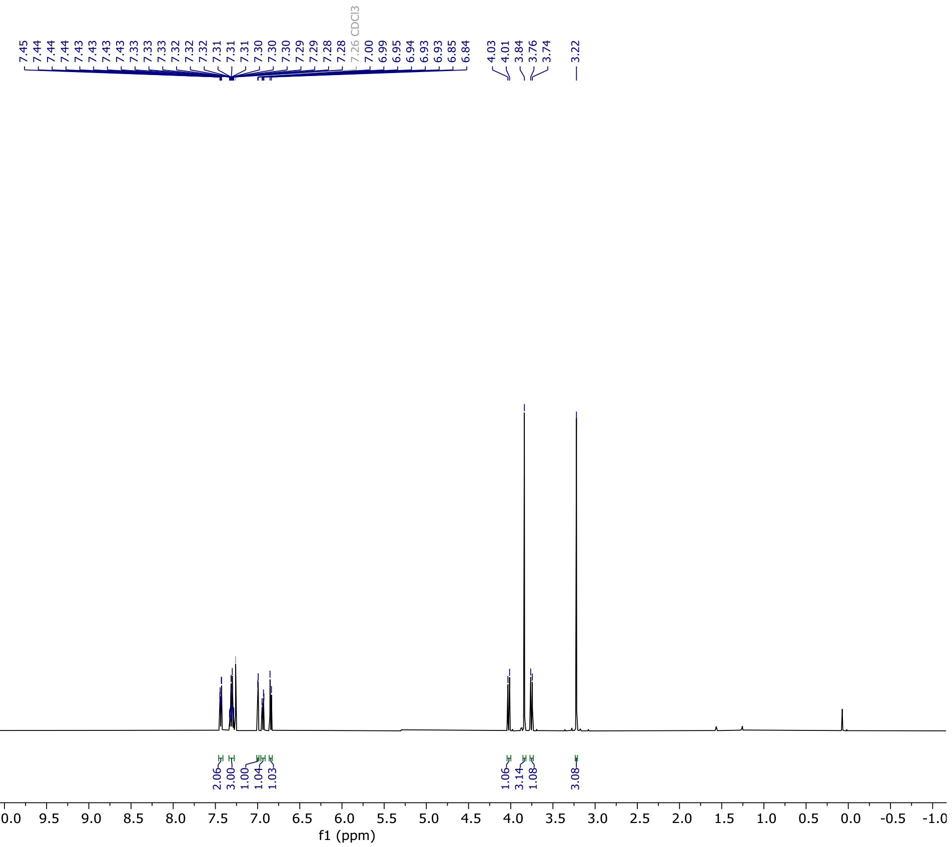
7.45 7.44 7.44 7.43 7.43 7.43 7.43 7.33 7.33 7.33 7.32 7.32 7.31 7.31 7.31 7.30 7.30 7.30 7.29 7.29 7.28 7.28 7.26 CDCl3 7.00 6.99 6.95 6.94 6.93 6.93 6.85 6.84
4.03 4.01 3.84 3.76 3.74
3.22
2.06 3.00 1.00 1.04 1.03
1.06 3.14 1.08
3.08
13.0 12.5 12.0 11.5 11.0 10.5 10.0 9.5 9.0 8.5 8.0 7.5 7.0 6.5 6.0 5.5 5.0 4.5 4.0 3.5 3.0 2.5 2.0 1.5 1.0 0.5 0.0 -0.5 -1.0
f1 (ppm)

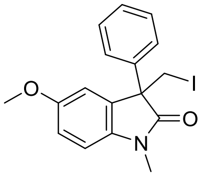
O
I
O
N

1cb

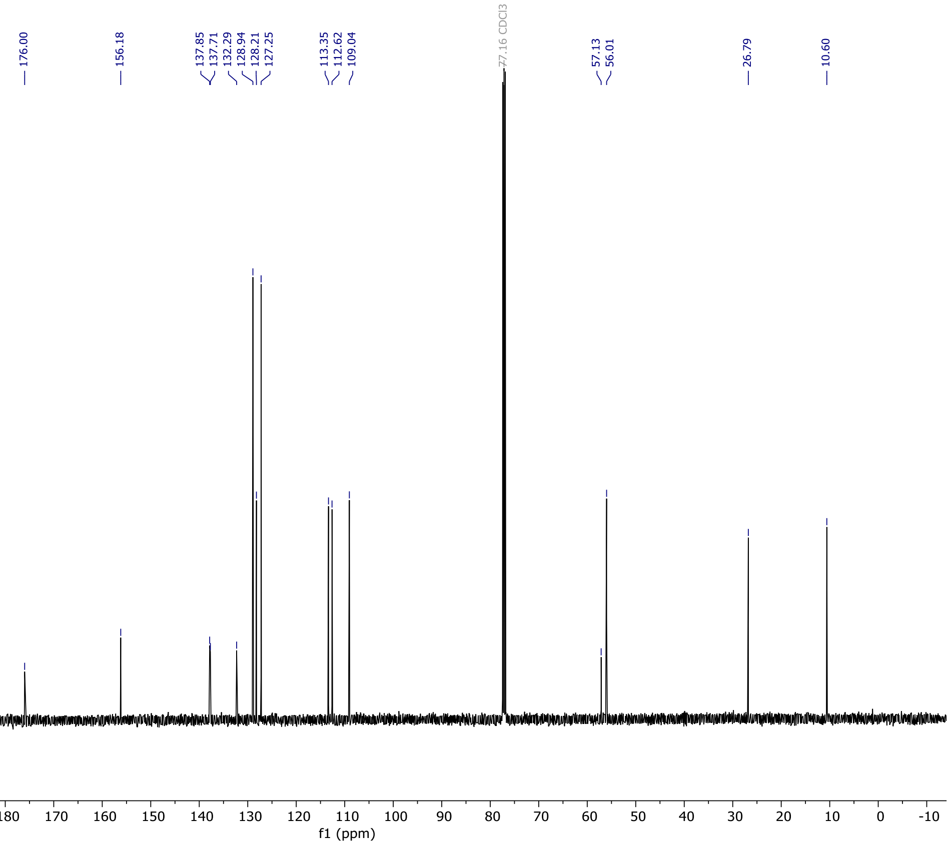
176.00
156.18
137.85 137.71 132.29 128.94 128.21 127.25
113.35 112.62 109.04
77.16 CDCl3
57.13 56.01
26.79
10.60
230 220 210 200 190 180 170 160 150 140 130 120 110 100 90 80 70 60 50 40 30 20 10 0 -10
f1 (ppm)

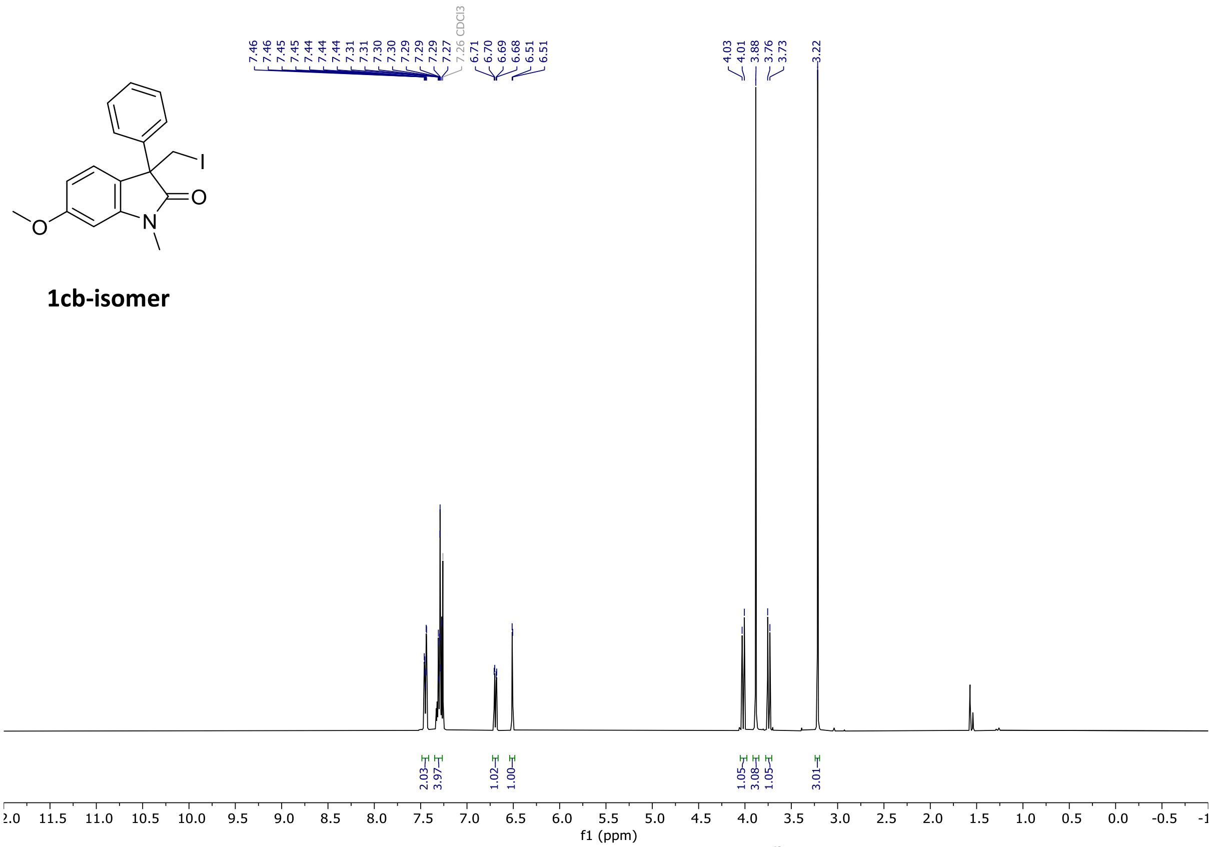
O
N
O
I
1cb-isomer
f1 (ppm)

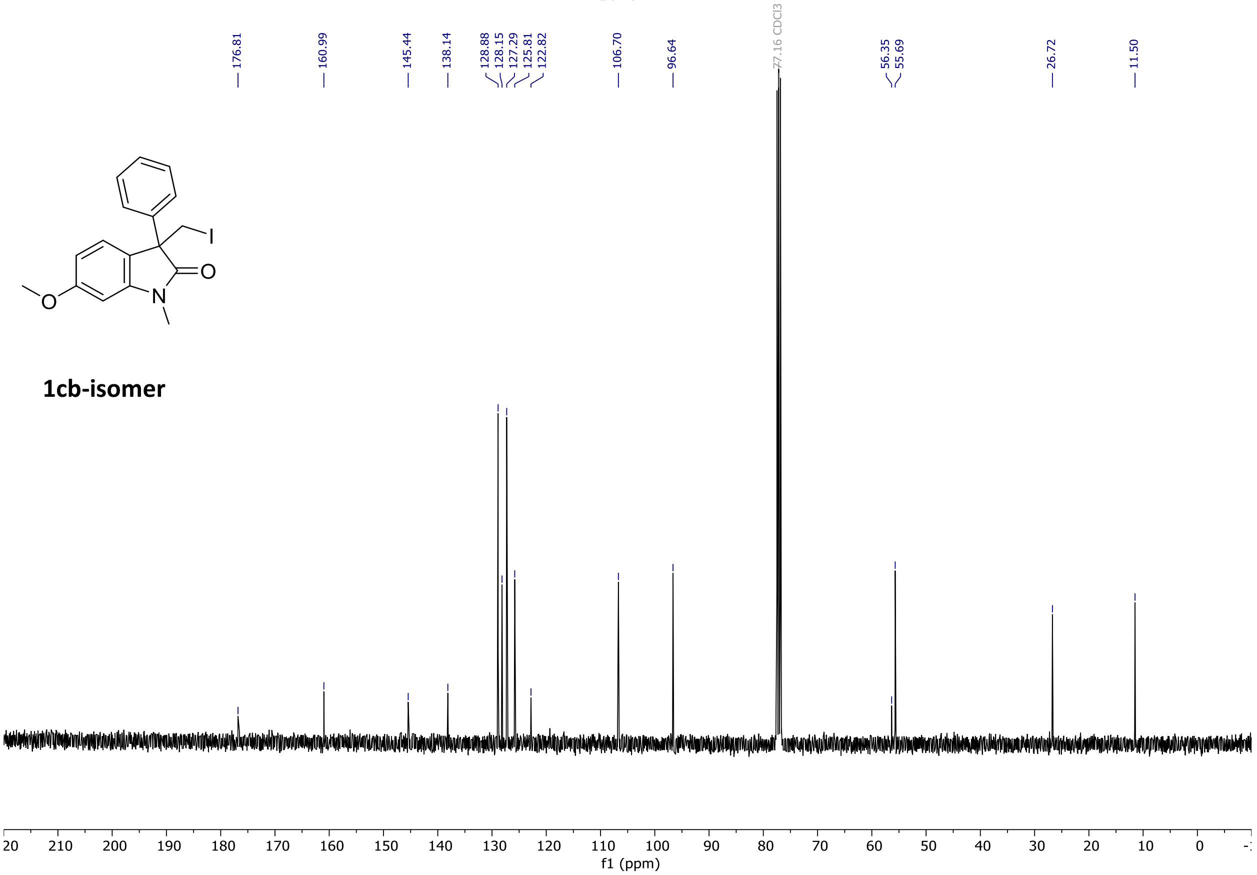
O
N
O
I
1cb-isomer
176.81
160.99
145.44
138.14
128.88
128.15
127.29
125.81
122.82
106.70
96.64
77.16 CDCl3
56.35
55.69
26.72
11.50
f1 (ppm)

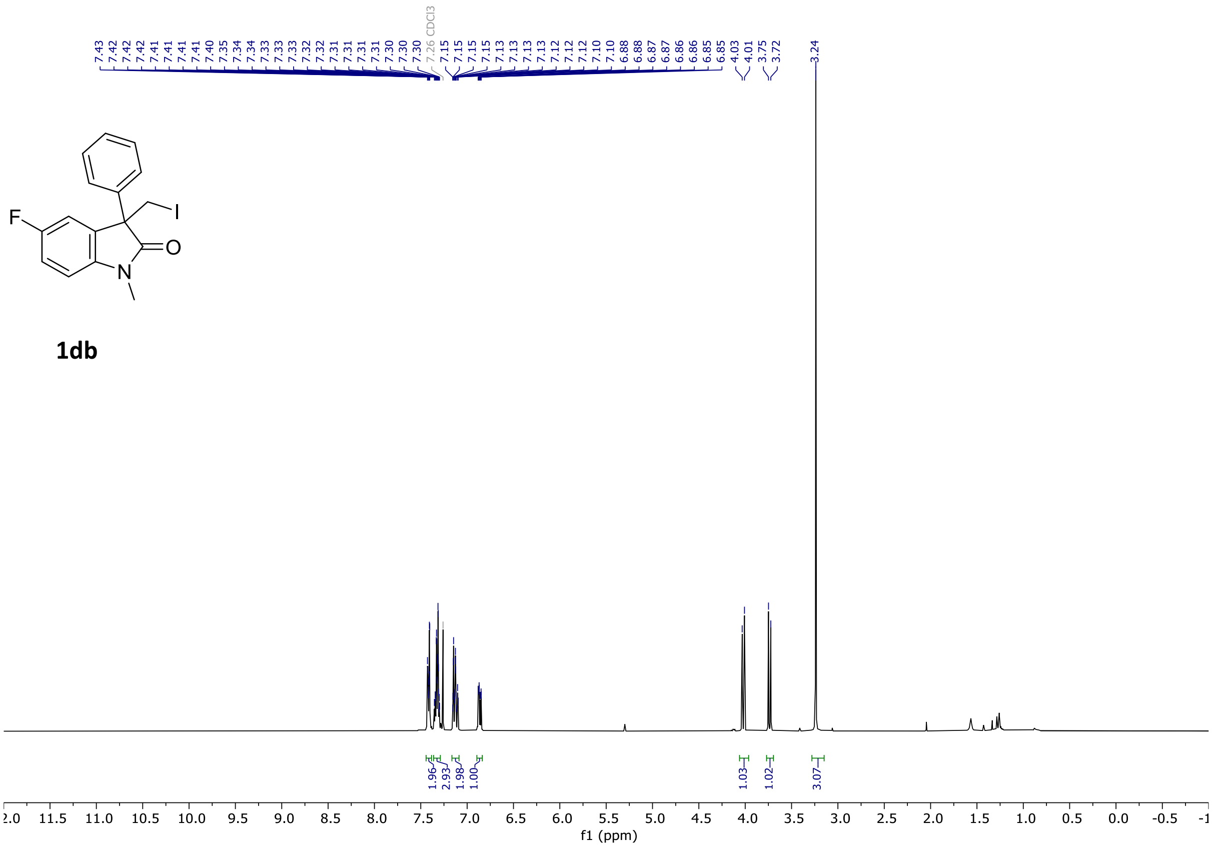
7.43
7.42
7.42
7.42
7.41
7.41
7.41
7.41
7.40
7.35
7.34
7.34
7.33
7.33
7.33
7.32
7.32
7.31
7.31
7.31
7.31
7.30
7.30
7.30
7.26 CDCl3
7.15
7.15
7.15
7.15
7.13
7.13
7.13
7.13
7.12
7.12
7.12
7.10
7.10
6.88
6.88
6.87
6.87
6.86
6.86
6.85
6.85
4.03
4.01
3.75
3.72
3.24
F
I
O
N
1.96
2.93
1.98
1.00
1.03
1.02
3.07
f1 (ppm)

1db

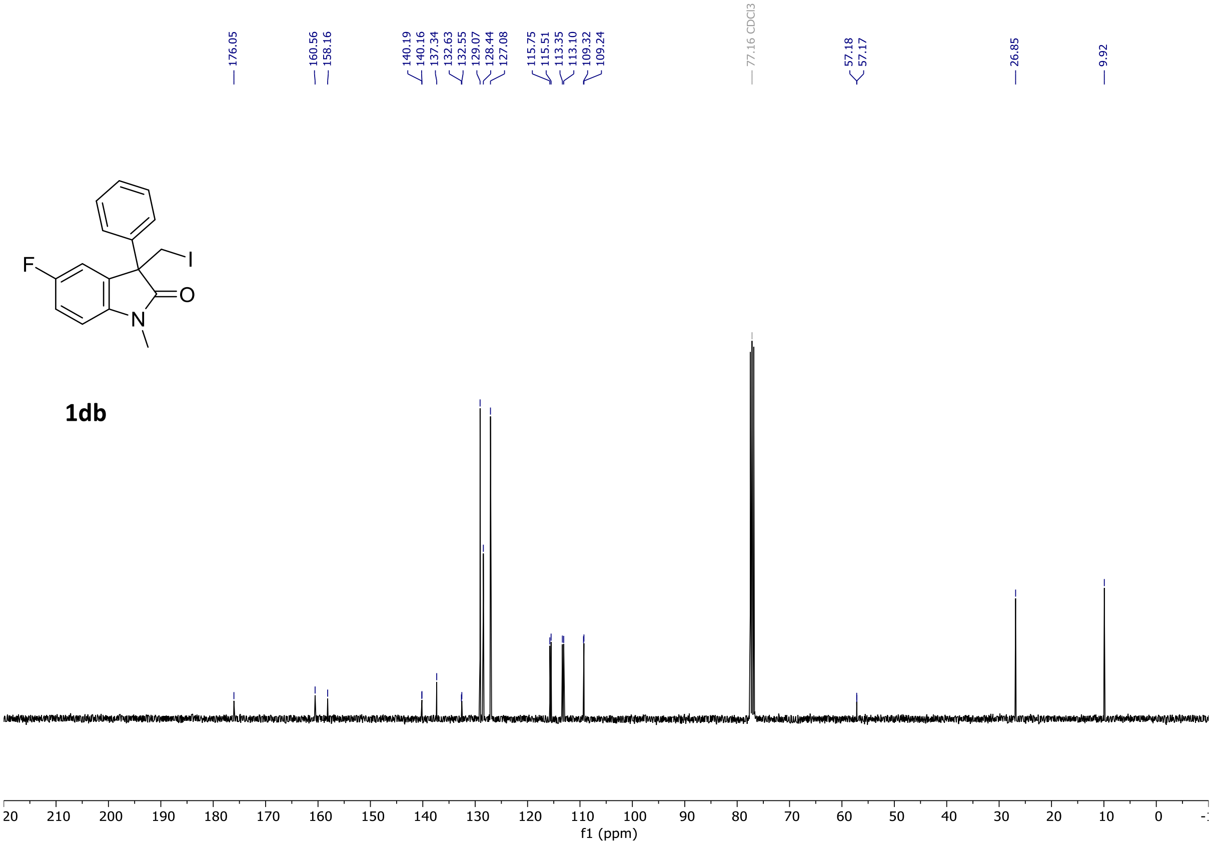
176.05
160.56
158.16
140.19
140.16
137.34
132.63
132.55
129.07
128.44
127.08
115.75
115.51
113.35
113.10
109.32
109.24
77.16 CDCl3
57.18
57.17
26.85
9.92
F
I
O
N
f1 (ppm)

1db

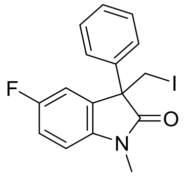

1db

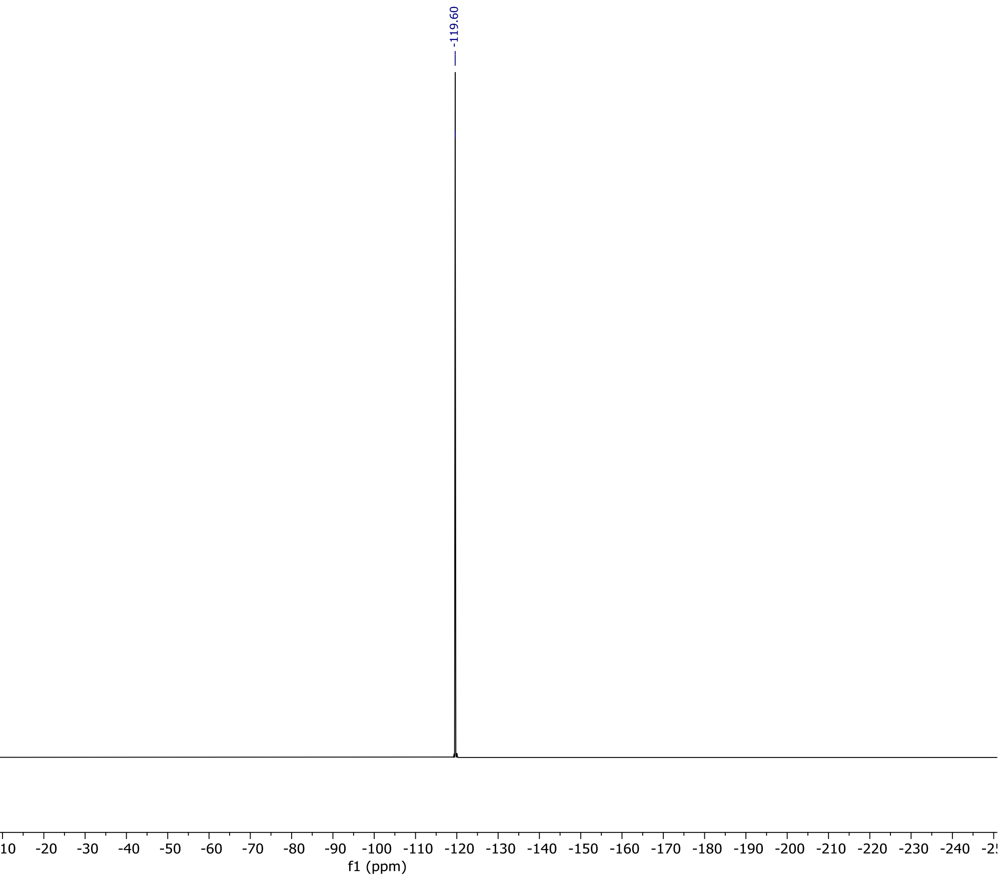

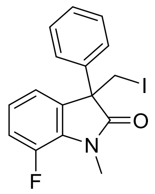

1eb

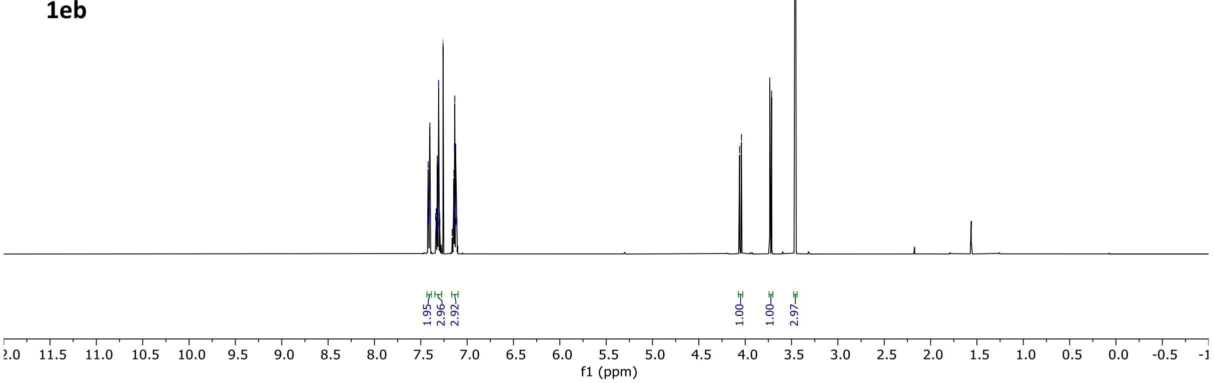

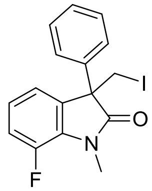

1eb

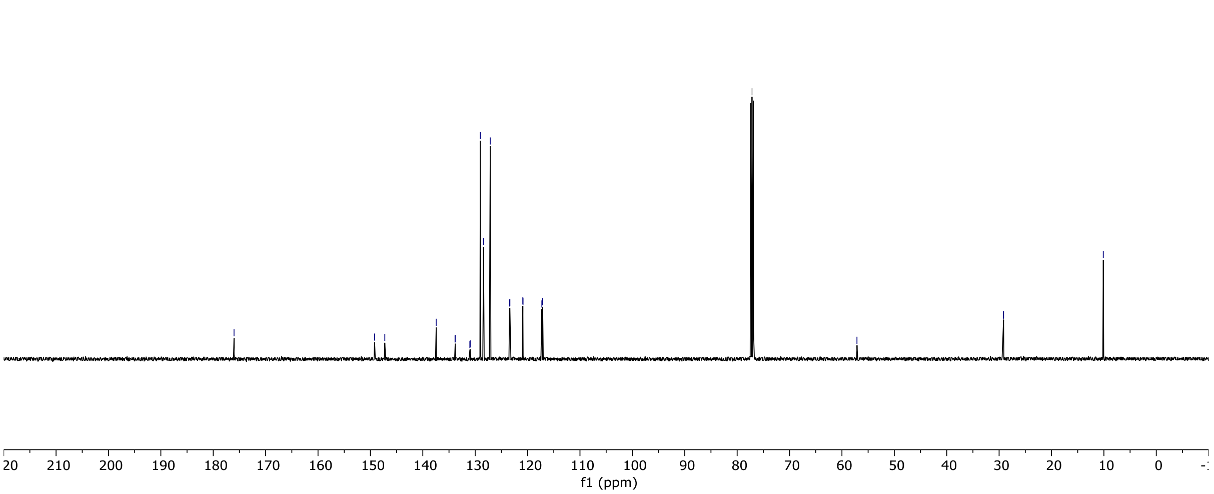
176.03
149.19
147.25
137.45
133.84
133.82
131.02
130.95
129.03
128.41
127.13
123.43
123.38
120.90
120.88
117.28
117.12
77.16 CDCl3
57.13
29.20
29.15
10.12
f1 (ppm)

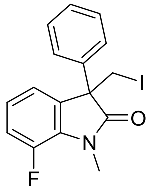

1eb

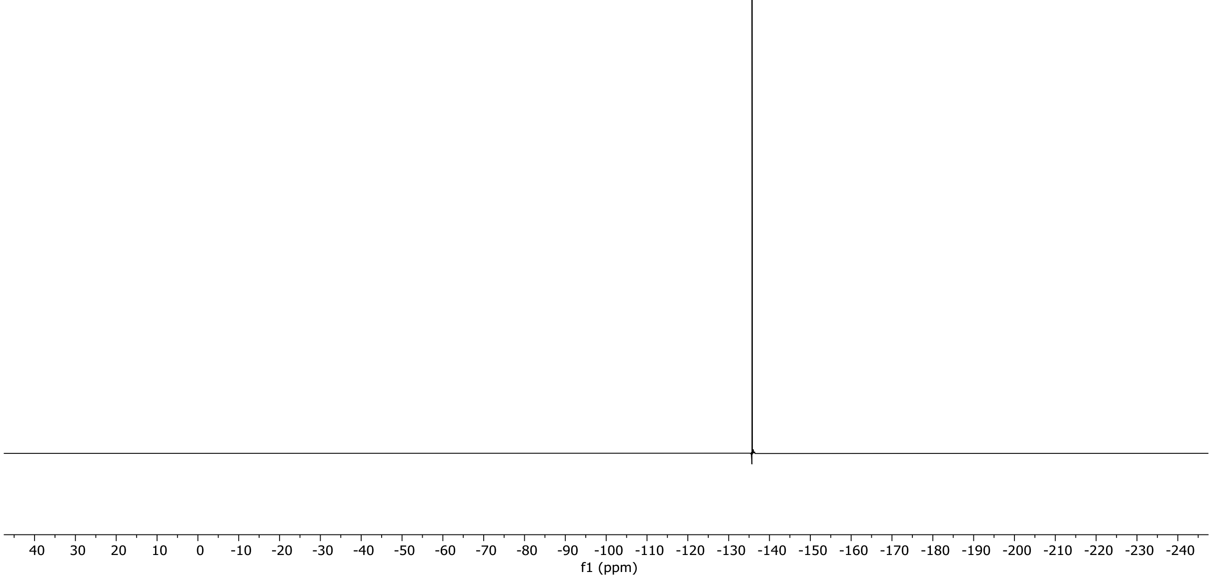
-135.71
f1 (ppm)

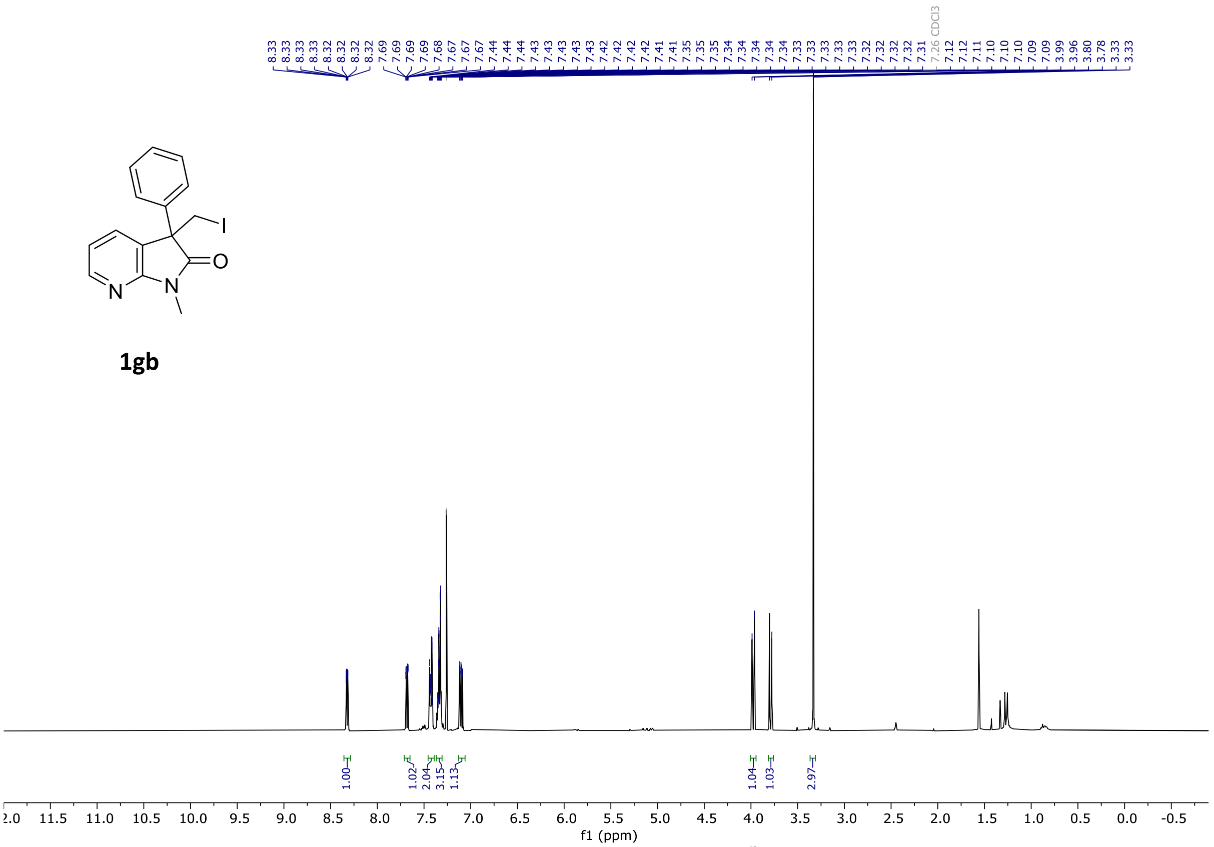
8.33 8.33 8.33 8.33 8.32 8.32 8.32 7.69 7.69 7.69 7.68 7.67 7.67 7.67 7.44 7.44 7.43 7.43 7.43 7.43 7.43 7.42 7.42 7.42 7.41 7.35 7.35 7.34 7.34 7.34 7.34 7.33 7.33 7.33 7.33 7.32 7.32 7.32 7.31 7.26 CDCl3 7.12 7.12 7.11 7.10 7.10 7.09 7.09 3.99 3.96 3.80 3.78 3.33 3.33
1gb
1.00
1.02
2.04
3.15
1.13
1.04
1.03
2.97
f1 (ppm)

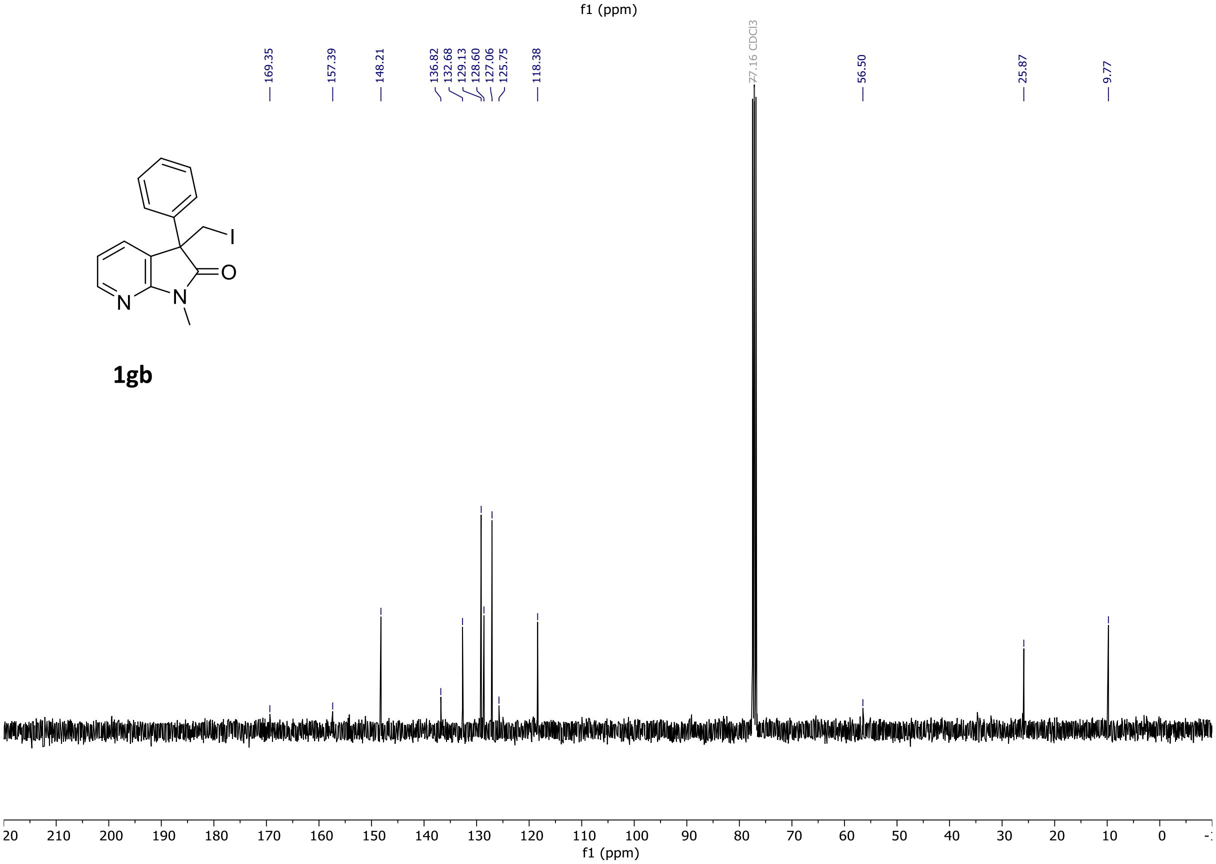
169.35
157.39
148.21
136.82
132.68
129.13
128.60
127.06
125.75
118.38
77.16 CDCl3
56.50
25.87
9.77
1gb
f1 (ppm)

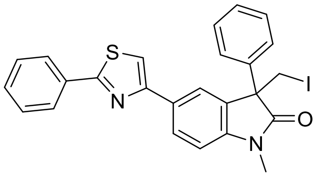
S
N
I
O
N

1hb

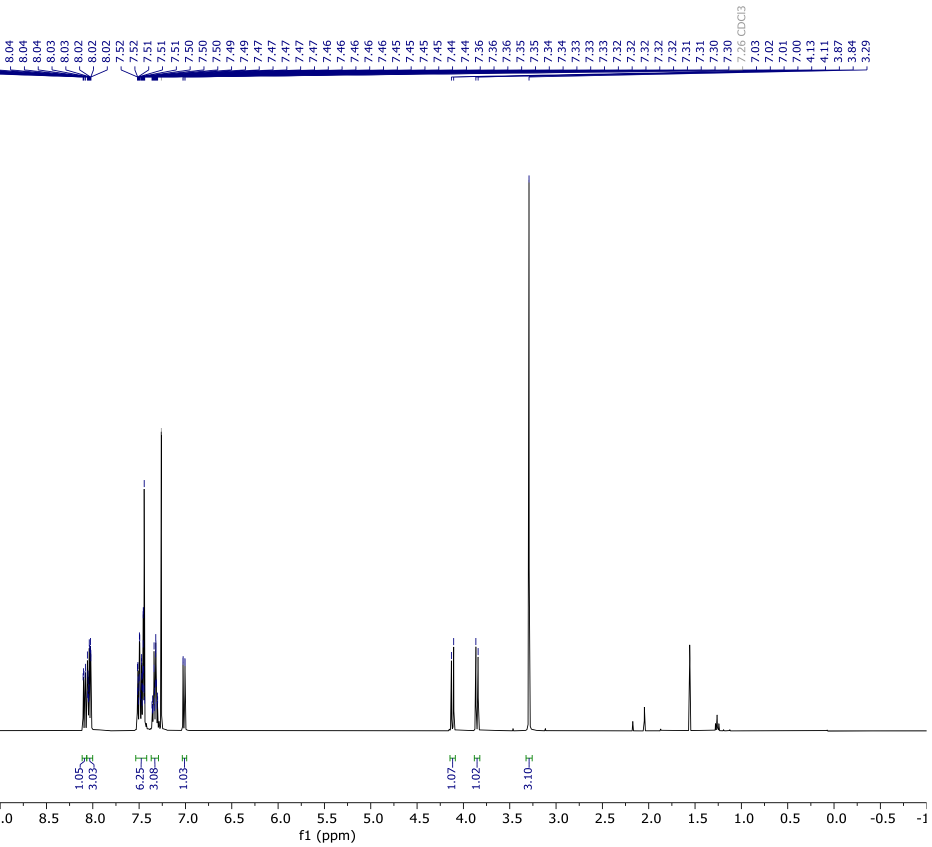
8.10 8.10 8.08 8.06 8.05 8.05 8.05 8.04 8.04 8.04 8.03 8.02 8.02 8.02 7.52 7.51 7.51 7.51 7.50 7.50 7.49 7.49 7.47 7.47 7.47 7.46 7.46 7.46 7.46 7.45 7.45 7.45 7.44 7.44 7.36 7.36 7.36 7.35 7.35 7.34 7.33 7.33 7.33 7.32 7.32 7.32 7.32 7.31 7.31 7.30 7.30 7.26 CDCl3 7.03 7.02 7.01 7.00 4.13 4.11 3.87 3.84 3.29
f1 (ppm)

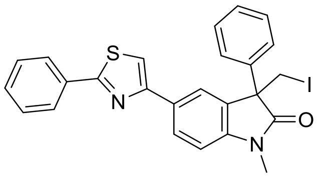
S
N
I
O
N

1hb

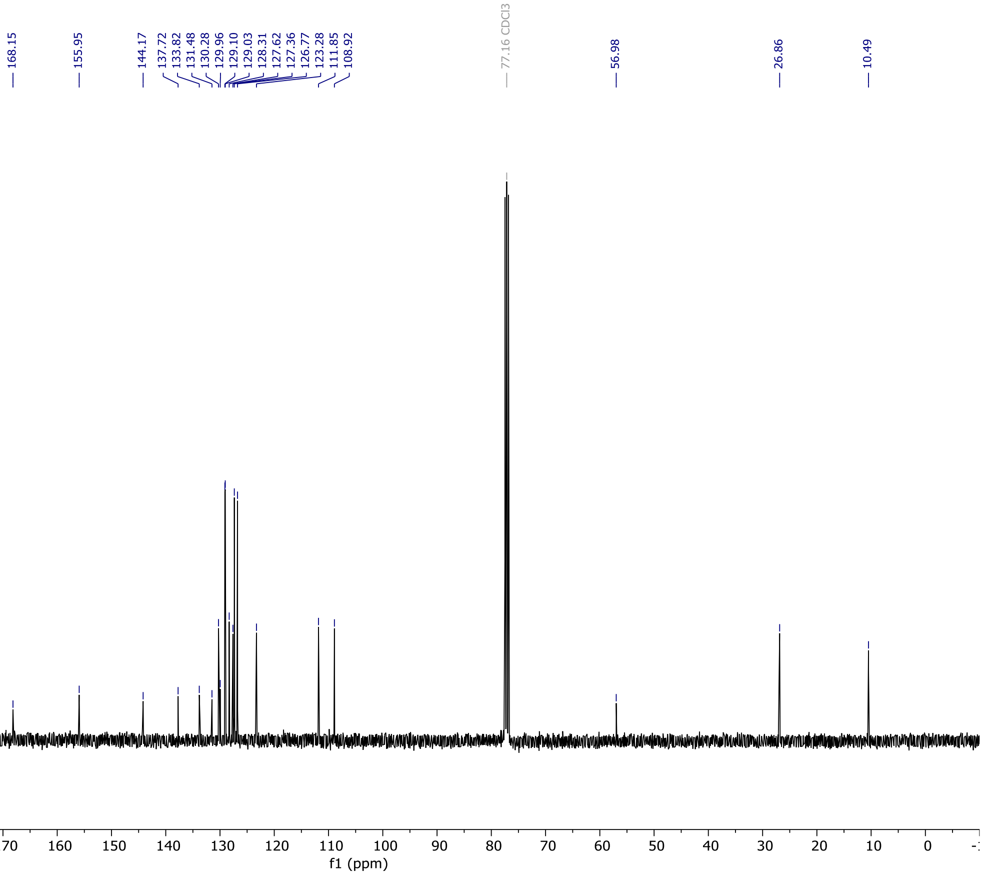
176.47
168.15
155.95
144.17 137.72 133.82 131.48 130.28 129.96 129.10 129.03 128.31 127.62 127.36 126.77 123.28 111.85 108.92
77.16 CDCl3
56.98
26.86
10.49
f1 (ppm)

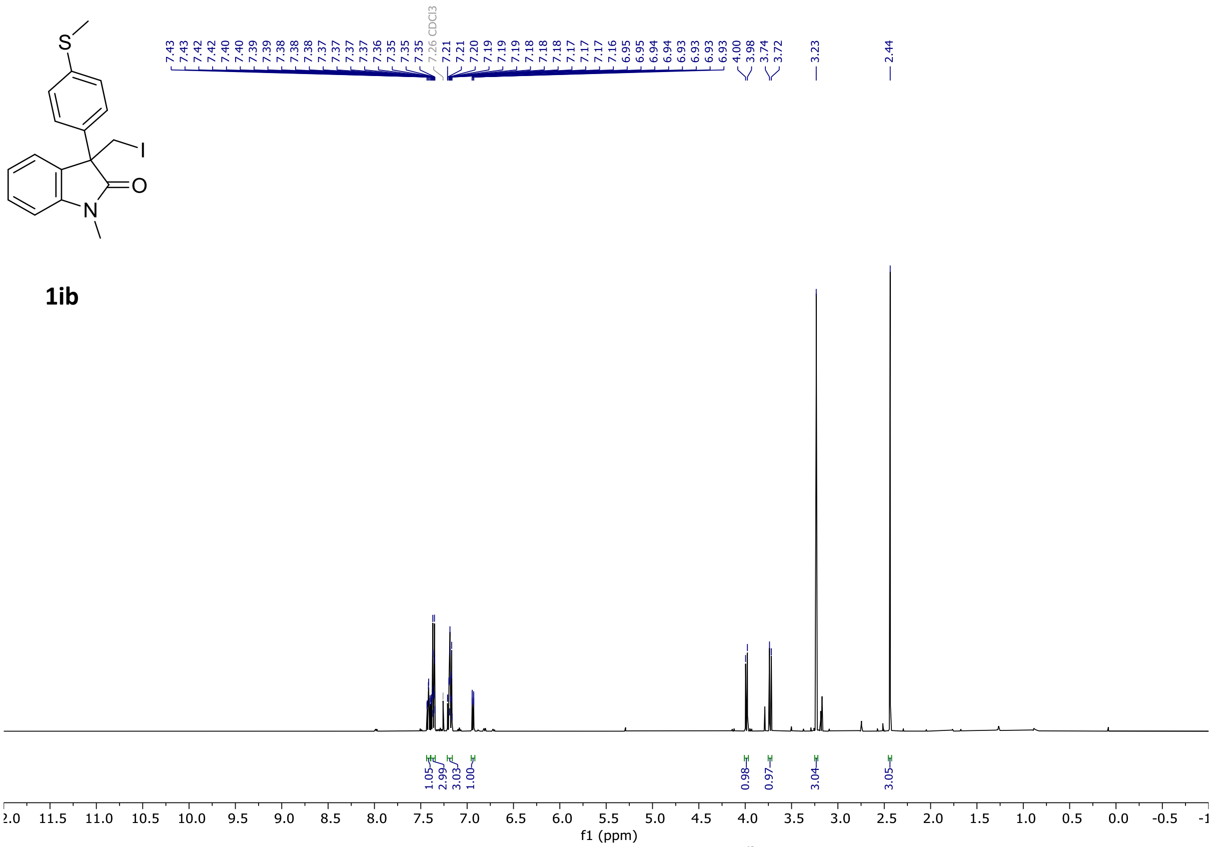

1ib

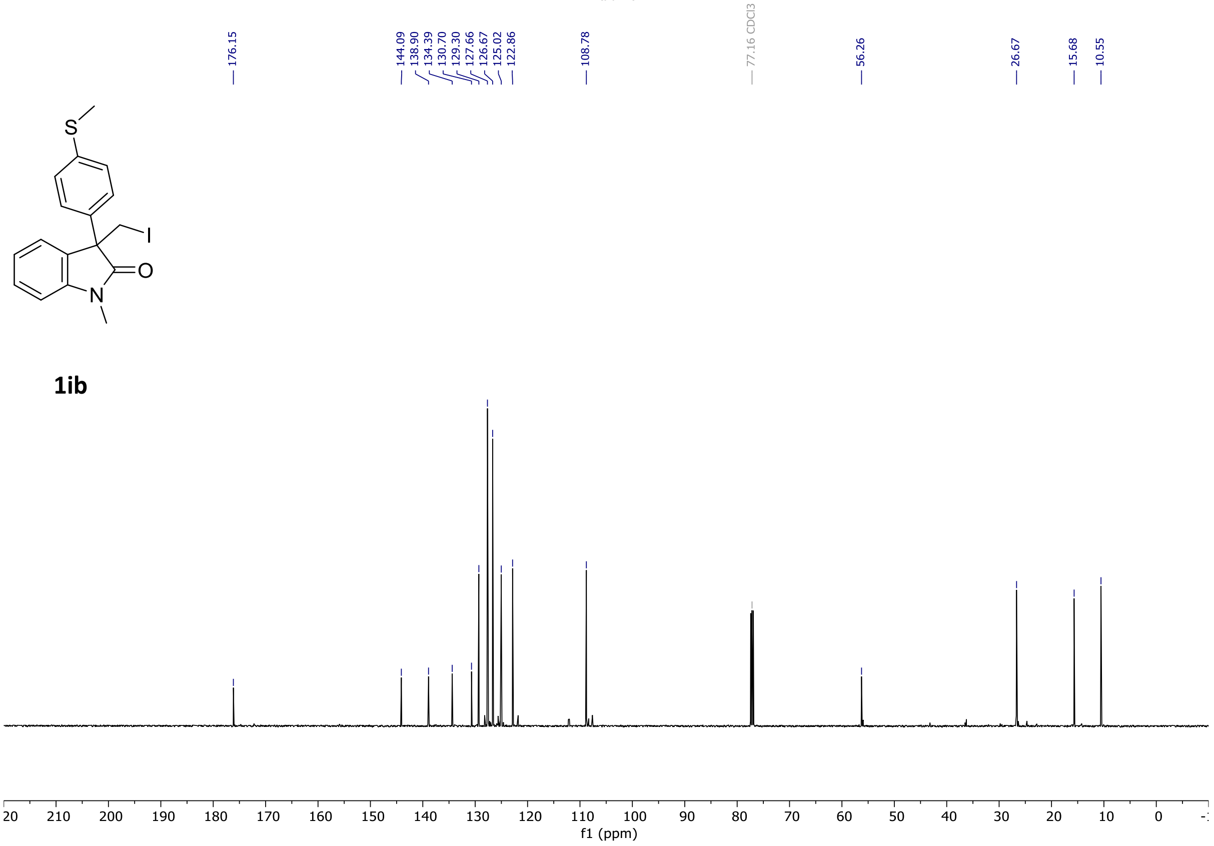

1ib

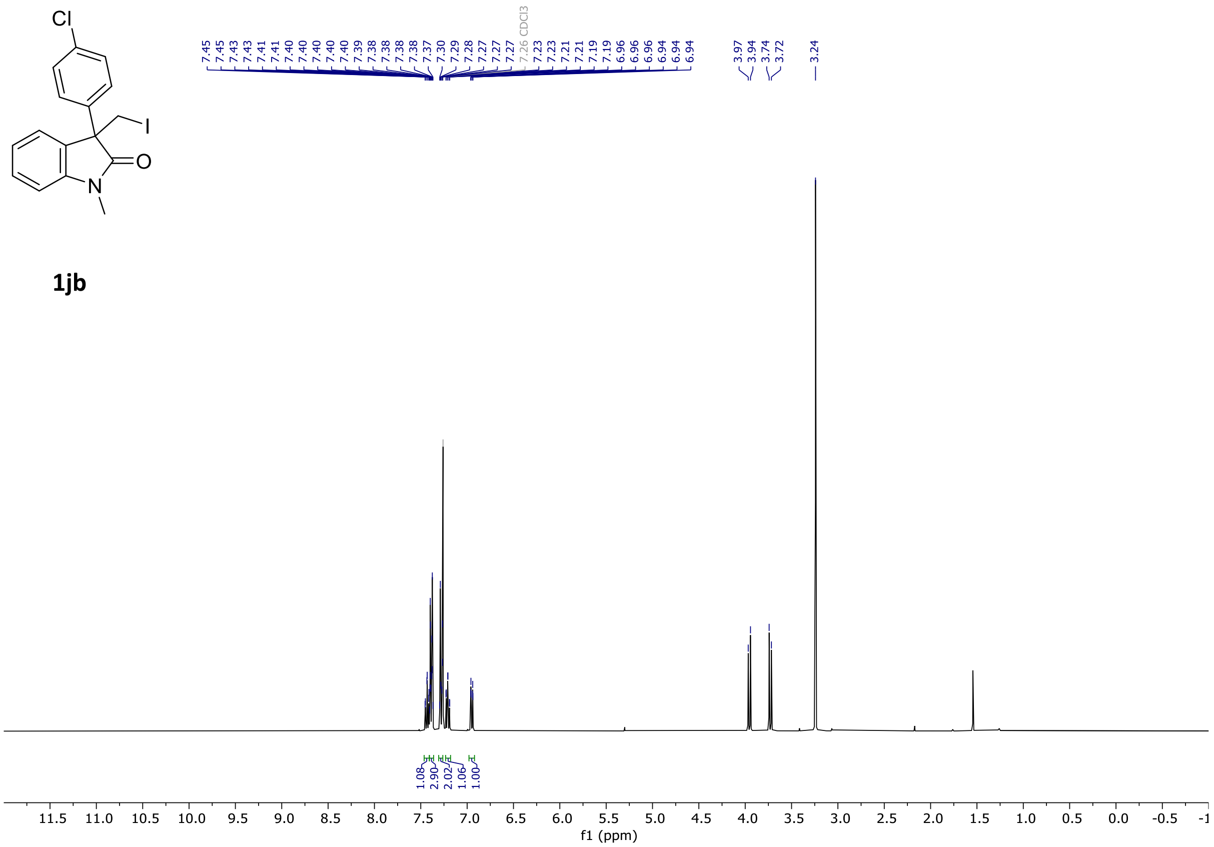

Cl
I
O
N
1jb
7.45 7.45 7.43 7.43 7.41 7.41 7.40 7.40 7.40 7.40 7.39 7.38 7.38 7.38 7.37 7.30 7.29 7.28 7.27 7.27 7.27 7.26 CDCl3 7.23 7.23 7.21 7.21 7.19 7.19 6.96 6.96 6.94 6.94 6.94
3.97 3.94 3.74 3.72
3.24
1.08 2.90 2.02 1.06 1.00
f1 (ppm)

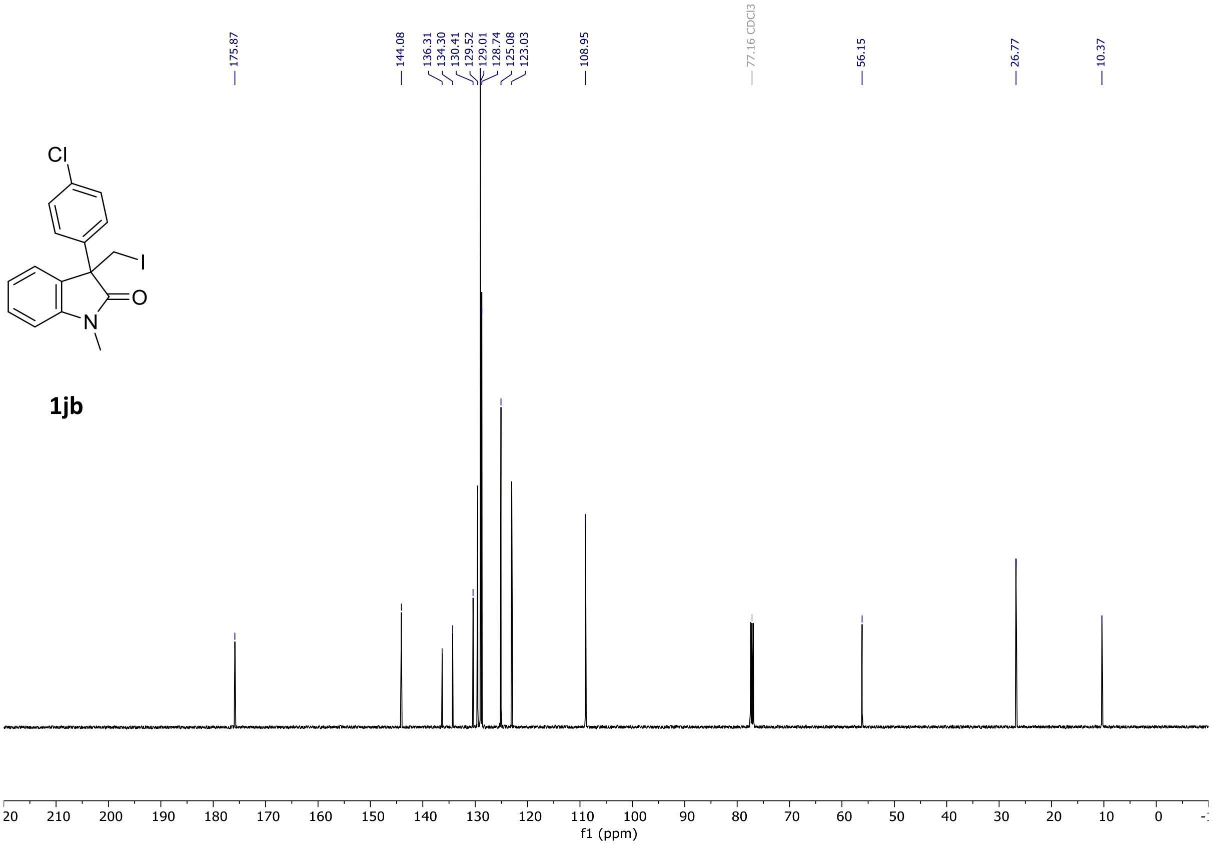

Cl
I
O
N
1jb
175.87
144.08
136.31 134.30 130.41 129.52 129.01 128.74 125.08 123.03
108.95
77.16 CDCl3
56.15
26.77
10.37
f1 (ppm)

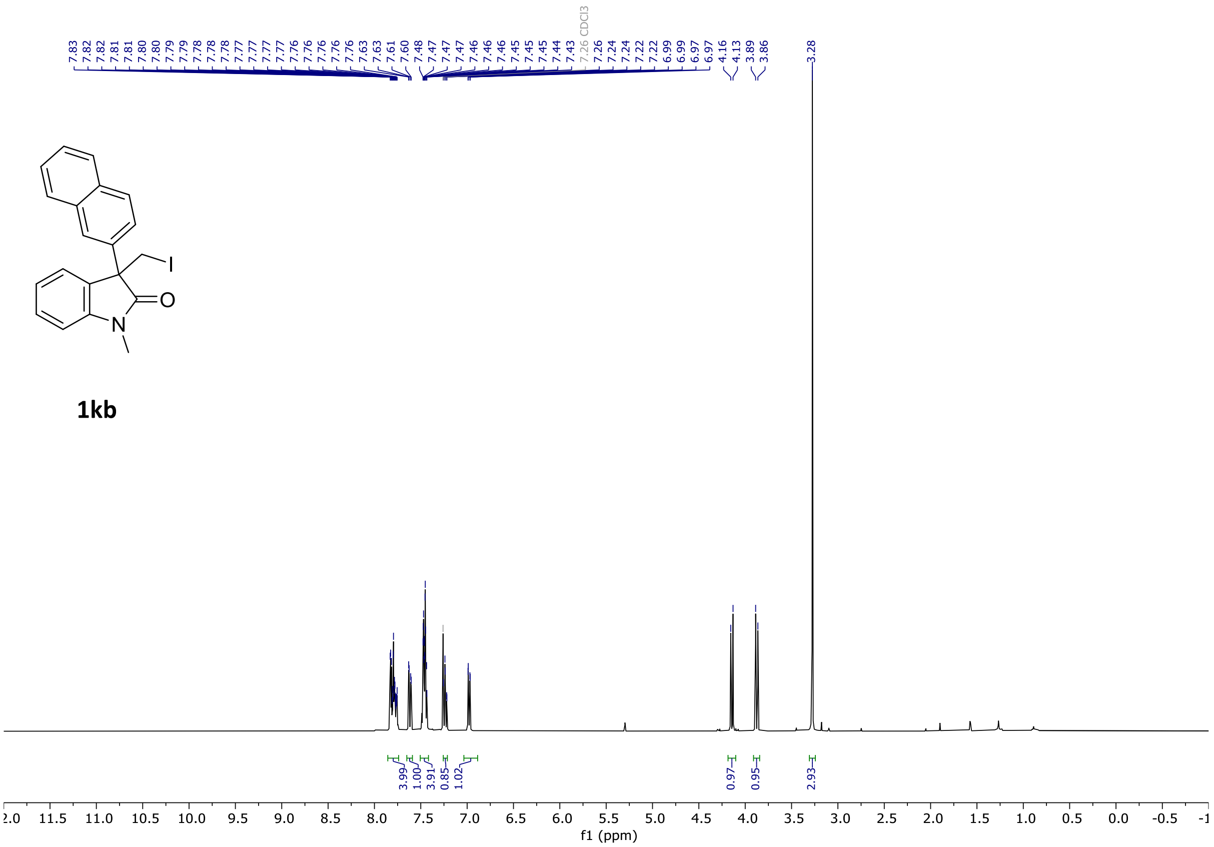
7.83 7.82 7.81 7.81 7.80 7.80 7.79 7.79 7.78 7.78 7.78 7.77 7.77 7.77 7.76 7.76 7.76 7.76 7.76 7.63 7.61 7.60 7.48 7.47 7.47 7.47 7.46 7.46 7.46 7.45 7.45 7.45 7.44 7.43
7.26 CDCl3
7.26 7.24 7.24 7.22 7.22 6.99 6.99 6.97 6.97 4.16 4.13 3.89 3.86 3.28
I
O
N
3.99 1.00 3.91 0.85 1.02 0.97 0.95 2.93
12.0 11.5 11.0 10.5 10.0 9.5 9.0 8.5 8.0 7.5 7.0 6.5 6.0 5.5 5.0 4.5 4.0 3.5 3.0 2.5 2.0 1.5 1.0 0.5 0.0 -0.5 -1
f1 (ppm)

1kb

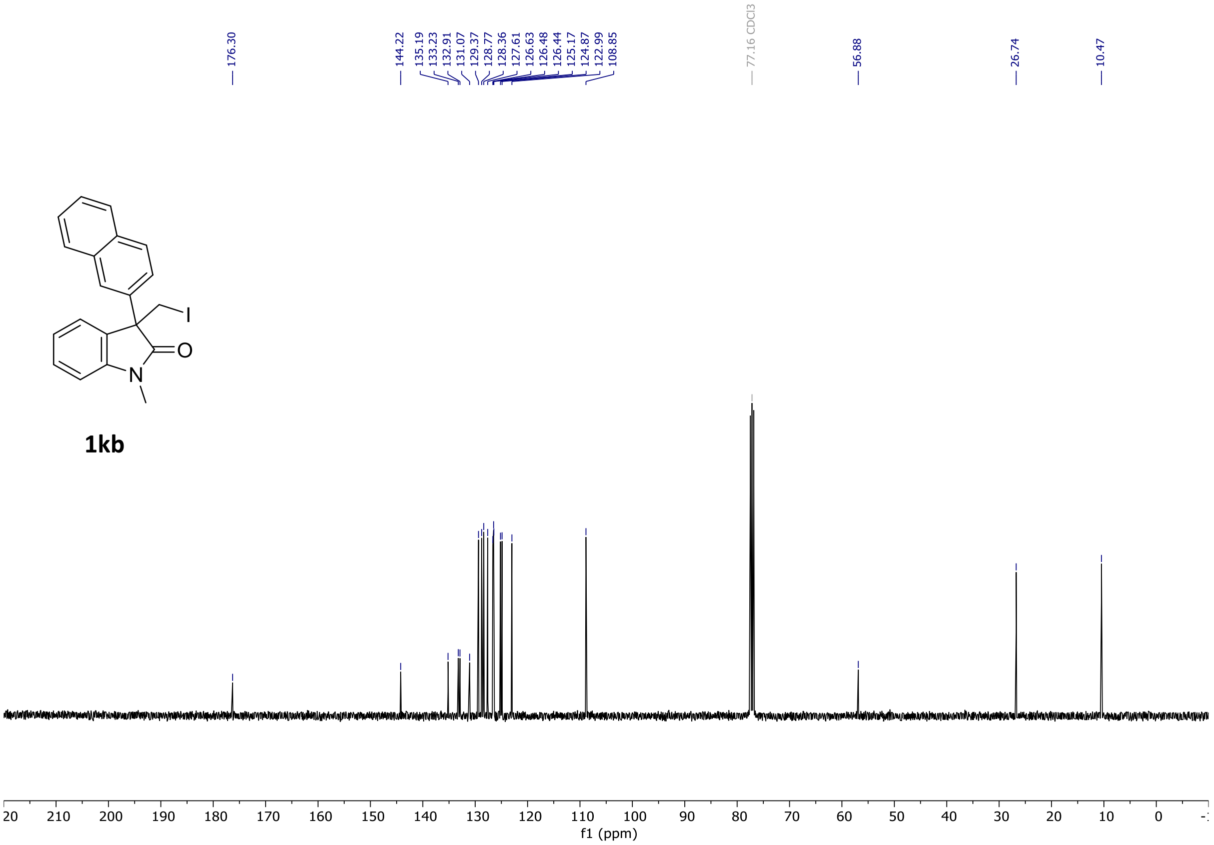
176.30
144.22 135.19 133.23 132.91 131.07 129.37 128.77 128.36 127.61 126.63 126.48 126.44 125.17 124.87 122.99 108.85
77.16 CDCl3
56.88
26.74
10.47
I
O
N
220 210 200 190 180 170 160 150 140 130 120 110 100 90 80 70 60 50 40 30 20 10 0 -10
f1 (ppm)

1kb

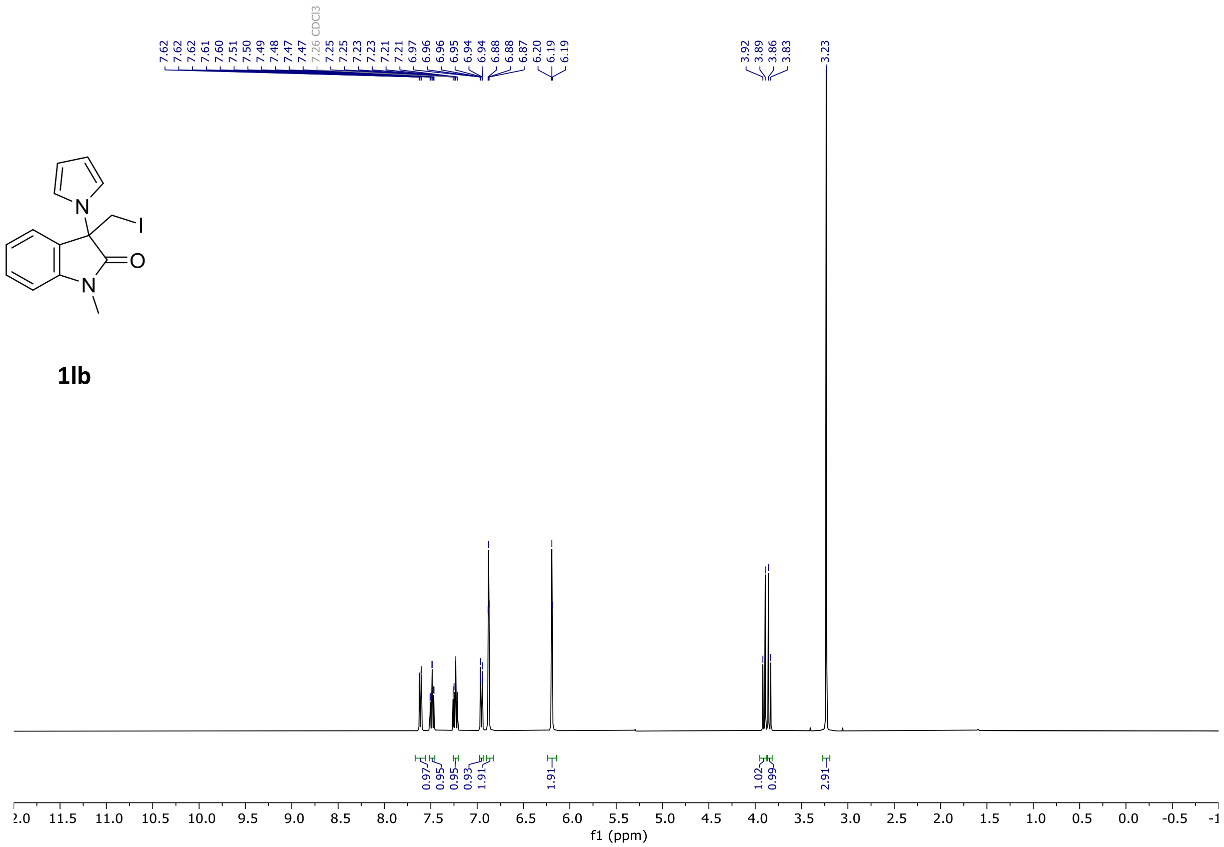
7.62 7.62 7.62 7.61 7.60 7.51 7.50 7.49 7.48 7.47 7.47 7.26 CDCl3 7.25 7.25 7.25 7.23 7.23 7.21 7.21 6.97 6.96 6.96 6.95 6.94 6.94 6.88 6.88 6.87 6.20 6.19 6.19
3.92 3.89 3.86 3.83
3.23
1lb
0.97 0.95 0.95 0.93 1.91 1.91 1.02 0.99 2.91
f1 (ppm)

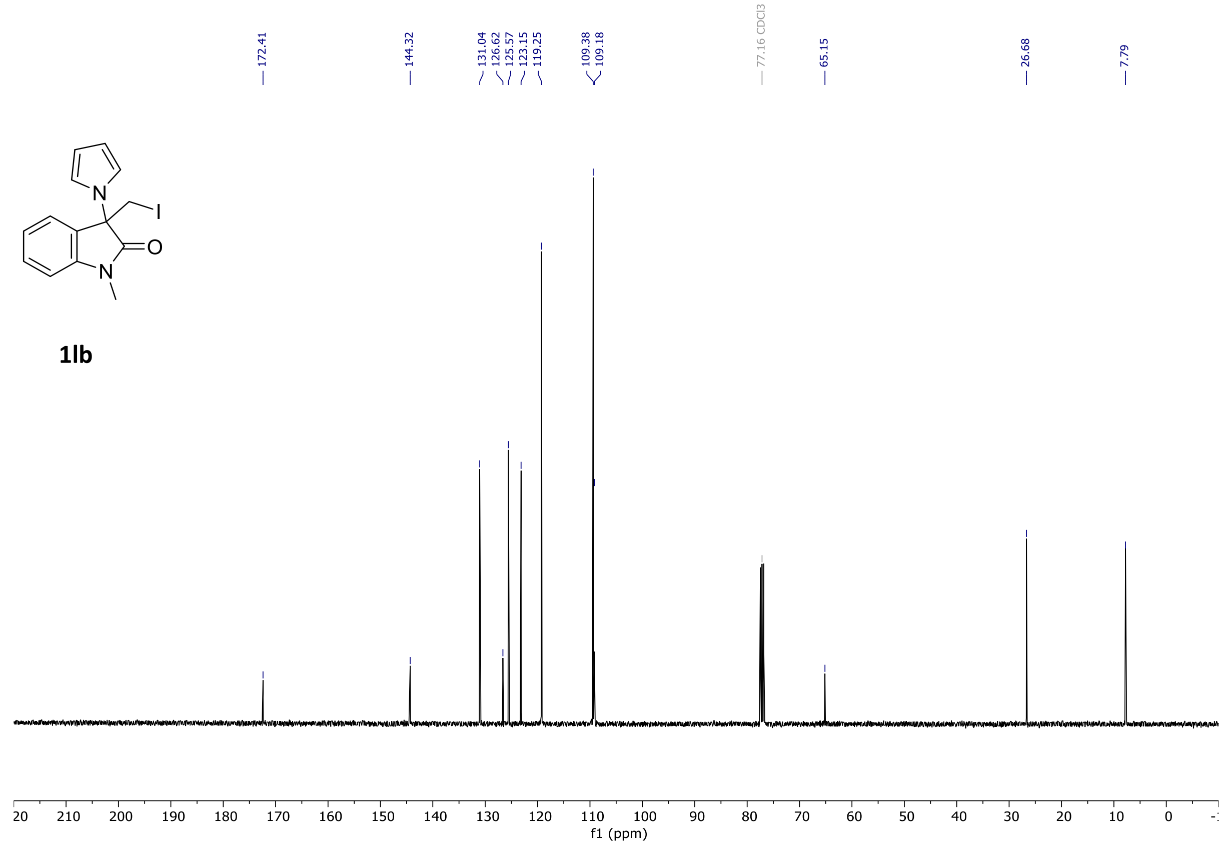
172.41
144.32
131.04 126.62 125.57 123.15 119.25
109.38 109.18
77.16 CDCl3
65.15
26.68
7.79
1lb
f1 (ppm)

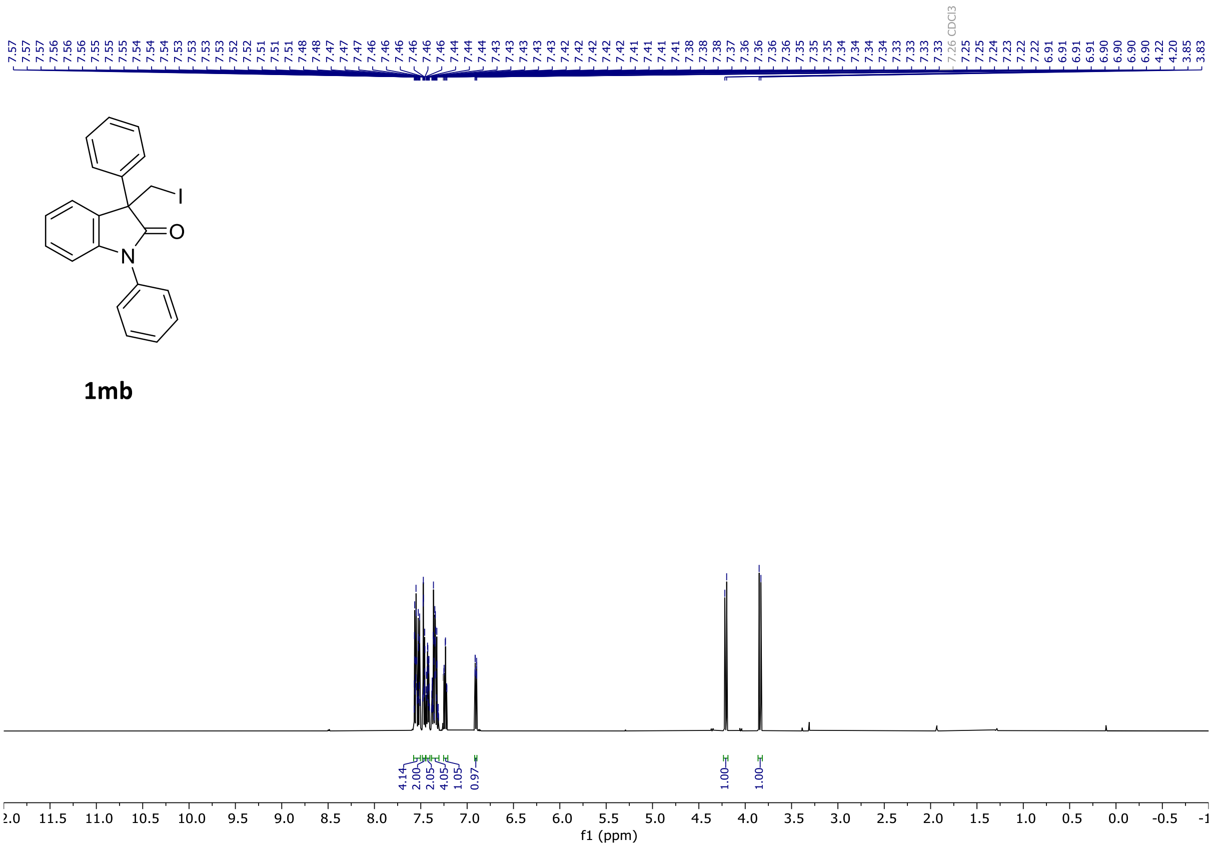

1mb

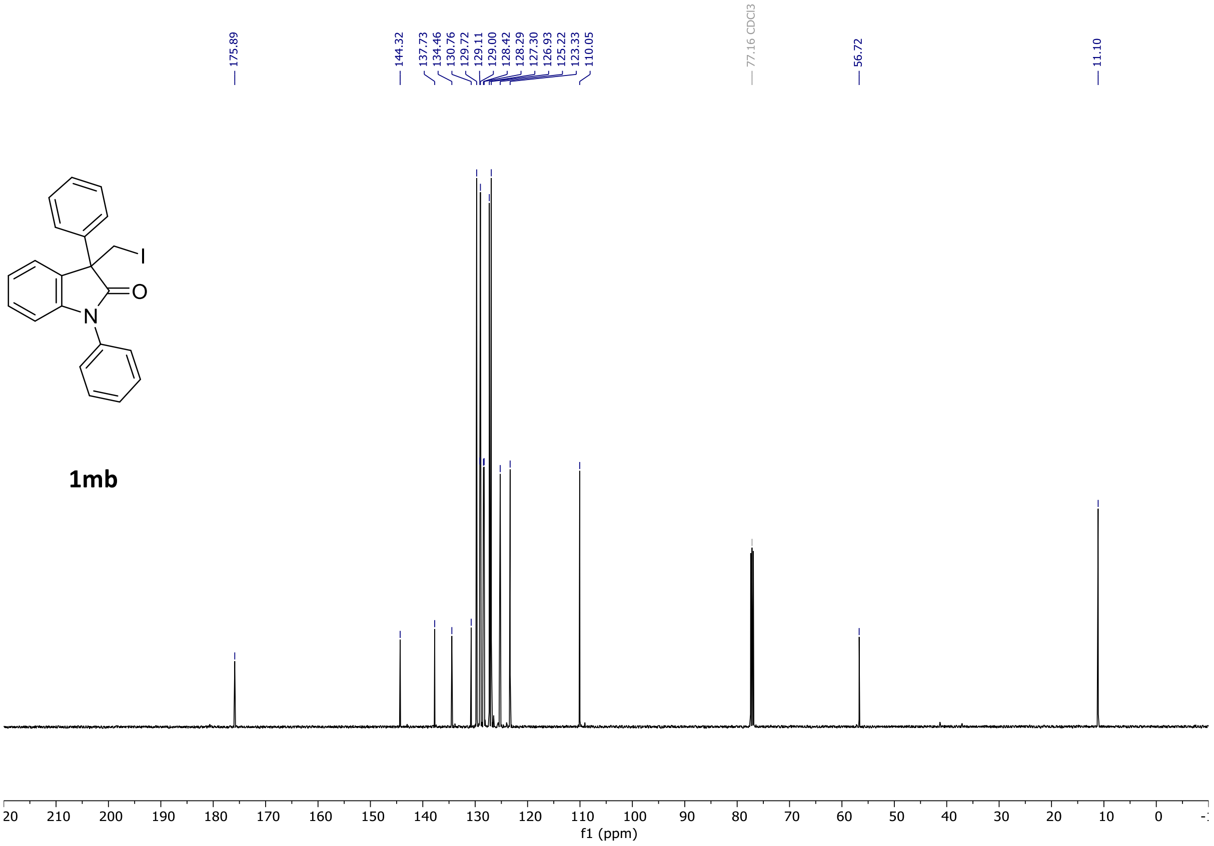

1mb

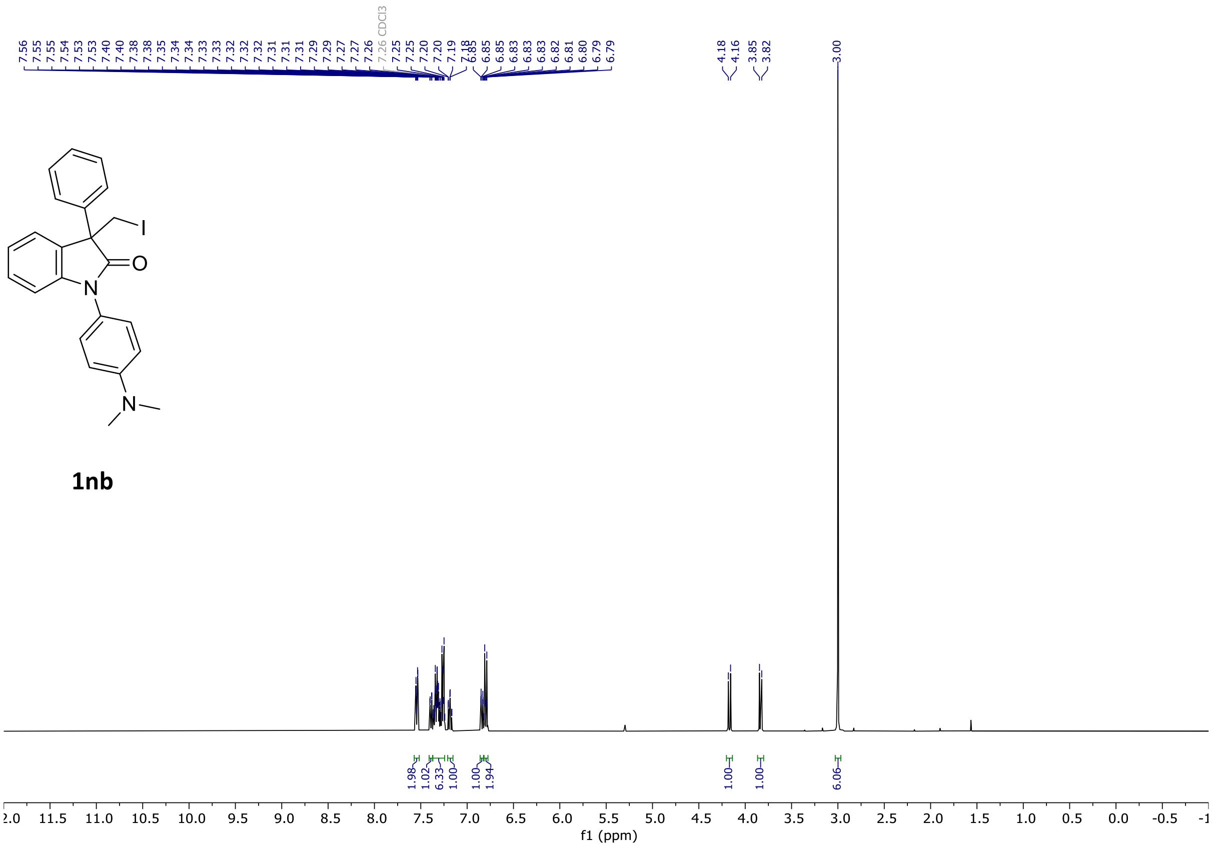
7.56 7.55 7.55 7.54 7.53 7.53 7.40 7.40 7.38 7.38 7.35 7.34 7.33 7.33 7.32 7.32 7.31 7.31 7.29 7.29 7.27 7.27 7.26 7.26 CDCl3 7.25 7.25 7.20 7.20 7.19 7.18 6.88 6.85 6.85 6.83 6.83 6.82 6.81 6.80 6.79 6.79
4.18 4.16 3.85 3.82
3.00
I
O
N
N
1.98 1.02 6.33 1.00 1.00 1.94 1.00 1.00 6.06
2.0 11.5 11.0 10.5 10.0 9.5 9.0 8.5 8.0 7.5 7.0 6.5 6.0 5.5 5.0 4.5 4.0 3.5 3.0 2.5 2.0 1.5 1.0 0.5 0.0 -0.5 -1
f1 (ppm)

1nb

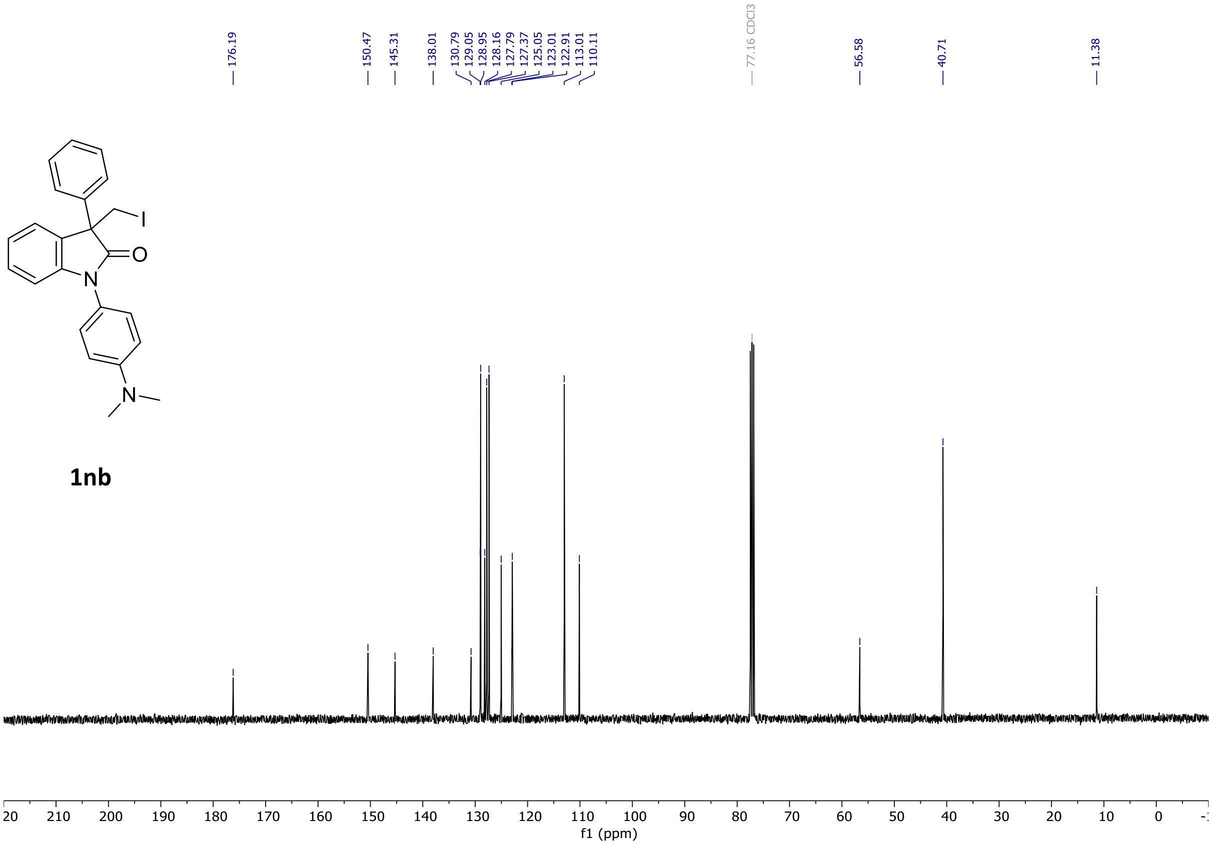
176.19
150.47 145.31 138.01 130.79 129.05 128.95 128.16 127.79 127.37 125.05 123.01 122.91 113.01 110.11
77.16 CDCl3
56.58
40.71
11.38
I
O
N
N
20 210 200 190 180 170 160 150 140 130 120 110 100 90 80 70 60 50 40 30 20 10 0
f1 (ppm)

1nb

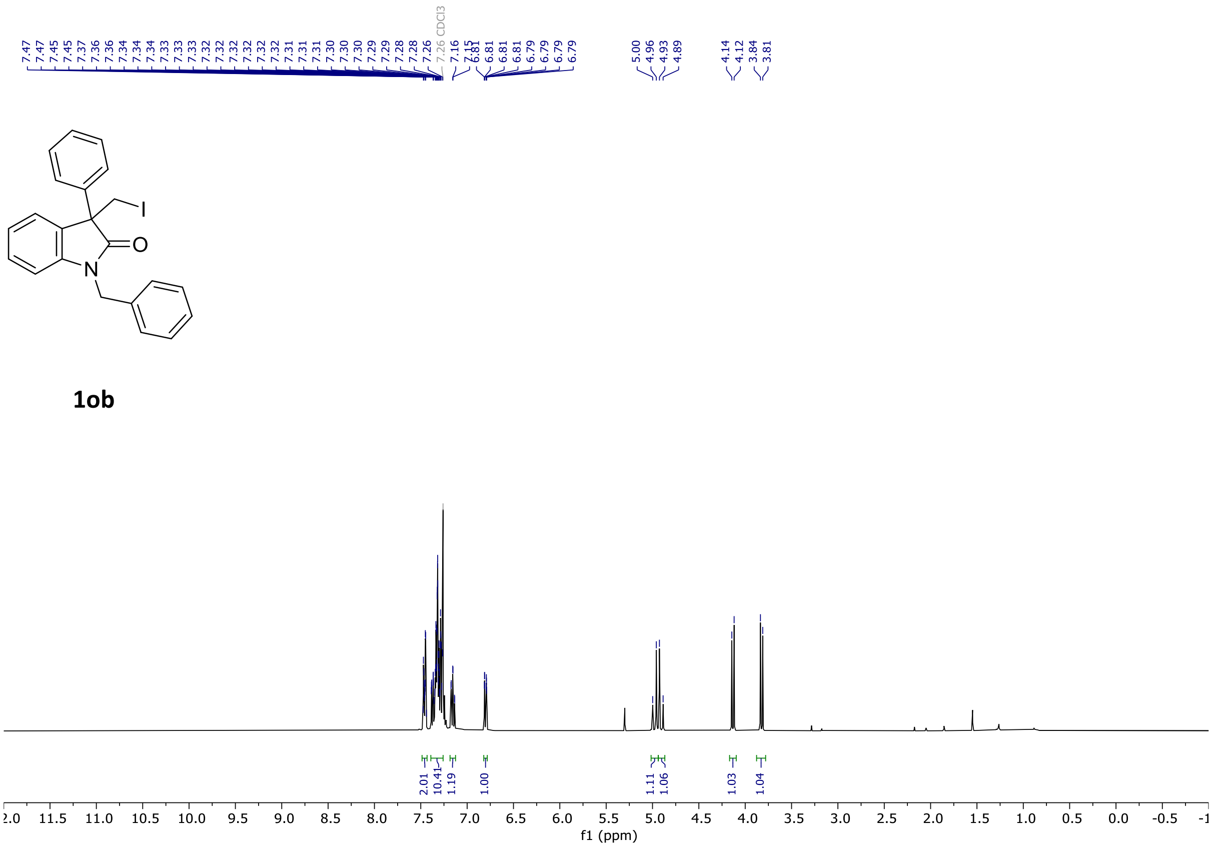
7.47 7.47 7.45 7.45 7.37 7.36 7.36 7.34 7.34 7.33 7.33 7.33 7.32 7.32 7.32 7.32 7.32 7.31 7.31 7.31 7.30 7.30 7.29 7.29 7.28 7.26 7.26 CDCl3 7.16 7.15 6.81 6.81 6.81 6.81 6.79 6.79 6.79
5.00 4.96 4.93 4.89
4.14 4.12 3.84 3.81
I
O
N
1ob
2.01 10.41 1.19 1.00 1.11 1.06 1.03 1.04
f1 (ppm)

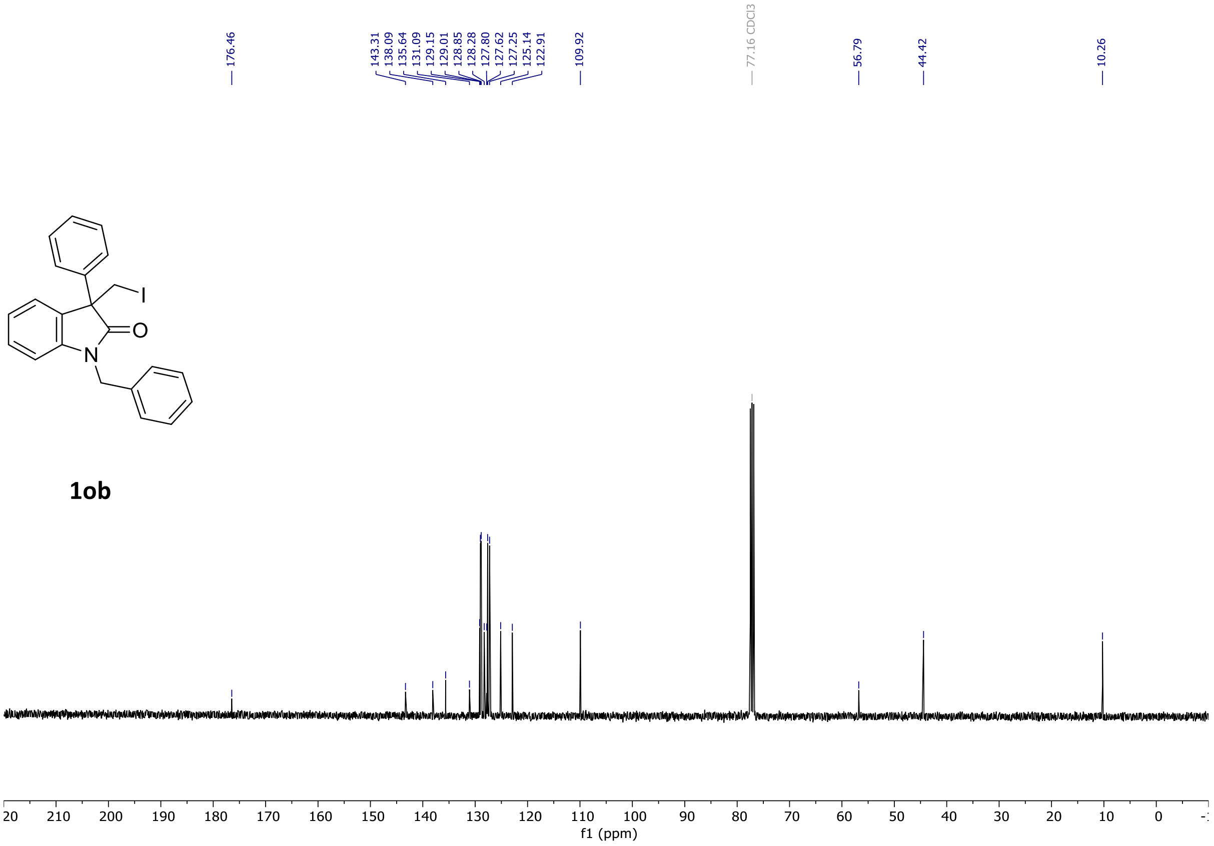
176.46
143.31 138.09 135.64 131.09 129.15 129.01 128.85 128.28 127.80 127.62 127.25 125.14 122.91
109.92
77.16 CDCl3
56.79
44.42
10.26
I
O
N
1ob
f1 (ppm)

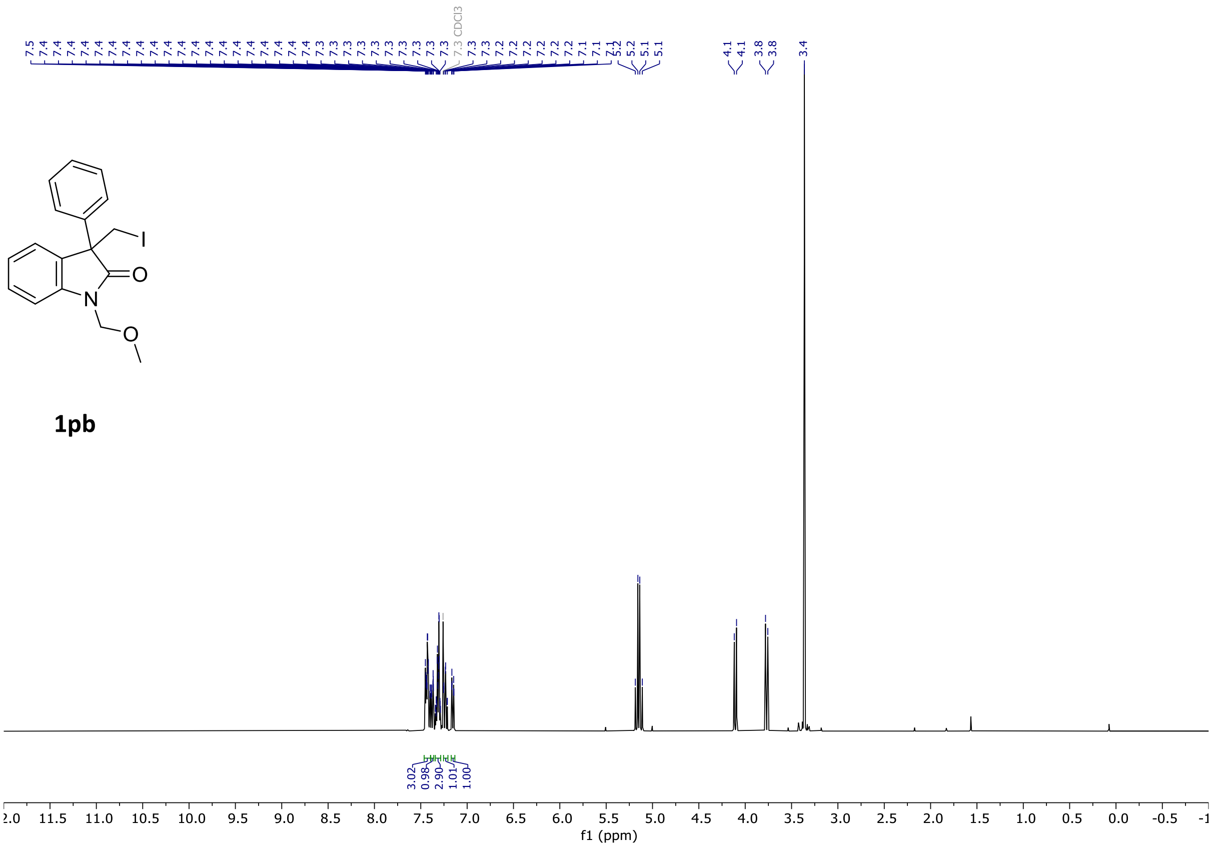
7.5 7.4 7.4 7.4 7.4 7.4 7.4 7.4 7.4 7.4 7.4 7.4 7.4 7.4 7.4 7.4 7.4 7.4 7.3 7.3 7.3 7.3 7.3 7.3 7.3 7.3 7.3 7.3 CDCl3 7.3 7.3 7.2 7.2 7.2 7.2 7.2 7.2 7.1 7.1 5.2 5.2 5.1 5.1
4.1 4.1 3.8 3.8 3.4
3.02 0.98 2.90 1.01 1.00
f1 (ppm)

1pb

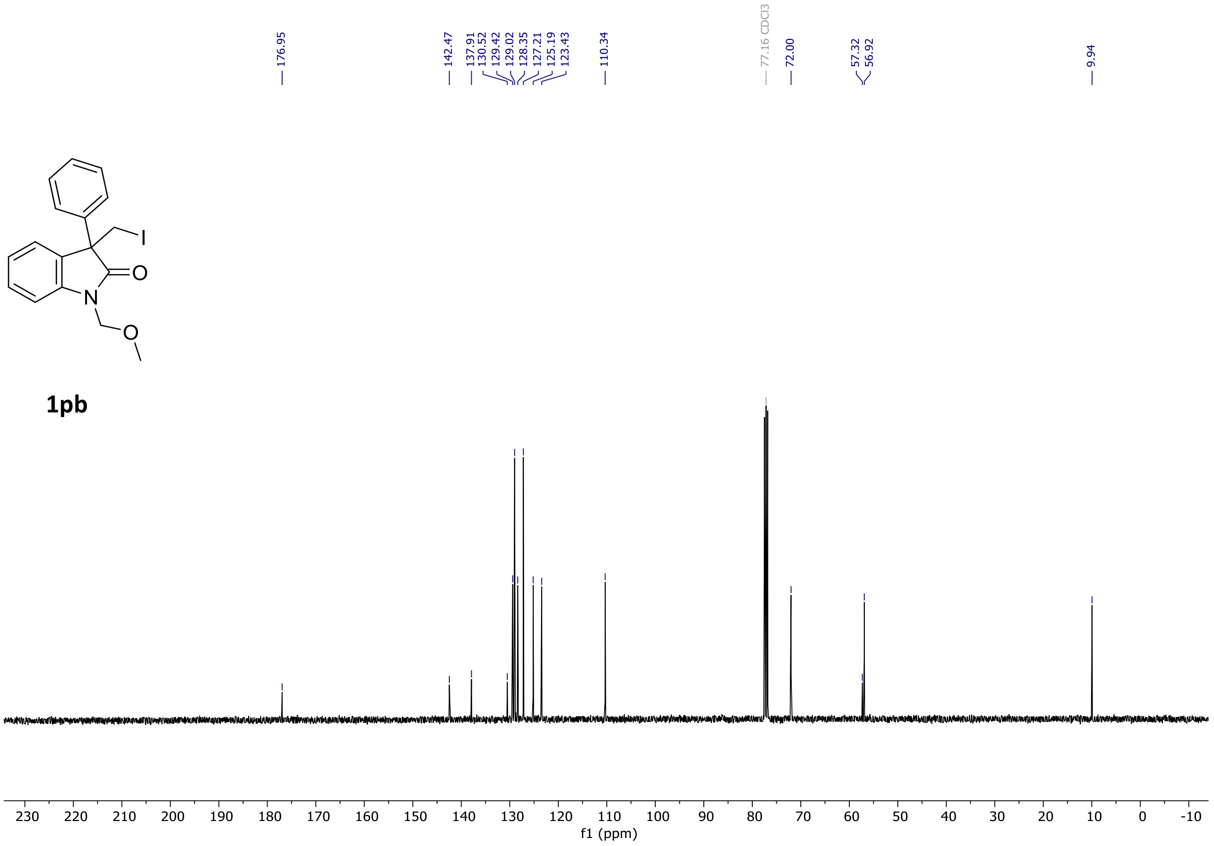
176.95
142.47 137.91 130.52 129.42 129.02 128.35 127.21 125.19 123.43
110.34
77.16 CDCl3
72.00
57.32 56.92
9.94
f1 (ppm)

1pb

1qb

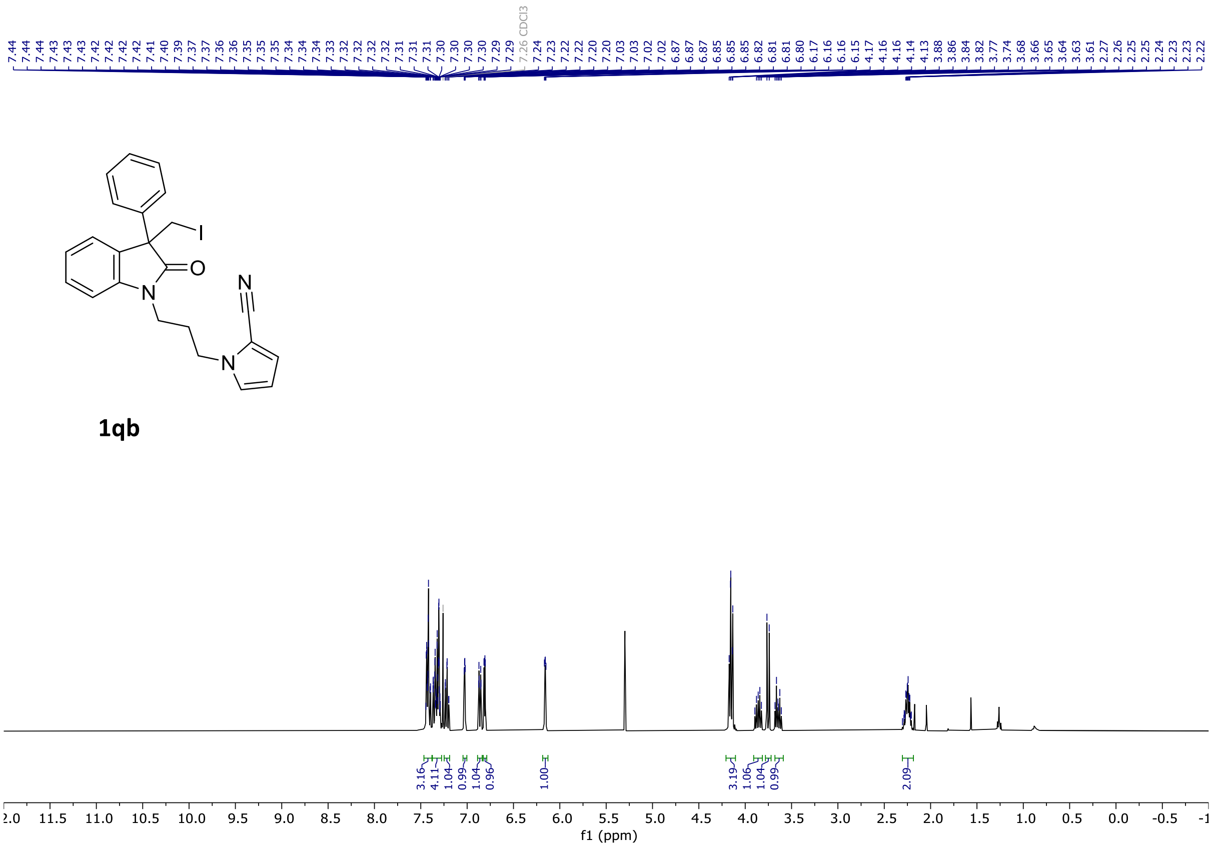

7.44 7.44 7.44 7.43 7.43 7.42 7.42 7.42 7.42 7.41 7.40 7.39 7.37 7.37 7.36 7.35 7.35 7.35 7.34 7.34 7.34 7.33 7.32 7.32 7.32 7.31 7.31 7.30 7.30 7.30 7.29 7.29 7.26 CDCl3 7.24 7.23 7.22 7.22 7.20 7.20 7.03 7.03 7.02 7.02 6.87 6.87 6.85 6.85 6.82 6.81 6.81 6.80 6.17 6.16 6.16 6.15 4.17 4.16 4.16 4.14 4.13 3.88 3.86 3.84 3.82 3.77 3.74 3.68 3.66 3.65 3.64 3.63 3.61 2.27 2.26 2.25 2.25 2.24 2.23 2.23 2.22
3.16 4.11 1.04 0.99 1.04 0.96 1.00 3.19 1.06 1.04 0.99 2.09
f1 (ppm)

1qb

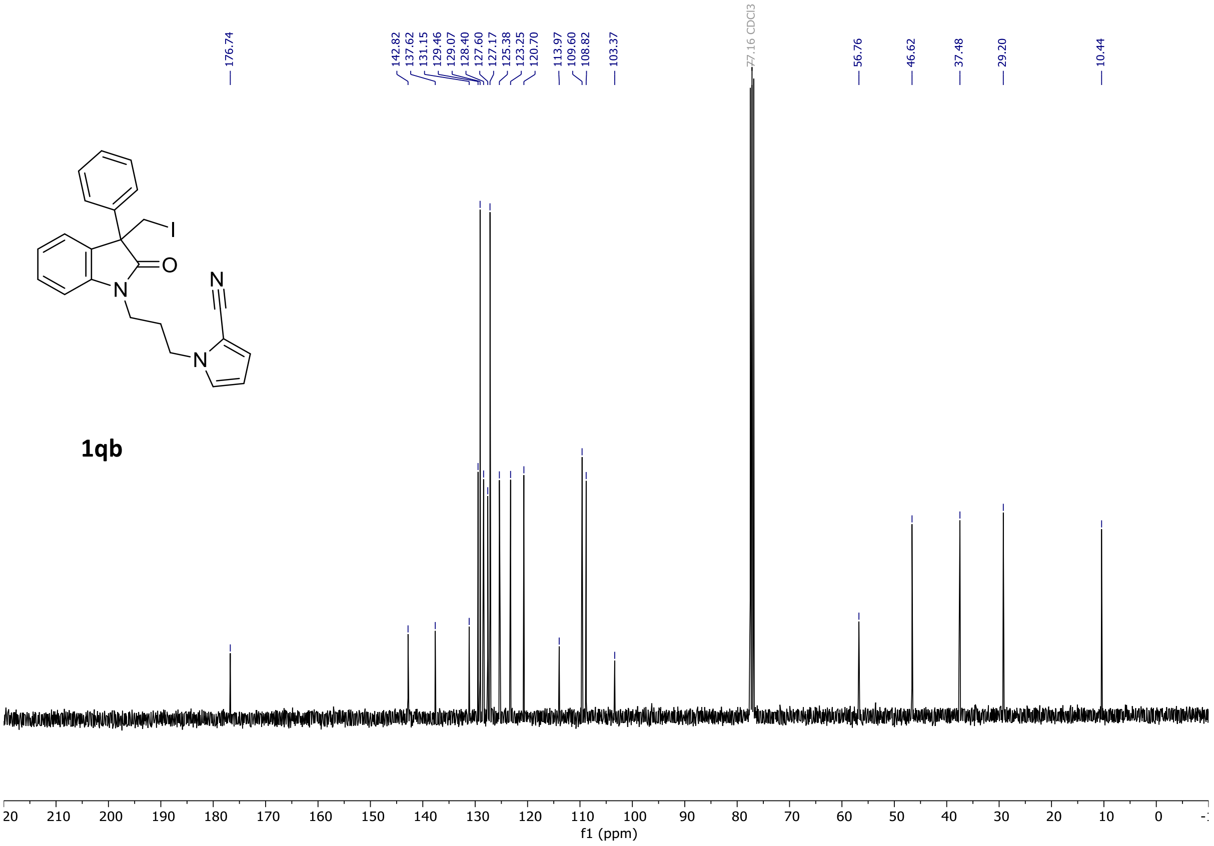

176.74 142.82 137.62 131.15 129.46 129.07 128.40 127.60 127.17 125.38 123.25 120.70 113.97 109.60 108.82 103.37 77.16 CDCl3 56.76 46.62 37.48 29.20 10.44
f1 (ppm)

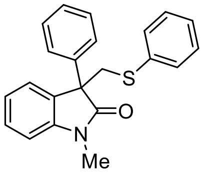

1aa

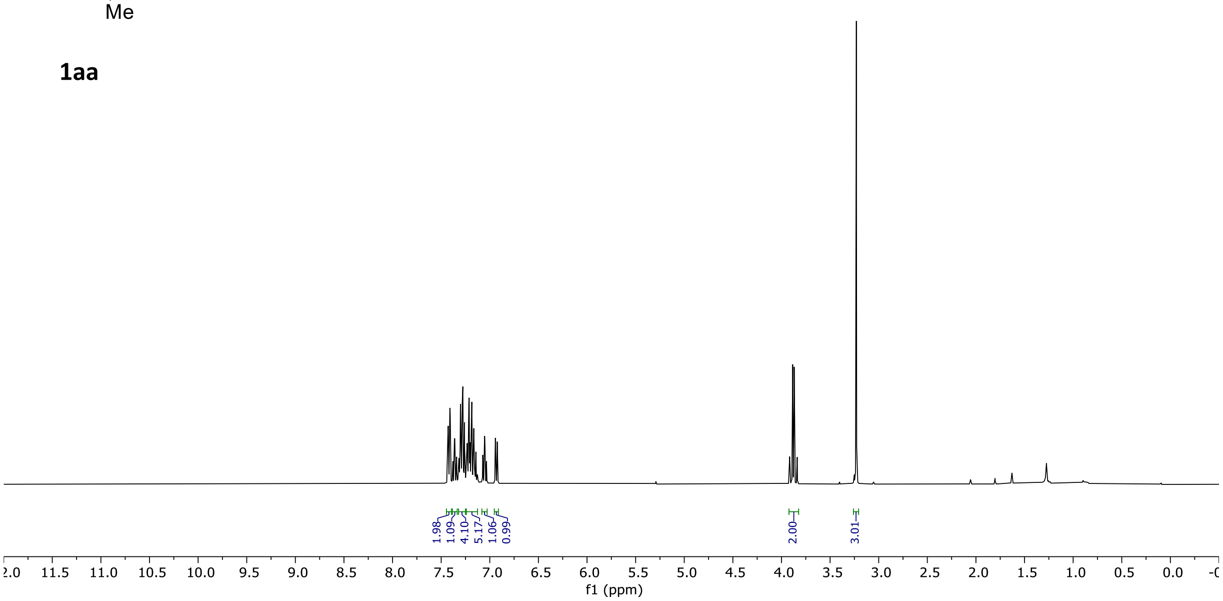
1.98
1.09
4.10
5.17
1.06
0.99
2.00
3.01
f1 (ppm)

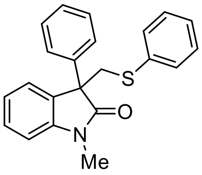

1aa

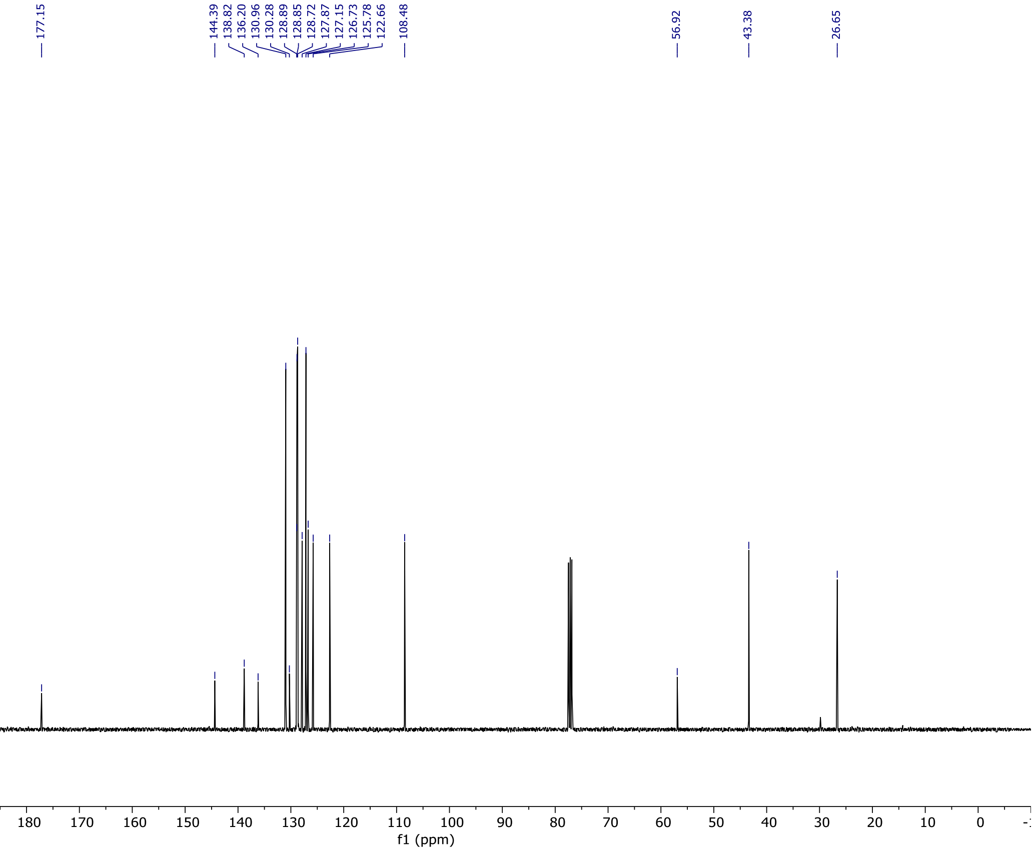
177.15
144.39
138.82
136.20
130.96
130.28
128.89
128.85
128.72
127.87
127.15
126.73
125.78
122.66
108.48
56.92
43.38
26.65
f1 (ppm)

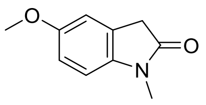

SM-B-1

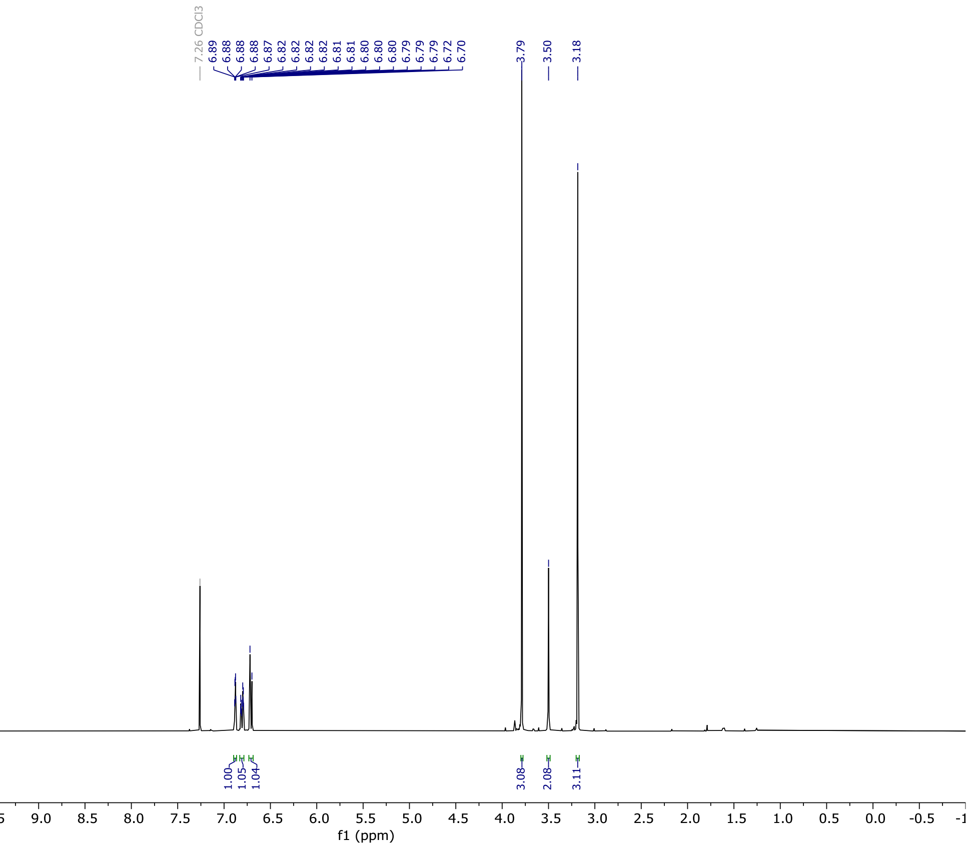
7.26 CDCl3
6.89
6.88
6.88
6.88
6.87
6.82
6.82
6.82
6.81
6.81
6.80
6.80
6.80
6.79
6.79
6.79
6.72
6.70
3.79
3.50
3.18
1.00
1.05
1.04
3.08
2.08
3.11
11.5 11.0 10.5 10.0 9.5 9.0 8.5 8.0 7.5 7.0 6.5 6.0 5.5 5.0 4.5 4.0 3.5 3.0 2.5 2.0 1.5 1.0 0.5 0.0 -0.5
f1 (ppm)

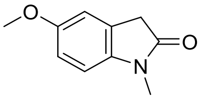

SM-B-1

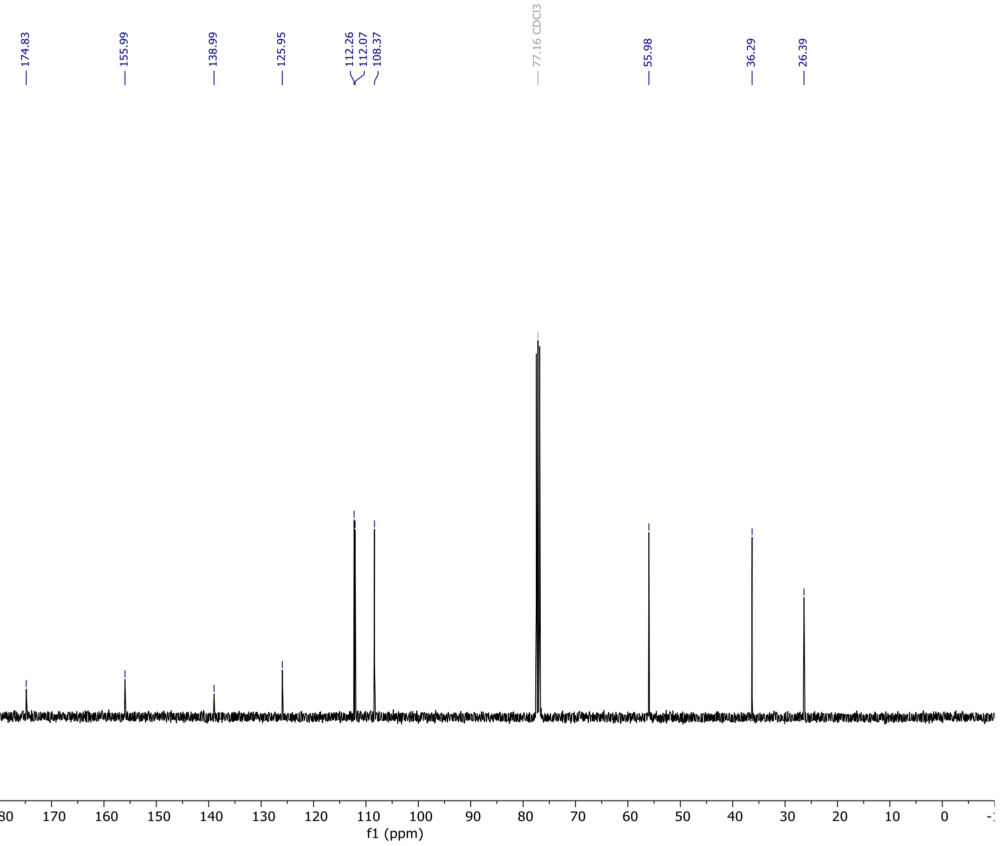
174.83
155.99
138.99
125.95
112.26
112.07
108.37
77.16 CDCl3
55.98
36.29
26.39
210 200 190 180 170 160 150 140 130 120 110 100 90 80 70 60 50 40 30 20 10 0
f1 (ppm)

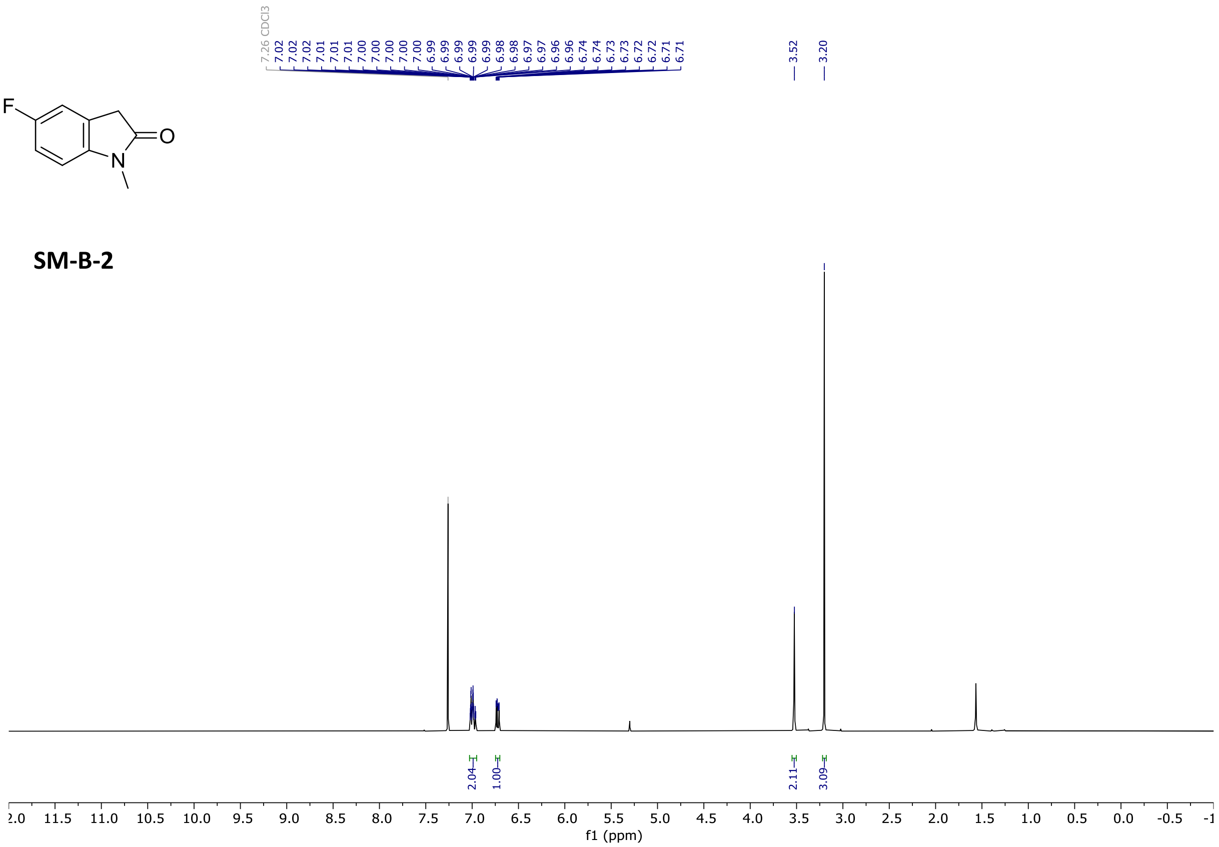
SM-B-2
7.26 CDCl3
7.02 7.02 7.02 7.01 7.01 7.01 7.00 7.00 7.00 7.00 6.99 6.99 6.99 6.99 6.98 6.98 6.97 6.96 6.96 6.74 6.74 6.73 6.73 6.72 6.72 6.71 6.71
3.52
3.20
2.04
1.00
2.11
3.09
f1 (ppm)

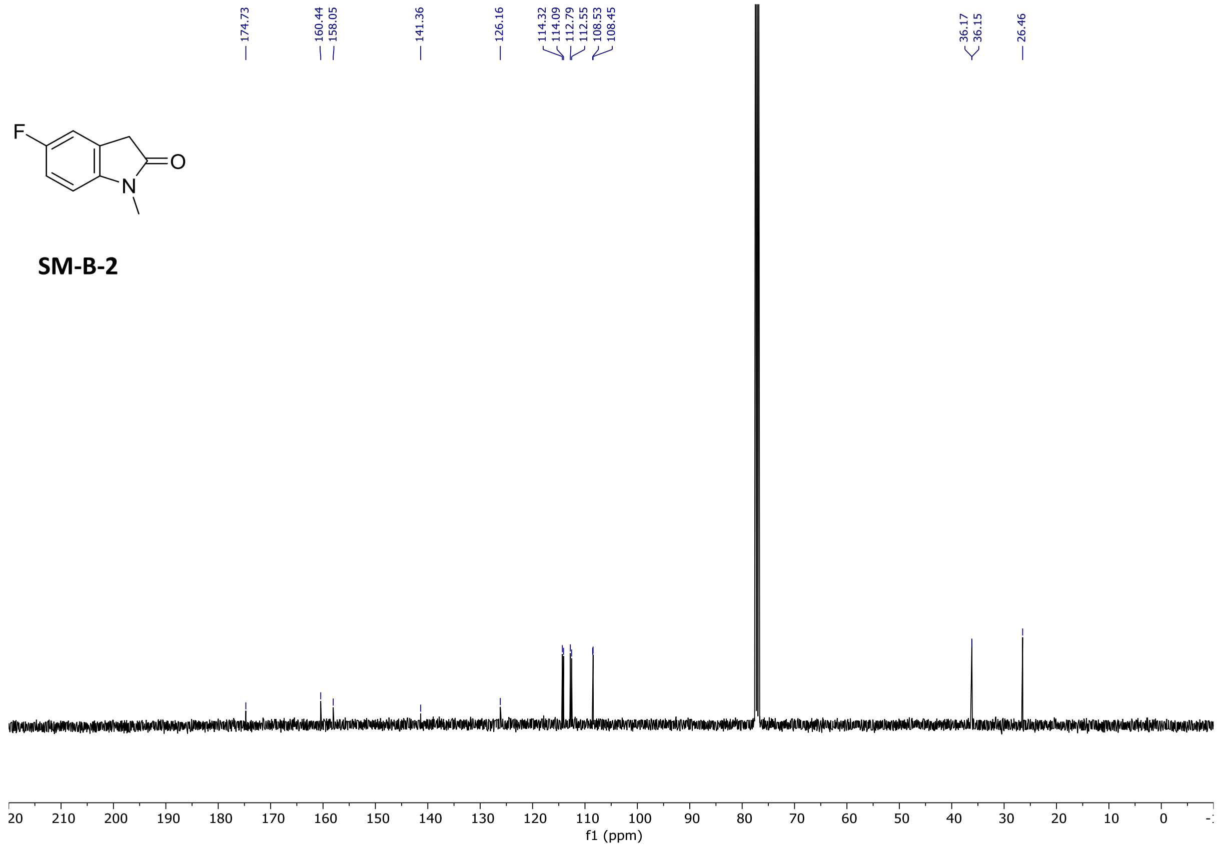
SM-B-2
174.73
160.44
158.05
141.36
126.16
114.32
114.09
112.79
112.55
108.53
108.45
36.17
36.15
26.46
f1 (ppm)

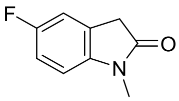

SM-B-2

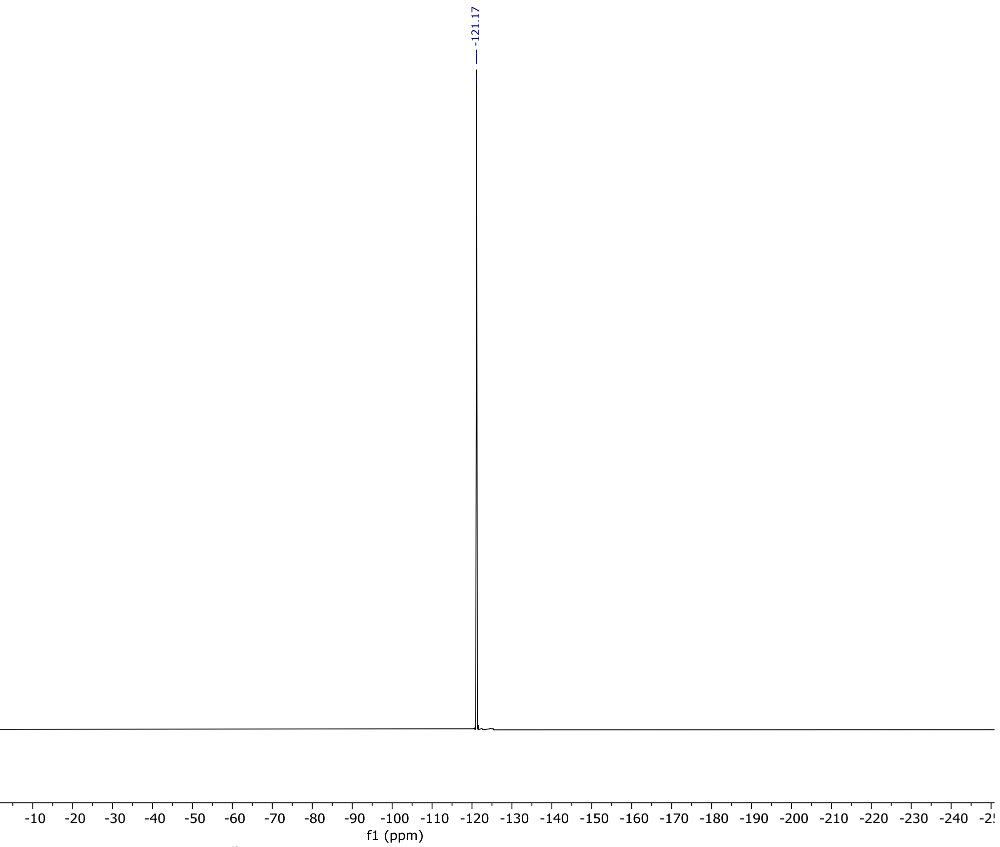

-121.17
50
40
30
20
10
0
-10
-20
-30
-40
-50
-60
-70
-80
-90
-100
-110
-120
-130
-140
-150
-160
-170
-180
-190
-200
-210
-220
-230
-240
f1 (ppm)

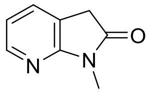

SM-B-3

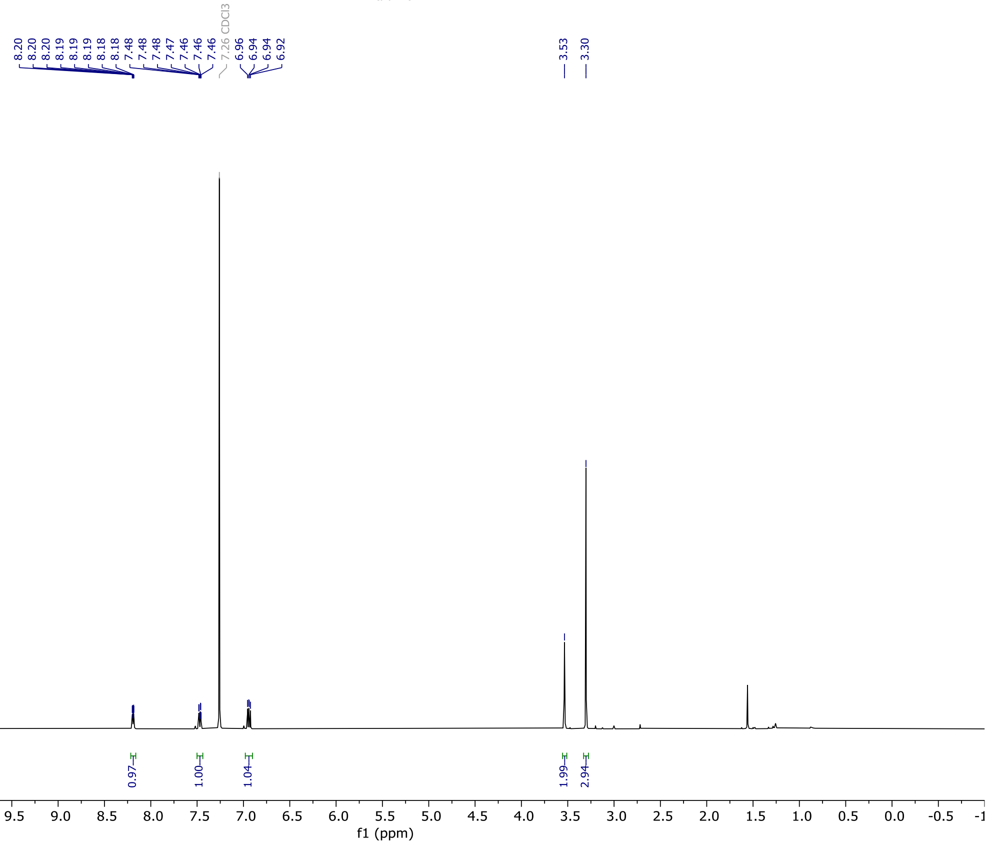

8.20
8.20
8.20
8.19
8.19
8.19
8.18
8.18
7.48
7.48
7.48
7.47
7.46
7.46
7.46
7.26 CDCl3
6.96
6.94
6.94
6.92
3.53
3.30
0.97
1.00
1.04
1.99
2.94
11.5
11.0
10.5
10.0
9.5
9.0
8.5
8.0
7.5
7.0
6.5
6.0
5.5
5.0
4.5
4.0
3.5
3.0
2.5
2.0
1.5
1.0
0.5
0.0
-0.5
f1 (ppm)

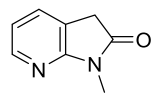

SM-B-3

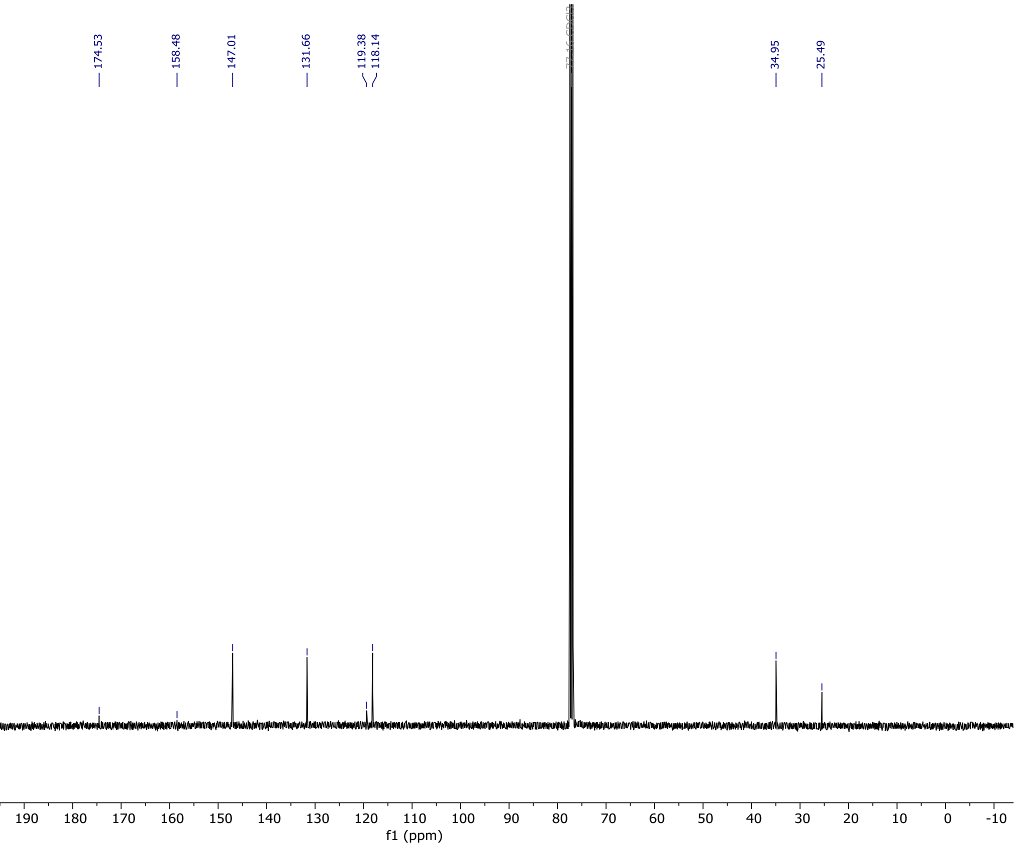
174.53
158.48
147.01
131.66
119.38
118.14
34.95
25.49
230 220 210 200 190 180 170 160 150 140 130 120 110 100 90 80 70 60 50 40 30 20 10 0 -10
f1 (ppm)

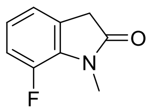

SM-B-4

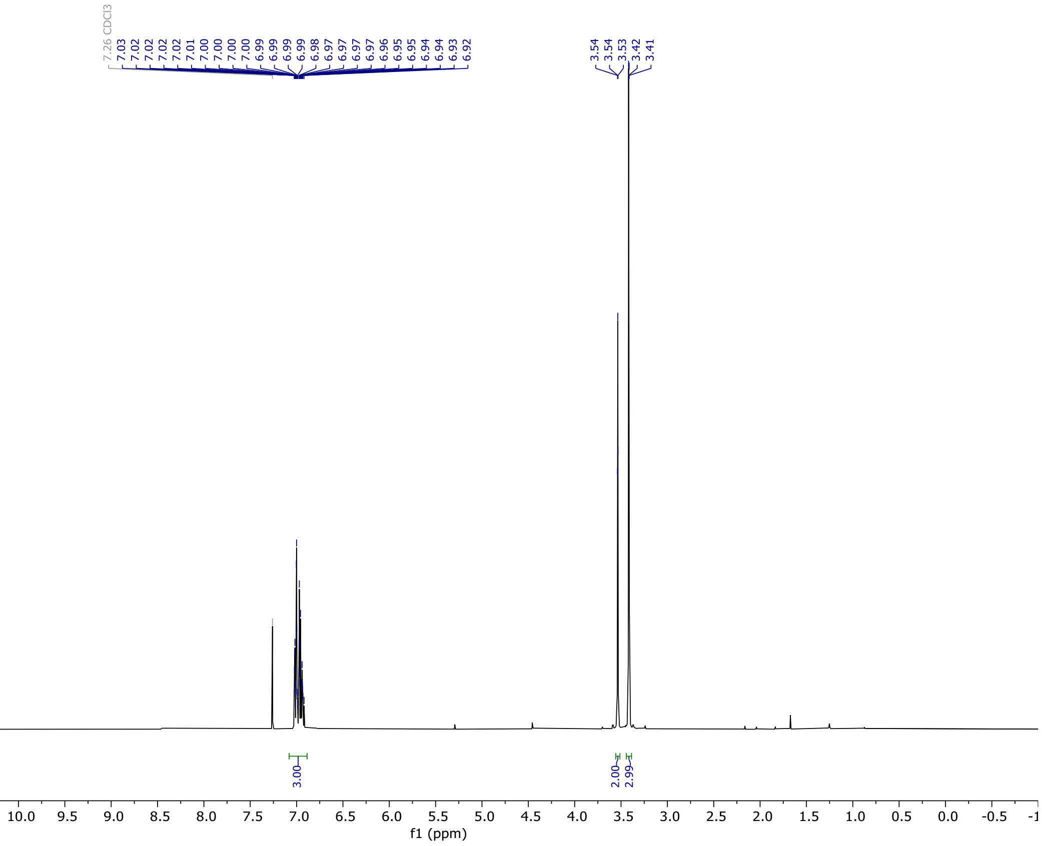
7.26 CDCl3
7.03 7.02 7.02 7.02 7.01 7.00 7.00 7.00 6.99 6.99 6.99 6.98 6.97 6.97 6.97 6.96 6.95 6.95 6.94 6.94 6.93 6.92
3.54 3.54 3.53 3.42 3.41
3.00
2.00
2.99
11.5 11.0 10.5 10.0 9.5 9.0 8.5 8.0 7.5 7.0 6.5 6.0 5.5 5.0 4.5 4.0 3.5 3.0 2.5 2.0 1.5 1.0 0.5 0.0 -0.5
f1 (ppm)

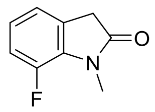
O
N
F

SM-B-4

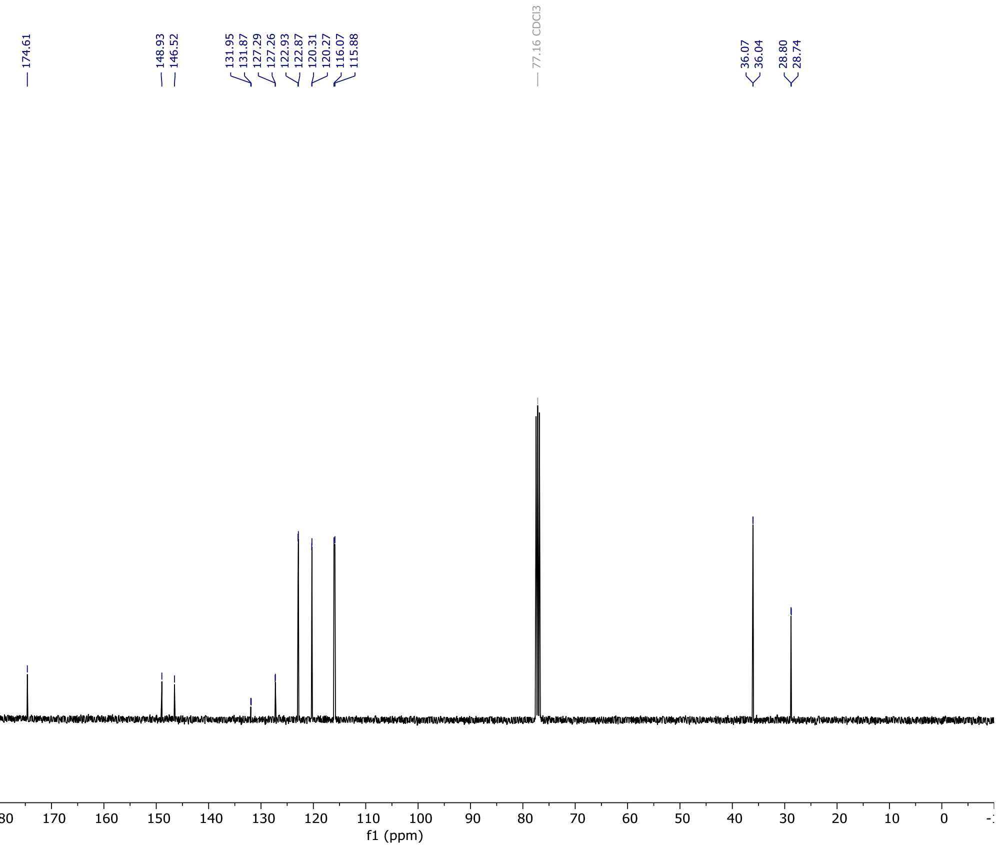
174.61
148.93
146.52
131.95
131.87
127.29
127.26
122.93
122.87
120.31
120.27
116.07
115.88
77.16 CDCl3
36.07
36.04
28.80
28.74
f1 (ppm)

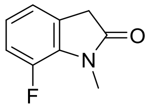
O
N
F

SM-B-4

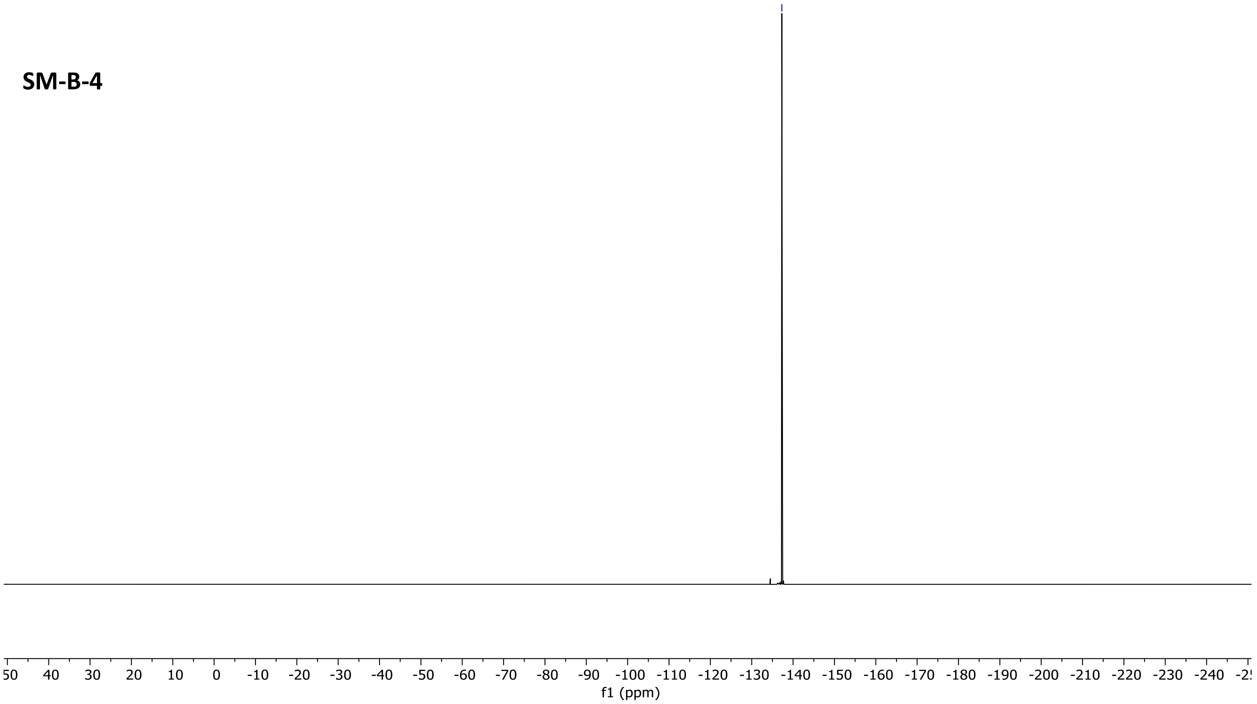
-137.28
f1 (ppm)

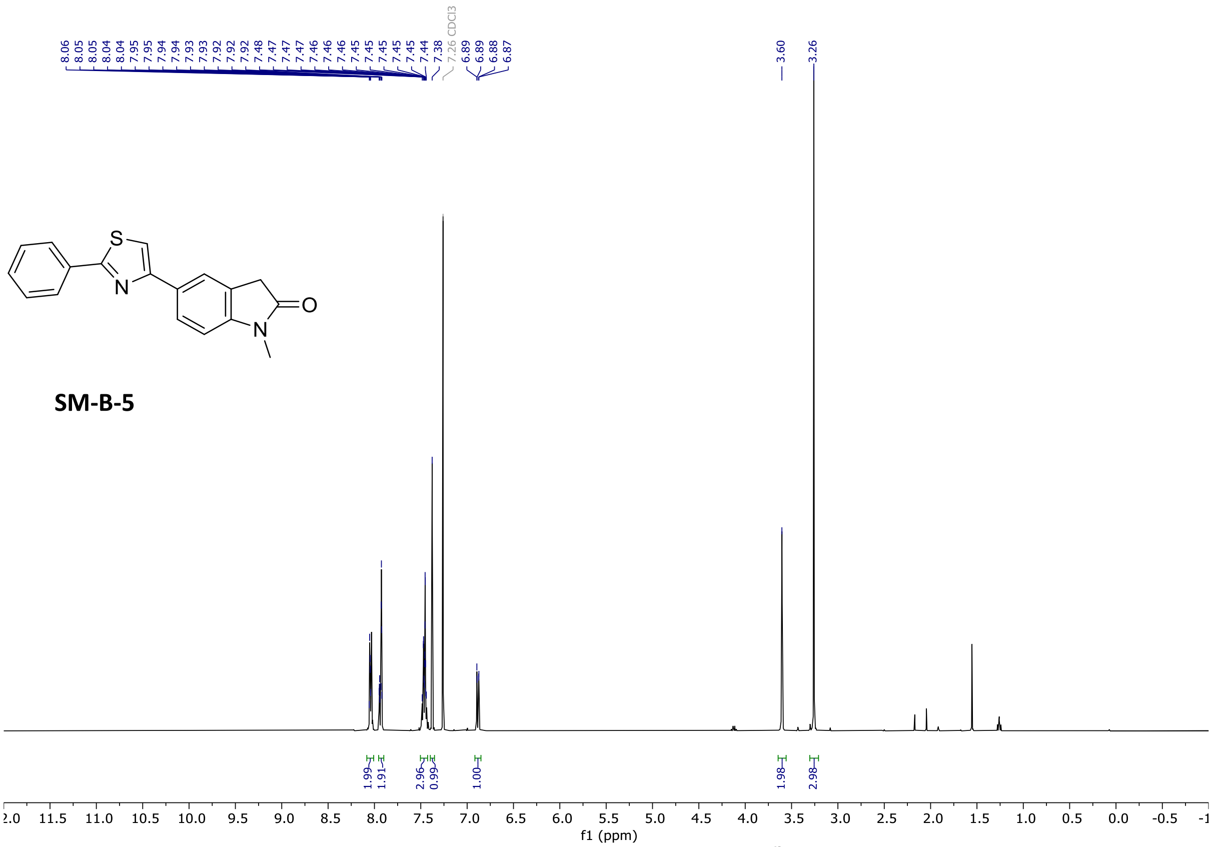
8.06 8.05 8.05 8.04 7.95 7.95 7.94 7.93 7.93 7.92 7.92 7.48 7.47 7.47 7.46 7.46 7.45 7.45 7.45 7.45 7.44 7.38
7.26 CDCl3
6.89 6.89 6.88 6.87
3.60
3.26
S
N
N
O
1.99 1.91 2.96 0.99 1.00 1.98 2.98
2.0 11.5 11.0 10.5 10.0 9.5 9.0 8.5 8.0 7.5 7.0 6.5 6.0 5.5 5.0 4.5 4.0 3.5 3.0 2.5 2.0 1.5 1.0 0.5 0.0 -0.5 -1
f1 (ppm)

SM-B-5

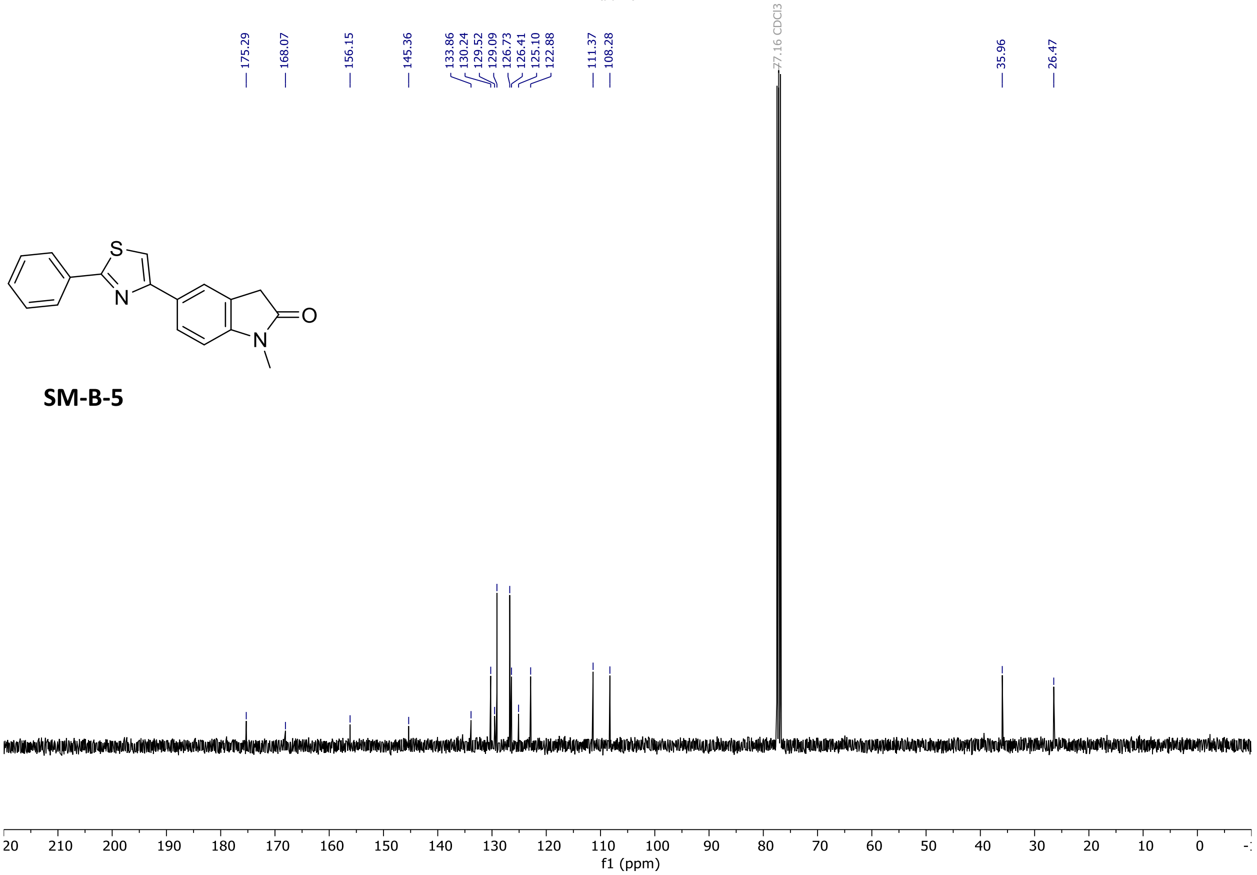
175.29
168.07
156.15
145.36
133.86 130.24 129.52 129.09 126.73 126.41 125.10 122.88
111.37
108.28
77.16 CDCl3
35.96
26.47
S
N
N
O
20 210 200 190 180 170 160 150 140 130 120 110 100 90 80 70 60 50 40 30 20 10 0
f1 (ppm)

SM-B-5

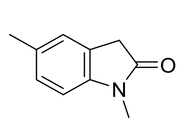

SM-6

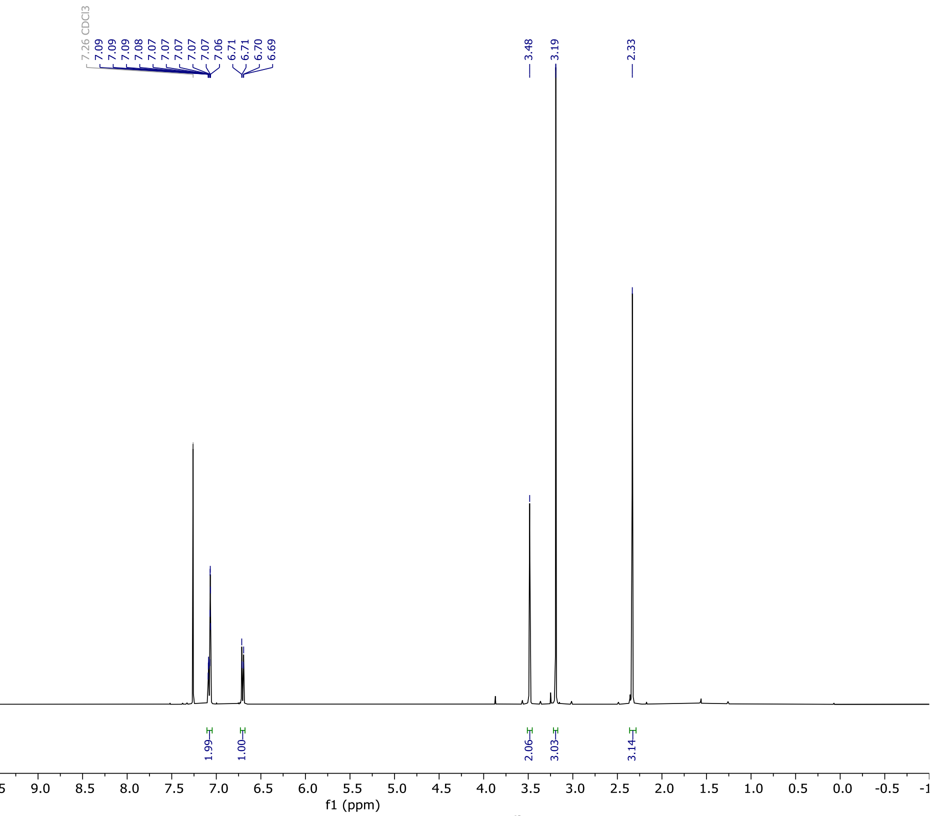

7.26 CDCl3
7.09
7.09
7.09
7.08
7.07
7.07
7.07
7.07
7.07
7.06
6.71
6.71
6.70
6.69
3.48
3.19
2.33
1.99
1.00
2.06
3.03
3.14
f1 (ppm)

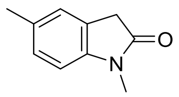

SM-6

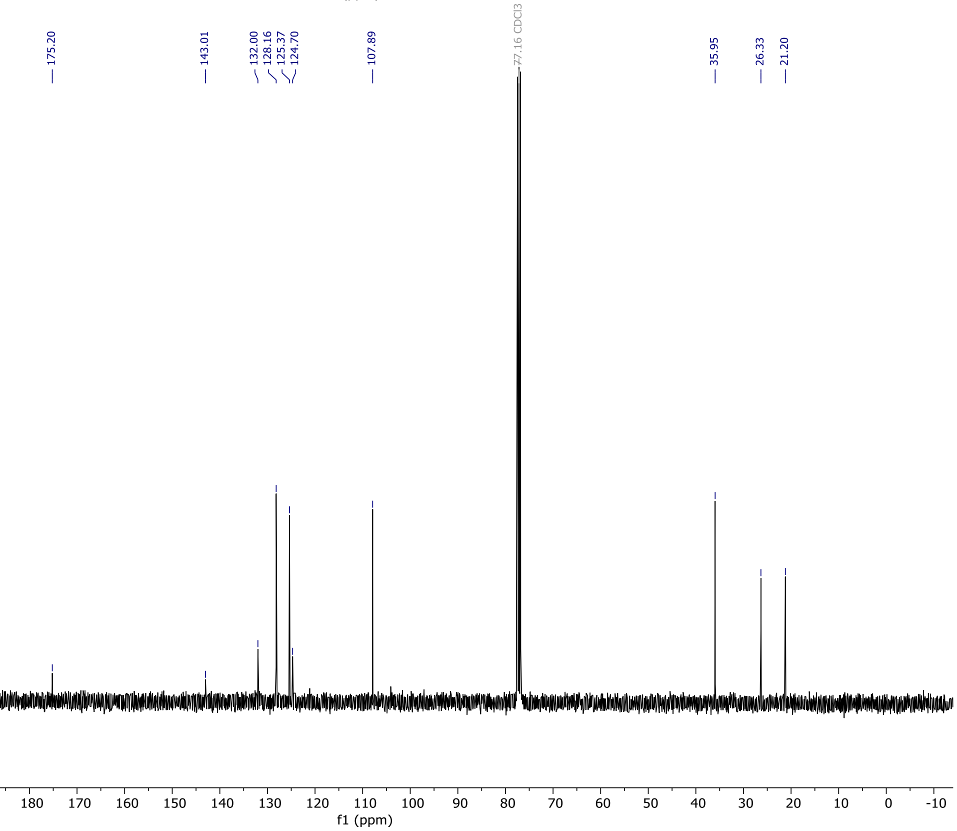

175.20
143.01
132.00
128.16
125.37
124.70
107.89
77.16 CDCl3
35.95
26.33
21.20
f1 (ppm)

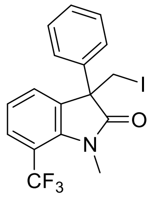

1fb

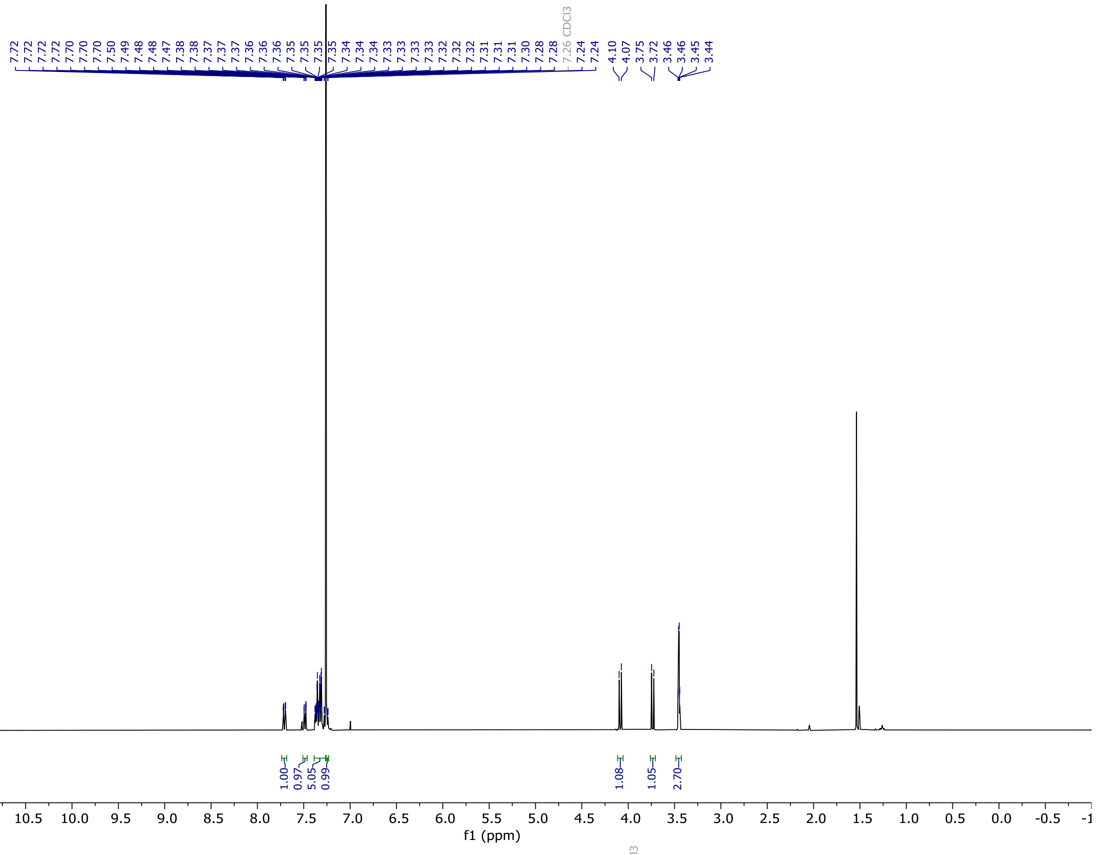

7.72 7.72 7.72 7.72 7.70 7.70 7.50 7.49 7.48 7.47 7.38 7.38 7.37 7.37 7.36 7.36 7.35 7.35 7.35 7.35 7.34 7.34 7.34 7.33 7.33 7.33 7.33 7.32 7.32 7.32 7.31 7.31 7.31 7.30 7.28 7.26 CDCl3 7.24 7.24
4.10 4.07 3.75 3.72 3.46 3.45 3.44
1.00 0.97 5.05 0.99 1.08 1.05 2.70
f1 (ppm)

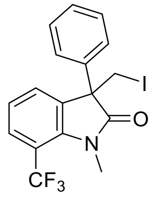

1fb

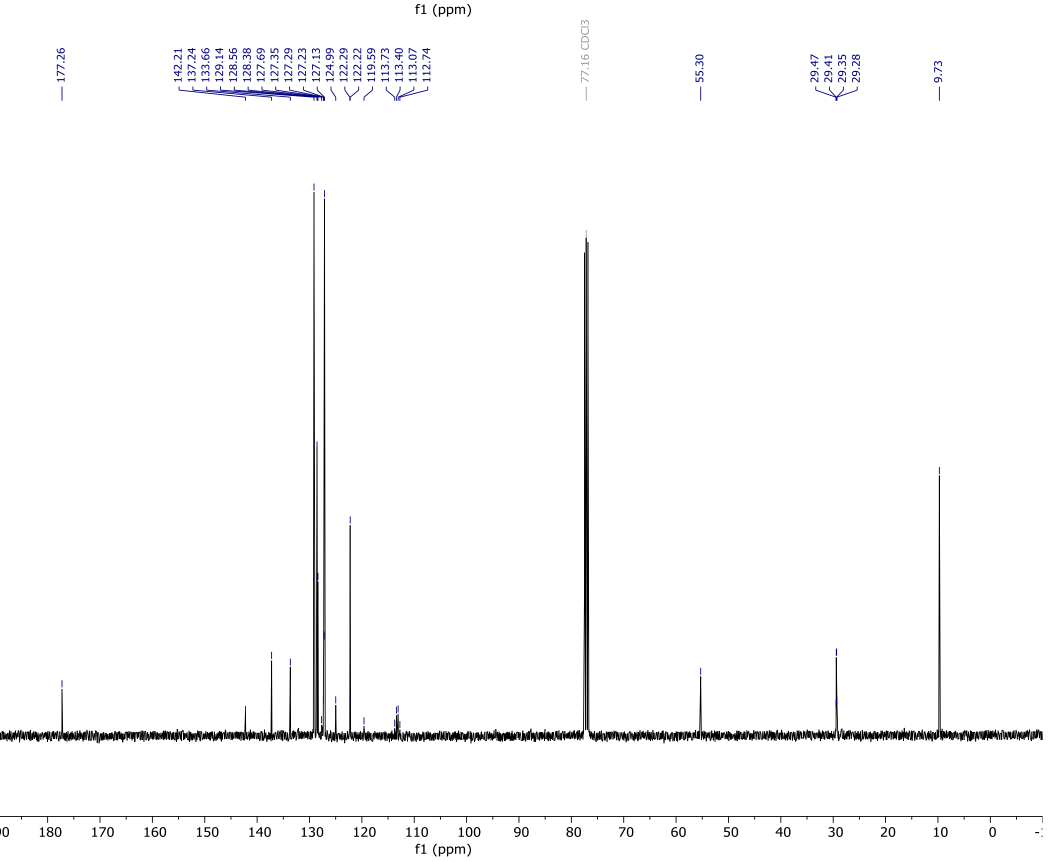

177.26
142.21 137.24 133.66 129.14 128.56 128.38 127.69 127.35 127.29 127.23 127.13 124.99 122.29 122.22 119.59 113.73 113.40 113.07 112.74
77.16 CDCl3
55.30
29.47 29.41 29.35 29.28
9.73
f1 (ppm)

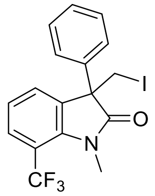

1fb

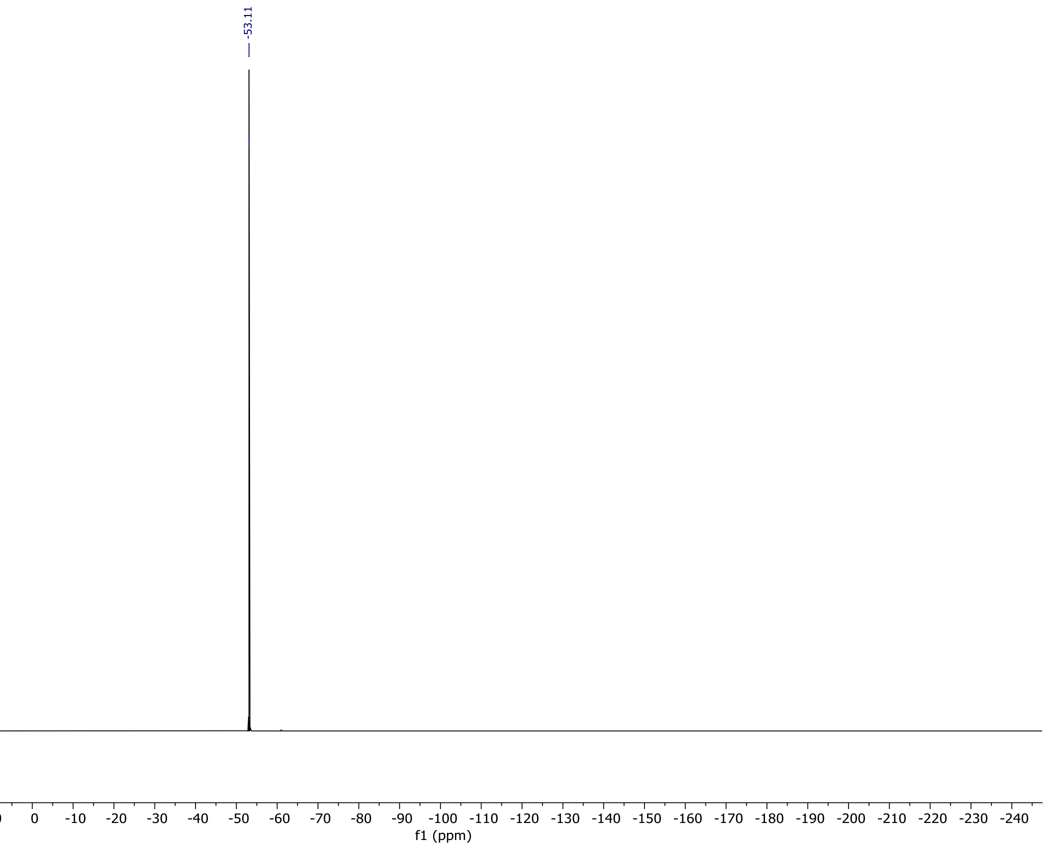

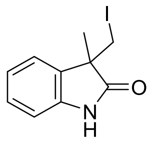

1rb

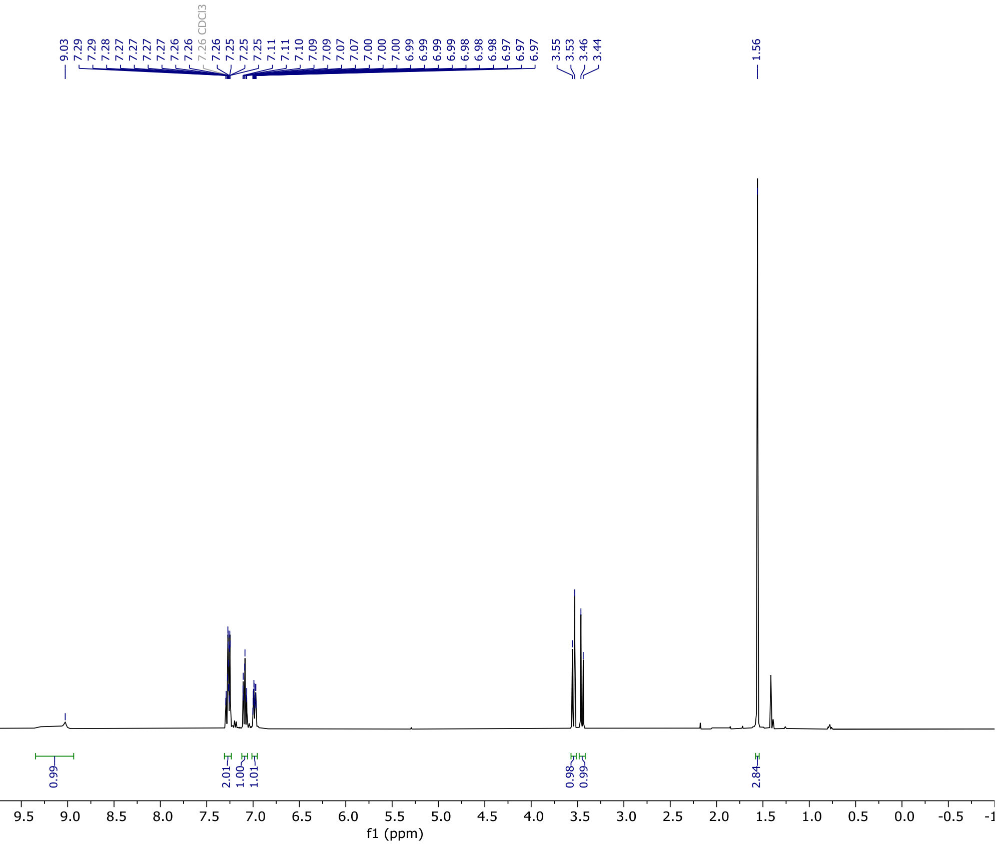

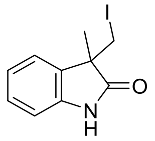
I
O
N
H

1rb

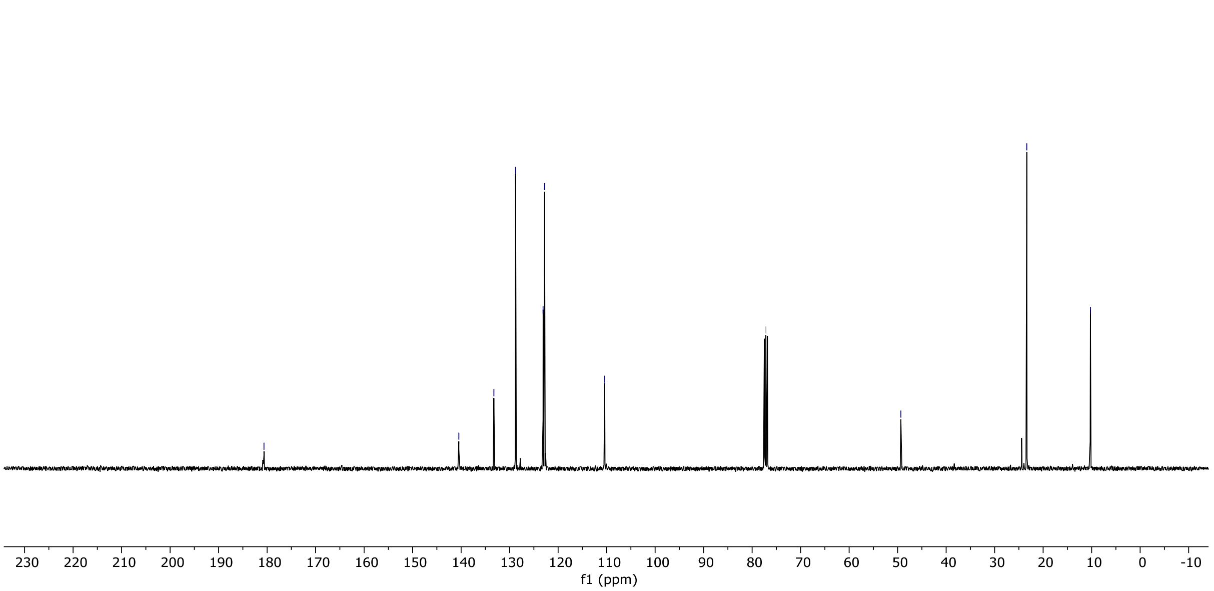
180.65
140.47
133.24
128.77
123.10
122.80
110.39
77.16 CDCl3
49.33
23.37
10.26
230
220
210
200
190
180
170
160
150
140
130
120
110
100
90
80
70
60
50
40
30
20
10
0
-10
f1 (ppm)

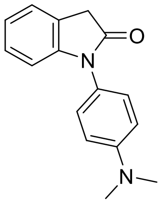

SM-7

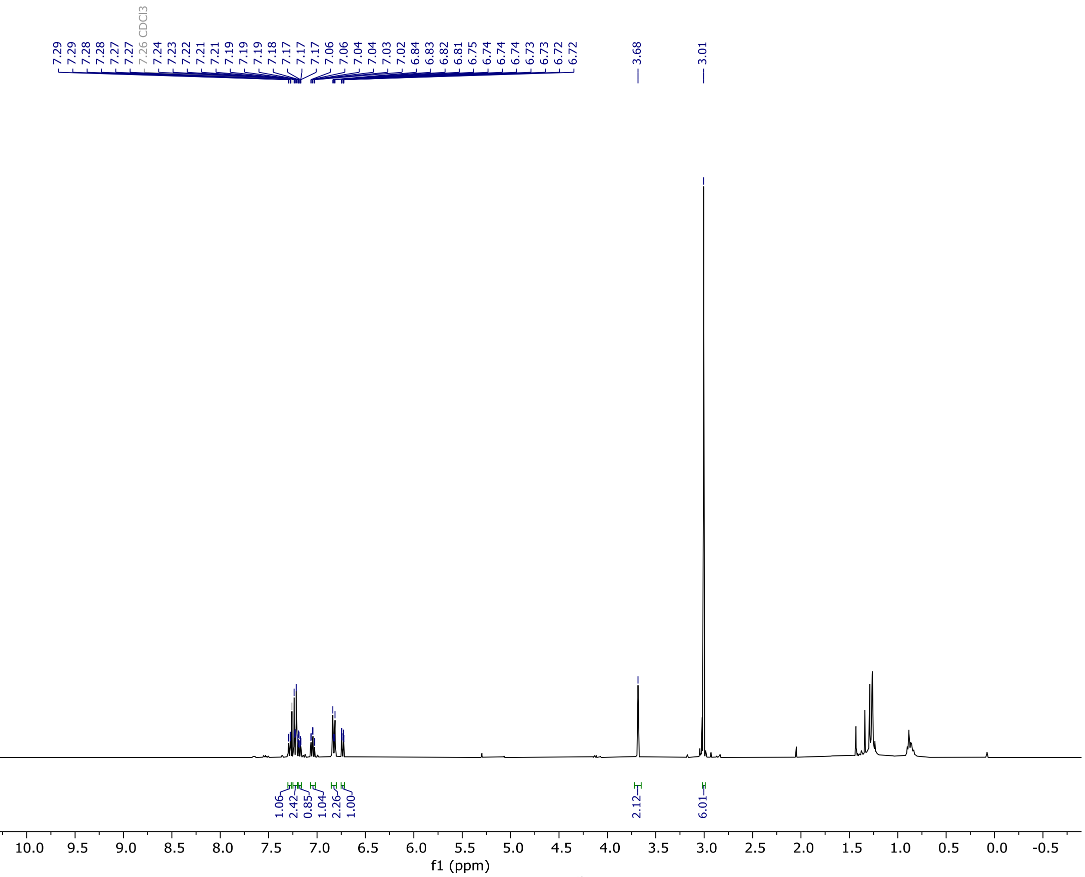
7.29 7.29 7.28 7.28 7.27 7.27 7.26 CDCl3 7.24 7.23 7.22 7.21 7.21 7.19 7.19 7.18 7.17 7.17 7.17 7.06 7.06 7.04 7.04 7.03 7.02 6.84 6.83 6.82 6.81 6.75 6.74 6.74 6.73 6.73 6.72 6.72
3.68
3.01
1.06 2.42 0.85 1.04 2.26 1.00
2.12
6.01
f1 (ppm)

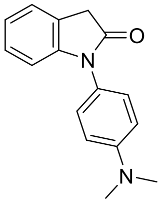

SM-7

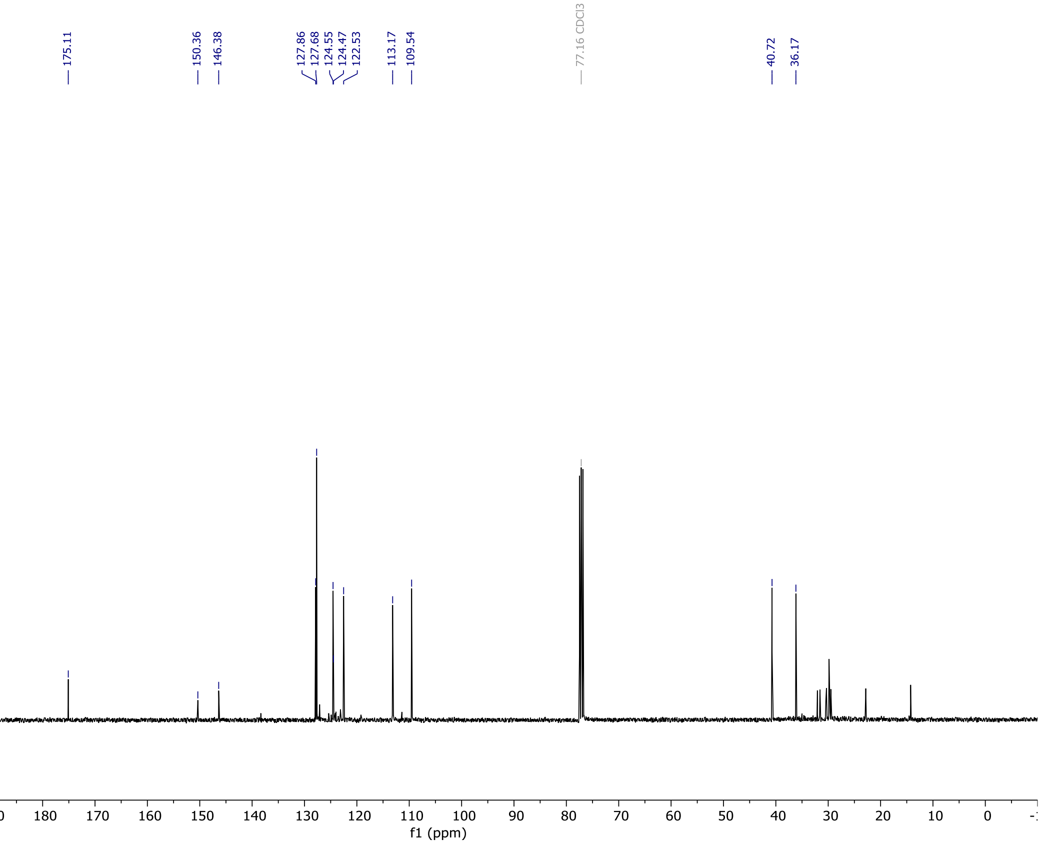
175.11
150.36 146.38
127.86 127.68 124.55 124.47 122.53
113.17 109.54
77.16 CDCl3
40.72 36.17
f1 (ppm)

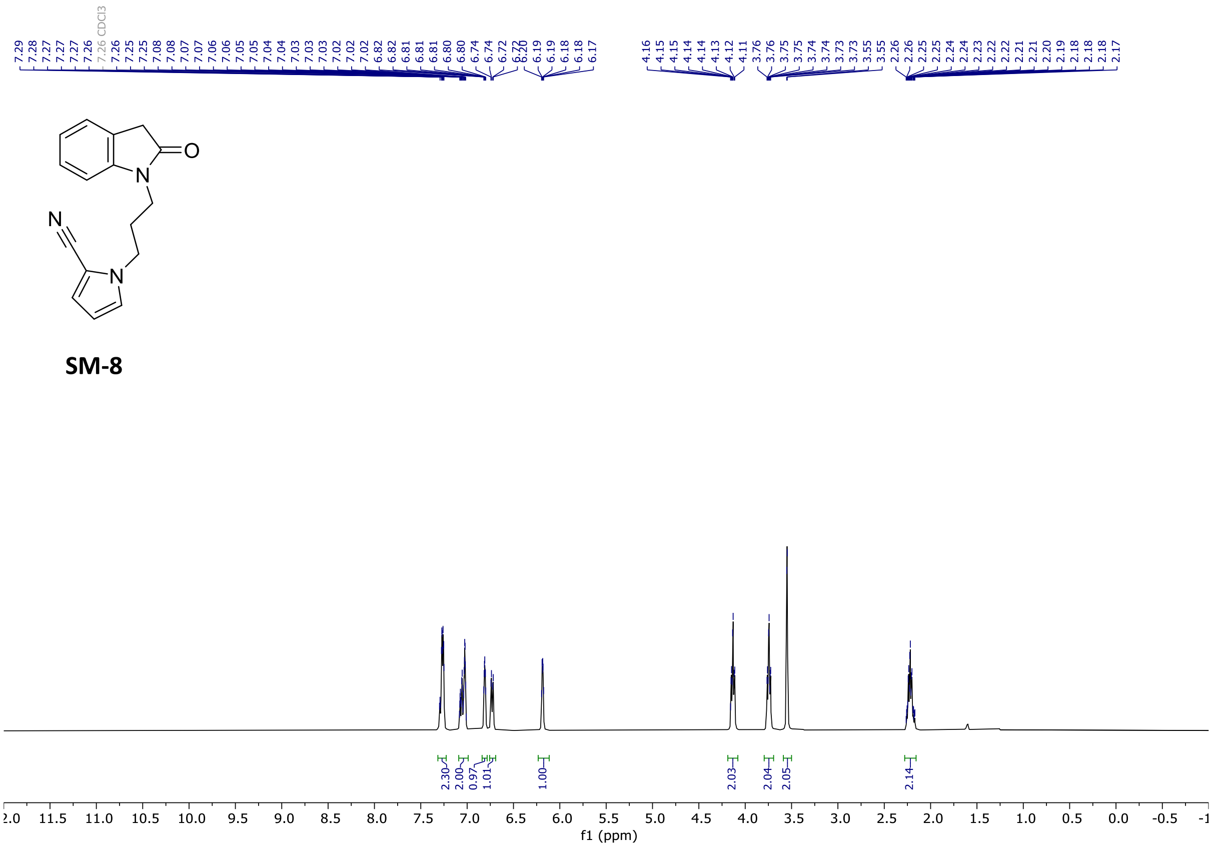

7.29 7.28 7.27 7.27 7.26 7.26 CDCl3 7.26 7.25 7.25 7.08 7.08 7.07 7.07 7.06 7.05 7.05 7.04 7.04 7.03 7.03 7.02 7.02 7.02 6.82 6.82 6.81 6.81 6.80 6.80 6.74 6.74 6.72 6.72 6.20 6.19 6.19 6.18 6.18 6.17
4.16 4.15 4.15 4.14 4.14 4.13 4.12 4.11 3.76 3.76 3.75 3.75 3.74 3.74 3.73 3.73 3.55 3.55 2.26 2.26 2.25 2.25 2.24 2.24 2.23 2.22 2.22 2.21 2.21 2.20 2.19 2.18 2.18 2.18 2.17
N
O
SM-8
2.30 2.00 0.97 1.01 1.00 2.03 2.04 2.05 2.14
f1 (ppm)

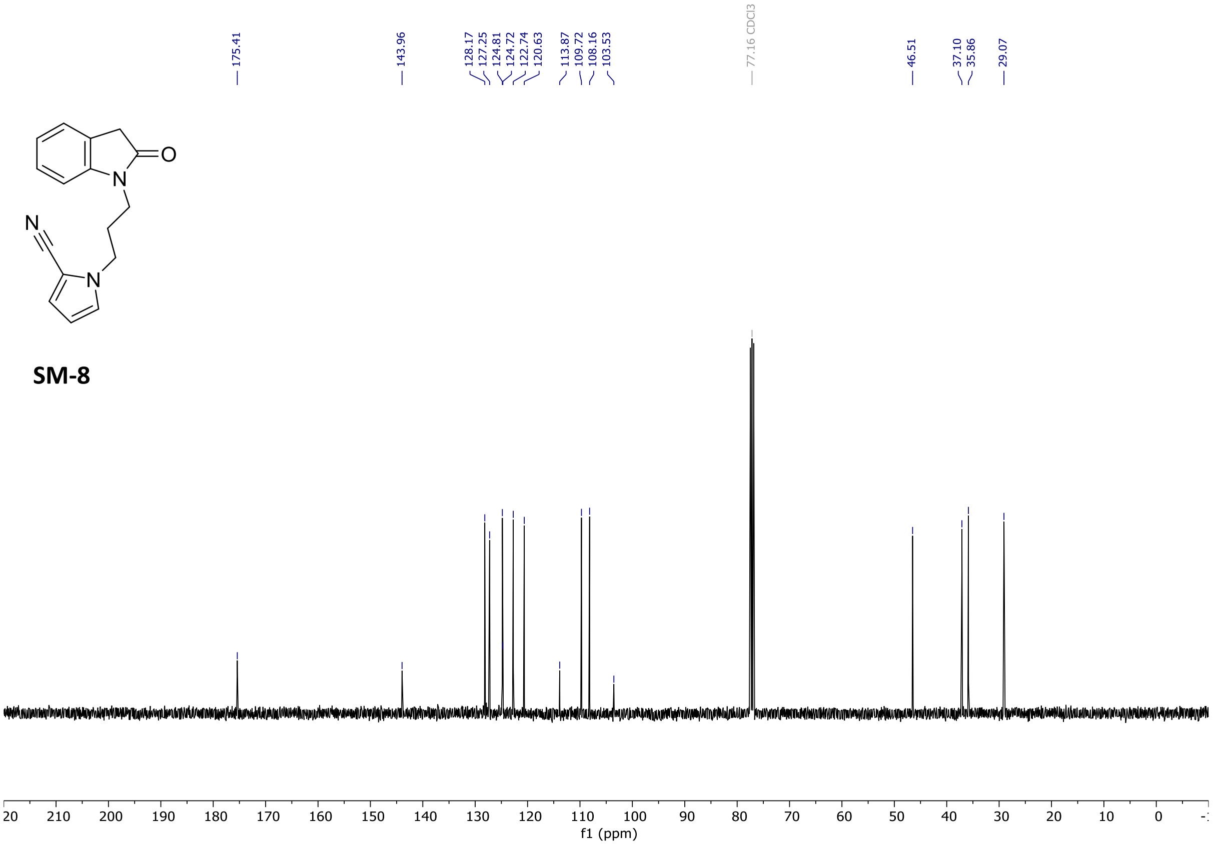

175.41 143.96 128.17 127.25 124.81 124.72 122.74 120.63 113.87 109.72 108.16 103.53 77.16 CDCl3 46.51 37.10 35.86 29.07
N
O
SM-8
f1 (ppm)

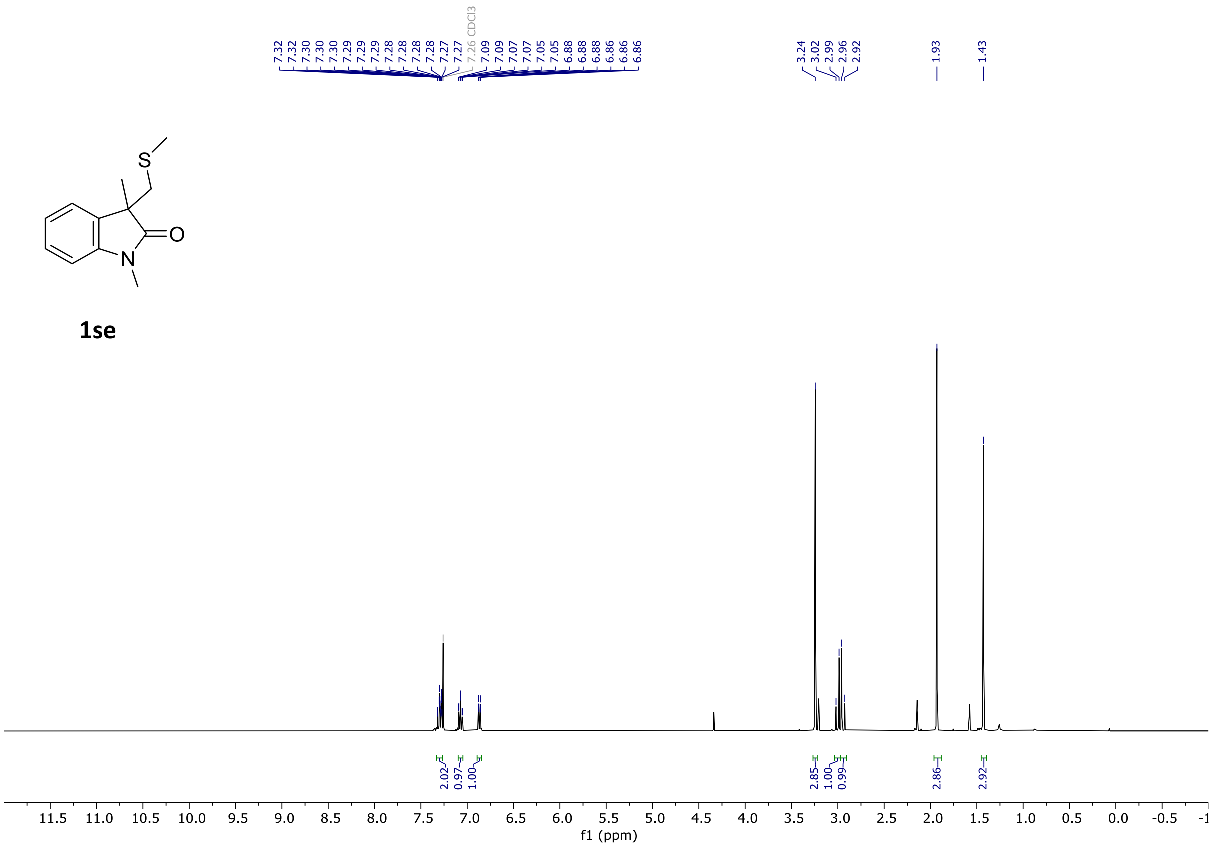
7.32 7.32 7.30 7.30 7.30 7.29 7.29 7.28 7.28 7.28 7.27 7.27 7.26 CDCl3 7.09 7.09 7.07 7.07 7.05 7.05 6.88 6.88 6.86 6.86 6.86
3.24 3.02 2.99 2.96 2.92
1.93
1.43
1se
2.02 0.97 1.00
2.85 1.00 0.99
2.86
2.92
f1 (ppm)

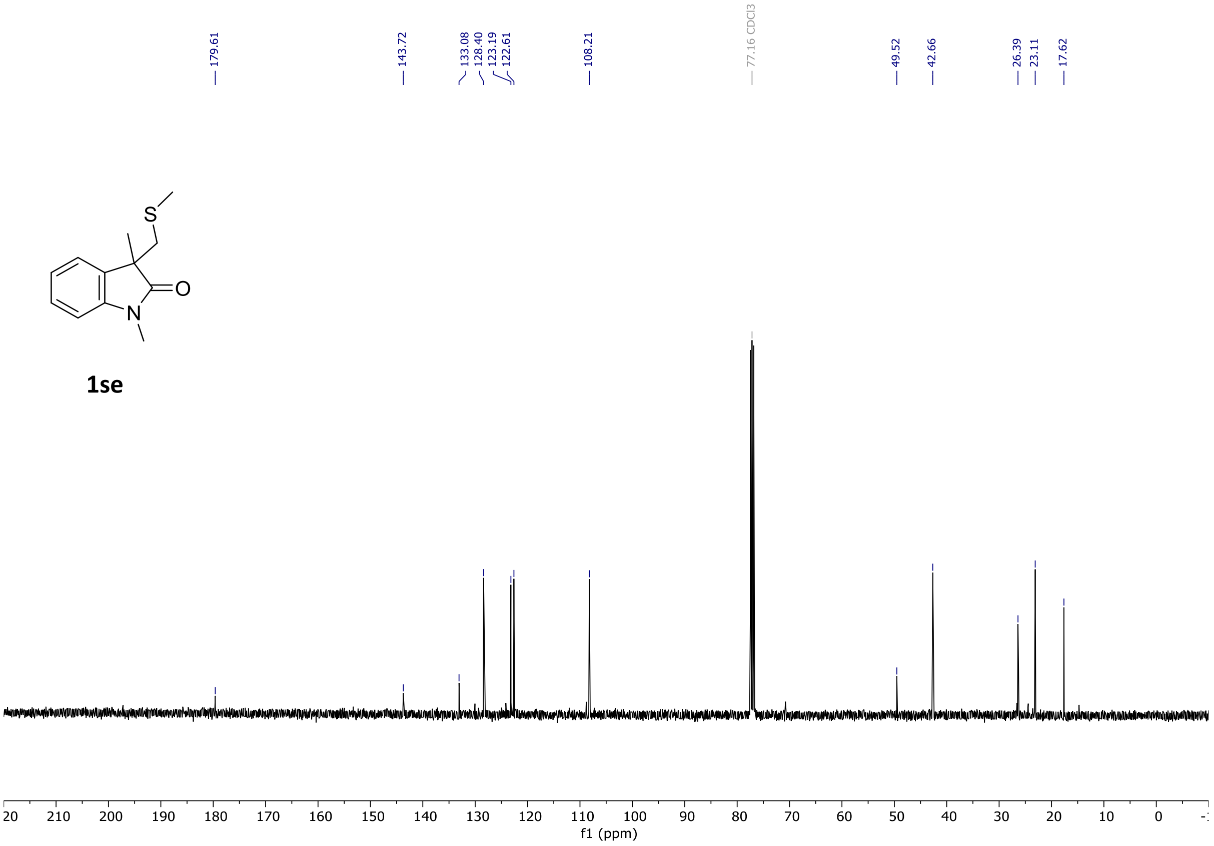
179.61
143.72
133.08 128.40 123.19 122.61
108.21
77.16 CDCl3
49.52
42.66
26.39 23.11 17.62
1se
f1 (ppm)

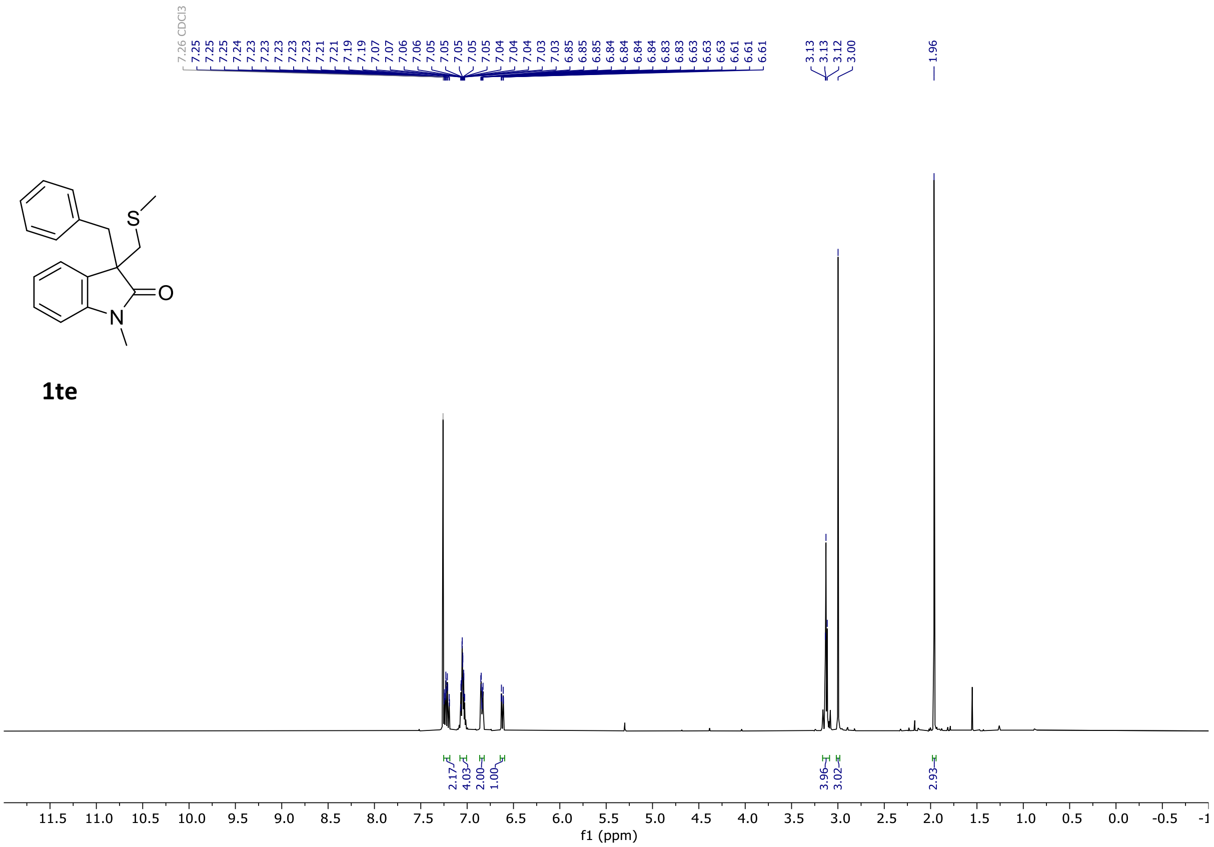
7.26 CDCl3
7.25 7.25 7.25 7.24 7.23 7.23 7.23 7.23 7.23 7.21 7.21 7.19 7.19 7.07 7.06 7.06 7.05 7.05 7.05 7.05 7.05 7.04 7.04 7.04 7.03 7.03 6.85 6.85 6.85 6.84 6.84 6.84 6.84 6.83 6.83 6.63 6.63 6.63 6.61 6.61 6.61
3.13 3.13 3.12 3.00
1.96
S
O
N
1te
2.17 4.03 2.00 1.00
3.96 3.02
2.93
11.5 11.0 10.5 10.0 9.5 9.0 8.5 8.0 7.5 7.0 6.5 6.0 5.5 5.0 4.5 4.0 3.5 3.0 2.5 2.0 1.5 1.0 0.5 0.0 -0.5 -1
f1 (ppm)

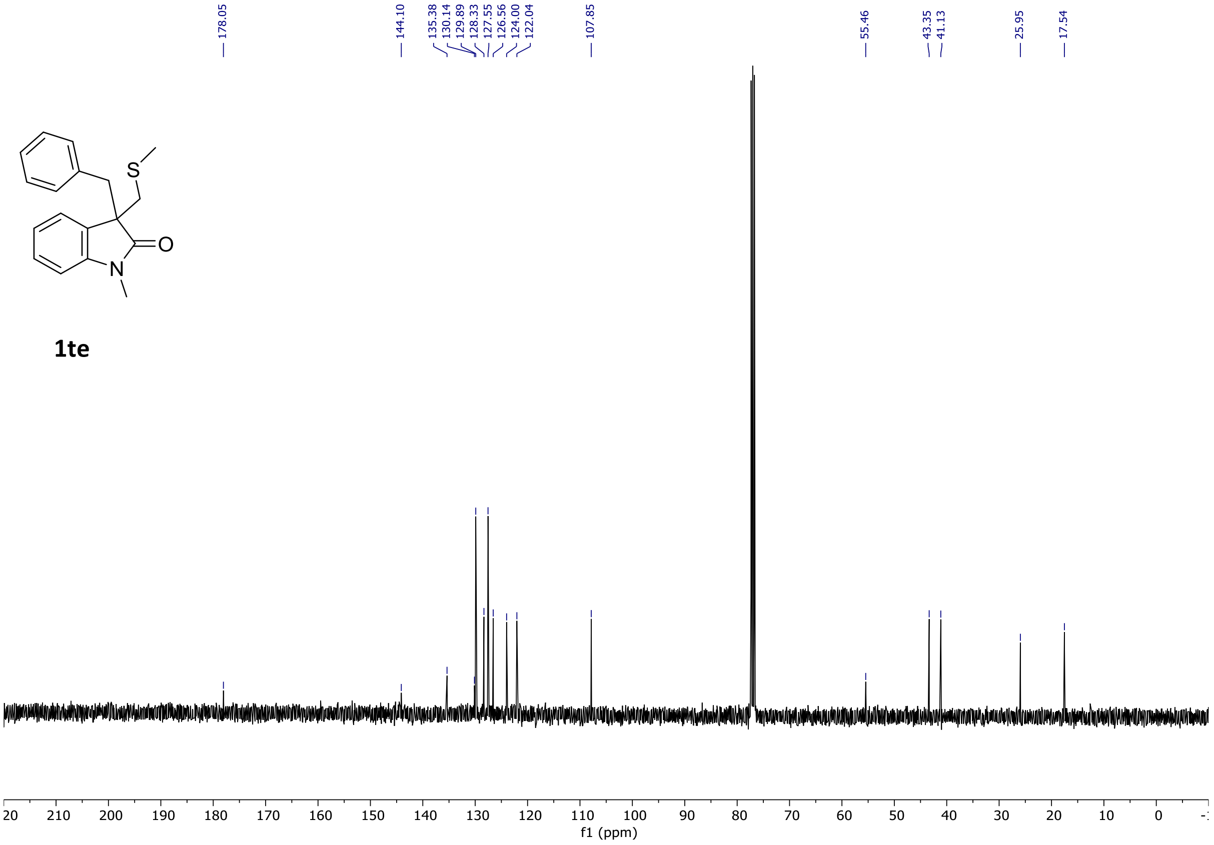
178.05
144.10
135.38 130.14 129.89 128.33 127.55 126.56 124.00 122.04
107.85
55.46
43.35 41.13
25.95
17.54
S
O
N
1te
20 210 200 190 180 170 160 150 140 130 120 110 100 90 80 70 60 50 40 30 20 10 0
f1 (ppm)

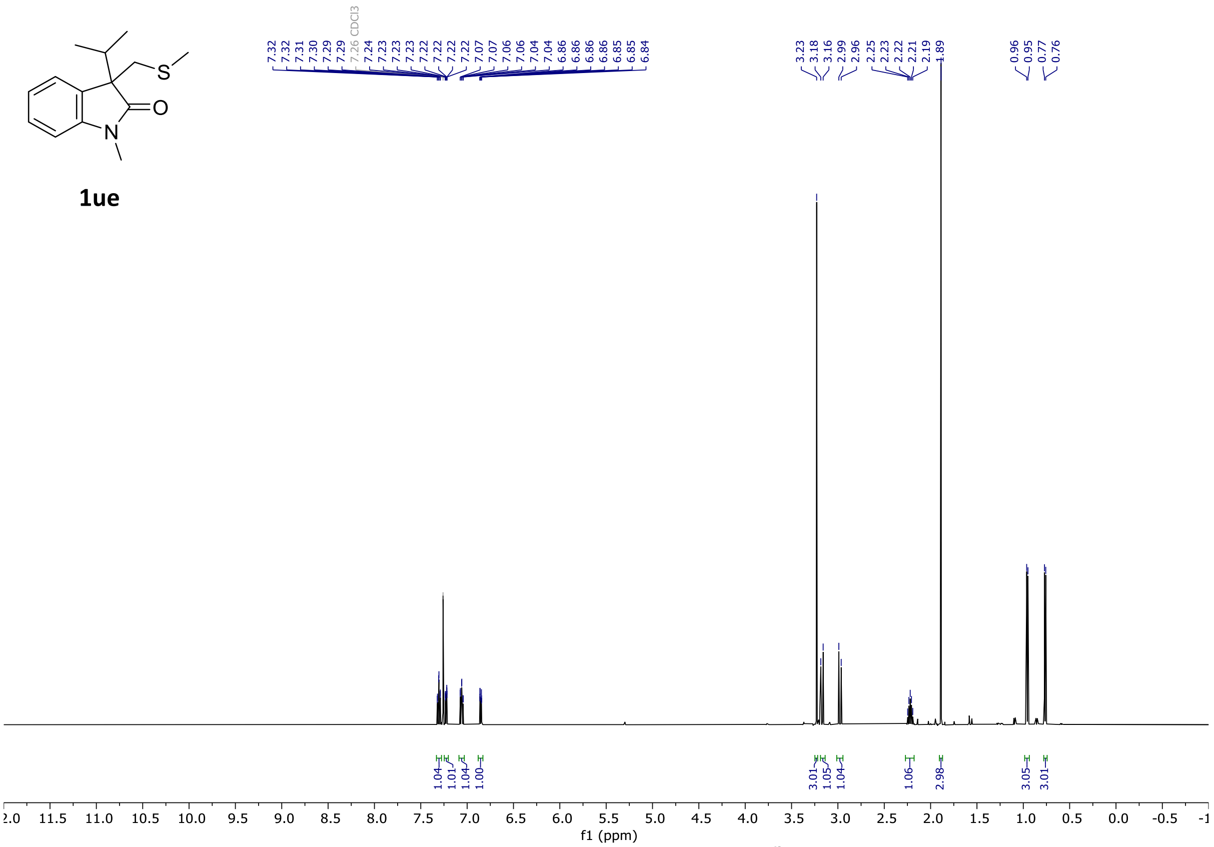
1ue
7.32 7.32 7.31 7.30 7.29 7.29 7.26 CDCl3 7.24 7.23 7.23 7.23 7.22 7.22 7.22 7.22 7.07 7.07 7.06 7.06 7.04 7.04 6.86 6.86 6.86 6.86 6.85 6.84
3.23 3.18 3.16 2.99 2.96 2.25 2.23 2.22 2.21 2.19 1.89
0.96 0.95 0.77 0.76
1.04 1.01 1.04 1.00 3.01 1.05 1.04 1.06 2.98 3.05 3.01
f1 (ppm)

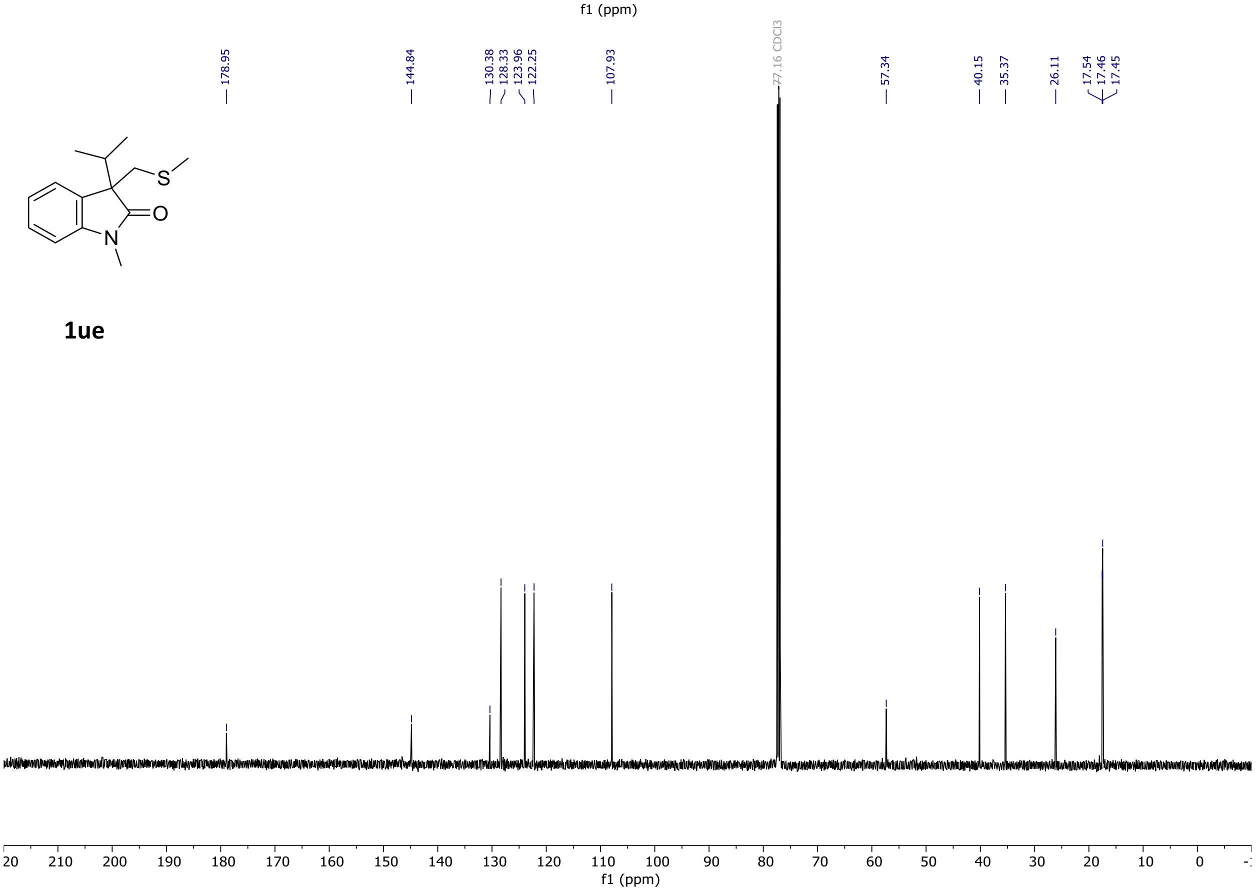
1ue
178.95
144.84
130.38 128.33 123.96 122.25
107.93
77.16 CDCl3
57.34
40.15
35.37
26.11
17.54 17.46 17.45
f1 (ppm)

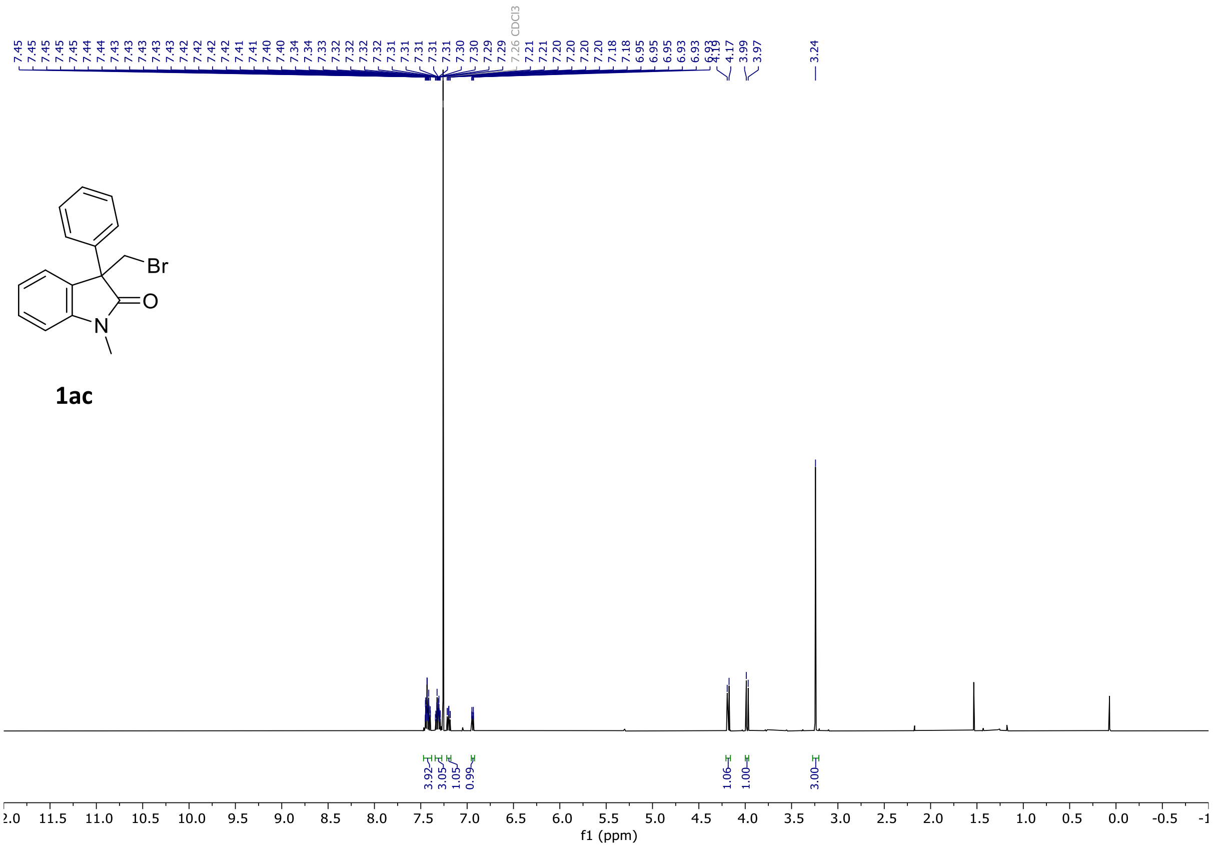
7.45 7.45 7.45 7.45 7.45 7.44 7.44 7.43 7.43 7.43 7.43 7.42 7.42 7.42 7.41 7.41 7.40 7.40 7.34 7.34 7.33 7.32 7.32 7.32 7.31 7.31 7.31 7.31 7.31 7.30 7.29 7.29
7.26 CDCl3
7.21 7.21 7.20 7.20 7.20 7.20 7.18 6.95 6.95 6.95 6.93 6.93 4.19 4.17 3.99 3.97 3.24
Br
O
N
1ac
3.92 3.05 1.05 0.99 1.06 1.00 3.00
f1 (ppm)

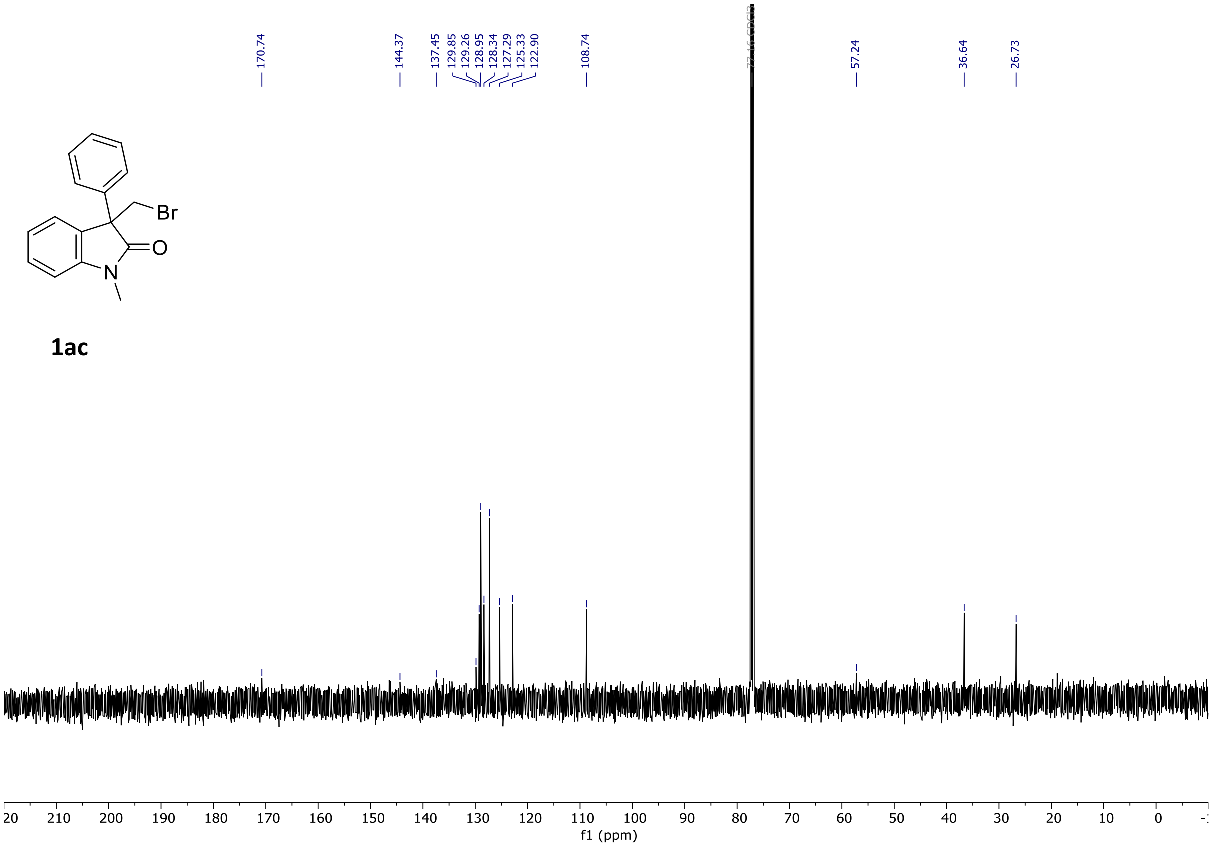
170.74
144.37
137.45 129.85 129.26 128.95 128.34 127.29 125.33 122.90
108.74
57.24
36.64
26.73
Br
O
N
1ac
f1 (ppm)

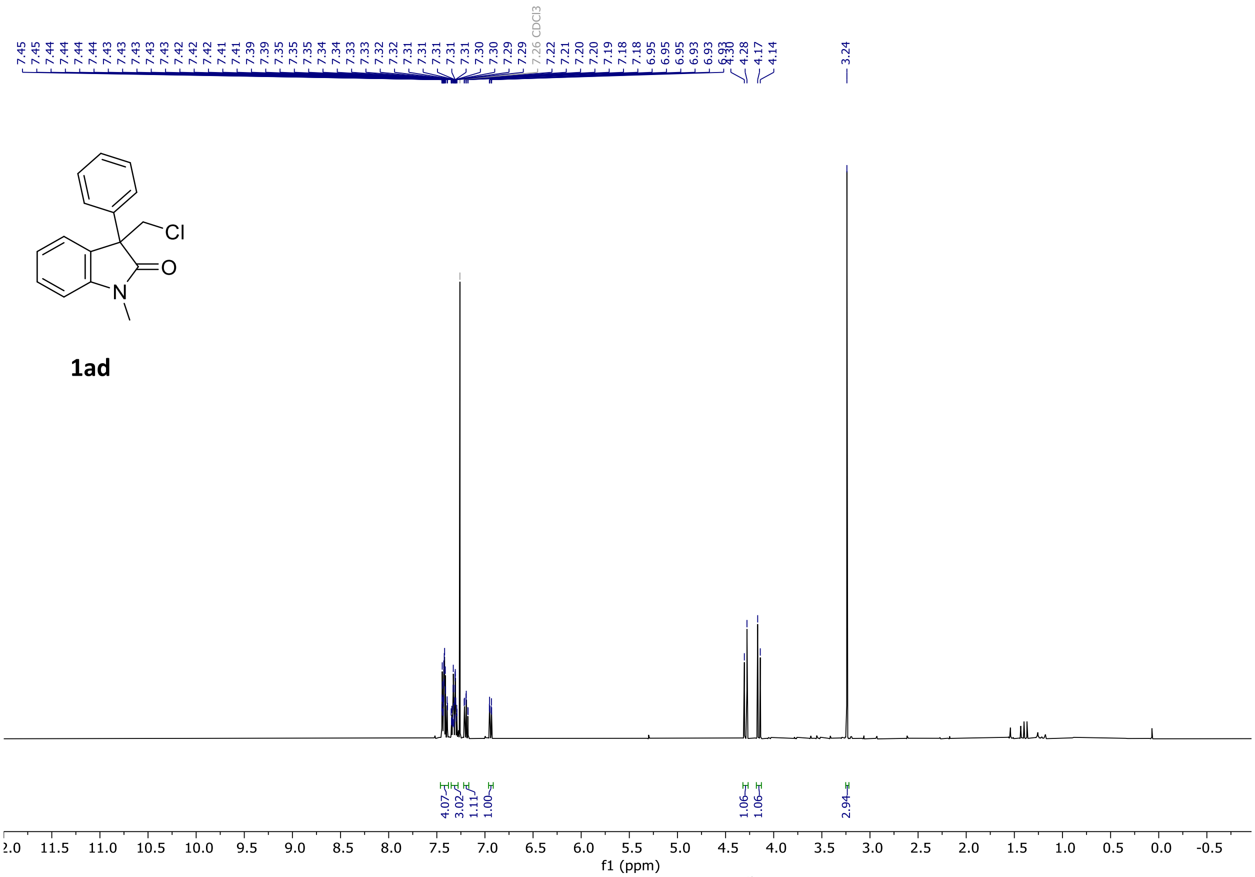
7.45
7.45
7.44
7.44
7.44
7.43
7.43
7.43
7.43
7.42
7.42
7.41
7.41
7.39
7.39
7.35
7.35
7.35
7.34
7.34
7.33
7.33
7.32
7.31
7.31
7.31
7.31
7.30
7.30
7.29
7.29
7.26 CDCl3
7.22
7.21
7.20
7.19
7.18
6.95
6.95
6.95
6.93
6.93
4.30
4.28
4.17
4.14
3.24
Cl
O
N
1ad
4.07
3.02
1.11
1.00
1.06
1.06
2.94
f1 (ppm)

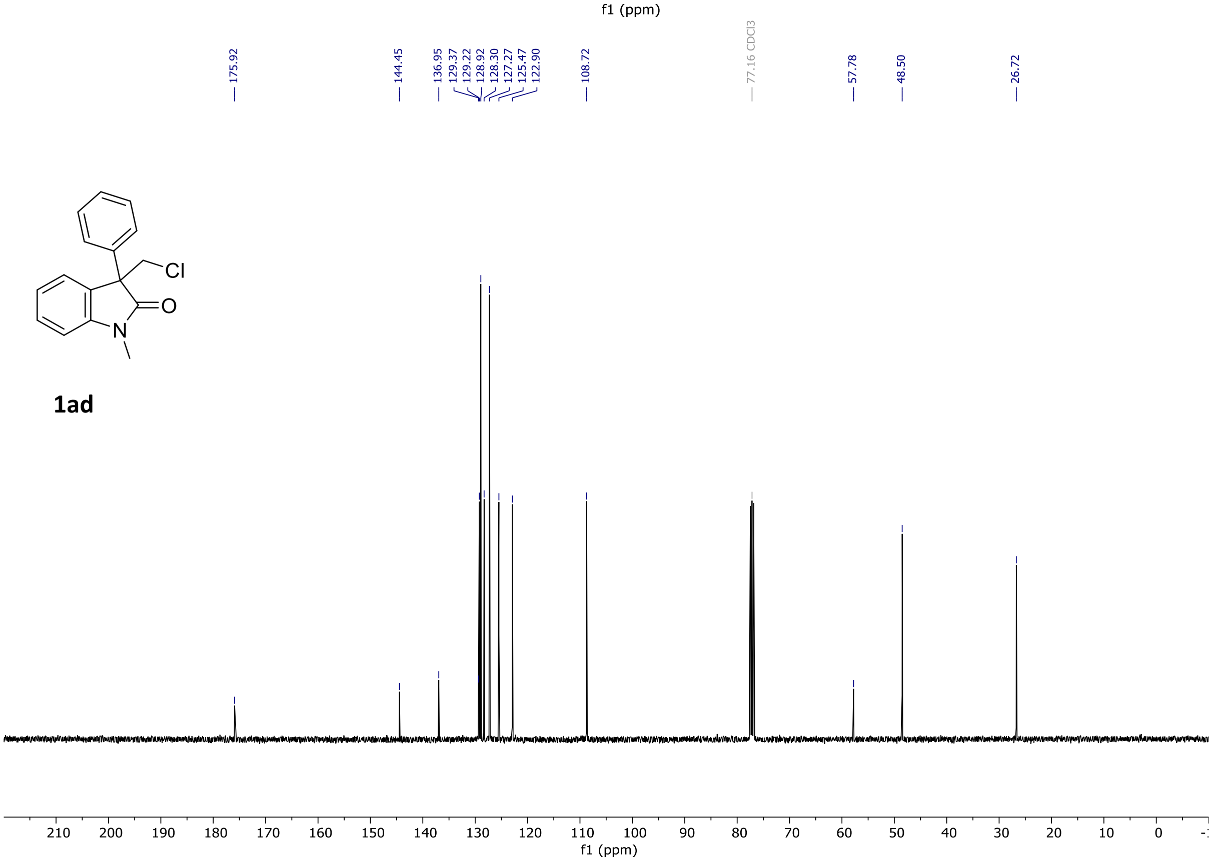
175.92
144.45
136.95
129.37
129.22
128.92
128.30
127.27
125.47
122.90
108.72
77.16 CDCl3
57.78
48.50
26.72
Cl
O
N
1ad
f1 (ppm)

Scope reaction A

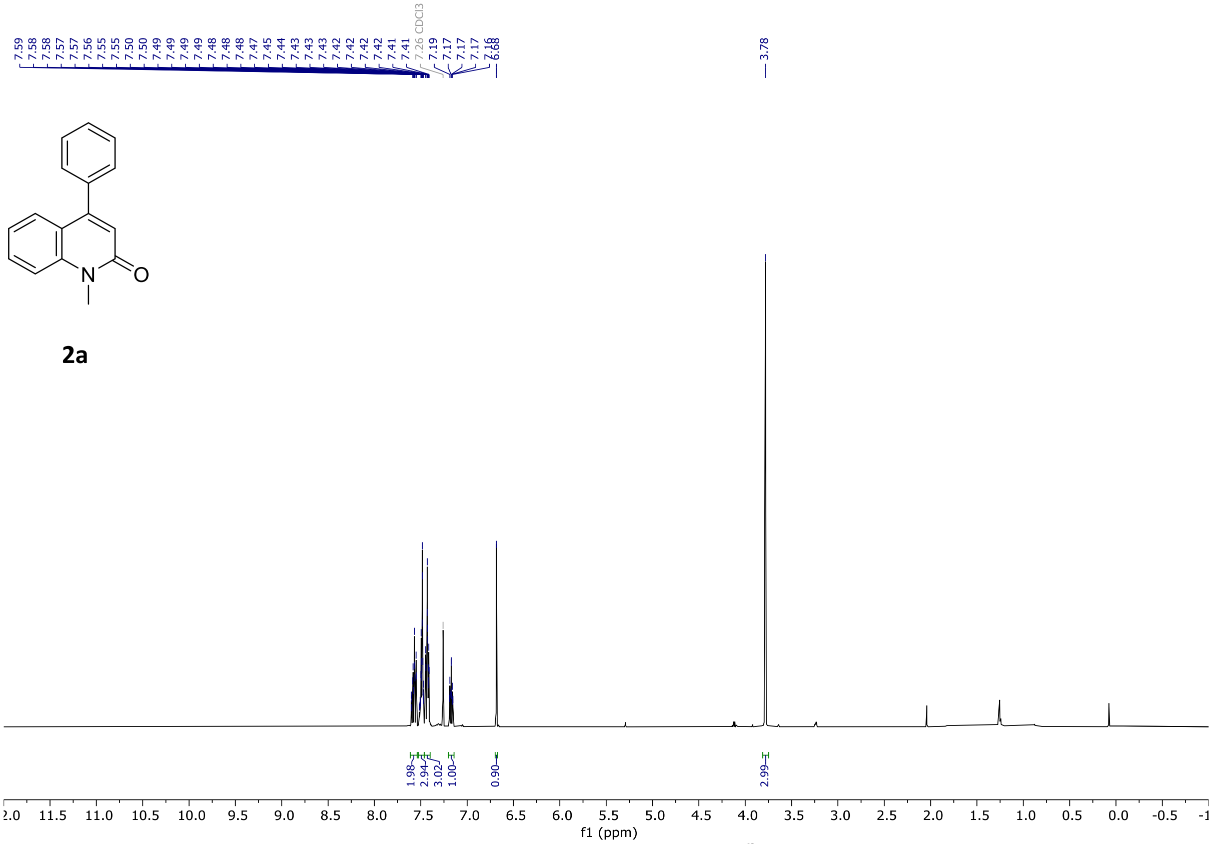
7.59
7.58
7.58
7.57
7.56
7.55
7.55
7.50
7.49
7.49
7.49
7.49
7.48
7.48
7.47
7.45
7.44
7.43
7.43
7.43
7.42
7.42
7.42
7.41
7.41
7.26 CDCl3
7.19
7.17
7.17
7.17
7.16
6.68
3.78
1.98
2.94
3.02
1.00
0.90
2.99
f1 (ppm)

2a

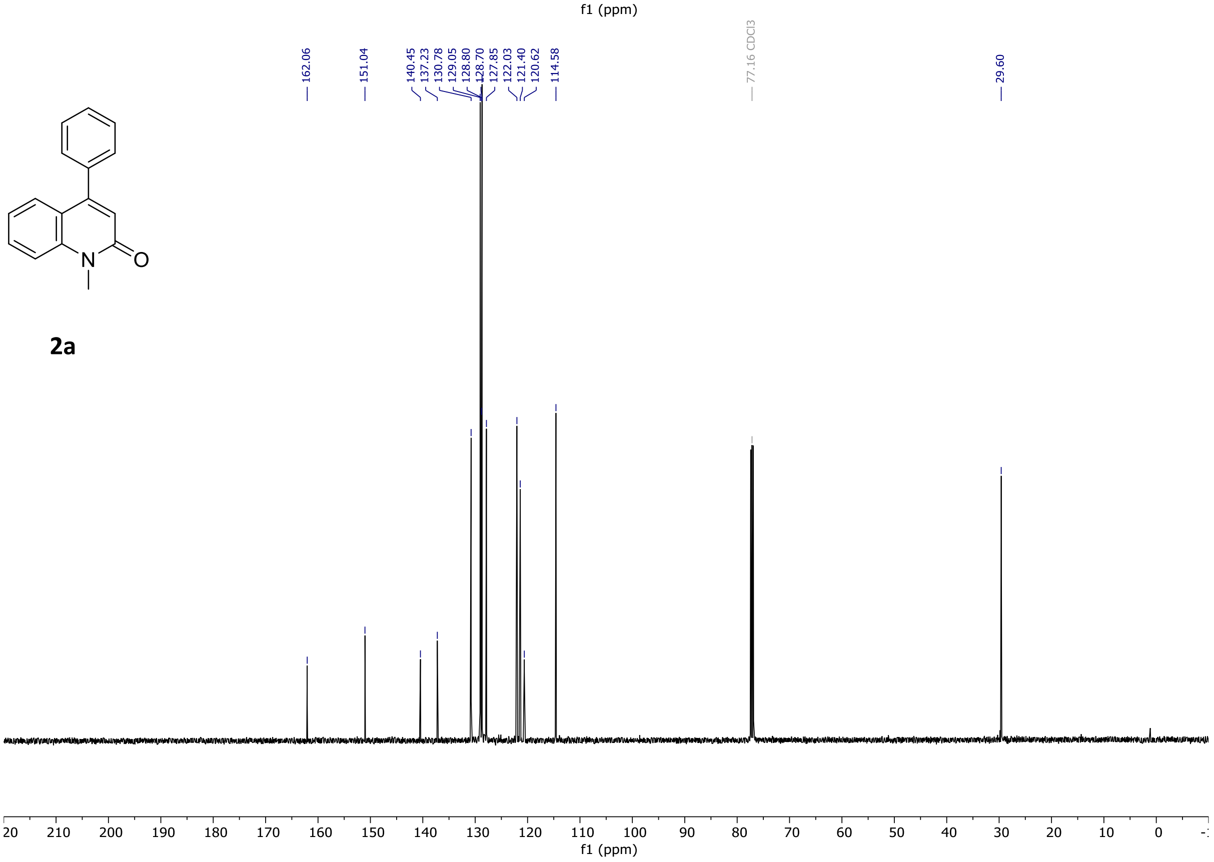
162.06
151.04
140.45
137.23
130.78
129.05
128.80
128.70
127.85
122.03
121.40
120.62
114.58
77.16 CDCl3
29.60
f1 (ppm)

2a

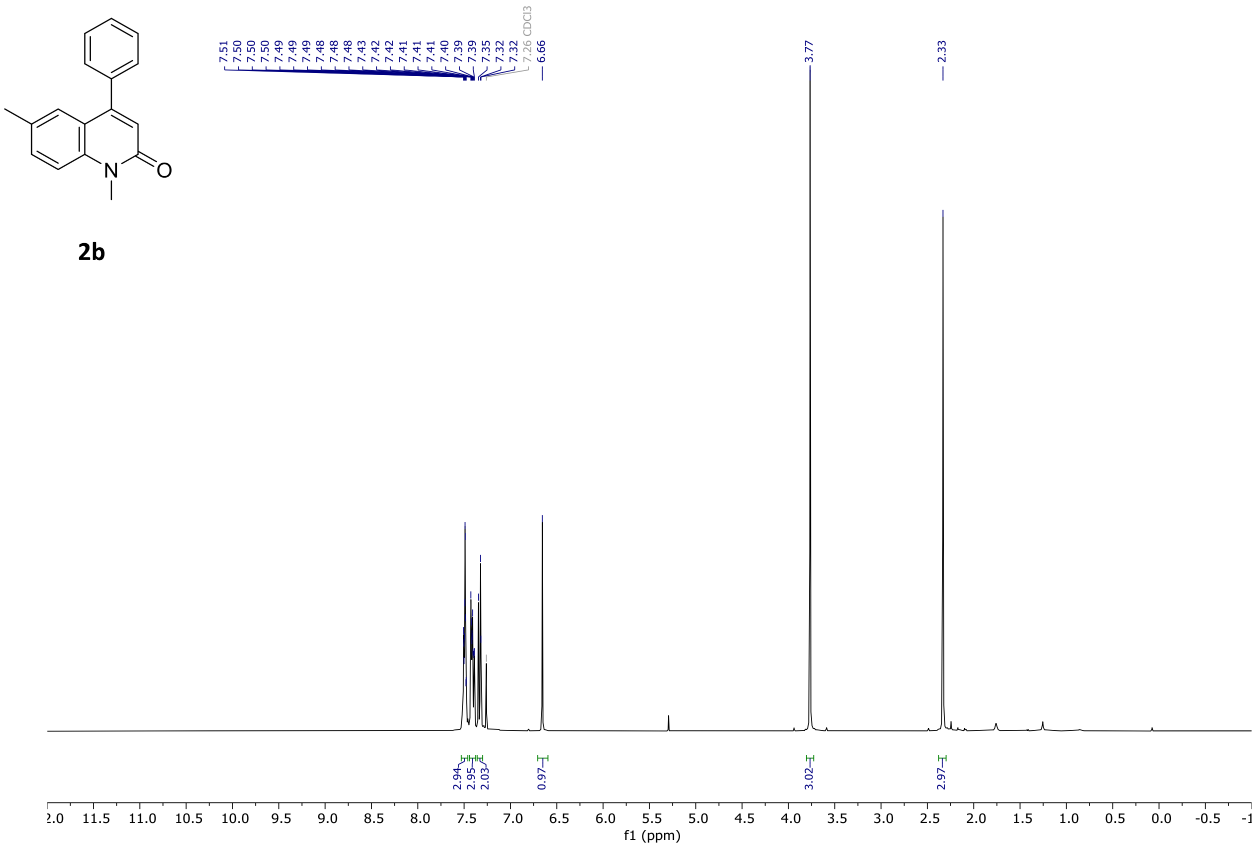
7.51
7.50
7.50
7.50
7.49
7.49
7.49
7.48
7.48
7.48
7.43
7.42
7.42
7.41
7.41
7.41
7.40
7.39
7.39
7.35
7.32
7.32
7.26 CDCl3
6.66
3.77
2.33
2.94
2.95
2.03
0.97
3.02
2.97
f1 (ppm)

2b

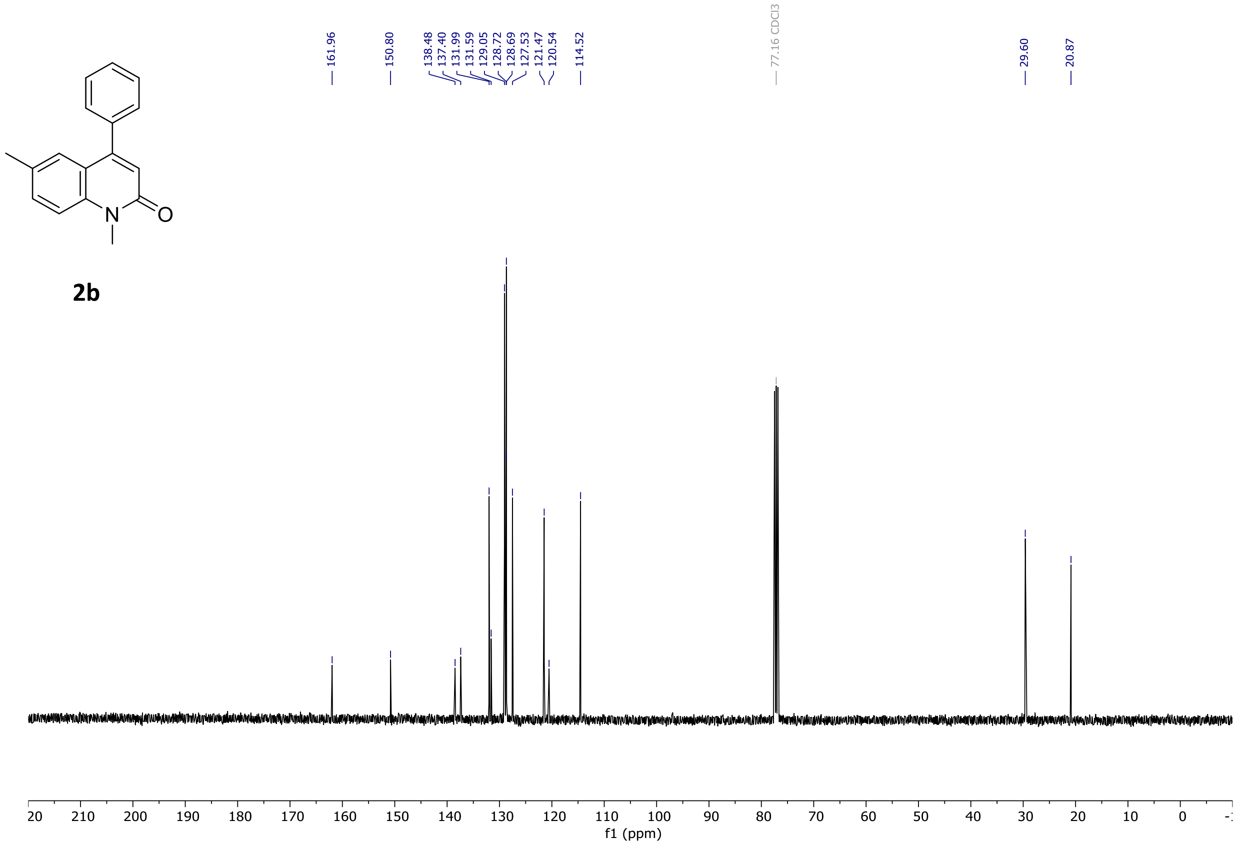
161.96
150.80
138.48
137.40
131.99
131.59
129.05
128.72
128.69
127.53
121.47
120.54
114.52
77.16 CDCl3
29.60
20.87
f1 (ppm)

2b

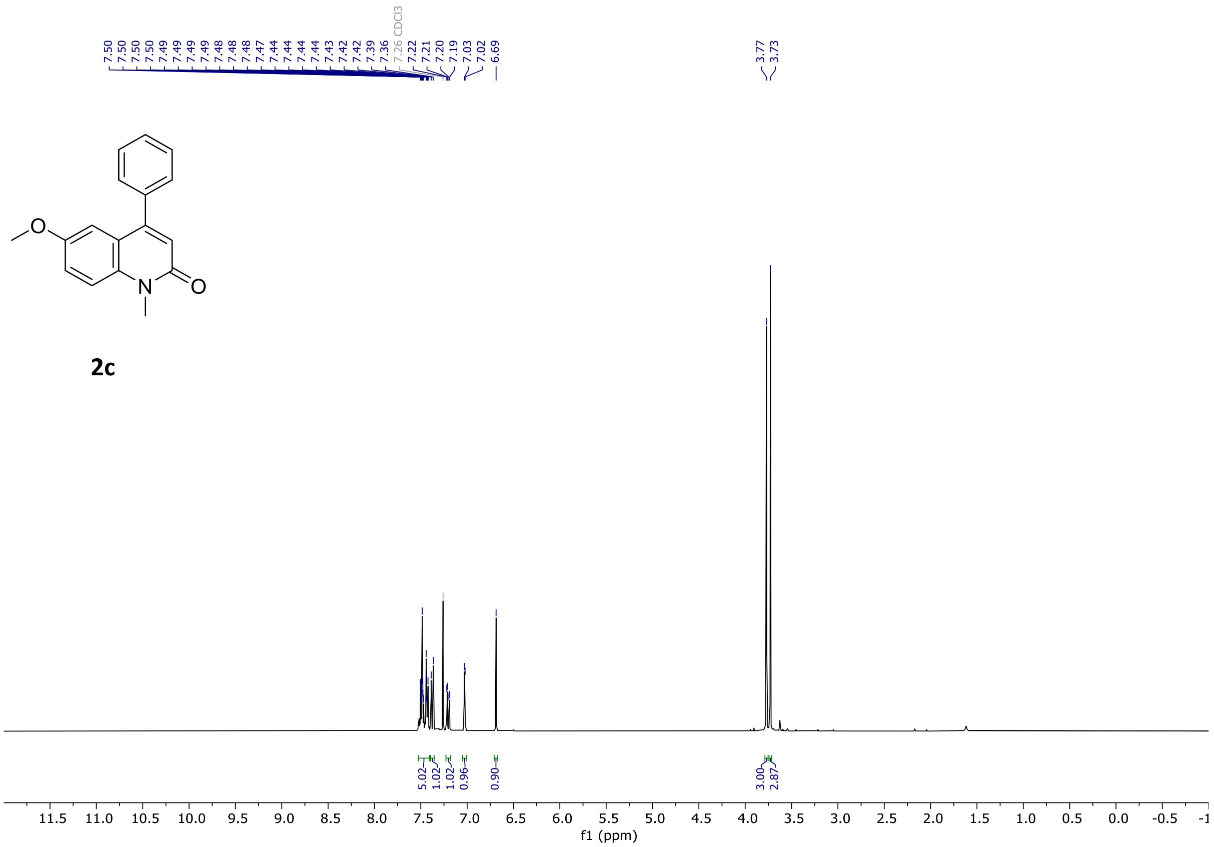
7.50 7.50 7.50 7.50 7.49 7.49 7.49 7.48 7.48 7.48 7.47 7.44 7.44 7.44 7.44 7.43 7.42 7.42 7.39 7.36 7.26 CDCl3 7.22 7.21 7.20 7.19 7.03 7.02 6.69
3.77 3.73
2c
5.02 1.02 1.02 0.96 0.90
3.00 2.87
11.5 11.0 10.5 10.0 9.5 9.0 8.5 8.0 7.5 7.0 6.5 6.0 5.5 5.0 4.5 4.0 3.5 3.0 2.5 2.0 1.5 1.0 0.5 0.0 -0.5
f1 (ppm)

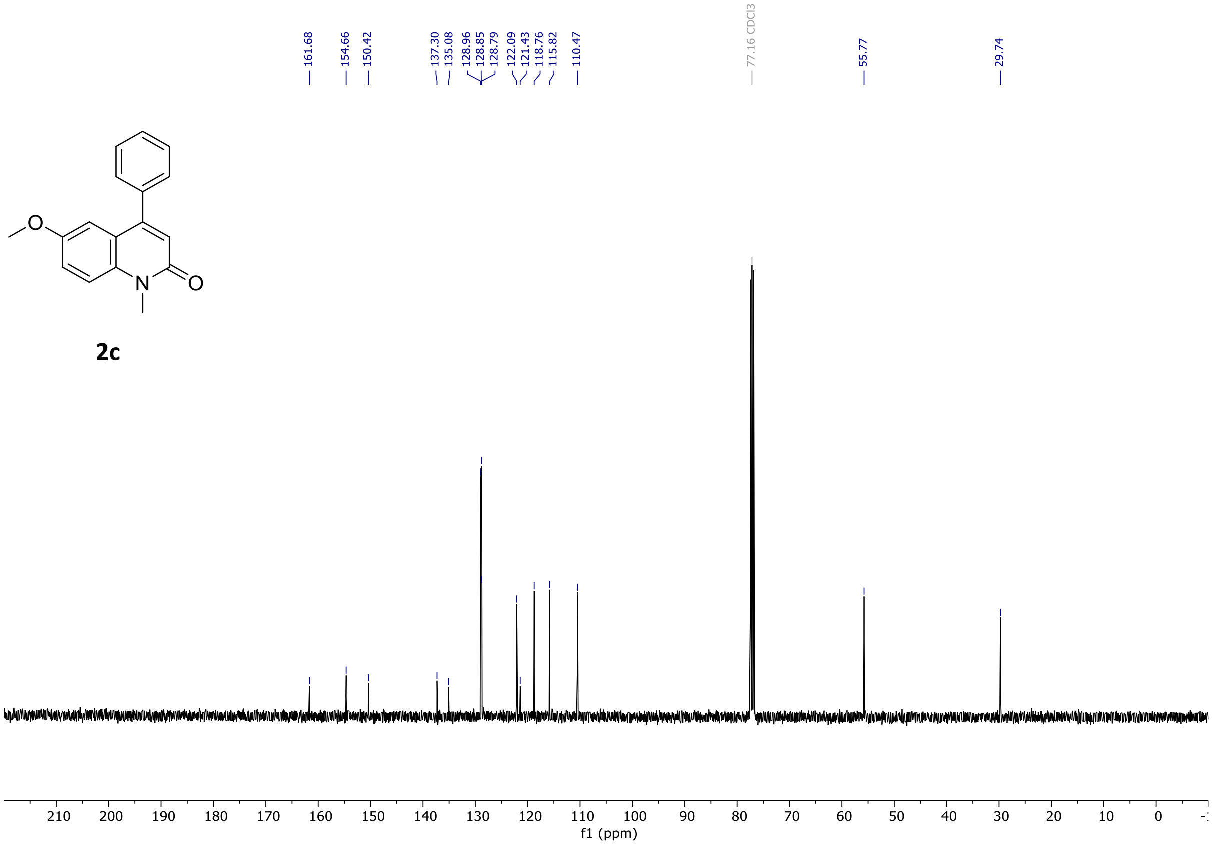
161.68 154.66 150.42 137.30 135.08 128.96 128.85 128.79 122.09 121.43 118.76 115.82 110.47
77.16 CDCl3
55.77
29.74
2c
210 200 190 180 170 160 150 140 130 120 110 100 90 80 70 60 50 40 30 20 10 0
f1 (ppm)

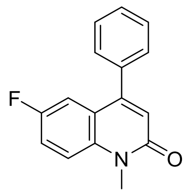

2d

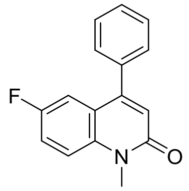

2d

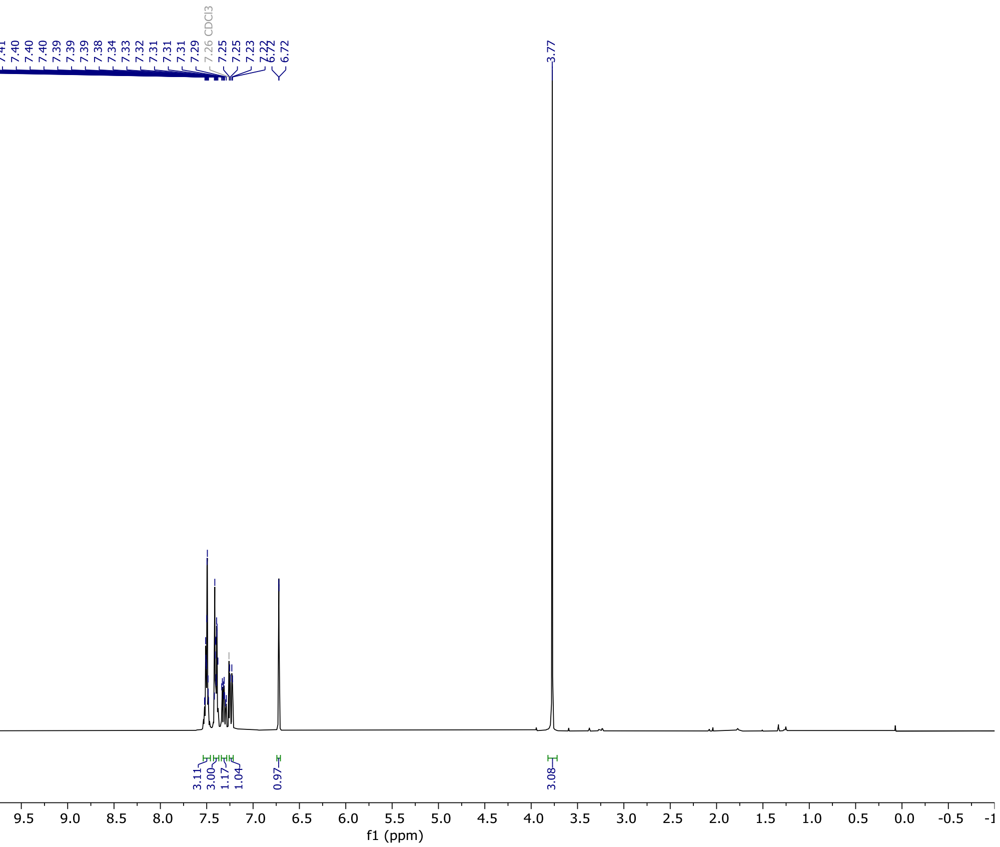

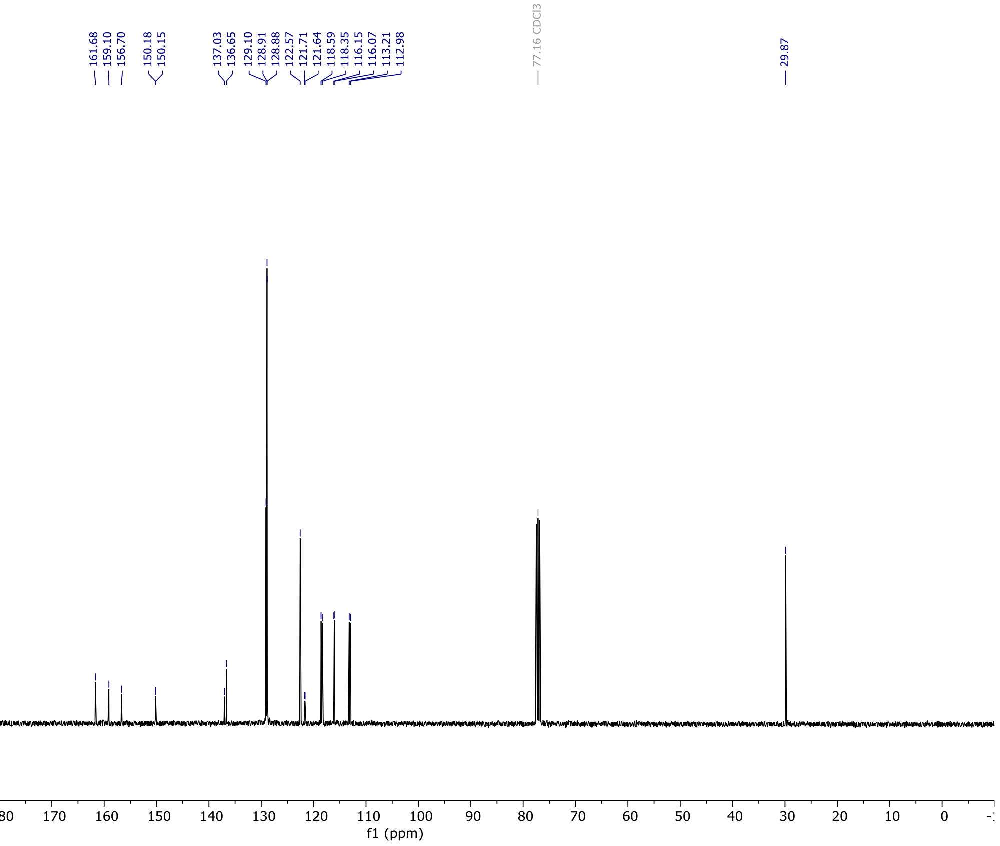

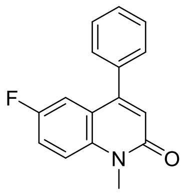

2d

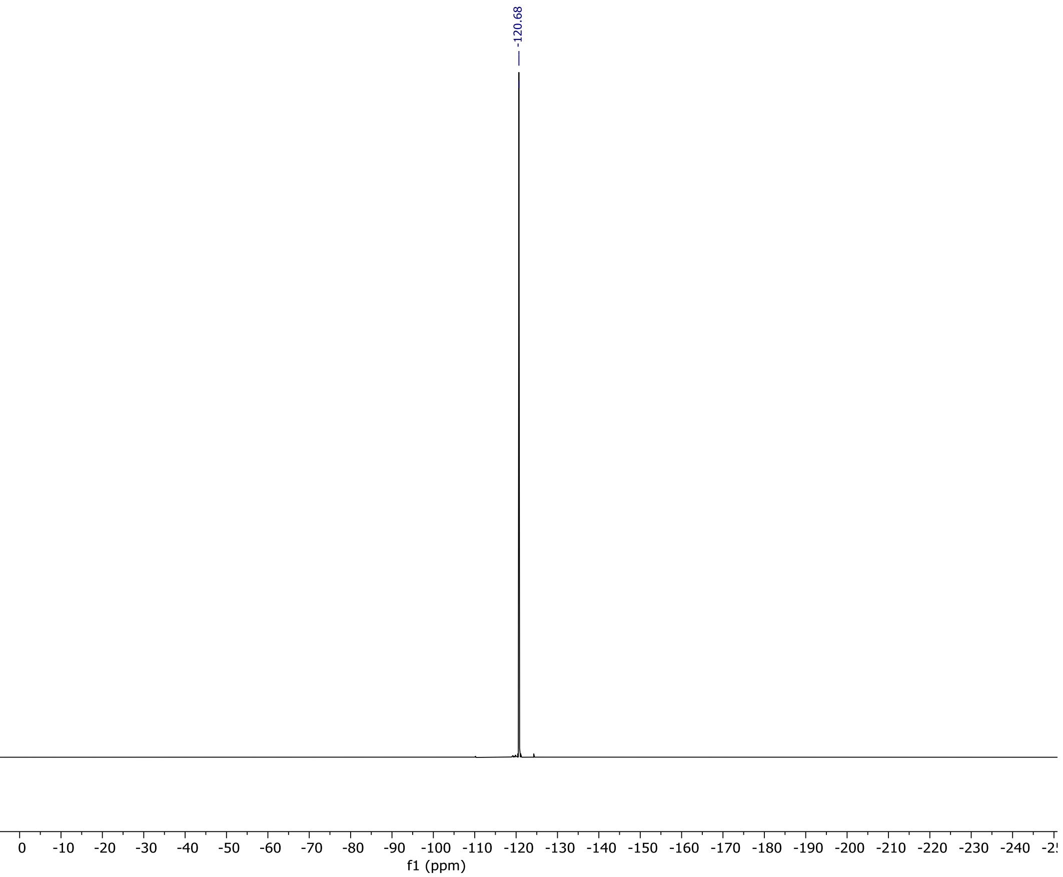

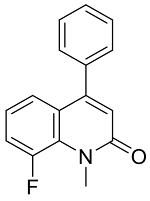

2e

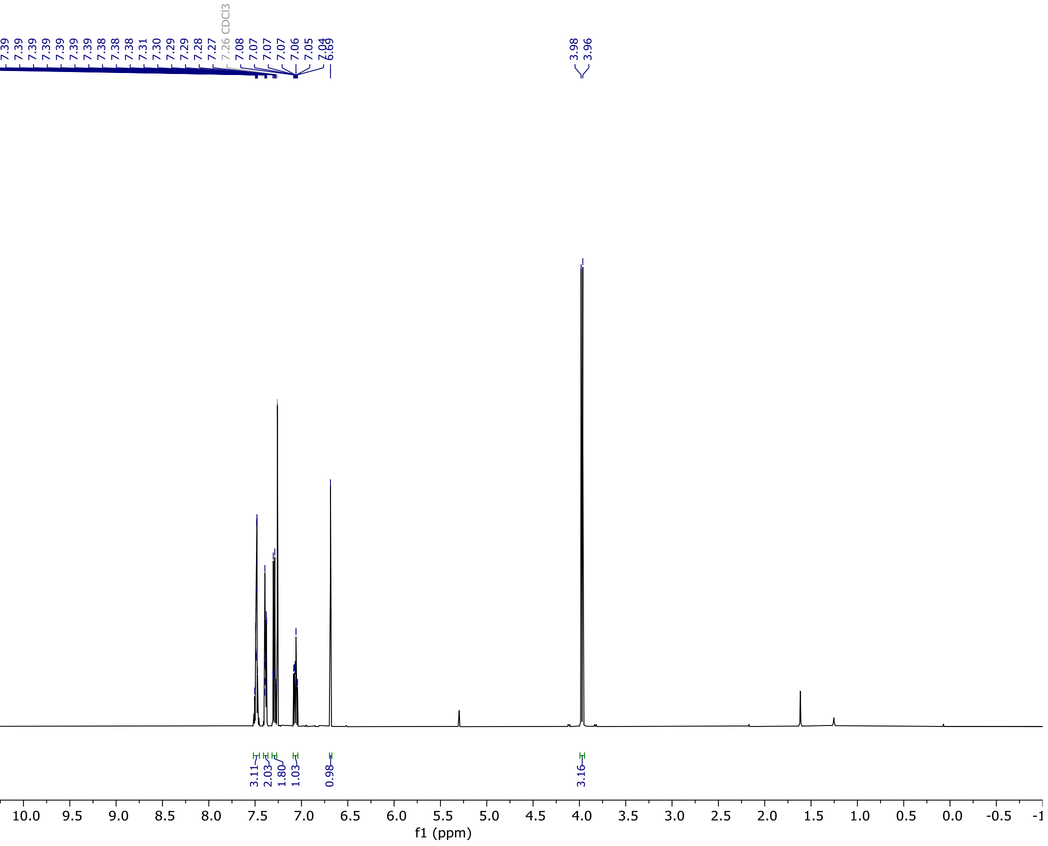

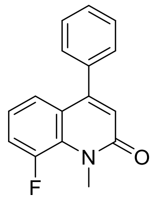

2e

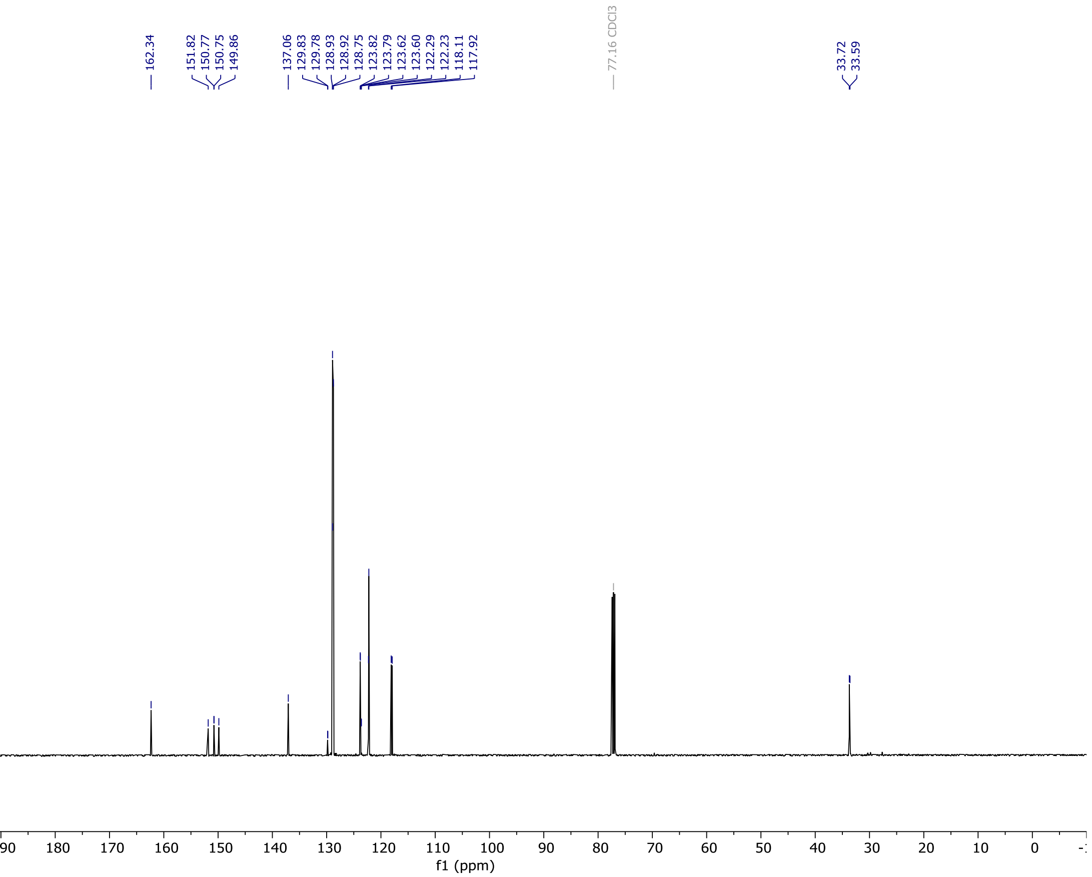
162.34
151.82
150.77
150.75
149.86
137.06
129.83
129.78
128.93
128.92
128.75
123.82
123.79
123.62
123.60
122.29
122.23
118.11
117.92
77.16 CDCl3
33.72
33.59
f1 (ppm)

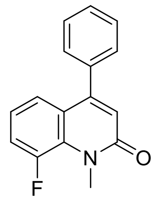

2e

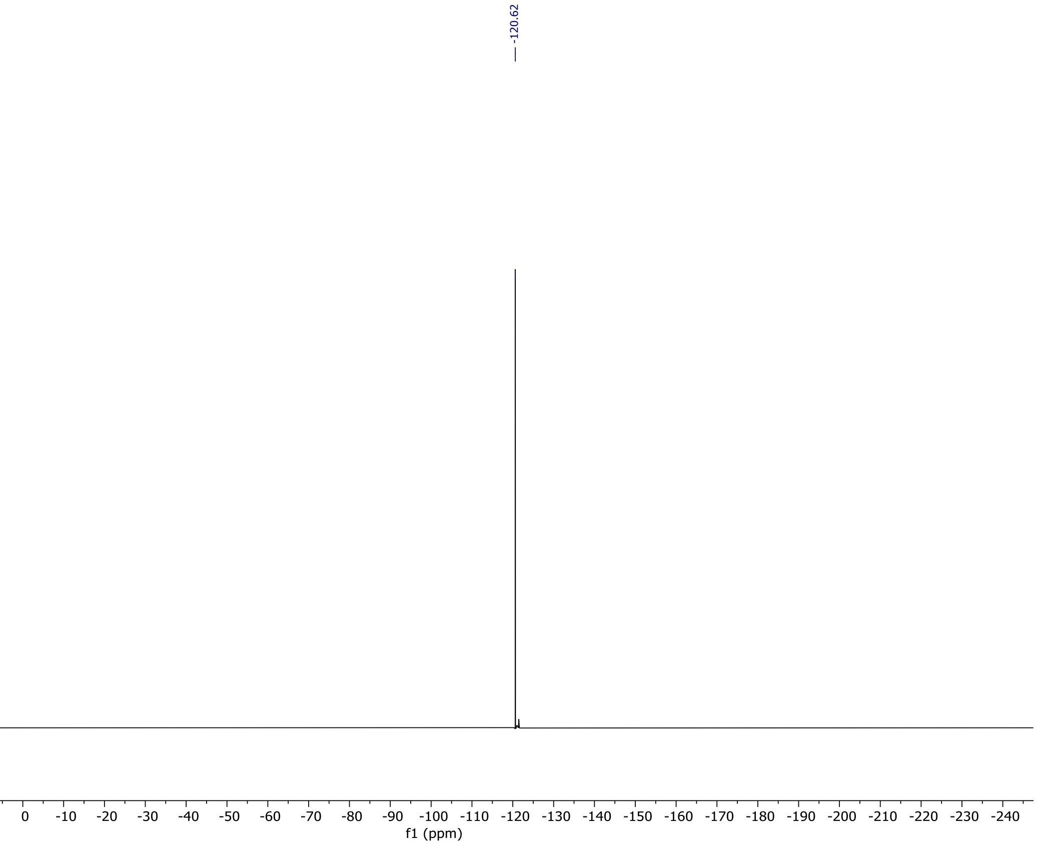
-120.62
f1 (ppm)

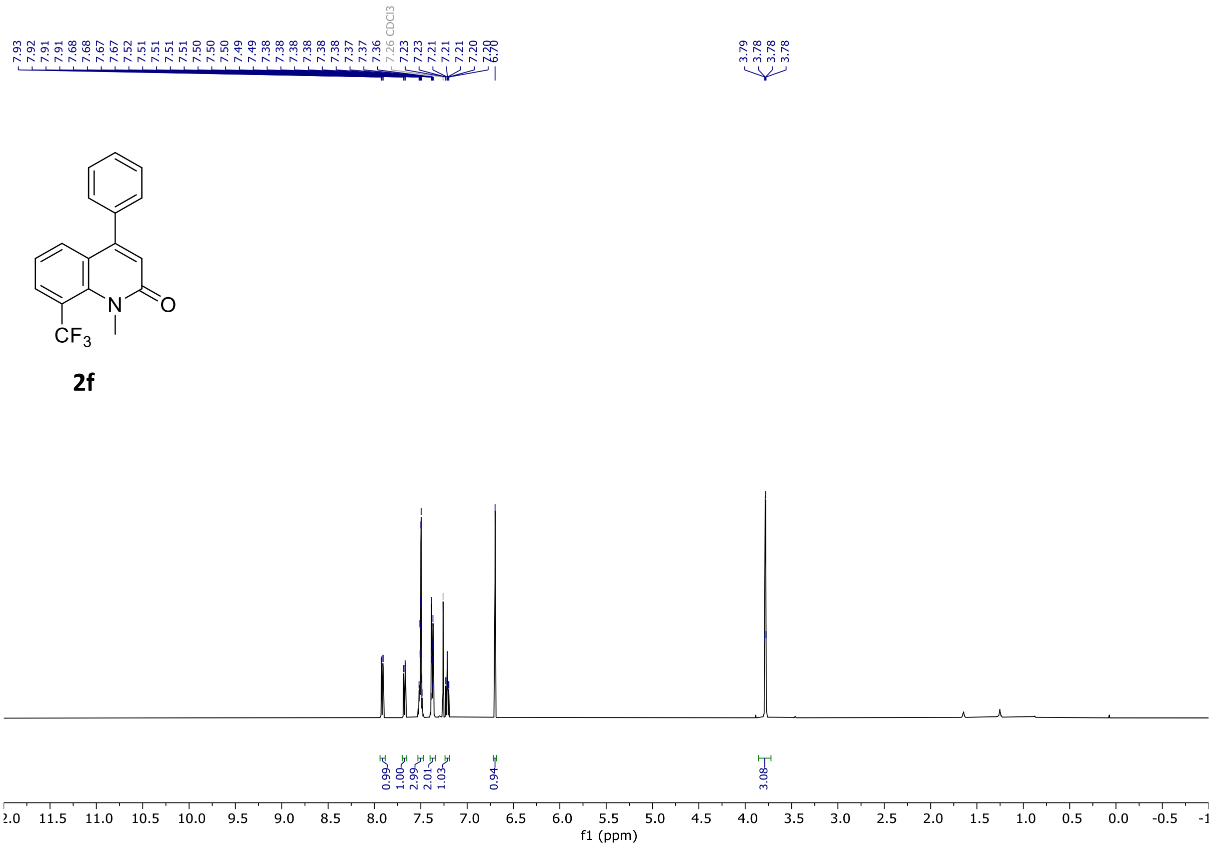
7.93 7.92 7.91 7.91 7.68 7.67 7.67 7.52 7.51 7.51 7.51 7.50 7.50 7.50 7.49 7.49 7.38 7.38 7.38 7.38 7.38 7.38 7.37 7.36 7.26 CDCl3 7.23 7.23 7.21 7.21 7.21 7.20 6.70
3.79 3.78 3.78 3.78
CF_3
N
O
2f
0.99 1.00 2.99 2.01 1.03 0.94 3.08
f1 (ppm)

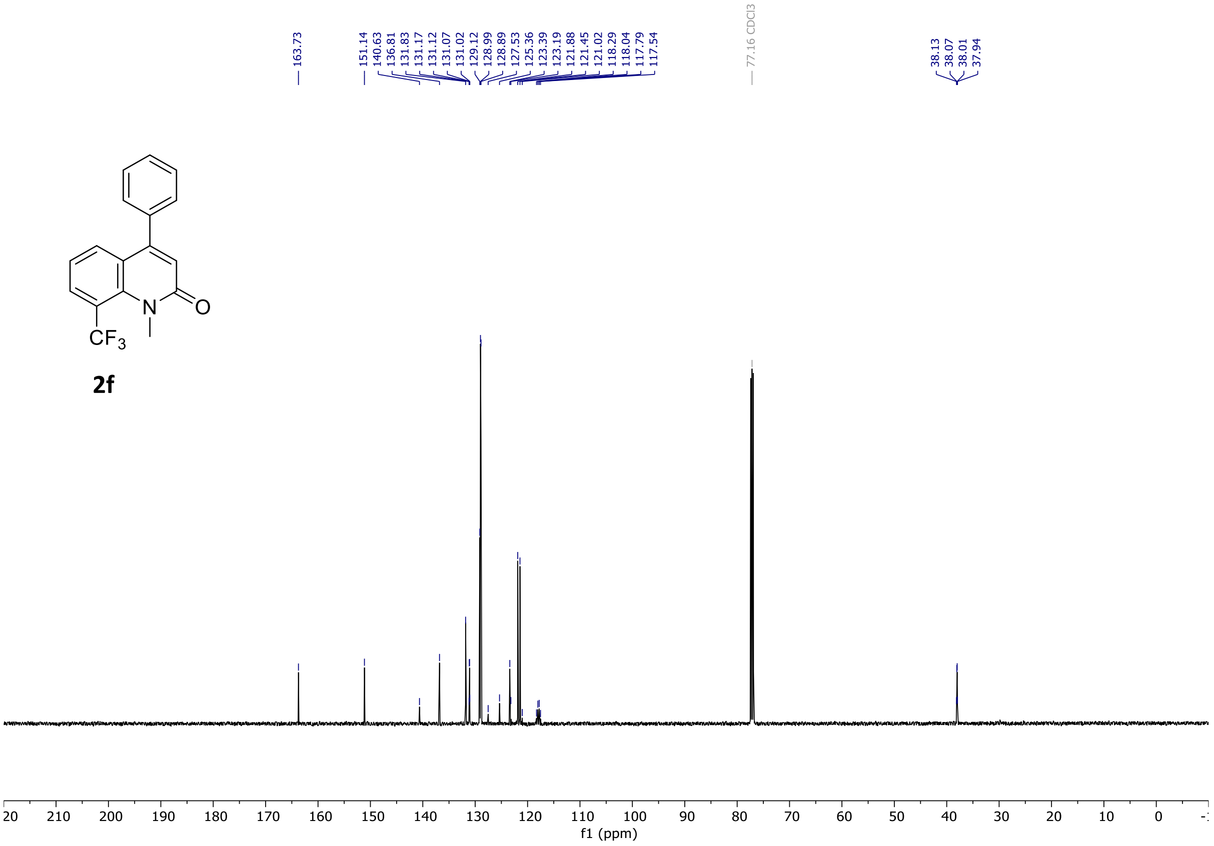
163.73
151.14 140.63 136.81 131.83 131.17 131.12 131.07 131.02 129.12 128.99 128.89 127.53 125.36 123.39 123.19 121.88 121.45 121.02 118.29 118.04 117.79 117.54
77.16 CDCl3
38.13 38.07 38.01 37.94
CF_3
N
O
2f
f1 (ppm)

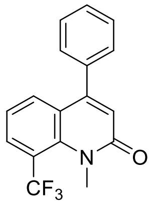

2f

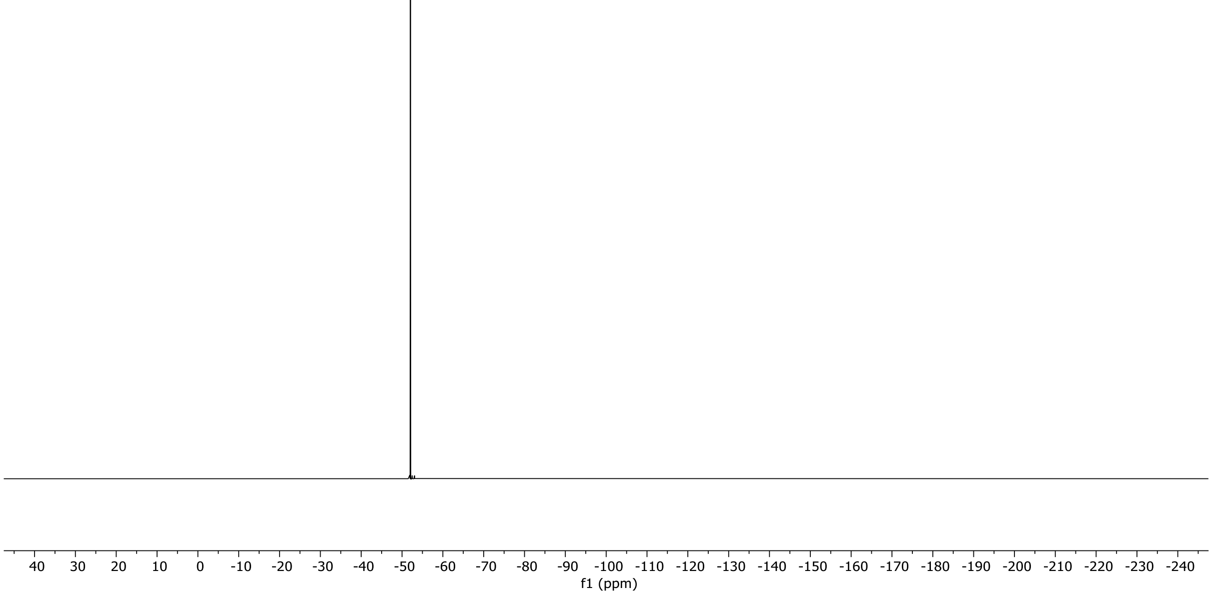

-52.06
40 30 20 10 0 -10 -20 -30 -40 -50 -60 -70 -80 -90 -100 -110 -120 -130 -140 -150 -160 -170 -180 -190 -200 -210 -220 -230 -240
f1 (ppm)

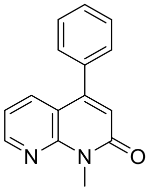

2g

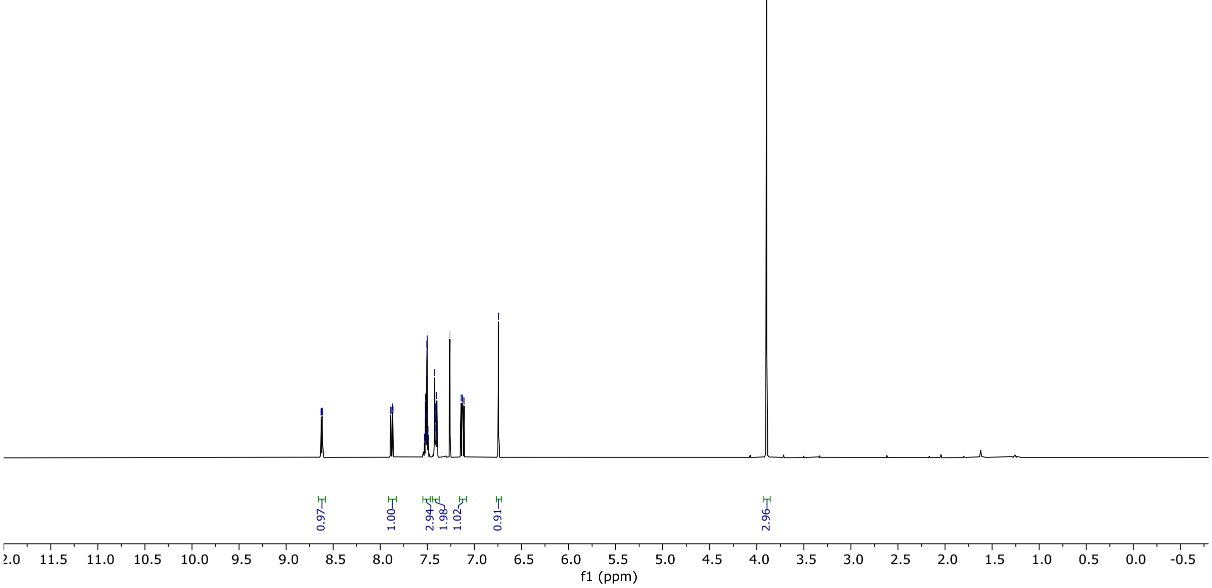

8.63 8.62 8.62 8.61 7.89 7.88 7.87 7.86 7.53 7.53 7.52 7.52 7.52 7.52 7.51 7.51 7.51 7.51 7.50 7.49 7.49 7.49 7.42 7.42 7.42 7.41 7.41 7.41 7.41 7.41 7.40 7.26 CDCl3 7.14 7.13 7.12 7.11 6.74 3.90
0.97 1.00 2.94 1.98 1.02 0.91 2.96
12.0 11.5 11.0 10.5 10.0 9.5 9.0 8.5 8.0 7.5 7.0 6.5 6.0 5.5 5.0 4.5 4.0 3.5 3.0 2.5 2.0 1.5 1.0 0.5 0.0 -0.5
f1 (ppm)

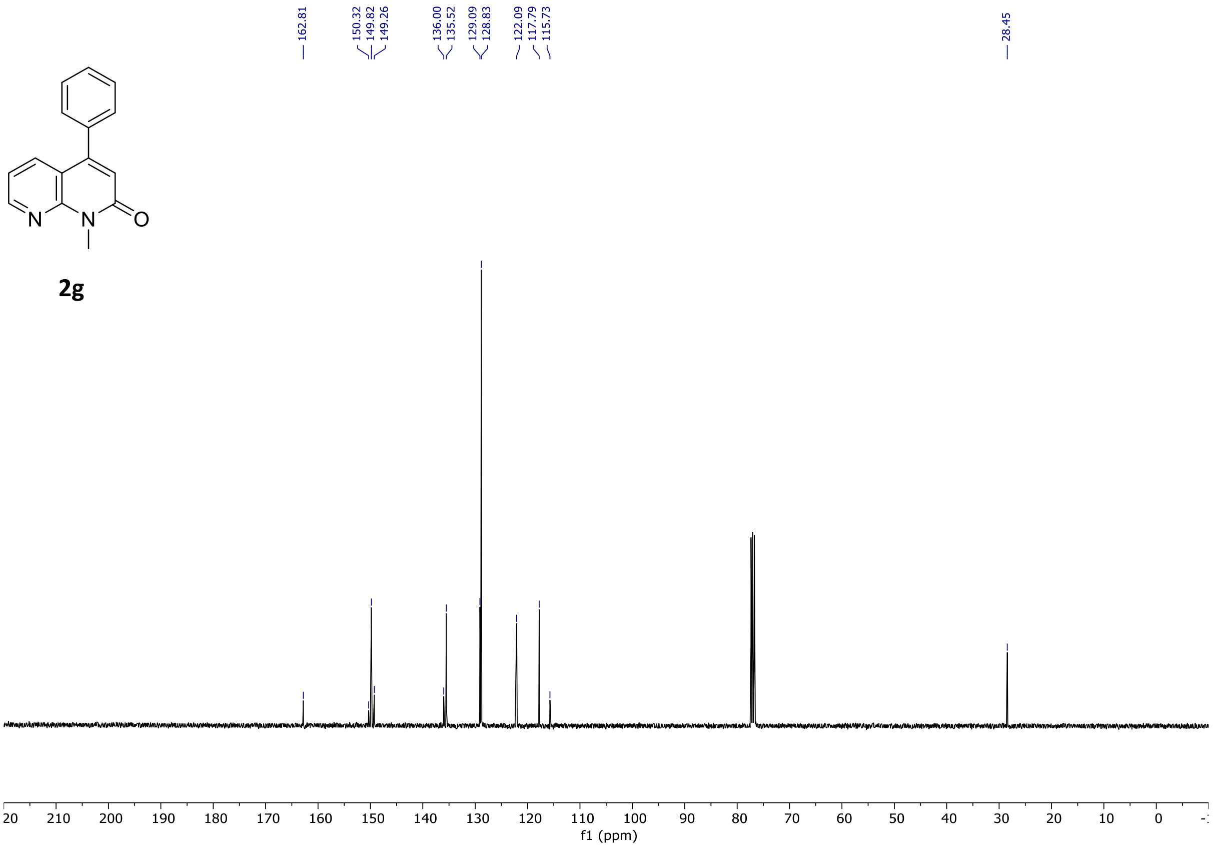
162.81
150.32
149.82
149.26
136.00
135.52
129.09
128.83
122.09
117.79
115.73
28.45
N
N
O
2g
f1 (ppm)

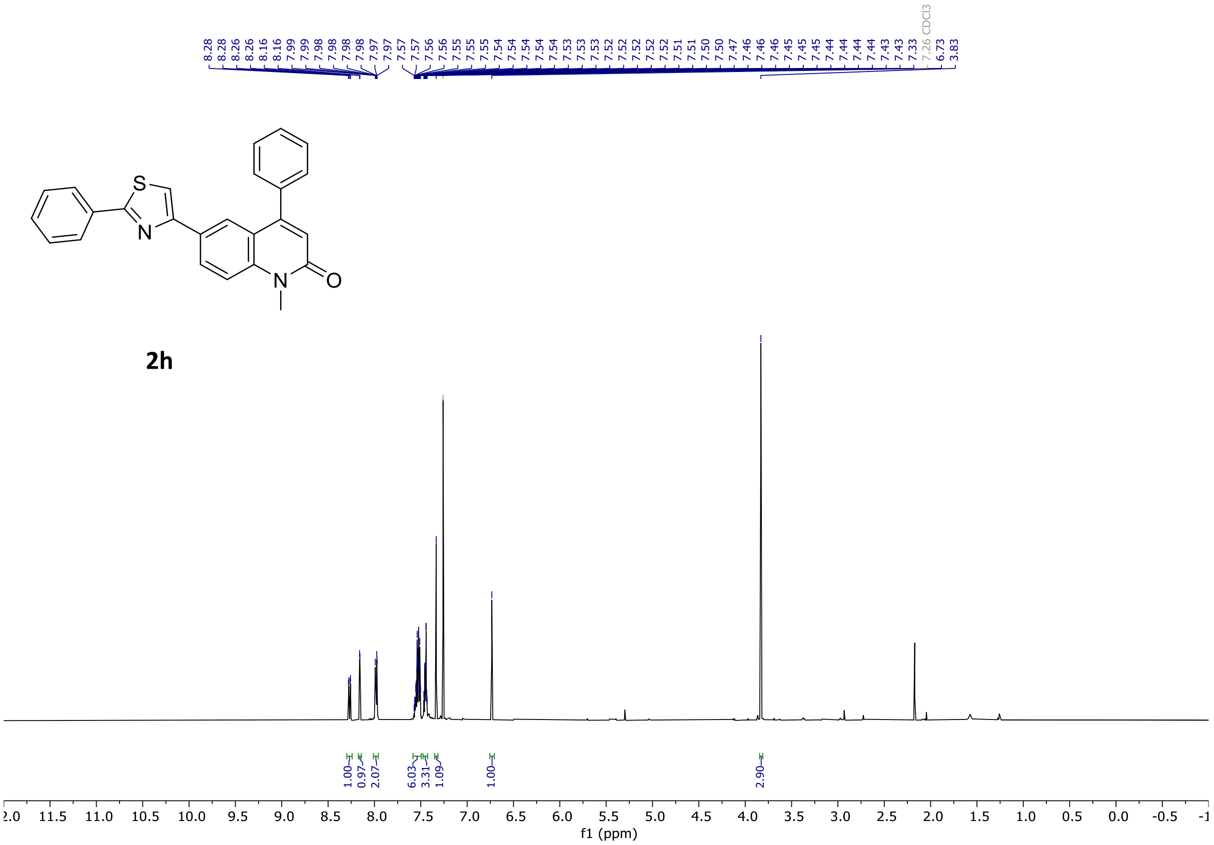
8.28
8.28
8.26
8.26
8.16
8.16
7.99
7.99
7.98
7.98
7.98
7.97
7.97
7.57
7.57
7.56
7.56
7.55
7.55
7.54
7.54
7.54
7.54
7.53
7.53
7.52
7.52
7.52
7.52
7.52
7.51
7.51
7.50
7.47
7.46
7.46
7.45
7.45
7.45
7.44
7.44
7.44
7.43
7.43
7.33
7.26 CDCl3
6.73
3.83
S
N
N
O
2h
1.00
0.97
2.07
6.03
3.31
1.09
1.00
2.90
f1 (ppm)

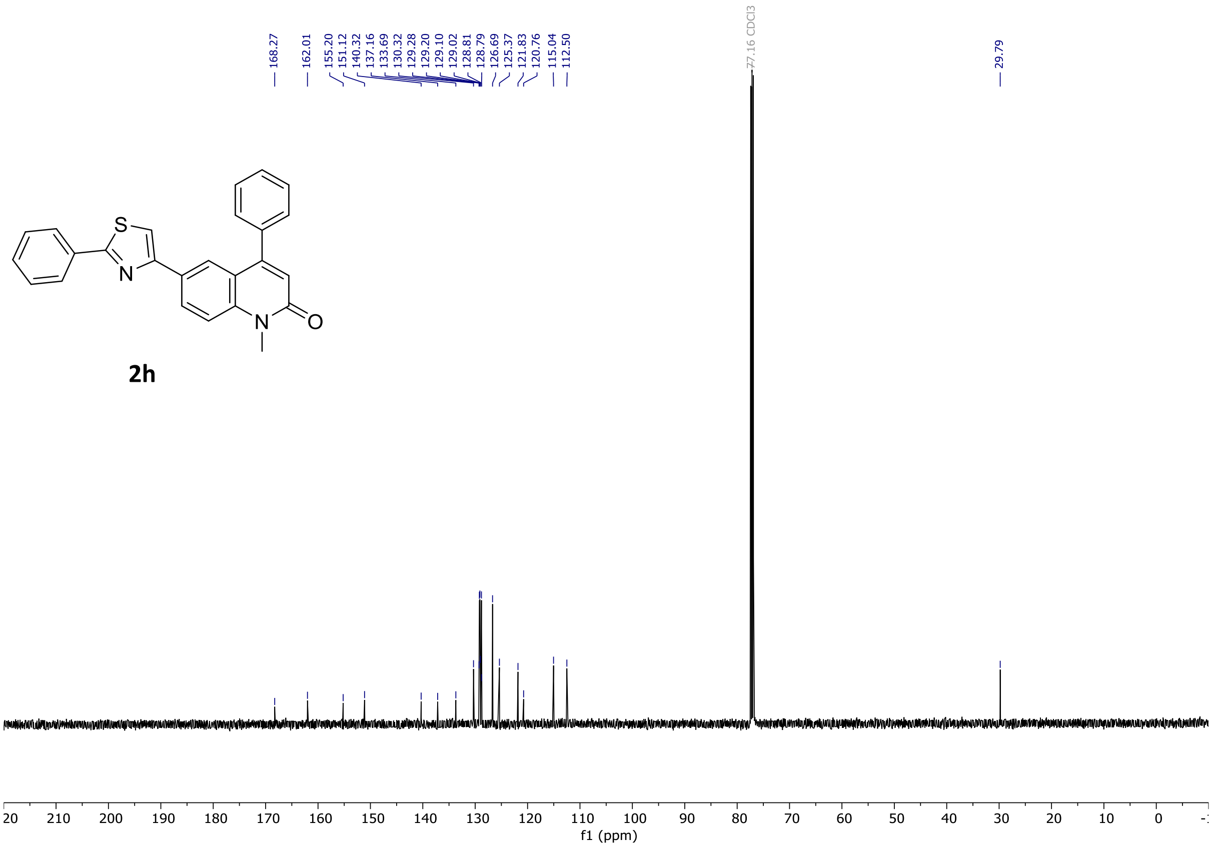
168.27
162.01
155.20
151.12
140.32
137.16
133.69
130.32
129.28
129.20
129.10
129.02
128.81
128.79
126.69
125.37
121.83
120.76
115.04
112.50
77.16 CDCl3
29.79
S
N
N
O
f1 (ppm)

2h

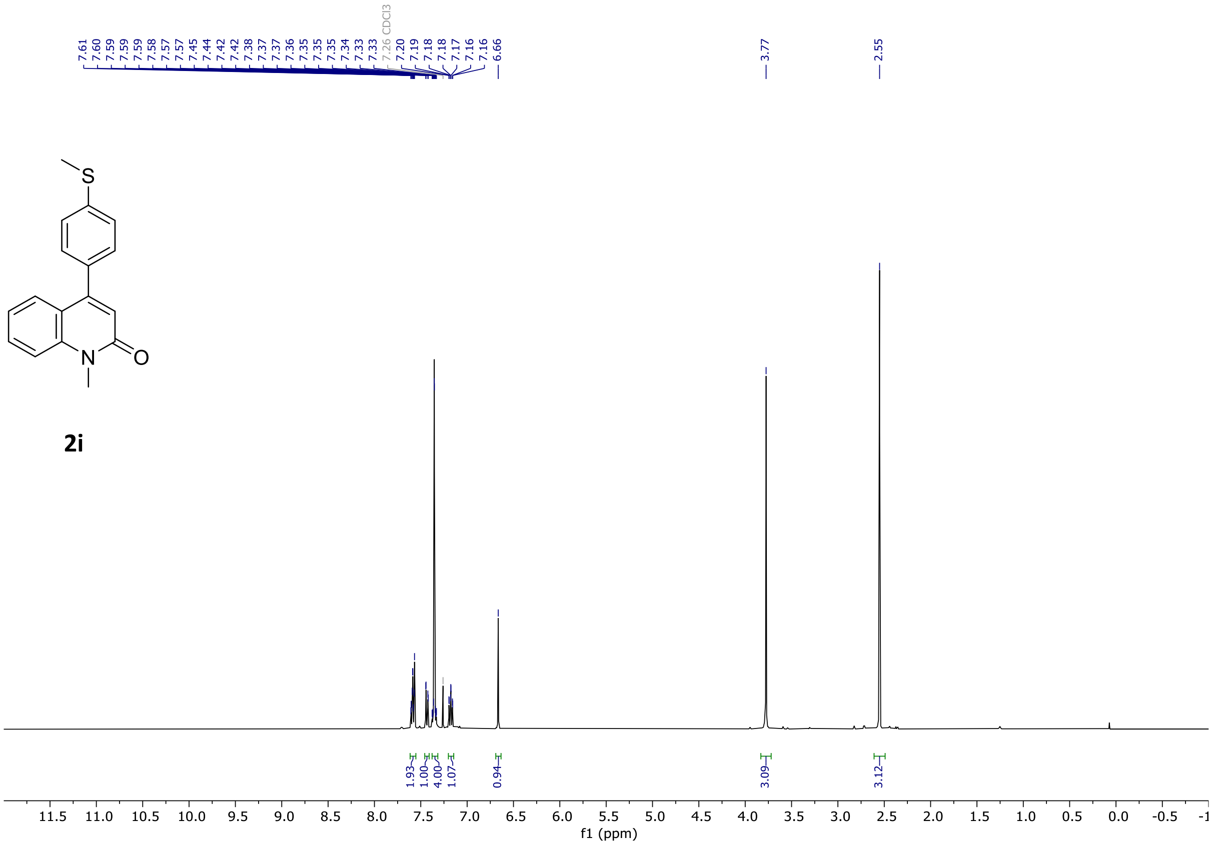
7.61
7.60
7.59
7.59
7.59
7.58
7.57
7.57
7.45
7.44
7.42
7.42
7.38
7.37
7.37
7.36
7.35
7.35
7.35
7.34
7.33
7.33
7.26 CDCl3
7.20
7.19
7.18
7.18
7.17
7.16
7.16
6.66
3.77
2.55
S
N
O
1.93
1.00
4.00
1.07
0.94
3.09
3.12
f1 (ppm)

2i

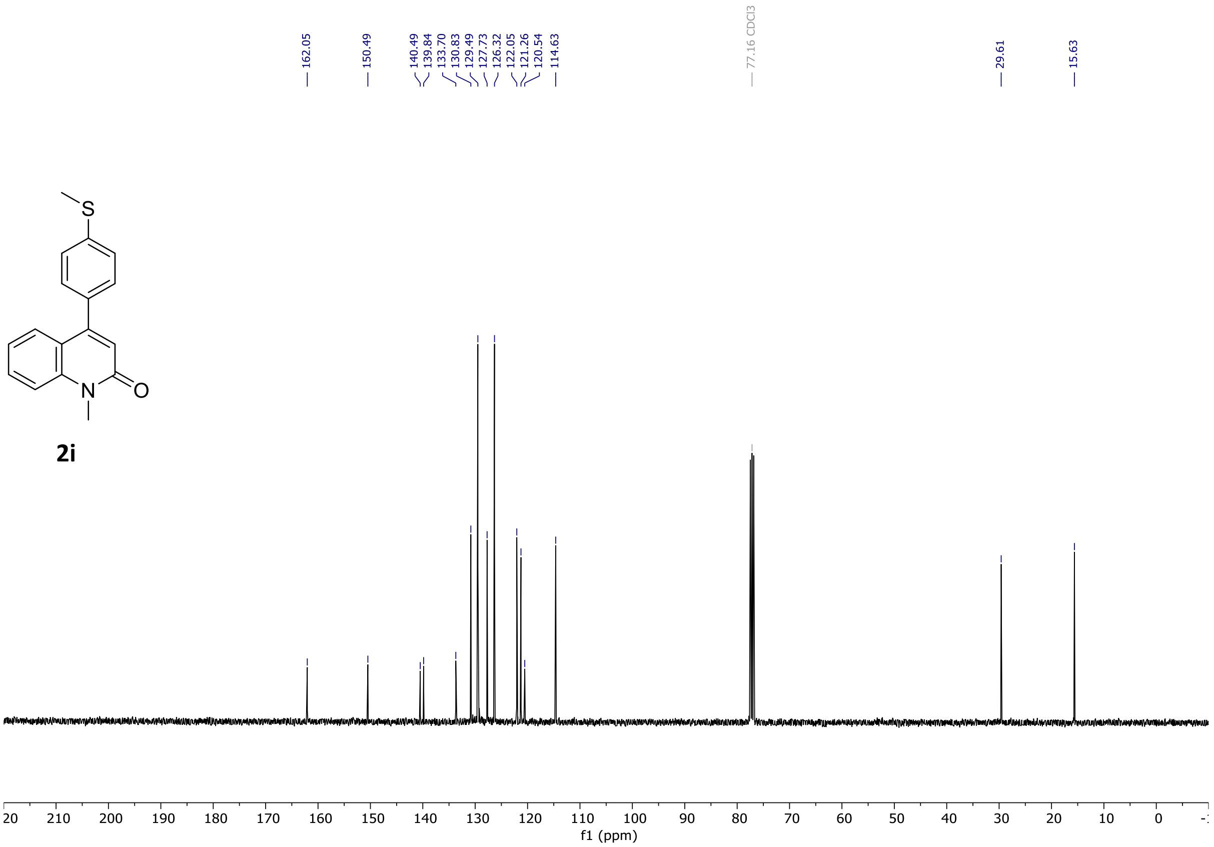
162.05
150.49
140.49
139.84
133.70
130.83
129.49
127.73
126.32
122.05
121.26
120.54
114.63
77.16 CDCl3
29.61
15.63
S
N
O
2i
f1 (ppm)

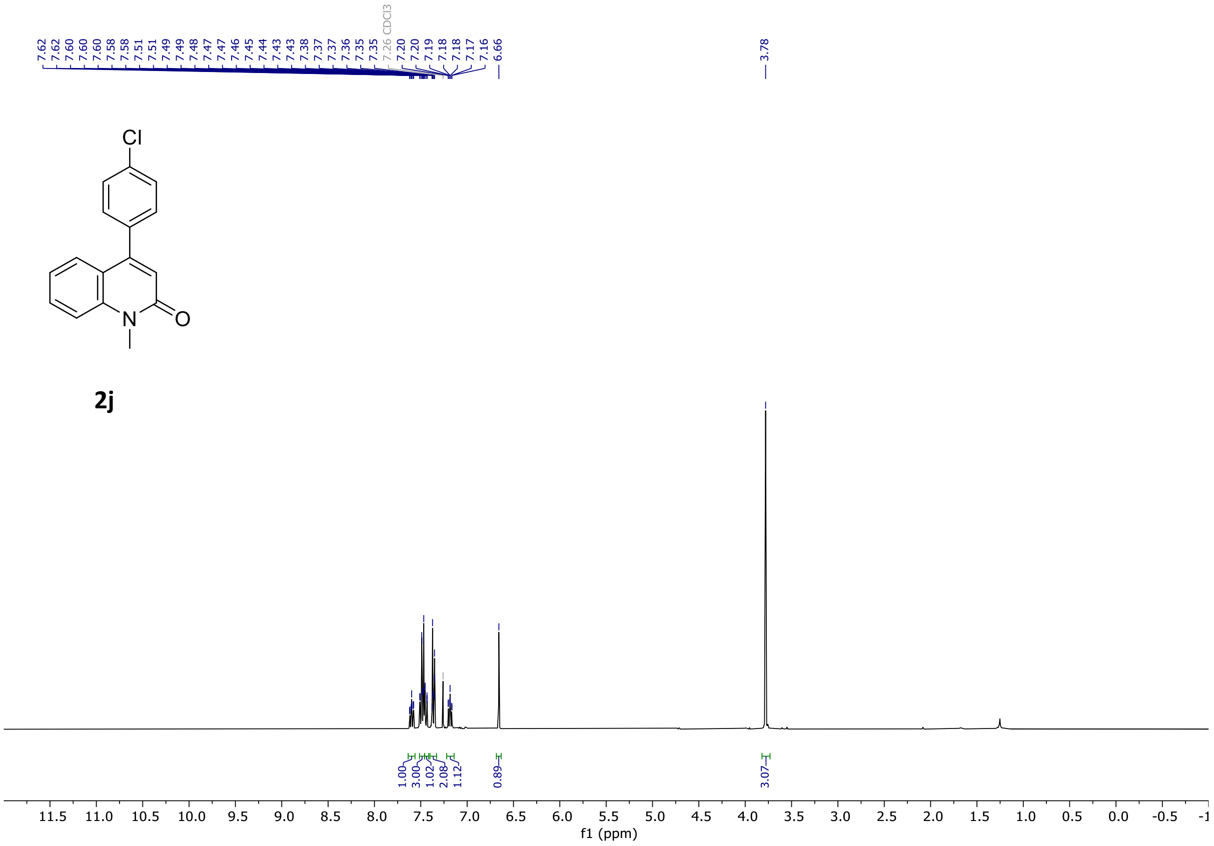
7.62
7.62
7.60
7.60
7.58
7.58
7.51
7.49
7.49
7.48
7.47
7.47
7.46
7.45
7.44
7.43
7.43
7.38
7.37
7.37
7.36
7.35
7.35
7.26 CDCl3
7.20
7.20
7.19
7.18
7.18
7.17
7.16
6.66
3.78
Cl
N
O
2j
1.00
3.00
1.02
2.08
1.12
0.89
3.07
f1 (ppm)

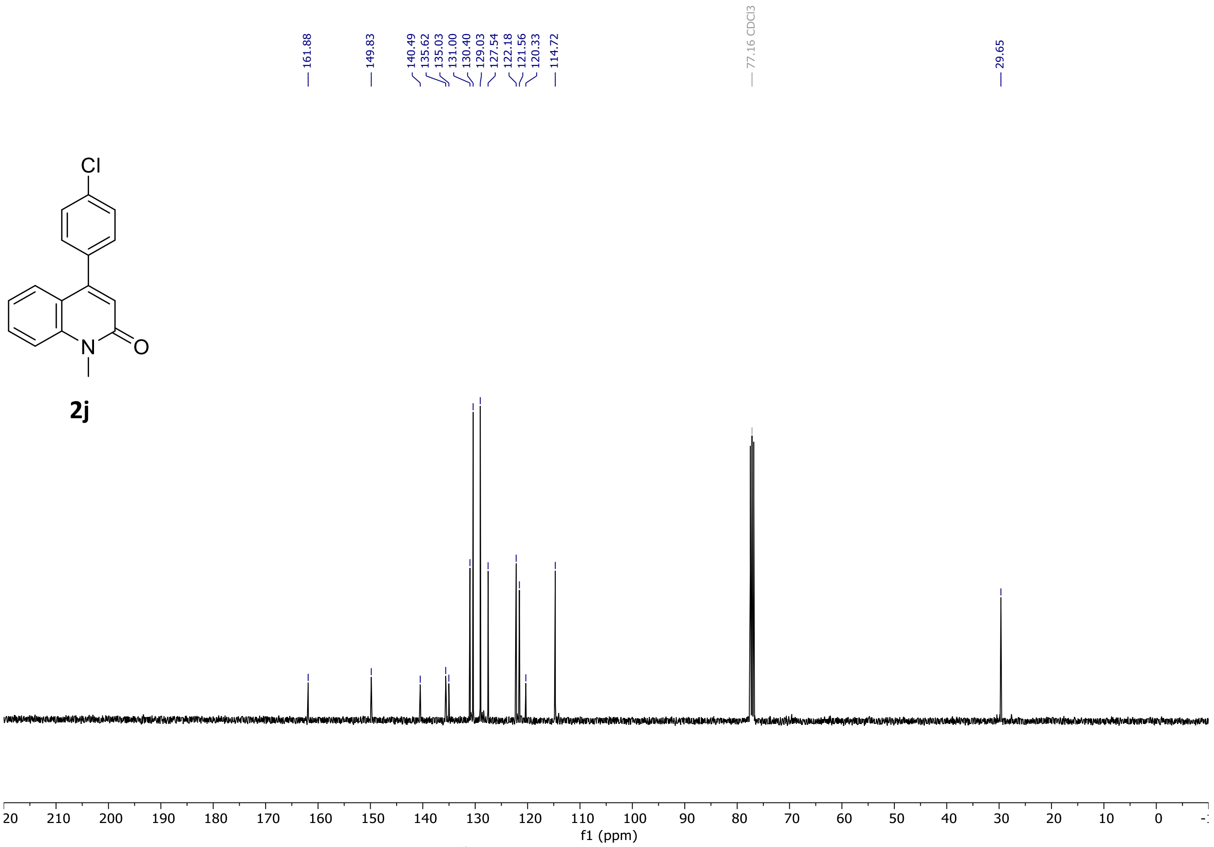
161.88
149.83
140.49
135.62
135.03
131.00
130.40
129.03
127.54
122.18
121.56
120.33
114.72
77.16 CDCl3
29.65
Cl
N
O
20 210 200 190 180 170 160 150 140 130 120 110 100 90 80 70 60 50 40 30 20 10 0
f1 (ppm)

2j

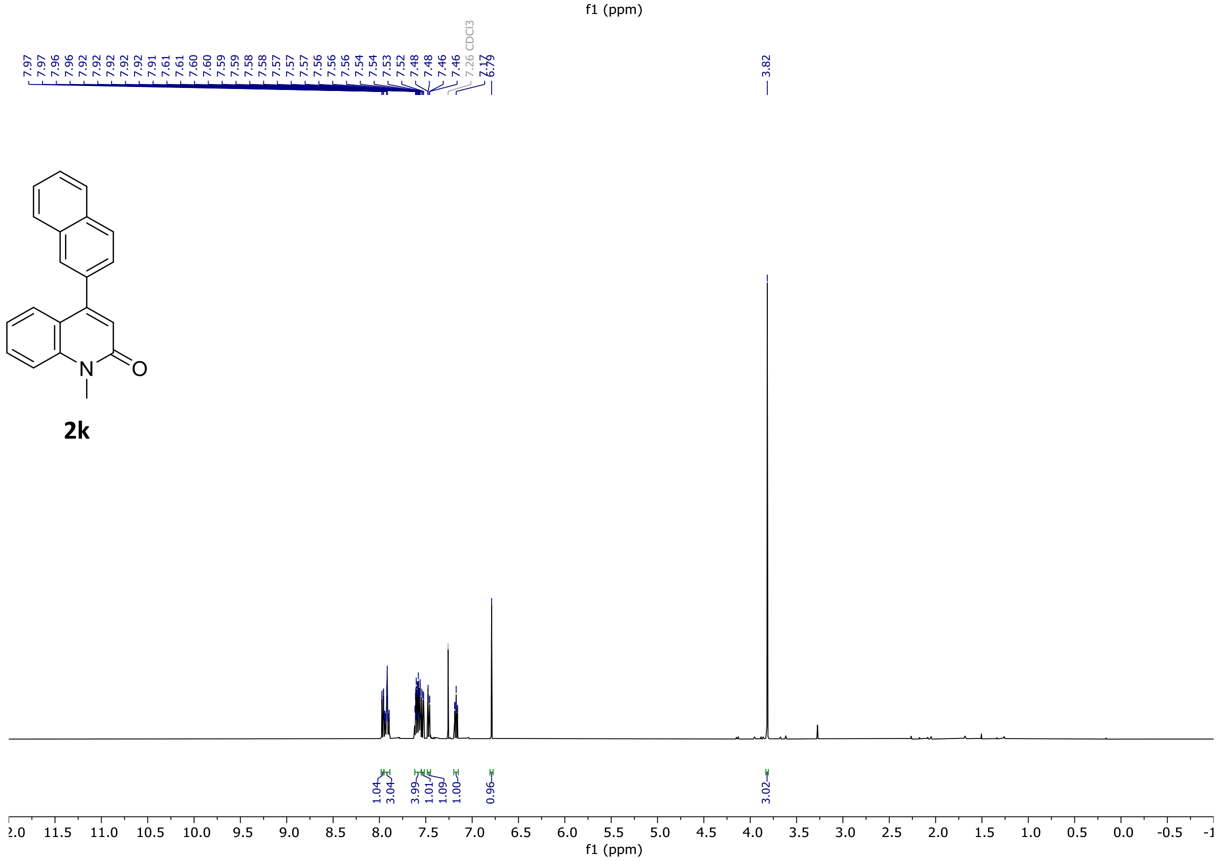
7.97
7.97
7.96
7.96
7.92
7.92
7.92
7.92
7.92
7.91
7.61
7.61
7.60
7.60
7.59
7.59
7.58
7.58
7.57
7.57
7.57
7.56
7.56
7.56
7.54
7.54
7.53
7.52
7.48
7.48
7.46
7.26 CDCl3
7.17
6.79
3.82
N
O
1.04
3.04
3.99
1.01
1.09
1.00
0.96
3.02
11.5 11.0 10.5 10.0 9.5 9.0 8.5 8.0 7.5 7.0 6.5 6.0 5.5 5.0 4.5 4.0 3.5 3.0 2.5 2.0 1.5 1.0 0.5 0.0 -0.5
f1 (ppm)

2k

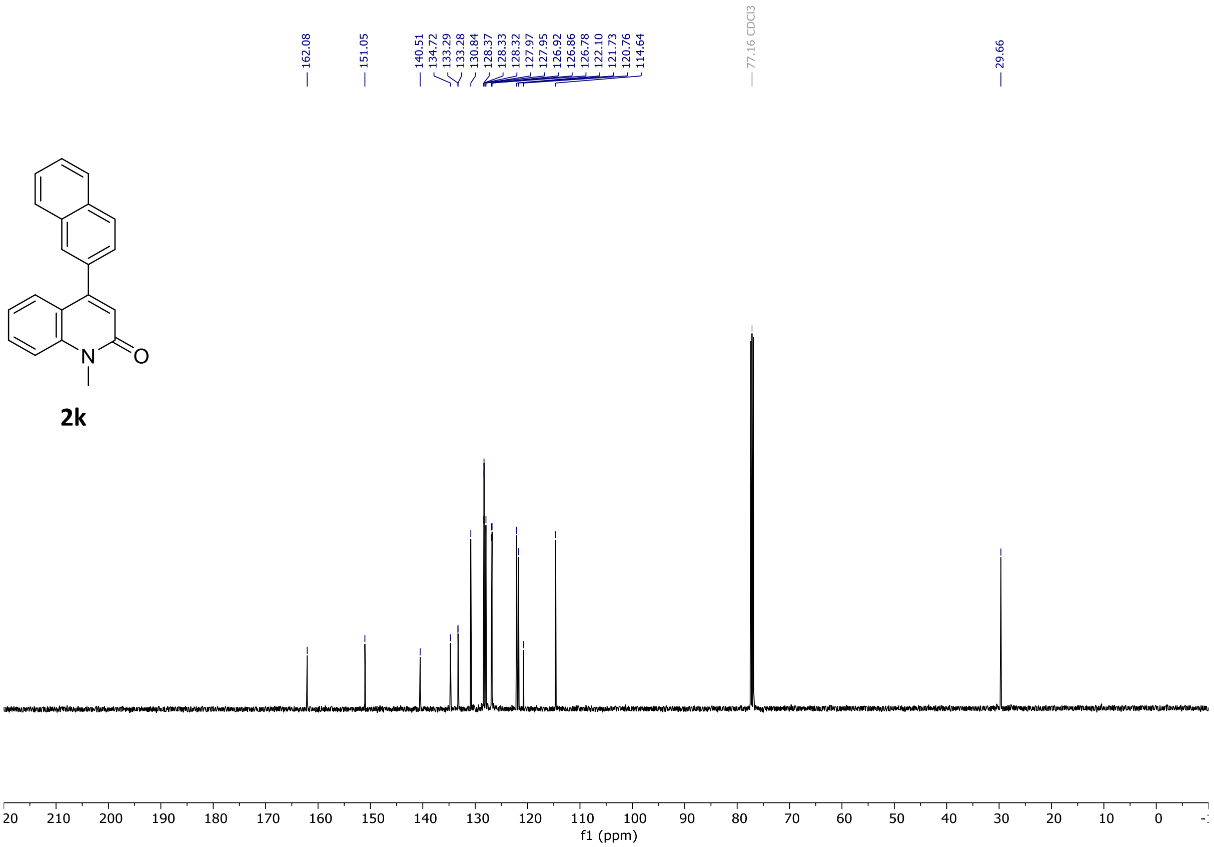

162.08
151.05
140.51
134.72
133.29
133.28
130.84
128.37
128.33
128.32
127.97
127.95
126.92
126.86
126.78
122.10
121.73
120.76
114.64
77.16 CDCl3
29.66
N
O
f1 (ppm)

2k

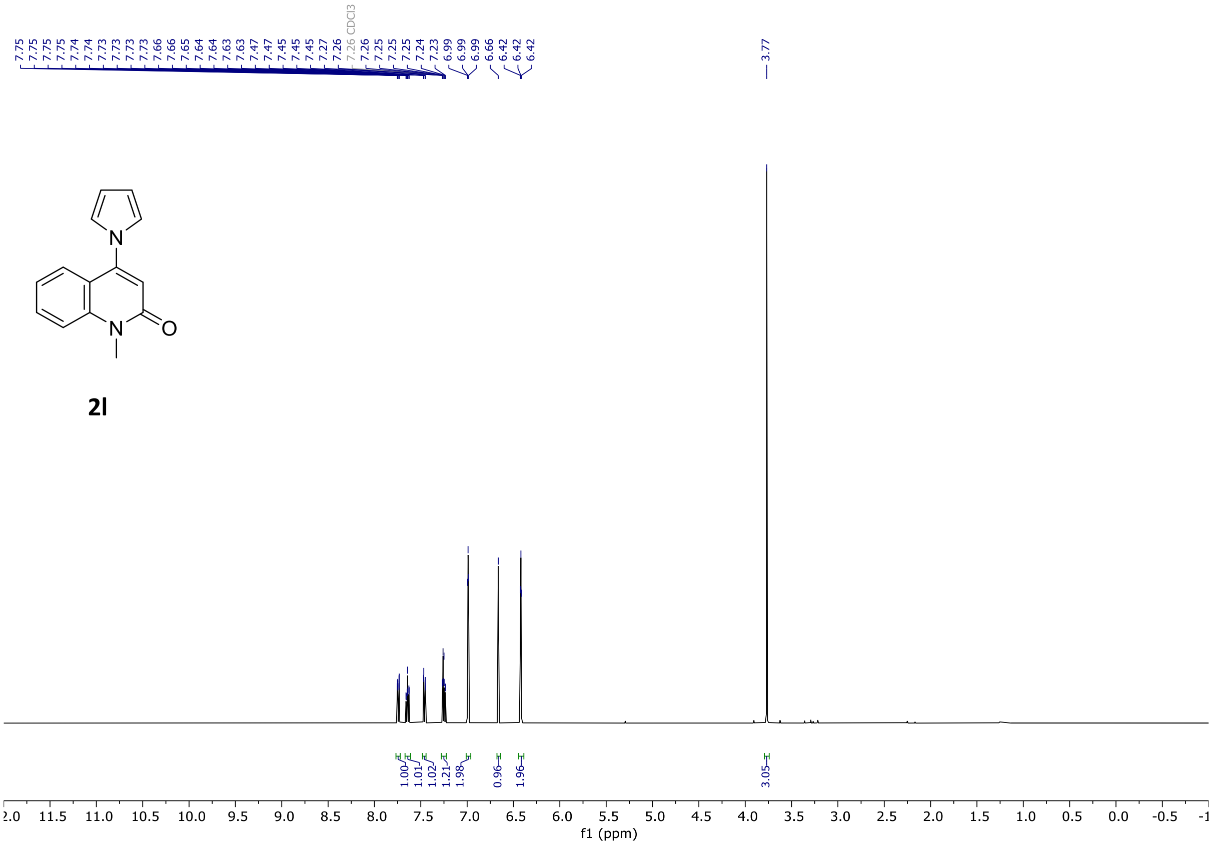

7.75
7.75
7.75
7.74
7.74
7.73
7.73
7.73
7.73
7.66
7.65
7.64
7.64
7.63
7.63
7.47
7.47
7.45
7.45
7.45
7.45
7.27
7.26
7.26 CDCl3
7.26
7.25
7.25
7.25
7.24
7.23
6.99
6.99
6.99
6.66
6.66
6.42
6.42
6.42
3.77
N
O
1.00
1.01
1.02
1.21
1.98
0.96
1.96
3.05
f1 (ppm)

2l

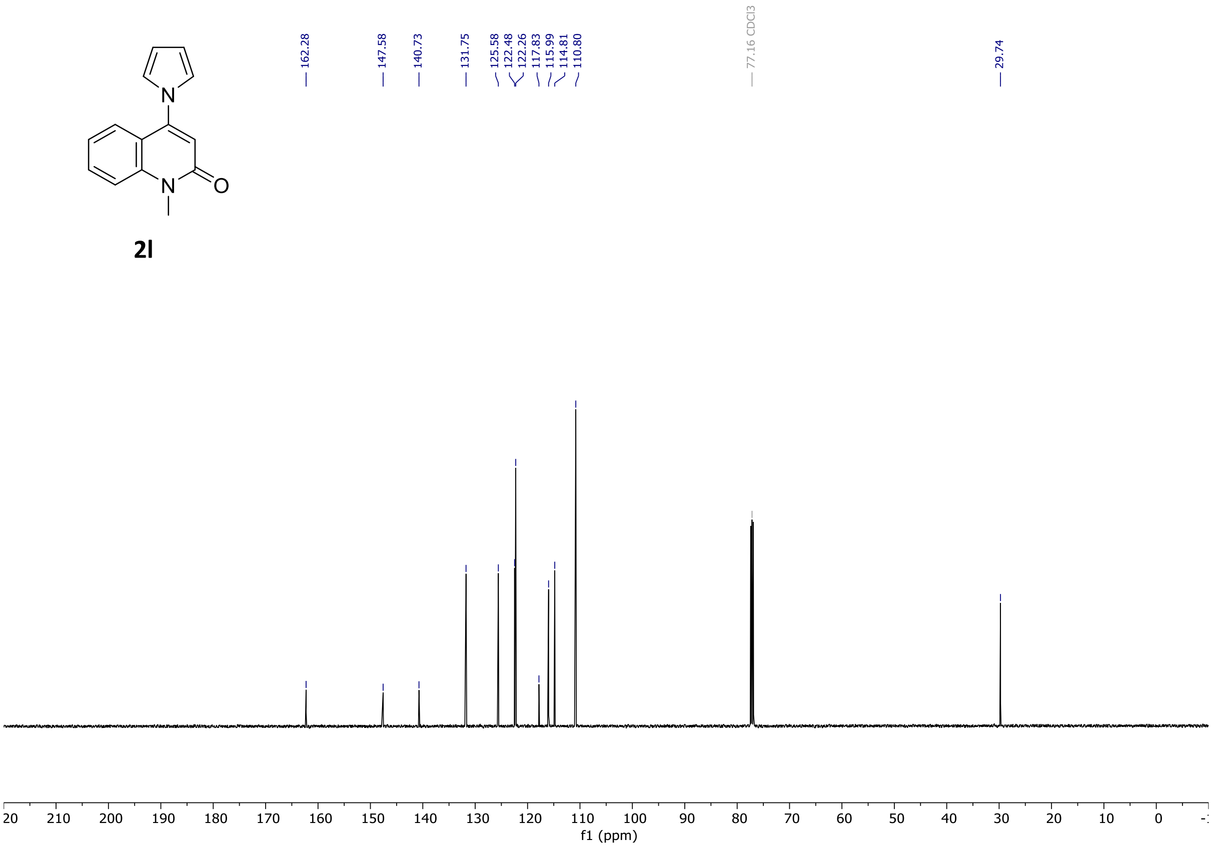
2l
162.28
147.58
140.73
131.75
125.58
122.48
122.26
117.83
115.99
114.81
110.80
77.16 CDCl3
29.74
f1 (ppm)

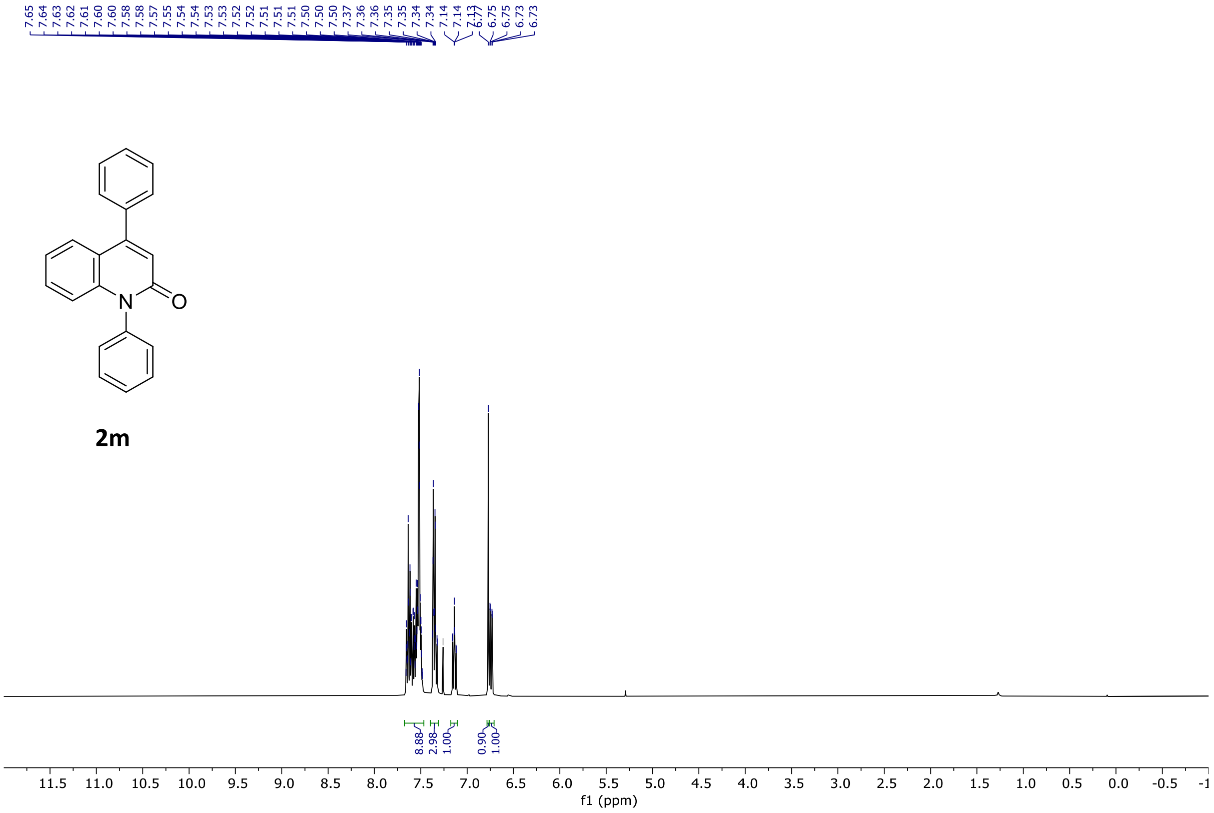
2m
7.65 7.64 7.63 7.62 7.61 7.60 7.60 7.58 7.58 7.57 7.55 7.54 7.54 7.53 7.53 7.52 7.52 7.51 7.51 7.50 7.50 7.50 7.37 7.36 7.36 7.35 7.35 7.34 7.34 7.14 7.14 7.13 6.77 6.75 6.75 6.73 6.73
8.88
2.98
1.00
0.90
1.00
f1 (ppm)

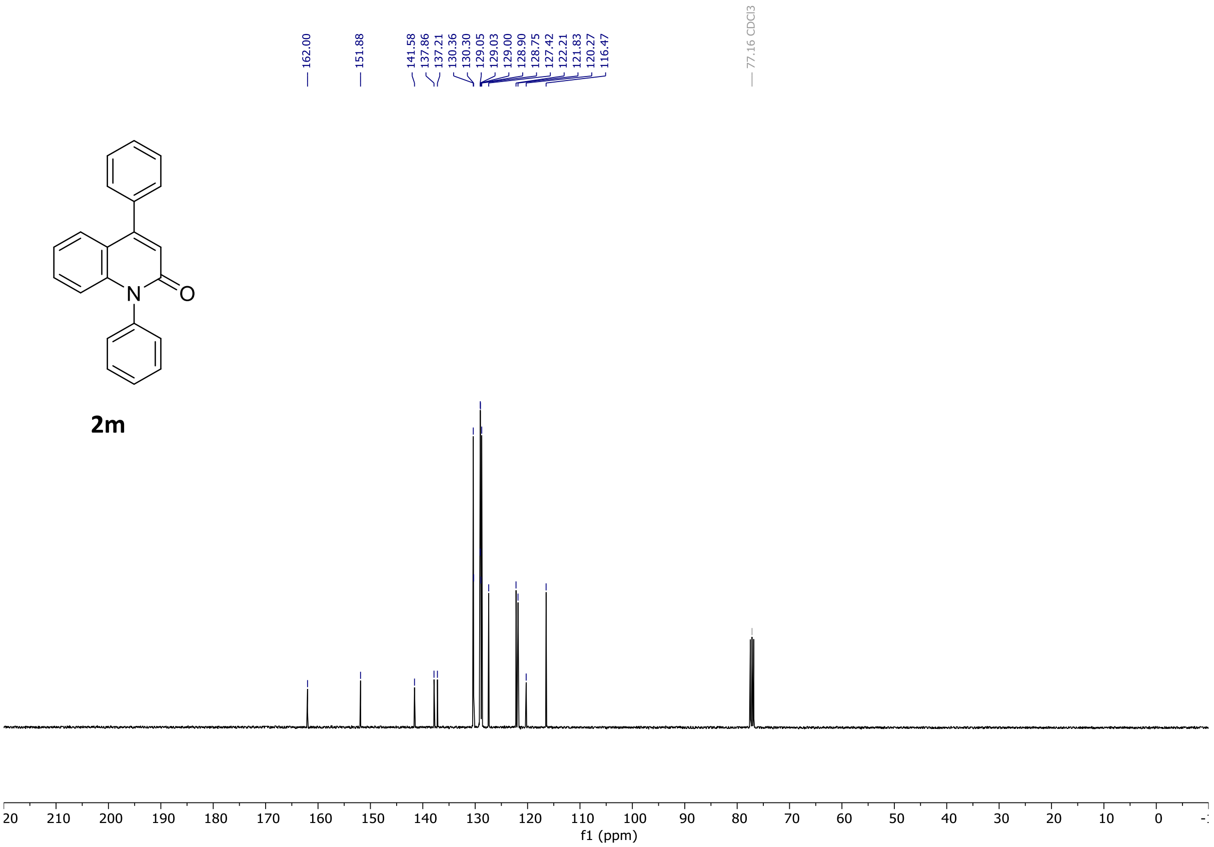
162.00
151.88
141.58
137.86
137.21
130.36
130.30
129.05
129.03
129.00
128.90
128.75
127.42
122.21
121.83
120.27
116.47
77.16 CDCl3
f1 (ppm)

2m

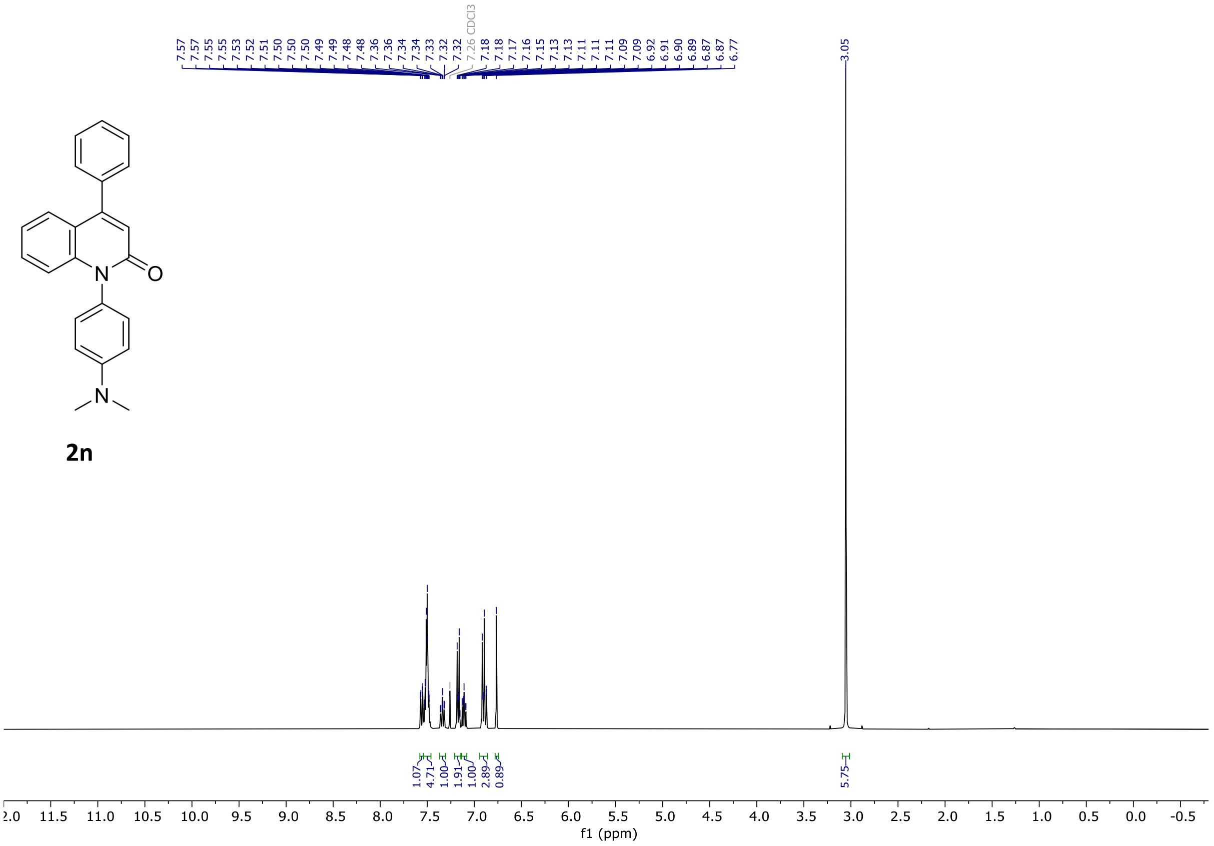
7.57
7.57
7.55
7.55
7.53
7.52
7.51
7.50
7.50
7.50
7.49
7.49
7.48
7.48
7.36
7.36
7.34
7.34
7.33
7.32
7.32
7.26 CDCl3
7.18
7.18
7.17
7.16
7.15
7.13
7.13
7.11
7.11
7.11
7.09
6.92
6.91
6.90
6.89
6.87
6.87
6.77
3.05
1.07
4.71
1.00
1.91
1.00
2.89
0.89
5.75
f1 (ppm)

2n

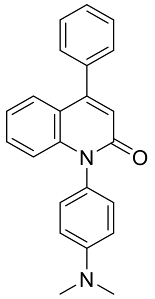

2n

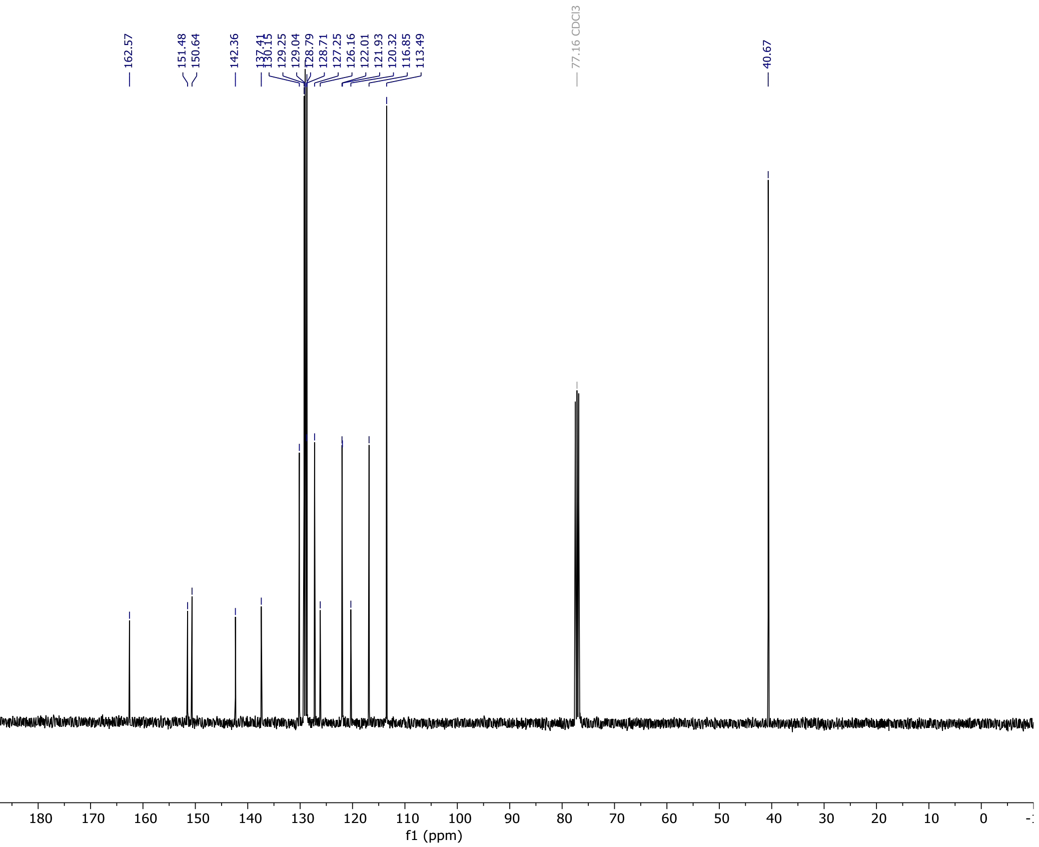
162.57
151.48
150.64
142.36
137.41
130.15
129.25
129.04
128.79
128.71
127.25
126.16
122.01
121.93
120.32
116.85
113.49
77.16 CDCl3
40.67
f1 (ppm)

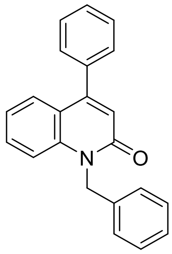

2o

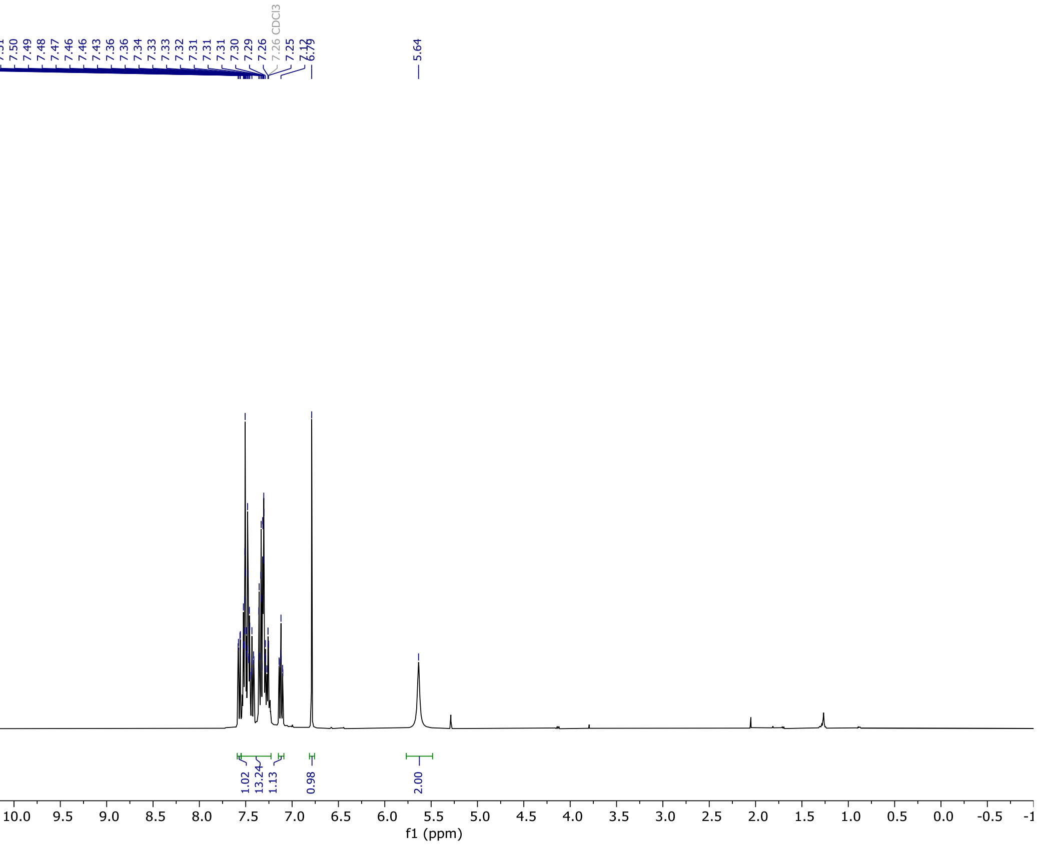
7.58
7.58
7.56
7.56
7.53
7.52
7.52
7.51
7.51
7.51
7.50
7.49
7.48
7.47
7.46
7.46
7.43
7.36
7.36
7.34
7.33
7.33
7.32
7.31
7.31
7.31
7.30
7.29
7.26 CDCl3
7.25
6.79
5.64
1.02
13.24
1.13
0.98
2.00
f1 (ppm)

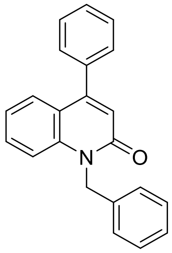

2o

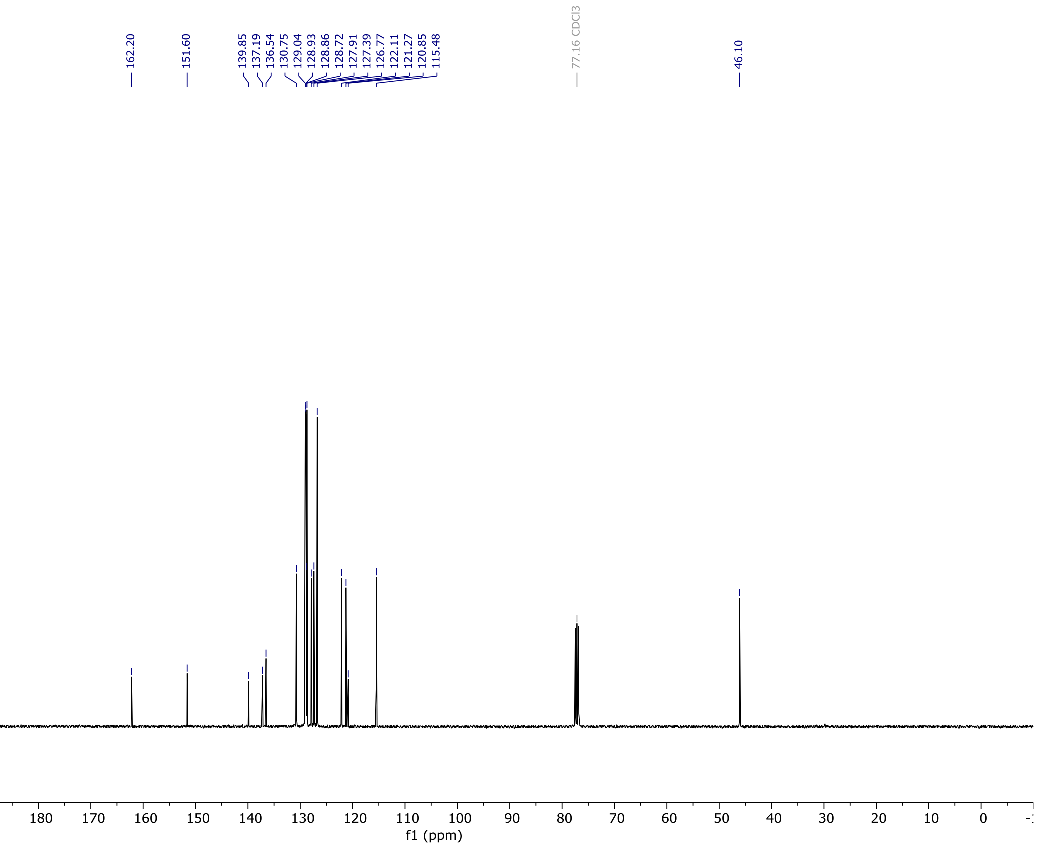
162.20
151.60
139.85
137.19
136.54
130.75
129.04
128.93
128.86
128.72
127.91
127.39
126.77
122.11
121.27
120.85
115.48
77.16 CDCl3
46.10
f1 (ppm)

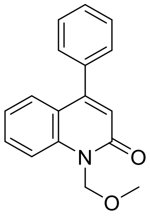

2p

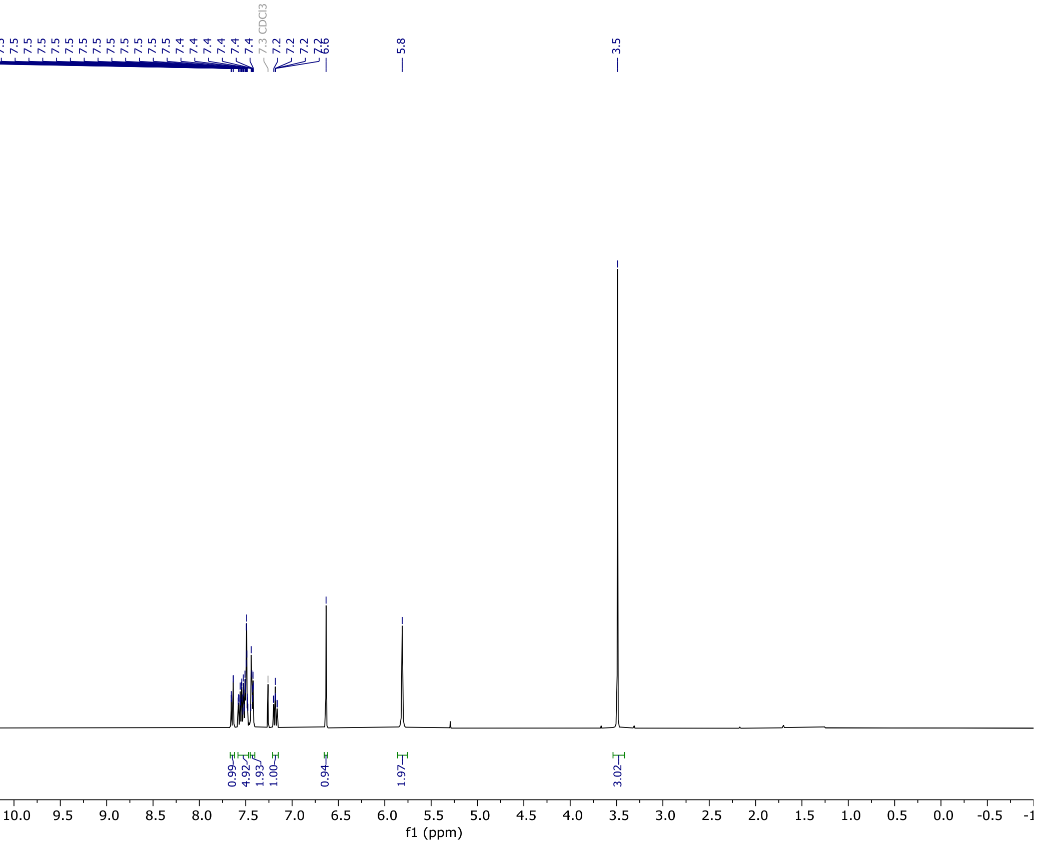
7.7 ×3, 7.6 ×4, 7.5 ×13, 7.4 ×6
7.3 CDCl3
7.2
7.2
7.2
7.2
6.6
5.8
3.5
0.99
4.92
1.93
1.00
0.94
1.97
3.02
f1 (ppm)

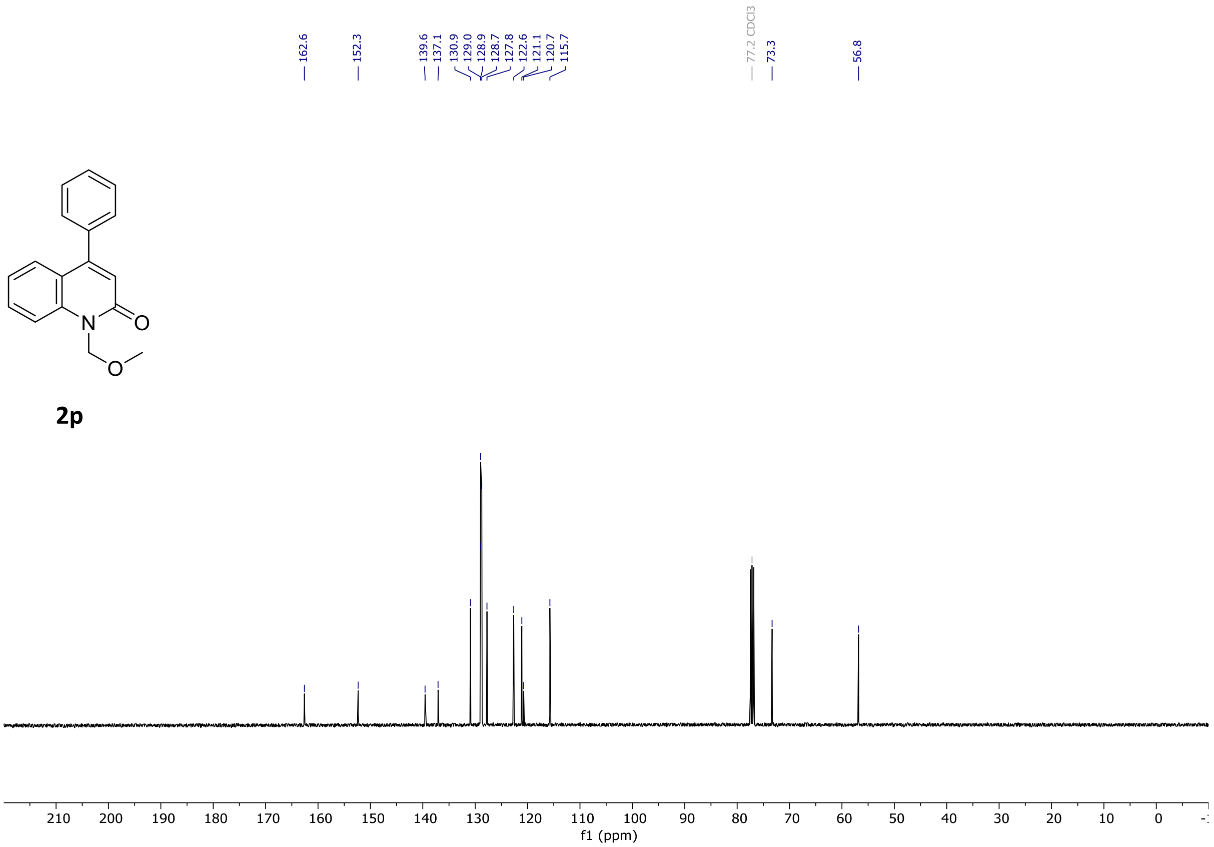
162.6
152.3
139.6
137.1
130.9
129.0
128.9
128.7
127.8
122.6
121.1
120.7
115.7
77.2 CDCl3
73.3
56.8
2p
f1 (ppm)

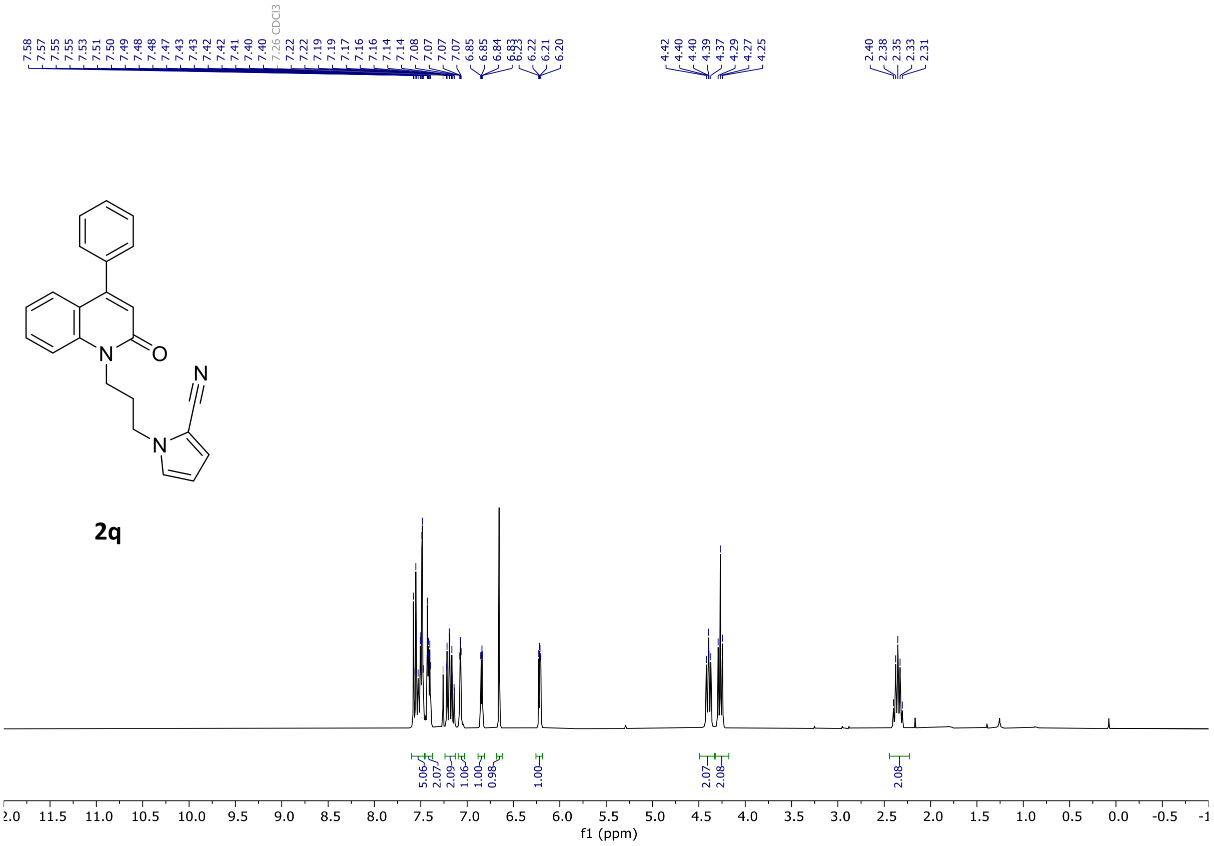
7.58
7.57
7.55
7.55
7.53
7.51
7.50
7.49
7.48
7.48
7.47
7.43
7.43
7.42
7.42
7.41
7.40
7.40
7.26 CDCl3
7.22
7.22
7.19
7.19
7.17
7.16
7.16
7.14
7.14
7.08
7.07
7.07
7.07
6.85
6.85
6.84
6.83
6.22
6.21
6.20
4.42
4.40
4.40
4.39
4.37
4.29
4.27
4.25
2.40
2.38
2.35
2.33
2.31
2q
5.06
2.07
2.09
1.06
1.00
0.98
1.00
2.07
2.08
2.08
f1 (ppm)

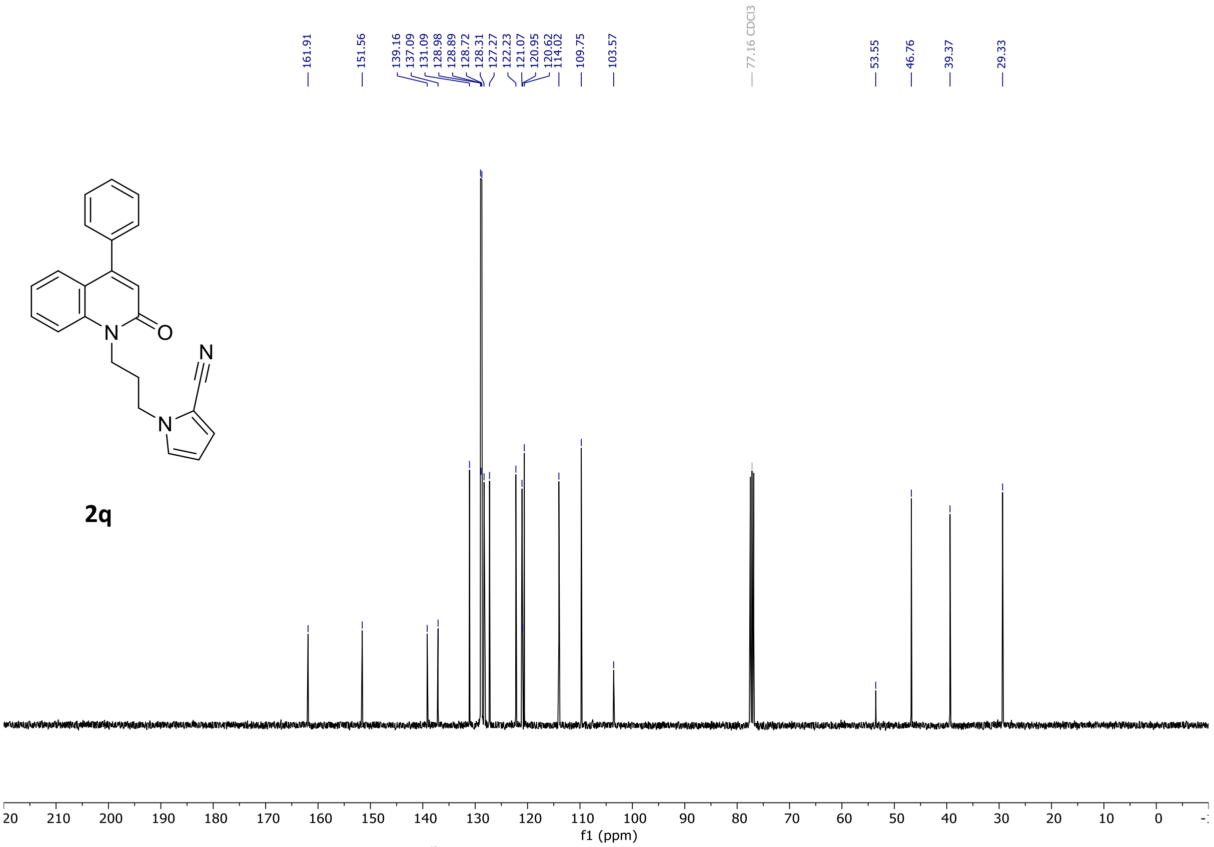

2q
161.91
151.56
139.16
137.09
131.09
128.98
128.89
128.72
128.31
127.27
122.23
121.07
120.95
120.62
114.02
109.75
103.57
77.16 CDCl3
53.55
46.76
39.37
29.33
f1 (ppm)

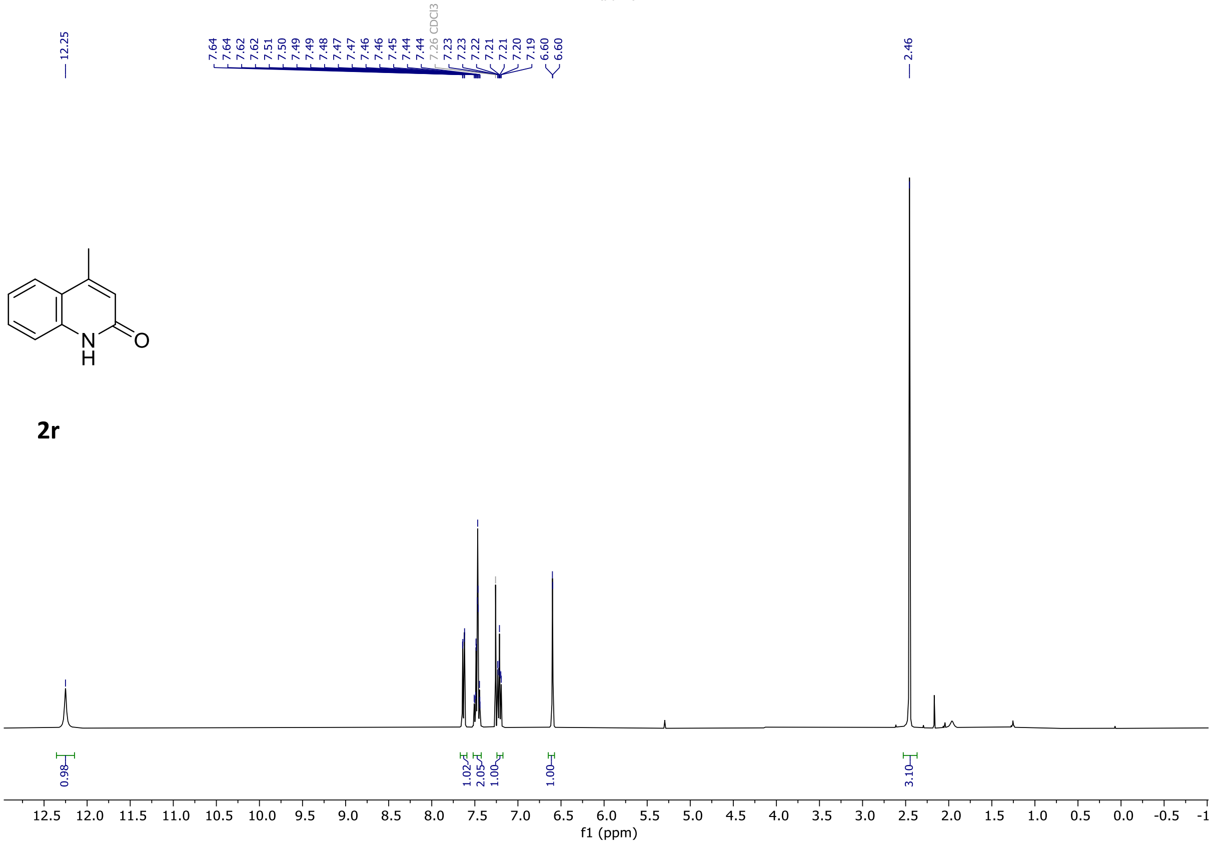

2r
12.25
7.64
7.64
7.62
7.51
7.50
7.49
7.49
7.48
7.47
7.47
7.46
7.45
7.44
7.44
7.26 CDCl3
7.23
7.23
7.22
7.21
7.21
7.20
7.19
6.60
6.60
2.46
0.98
1.02
2.05
1.00
1.00
3.10
f1 (ppm)

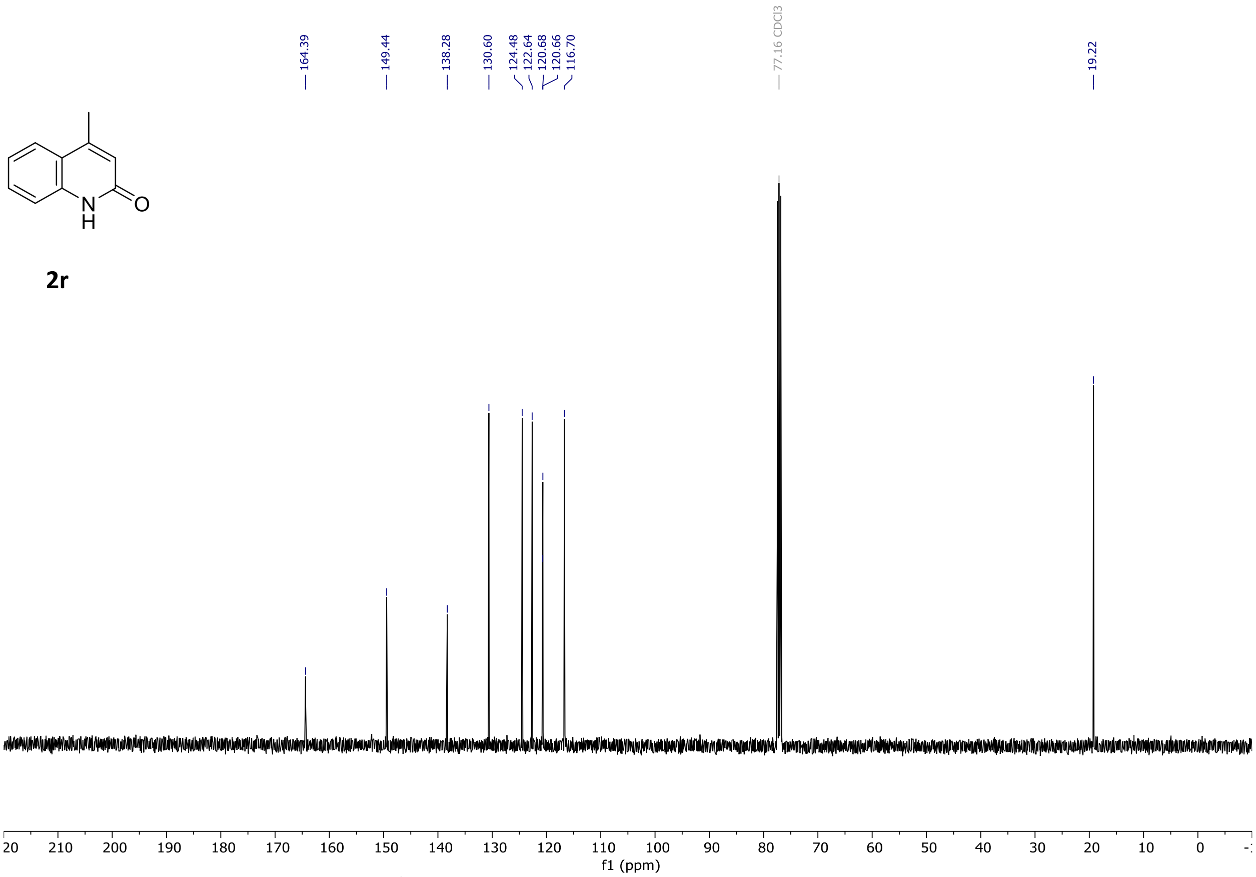
164.39
149.44
138.28
130.60
124.48
122.64
120.68
120.66
116.70
77.16 CDCl3
19.22
210 200 190 180 170 160 150 140 130 120 110 100 90 80 70 60 50 40 30 20 10 0
f1 (ppm)

2r

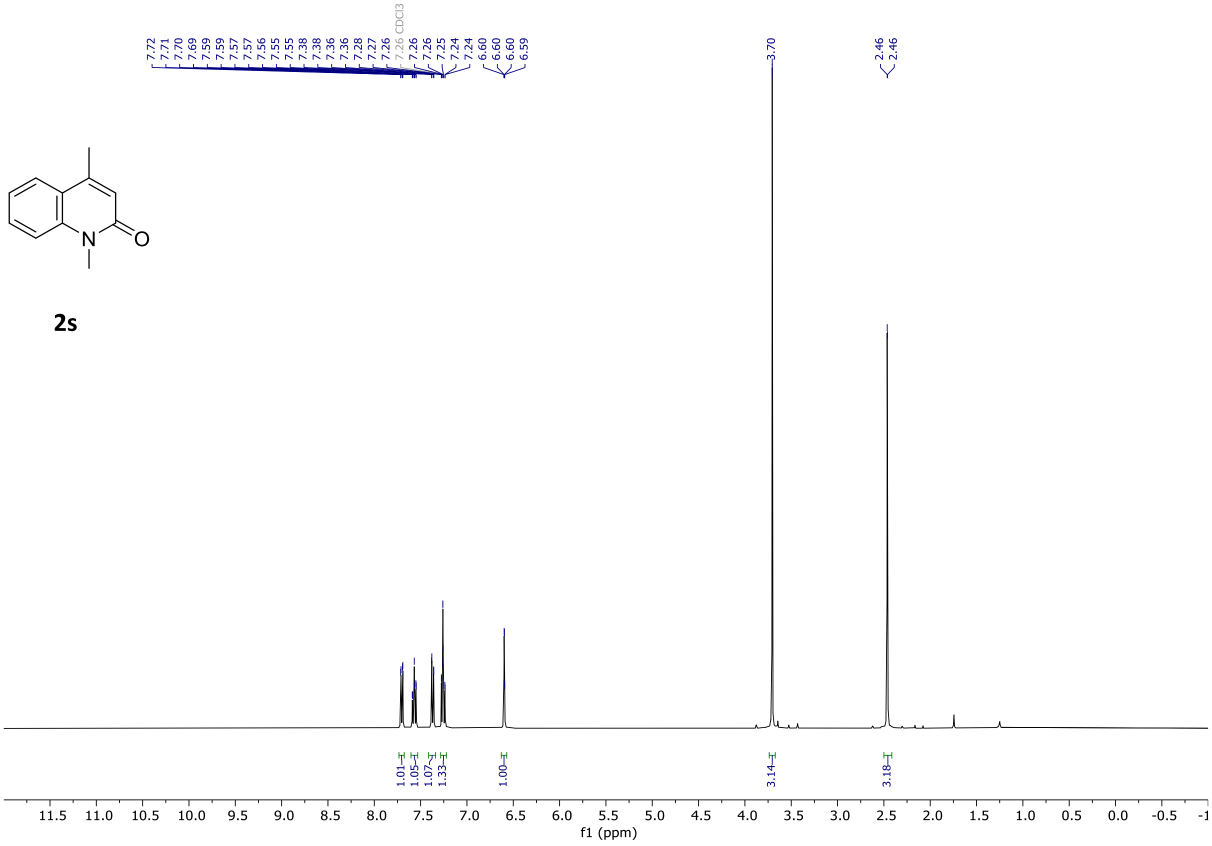
7.72
7.71
7.70
7.69
7.59
7.59
7.57
7.56
7.55
7.55
7.38
7.38
7.36
7.36
7.28
7.27
7.26 CDCl3
7.26
7.26
7.25
7.24
7.24
6.60
6.60
6.59
3.70
2.46
2.46
1.01
1.05
1.07
1.33
1.00
3.14
3.18
11.5 11.0 10.5 10.0 9.5 9.0 8.5 8.0 7.5 7.0 6.5 6.0 5.5 5.0 4.5 4.0 3.5 3.0 2.5 2.0 1.5 1.0 0.5 0.0 -0.5
f1 (ppm)

2s

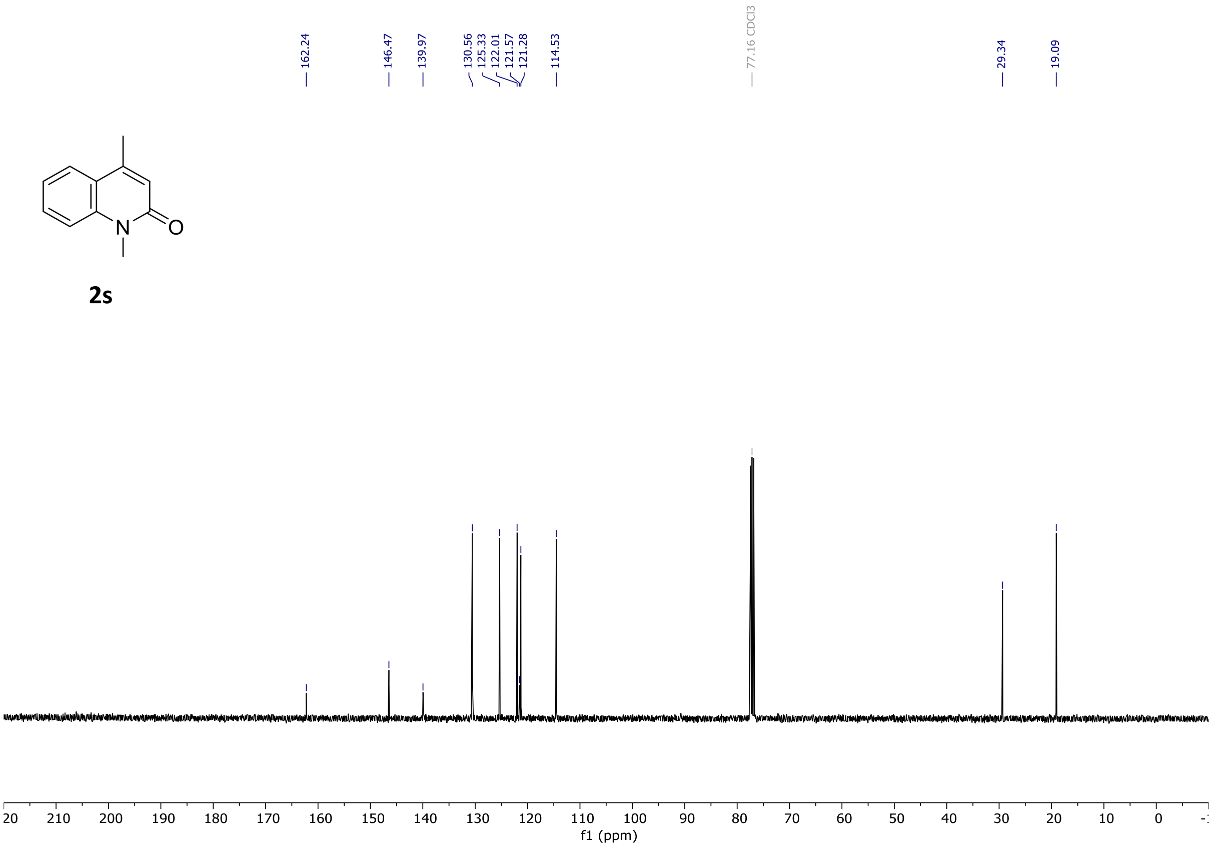
162.24
146.47
139.97
130.56
125.33
122.01
121.57
121.28
114.53
77.16 CDCl3
29.34
19.09
f1 (ppm)

2s

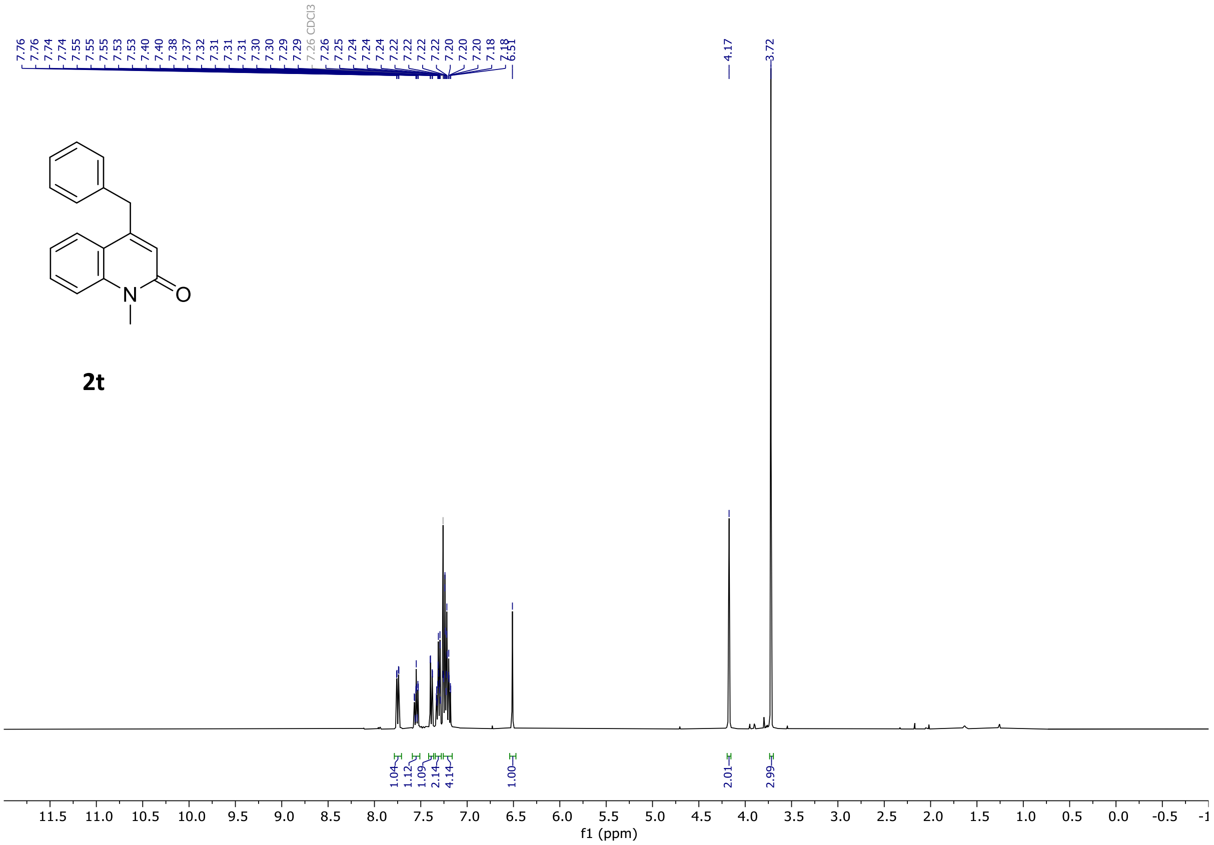
7.76
7.76
7.74
7.74
7.55
7.55
7.55
7.53
7.53
7.40
7.40
7.38
7.37
7.32
7.31
7.31
7.30
7.29
7.26 CDCl3
7.26
7.25
7.24
7.24
7.24
7.22
7.22
7.22
7.22
7.20
7.20
7.20
7.18
6.51
4.17
3.72
1.04
1.12
1.09
2.14
4.14
1.00
2.01
2.99
f1 (ppm)

2t

2t

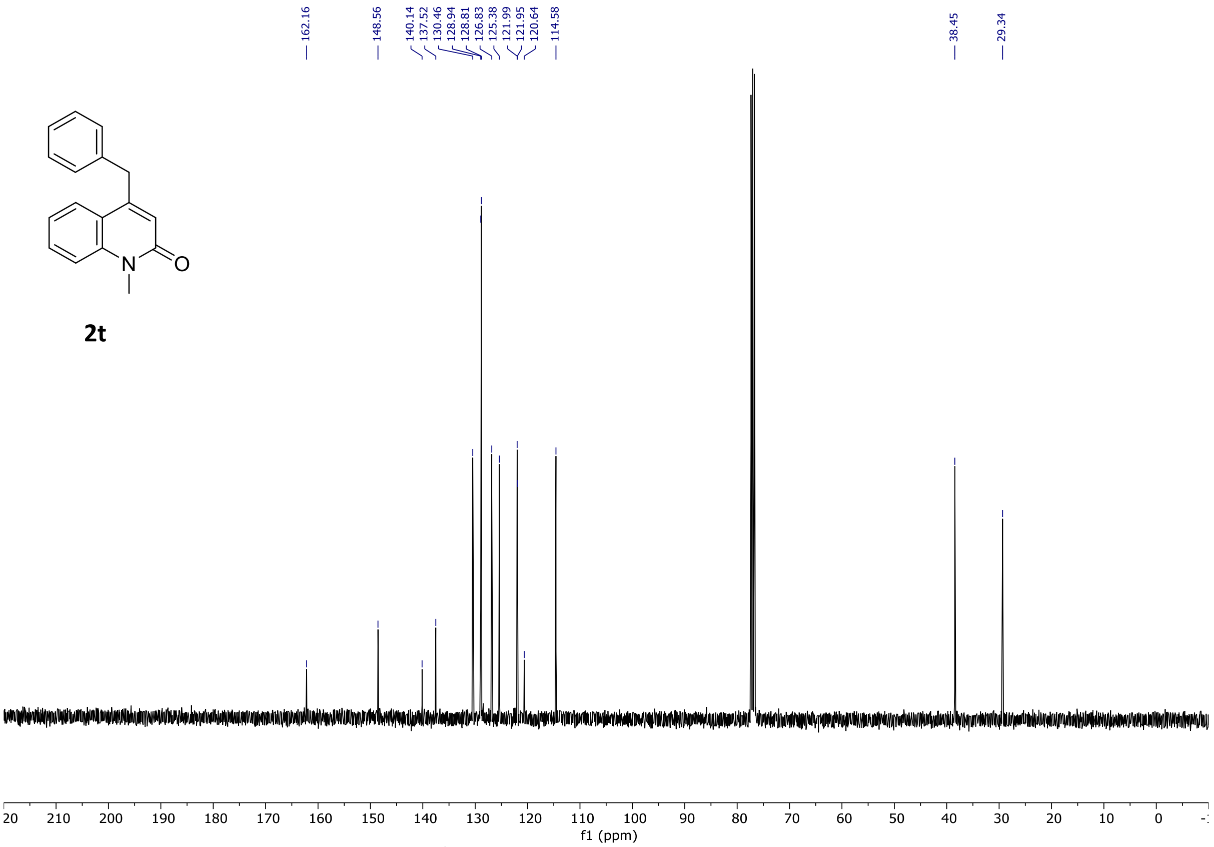
162.16
148.56
140.14
137.52
130.46
128.94
128.81
126.83
125.38
121.99
121.95
120.64
114.58
38.45
29.34
220 210 200 190 180 170 160 150 140 130 120 110 100 90 80 70 60 50 40 30 20 10 0
f1 (ppm)

2u

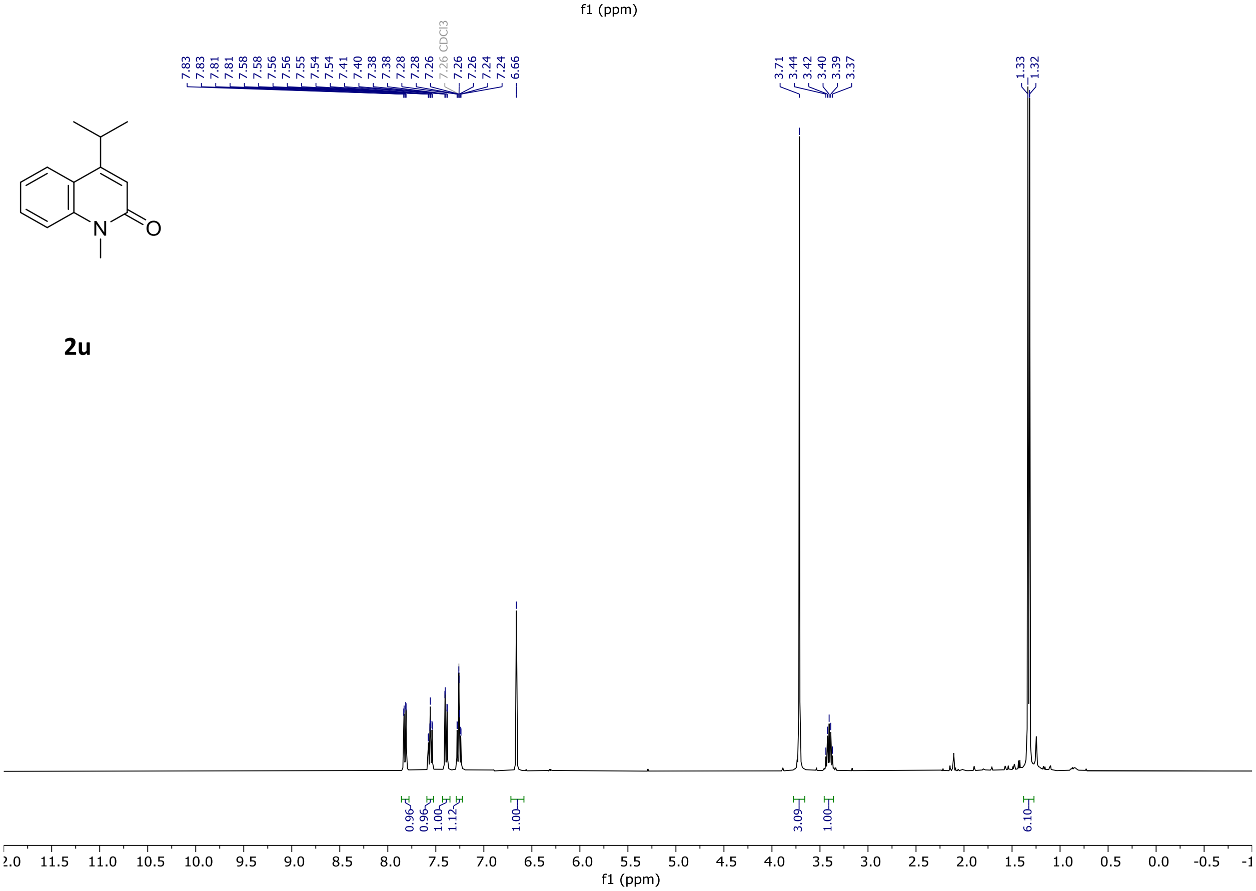
7.83
7.83
7.81
7.81
7.58
7.58
7.56
7.56
7.55
7.54
7.54
7.41
7.40
7.38
7.38
7.28
7.28
7.26 CDCl3
7.26
7.26
7.24
7.24
6.66
3.71
3.44
3.42
3.40
3.39
3.37
1.33
1.32
0.96
0.96
1.00
1.12
1.00
3.09
1.00
6.10
12.0 11.5 11.0 10.5 10.0 9.5 9.0 8.5 8.0 7.5 7.0 6.5 6.0 5.5 5.0 4.5 4.0 3.5 3.0 2.5 2.0 1.5 1.0 0.5 0.0 -0.5 -1
f1 (ppm)

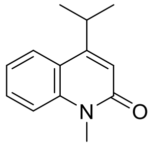

2u

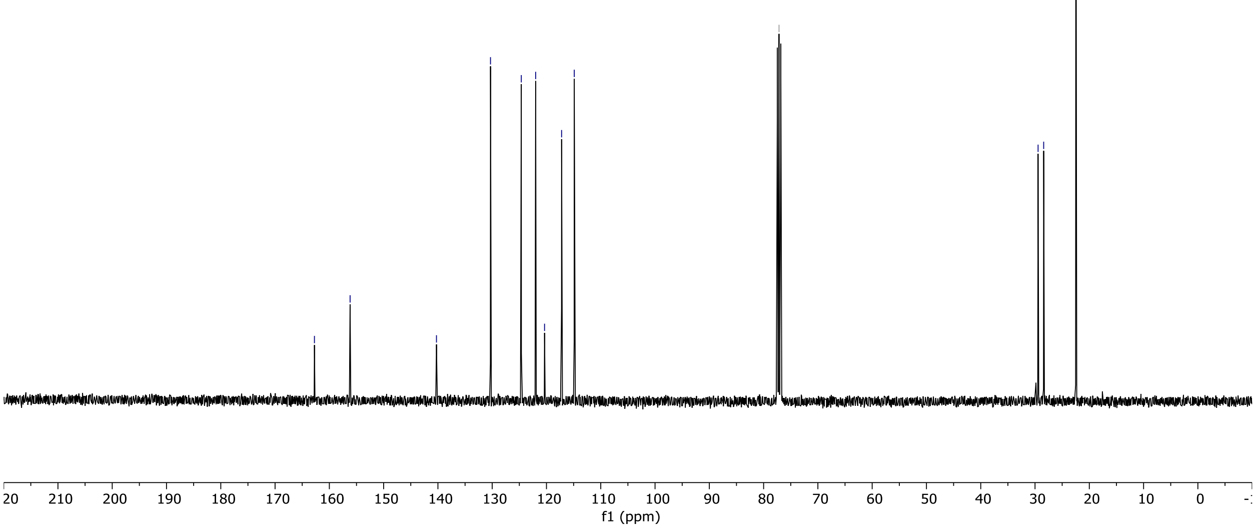

162.75
156.18
140.27
130.32
124.64
121.99
120.36
117.22
114.88
77.16 CDCl3
29.44
28.41
22.47
f1 (ppm)

Scope reaction B

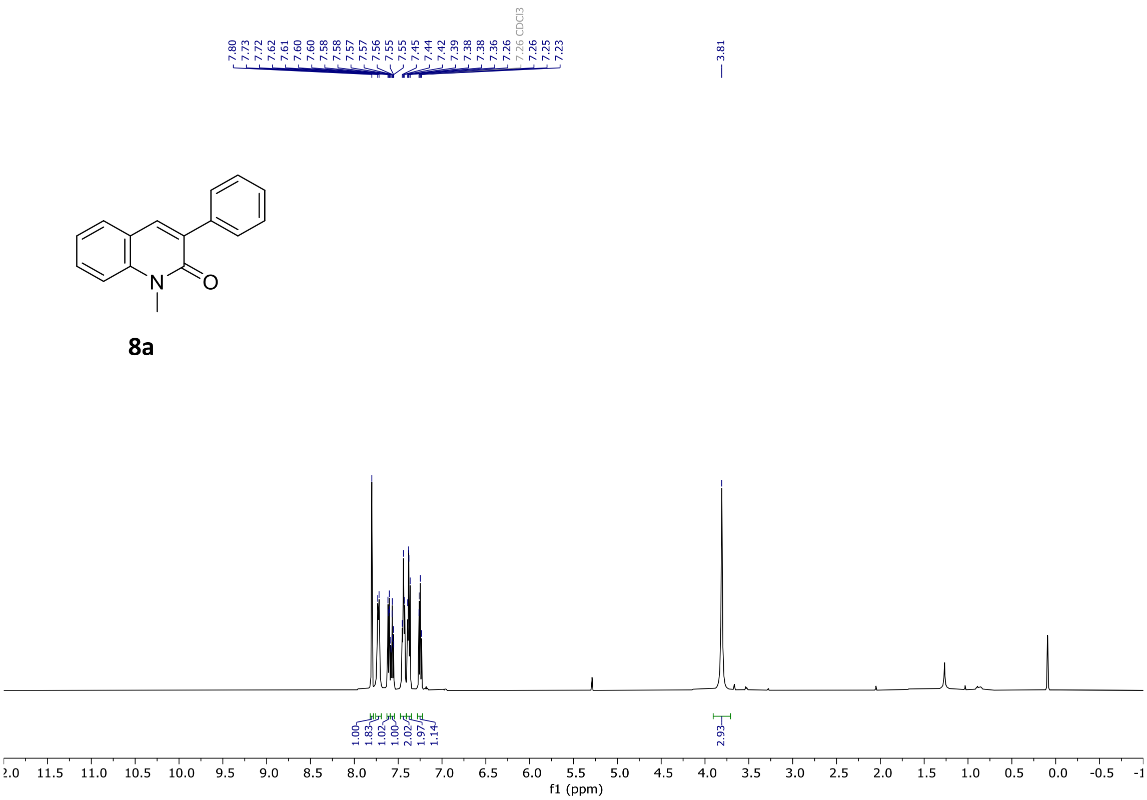

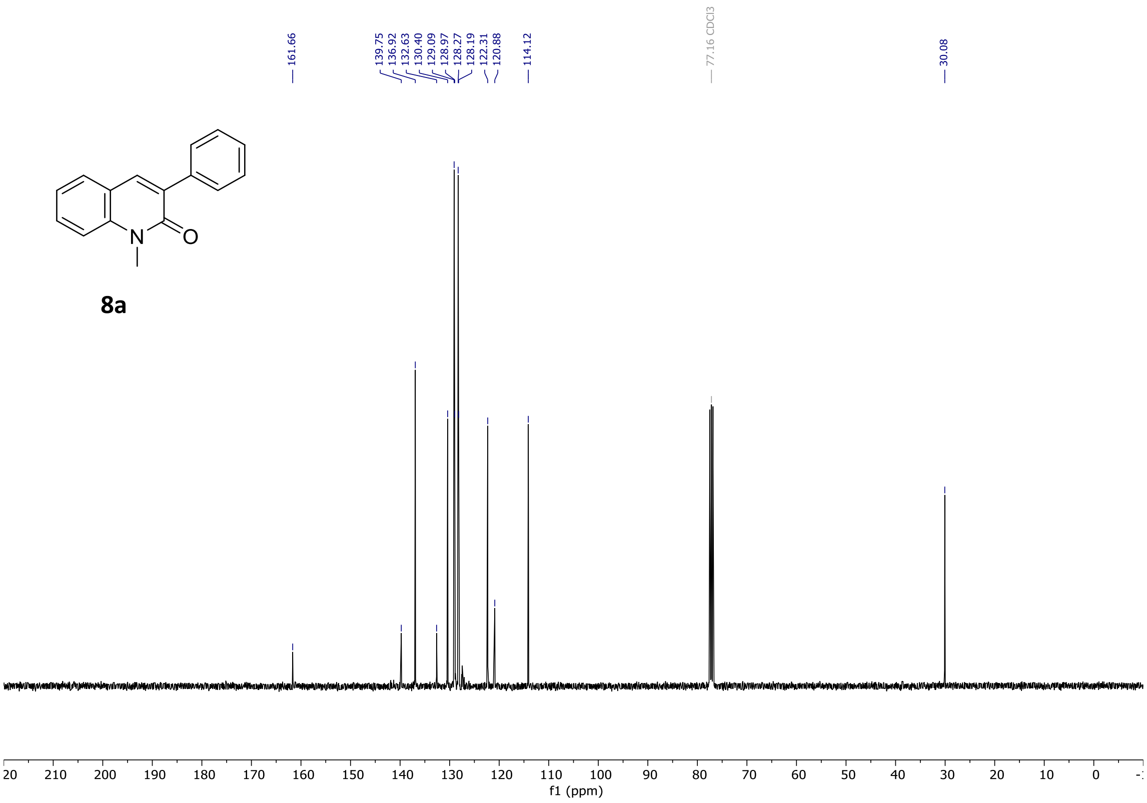

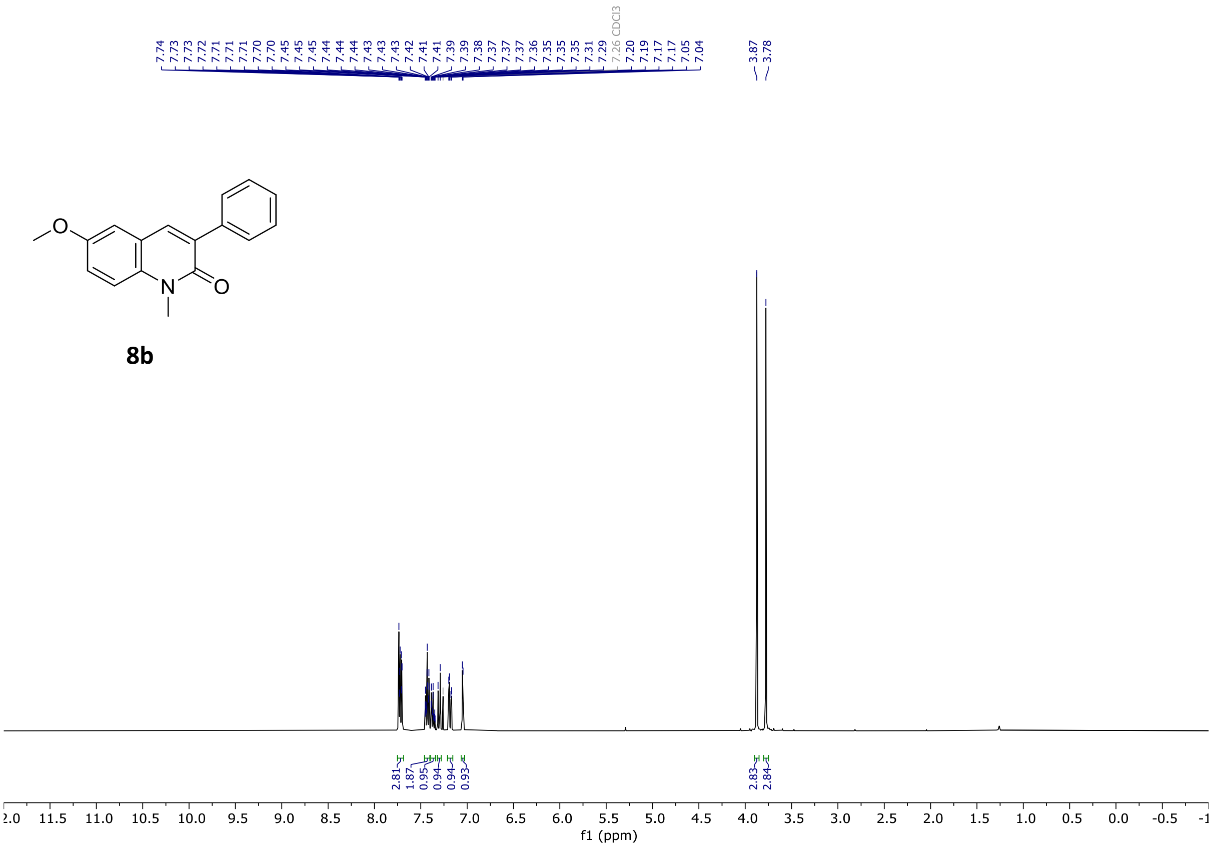
7.74 7.73 7.73 7.72 7.71 7.71 7.70 7.45 7.45 7.45 7.44 7.44 7.44 7.43 7.43 7.43 7.42 7.41 7.41 7.39 7.39 7.38 7.37 7.37 7.37 7.36 7.35 7.35 7.35 7.31 7.29 7.26 CDCl3 7.20 7.19 7.17 7.17 7.05 7.04
3.87 3.78
O
N
O
2.81 1.87 0.95 0.94 0.94 0.93
2.83 2.84
f1 (ppm)

8b

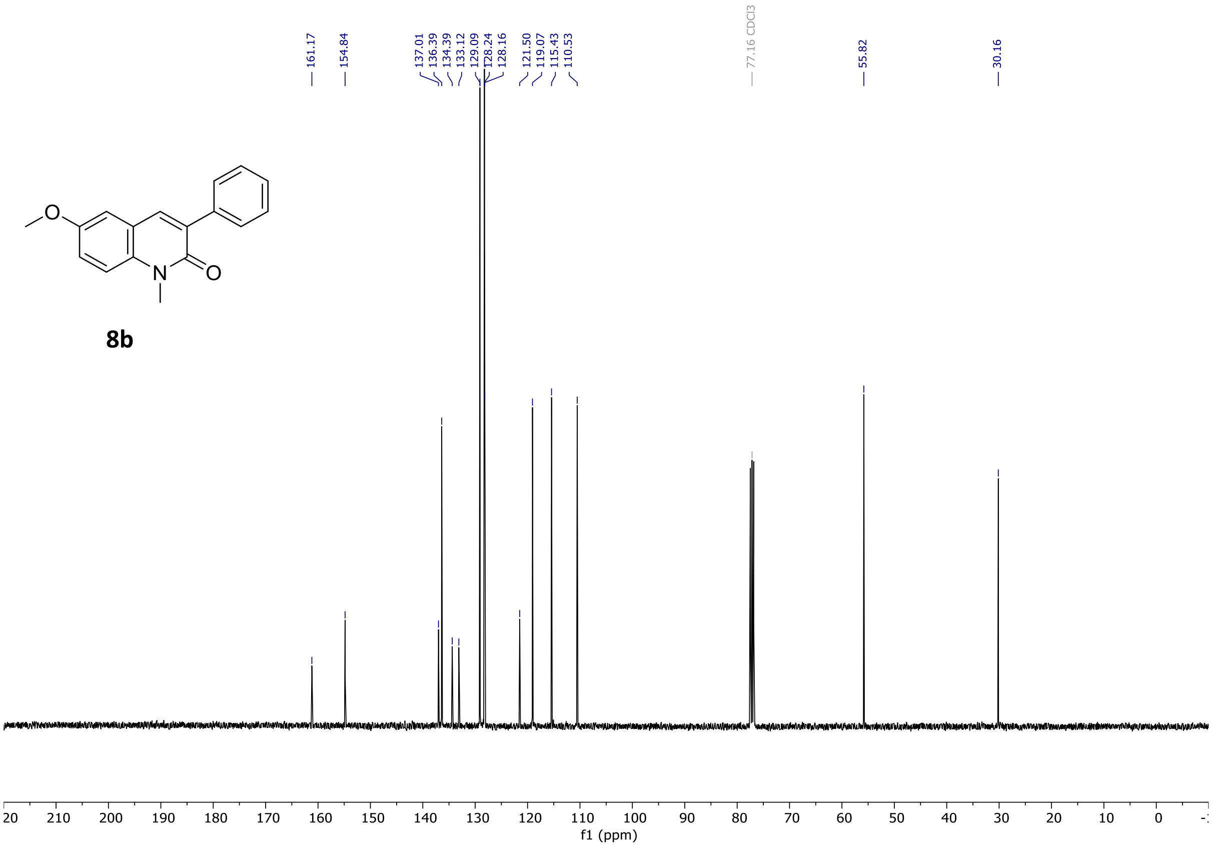
161.17 154.84
137.01 136.39 134.39 133.12 129.09 128.24 128.16
121.50 119.07 115.43 110.53
77.16 CDCl3
55.82
30.16
O
N
O
f1 (ppm)

8b

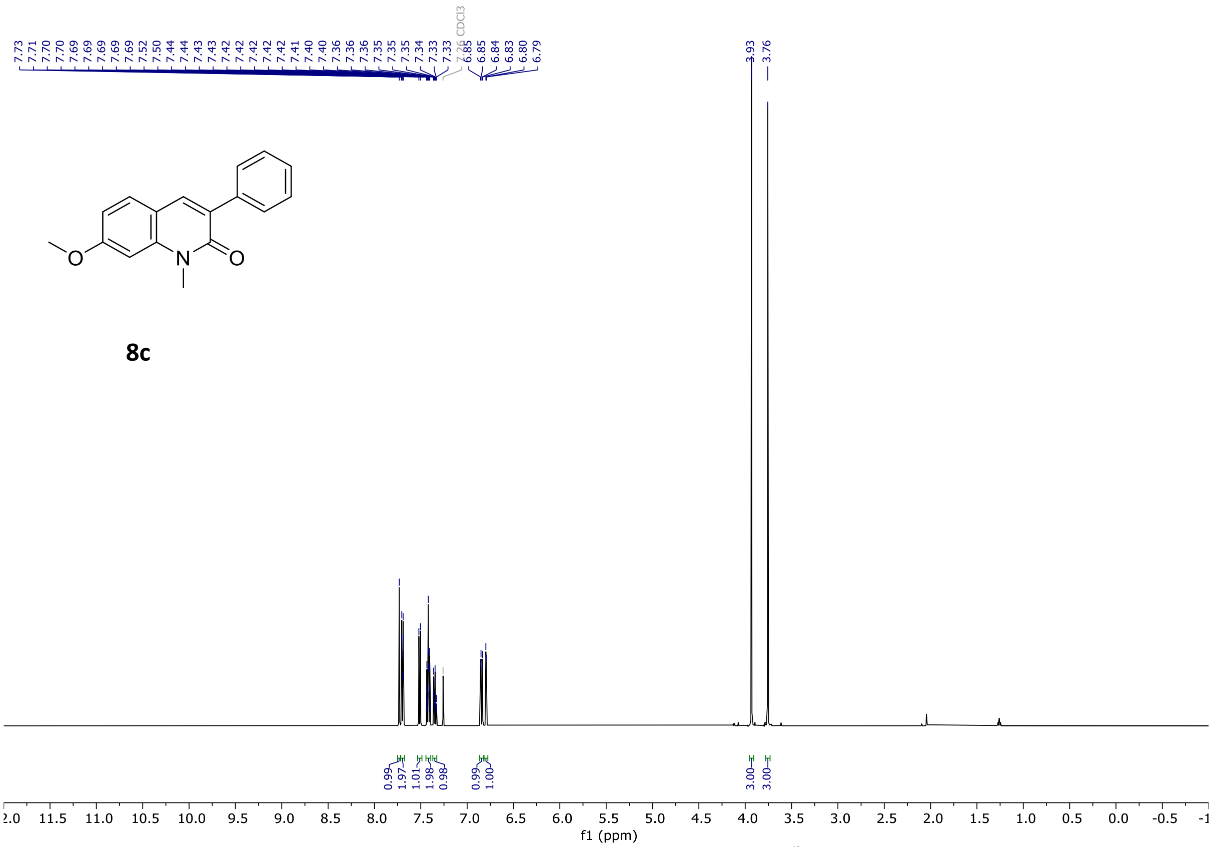
7.73 7.71 7.70 7.69 7.69 7.69 7.69 7.69 7.52 7.50 7.44 7.44 7.43 7.43 7.42 7.42 7.42 7.42 7.41 7.40 7.40 7.36 7.36 7.36 7.35 7.35 7.34 7.33 7.33
7.26 CDCl3
6.85 6.85 6.84 6.83 6.80 6.79
3.93
3.76
8c
0.99 1.97 1.01 1.98 0.98 0.99 1.00 3.00 3.00
f1 (ppm)

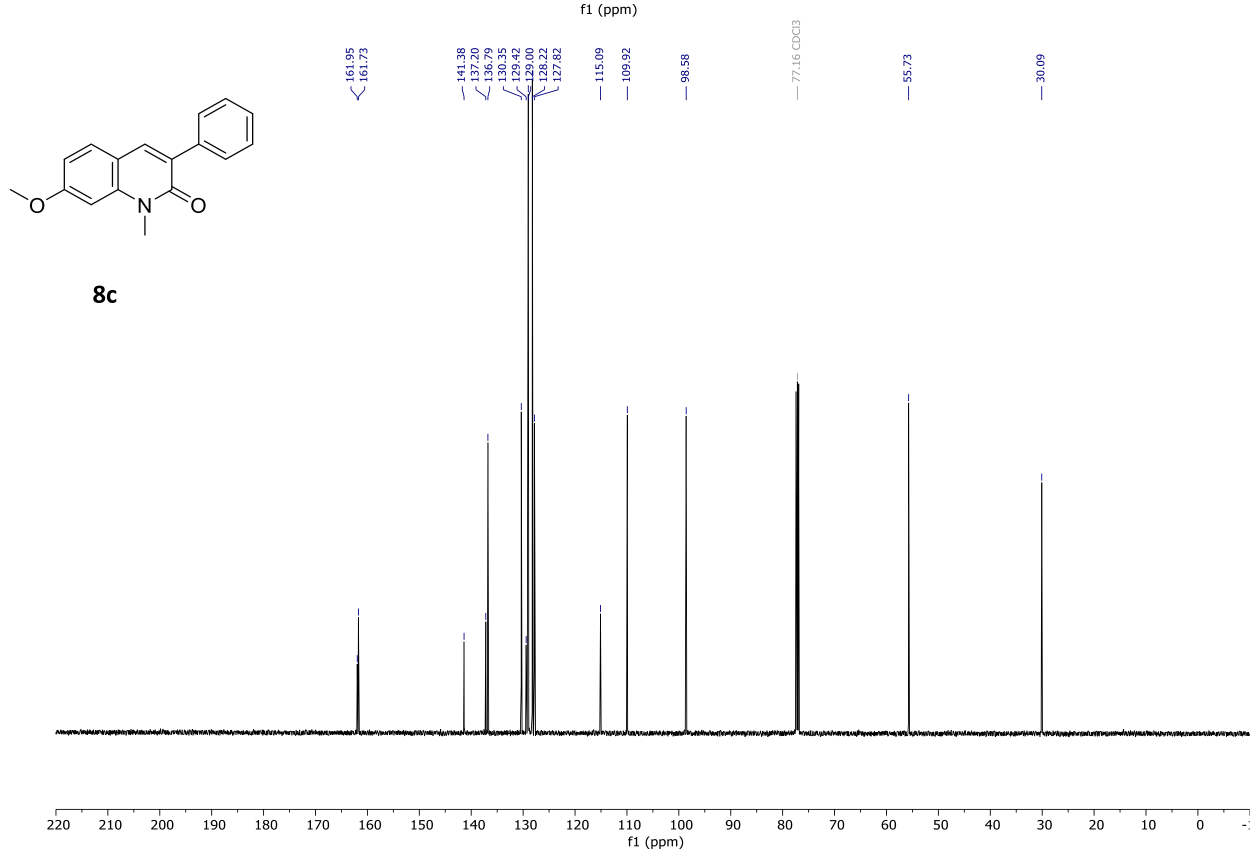
161.95 161.73
141.38 137.20 136.79 130.35 129.42 129.00 128.22 127.82
115.09
109.92
98.58
77.16 CDCl3
55.73
30.09
8c
f1 (ppm)

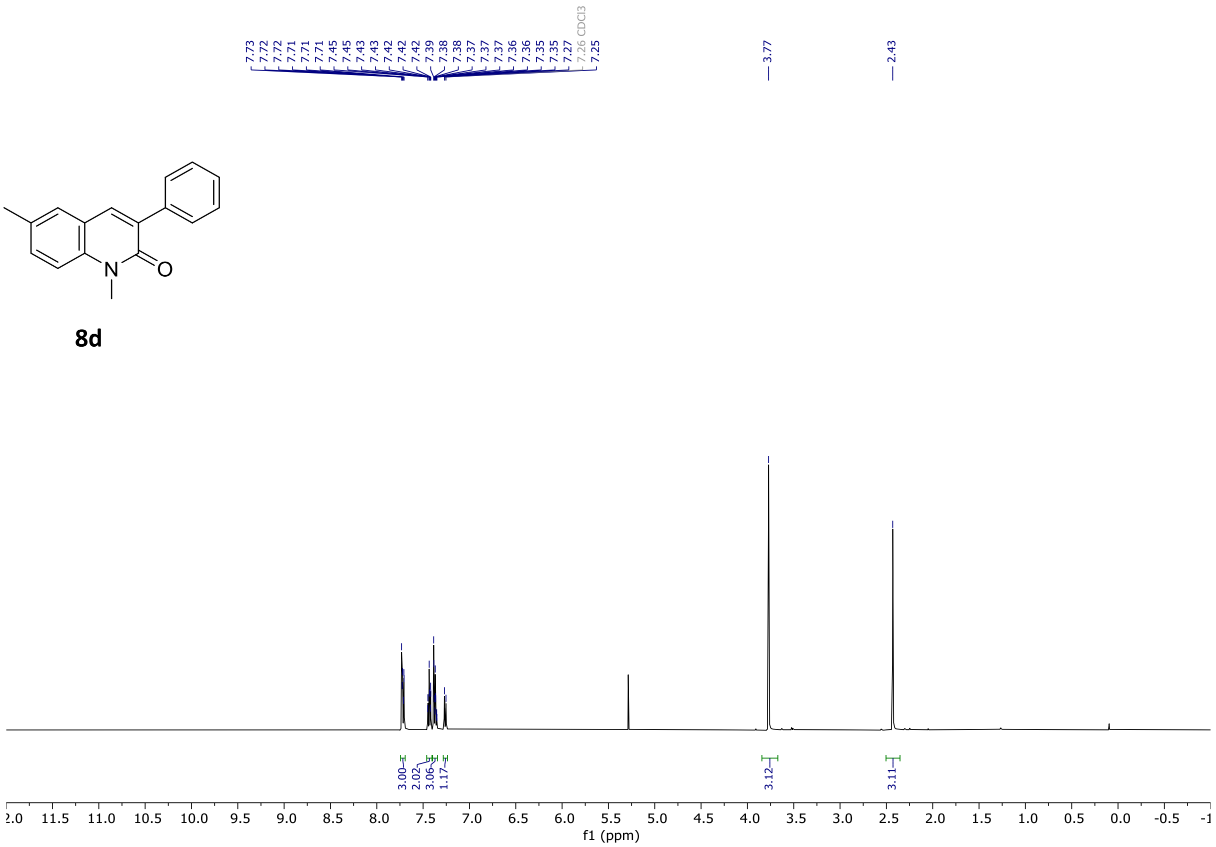
7.73 7.72 7.72 7.71 7.71 7.71 7.45 7.45 7.43 7.43 7.42 7.42 7.42 7.39 7.38 7.38 7.37 7.37 7.37 7.36 7.36 7.35 7.35 7.27 7.26 CDCl3 7.25
3.77
2.43
8d
3.00
2.02
3.06
1.17
3.12
3.11
f1 (ppm)

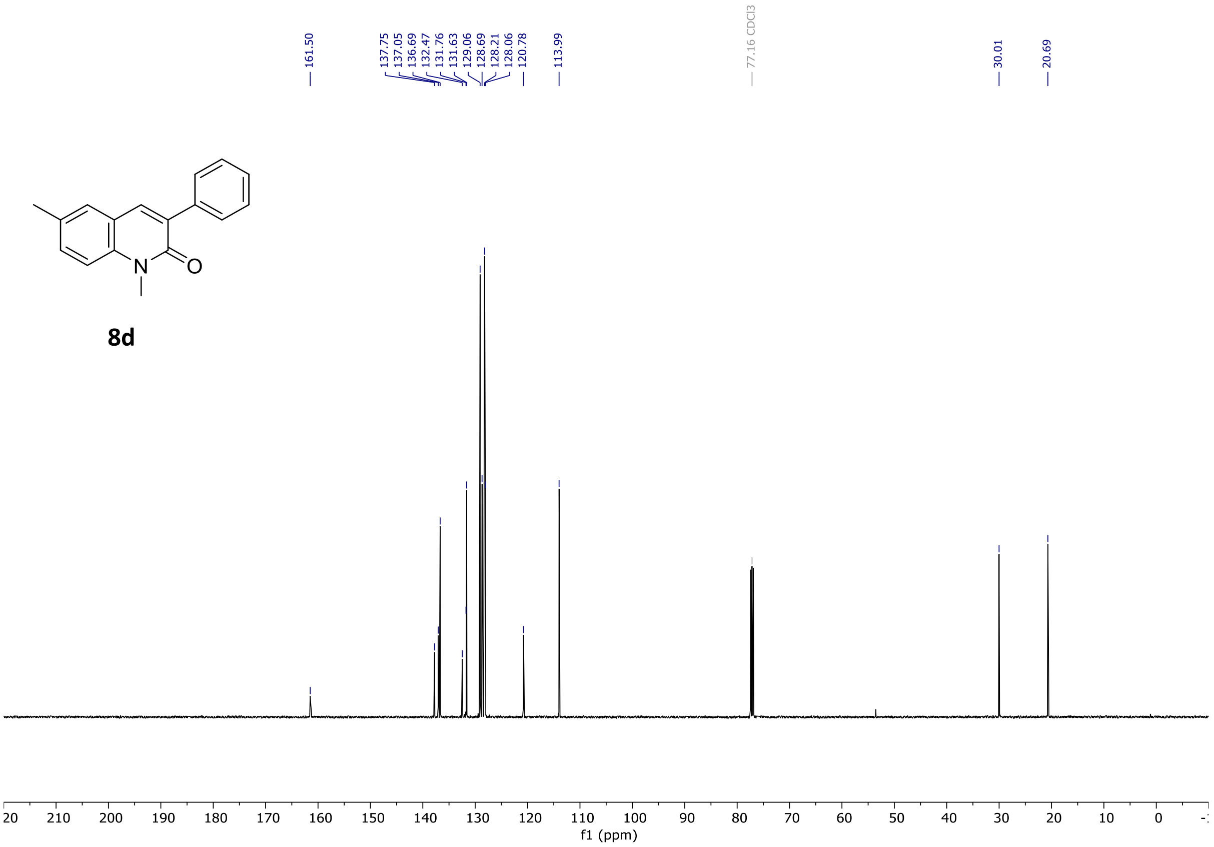
161.50
137.75 137.05 136.69 132.47 131.76 131.63 129.06 128.69 128.21 128.06 120.78
113.99
77.16 CDCl3
30.01
20.69
8d
f1 (ppm)

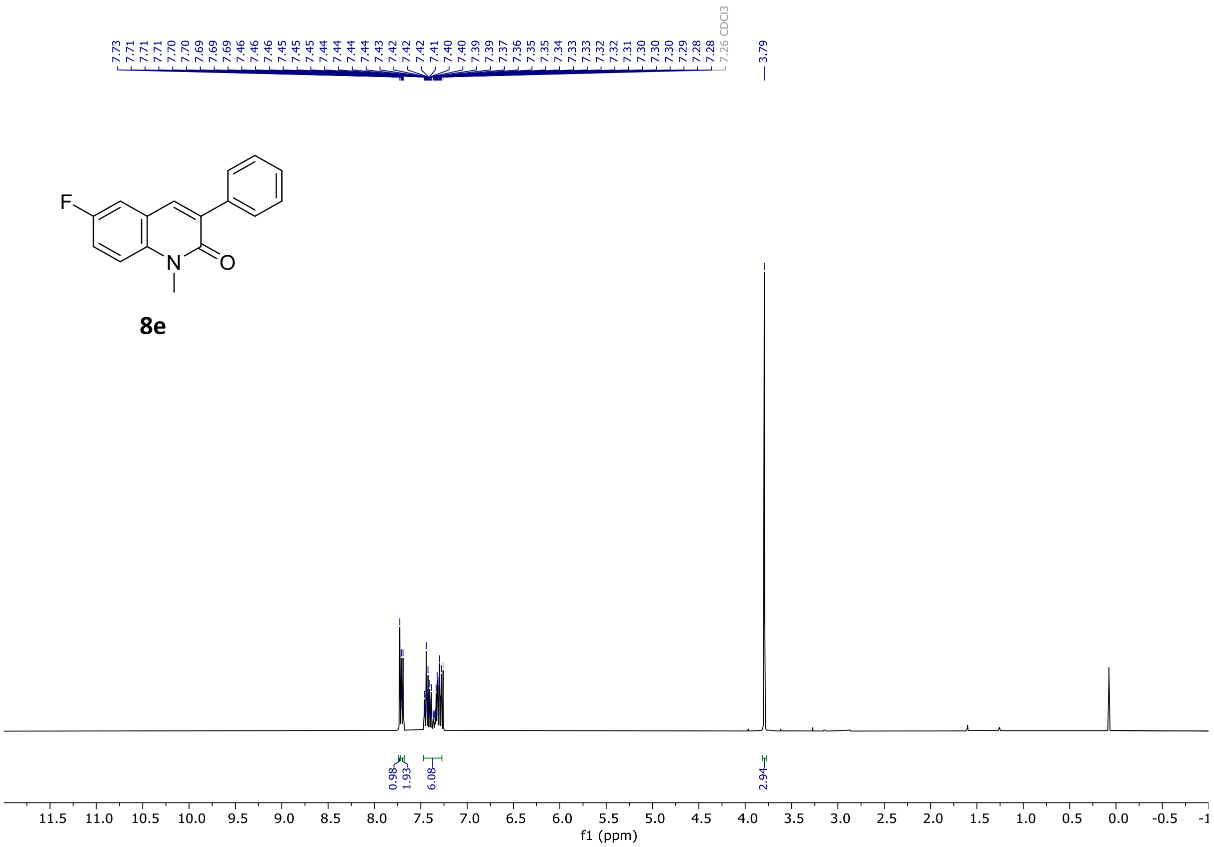

8e
7.73 7.71 7.71 7.71 7.70 7.70 7.69 7.69 7.69 7.46 7.46 7.45 7.45 7.45 7.44 7.44 7.44 7.43 7.42 7.42 7.42 7.41 7.40 7.40 7.39 7.39 7.37 7.36 7.35 7.35 7.34 7.33 7.33 7.32 7.32 7.31 7.30 7.30 7.29 7.28 7.28
7.26 CDCl3
3.79
0.98
1.93
6.08
2.94
11.5 11.0 10.5 10.0 9.5 9.0 8.5 8.0 7.5 7.0 6.5 6.0 5.5 5.0 4.5 4.0 3.5 3.0 2.5 2.0 1.5 1.0 0.5 0.0 -0.5 -1
f1 (ppm)

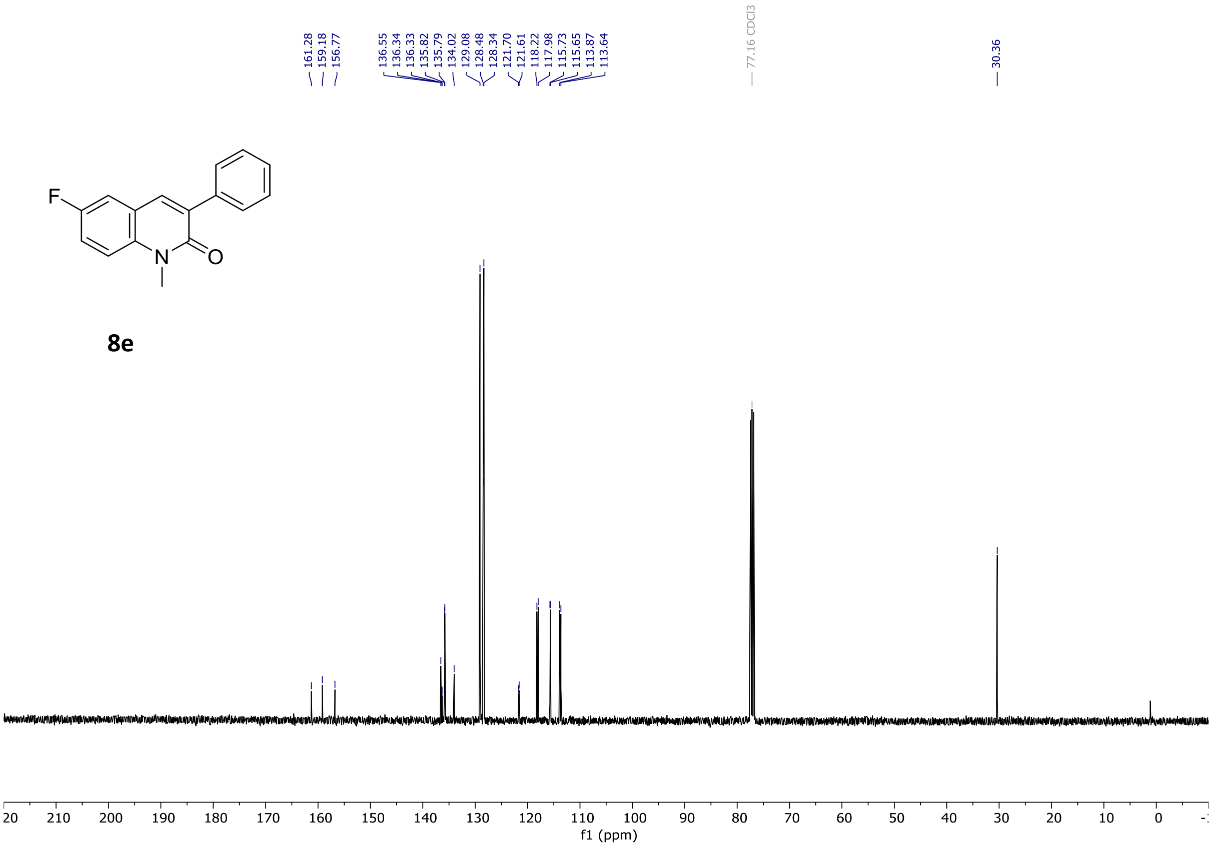

8e
161.28 159.18 156.77
136.55 136.34 136.33 135.82 135.79 134.02 129.08 128.48 128.34 121.70 121.61 118.22 117.98 115.73 115.65 113.87 113.64
77.16 CDCl3
30.36
20 210 200 190 180 170 160 150 140 130 120 110 100 90 80 70 60 50 40 30 20 10 0
f1 (ppm)

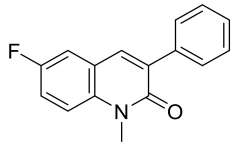

8e

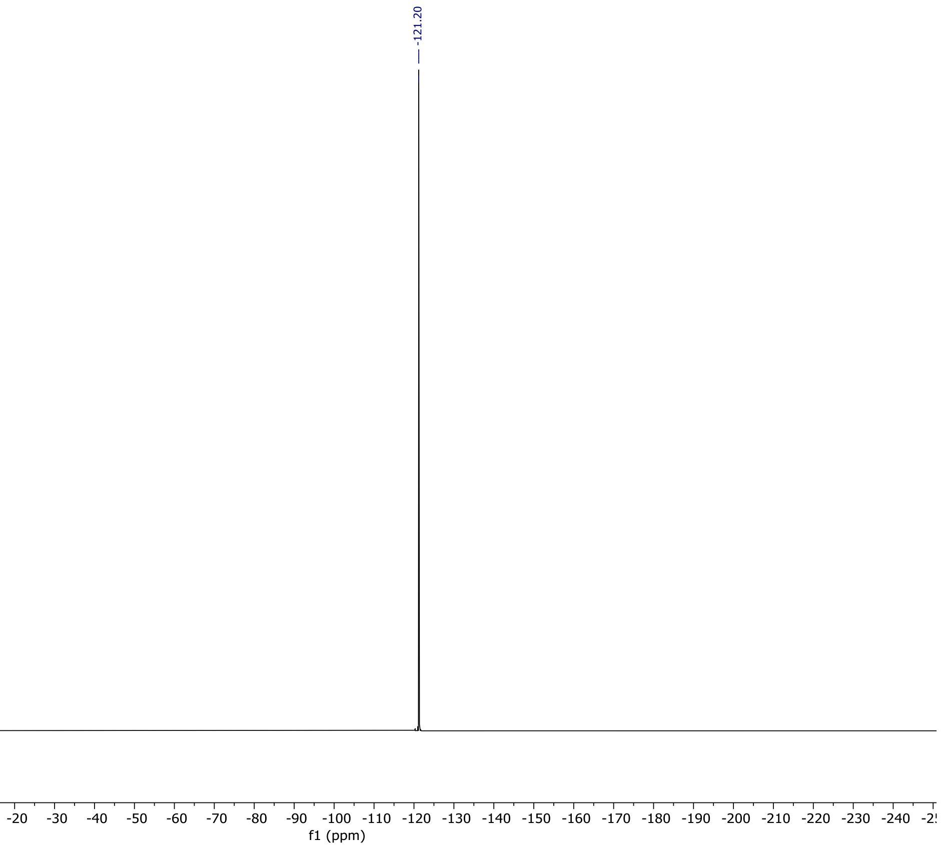

-121.20
50 40 30 20 10 0 -10 -20 -30 -40 -50 -60 -70 -80 -90 -100 -110 -120 -130 -140 -150 -160 -170 -180 -190 -200 -210 -220 -230 -240
f1 (ppm)

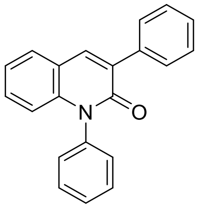

8f

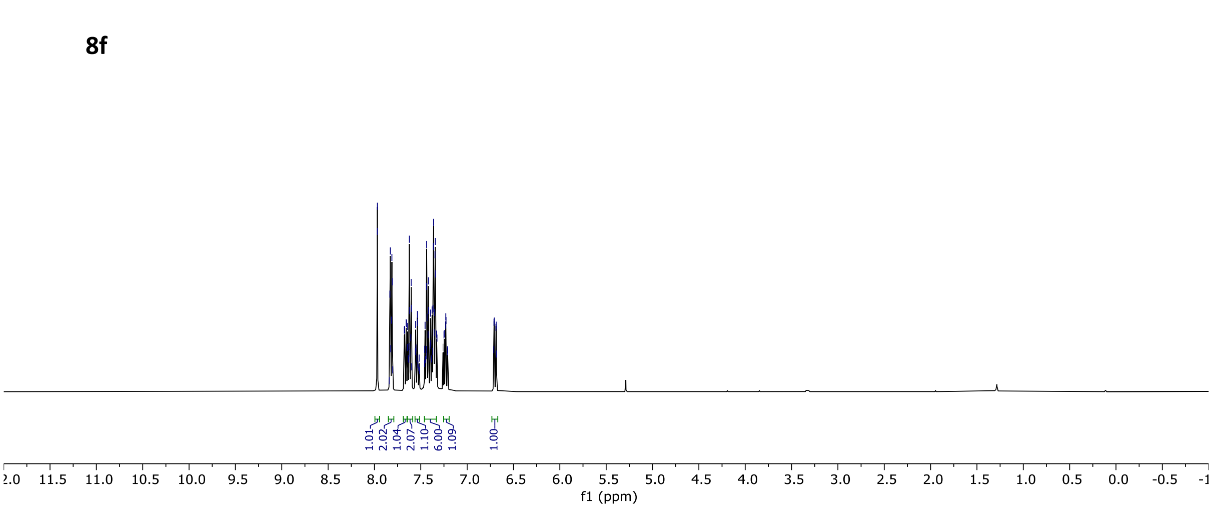

7.97 7.97 7.84 7.83 7.83 7.82 7.82 7.81 7.81 7.80 7.68 7.67 7.66 7.65 7.64 7.64 7.64 7.63 7.63 7.62 7.62 7.62 7.61 7.60 7.60 7.56 7.56 7.55 7.54 7.54 7.54 7.54 7.53 7.52 7.52 7.51 7.46 7.46 7.45 7.45 7.44 7.44 7.44 7.44 7.43 7.42 7.42 7.42 7.40 7.40 7.39 7.38 7.38 7.37 7.37 7.37 7.36 7.36 7.35 7.35 7.34 7.34 7.33 7.33 7.26 CDCl3 7.25 7.25 7.23 7.23 7.23 7.21 7.21 6.71 6.71 6.71 6.69 6.69 6.68
1.01 2.02 1.04 2.07 1.10 6.00 1.09 1.00
11.5 11.0 10.5 10.0 9.5 9.0 8.5 8.0 7.5 7.0 6.5 6.0 5.5 5.0 4.5 4.0 3.5 3.0 2.5 2.0 1.5 1.0 0.5 0.0 -0.5
f1 (ppm)

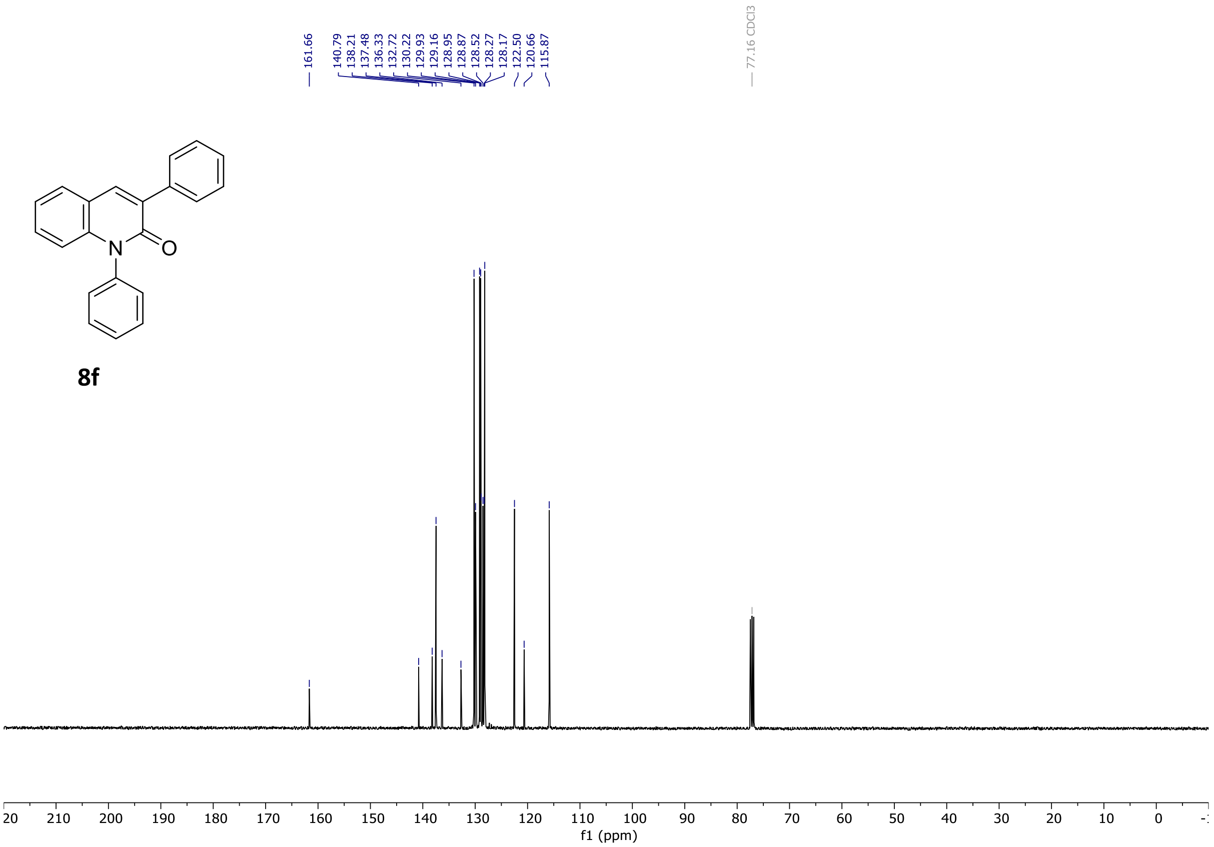

8f

161.66
140.79
138.21
137.48
136.33
132.72
130.22
129.93
129.16
128.95
128.87
128.52
128.27
128.17
122.50
120.66
115.87
77.16 CDCl3
20 210 200 190 180 170 160 150 140 130 120 110 100 90 80 70 60 50 40 30 20 10 0
f1 (ppm)

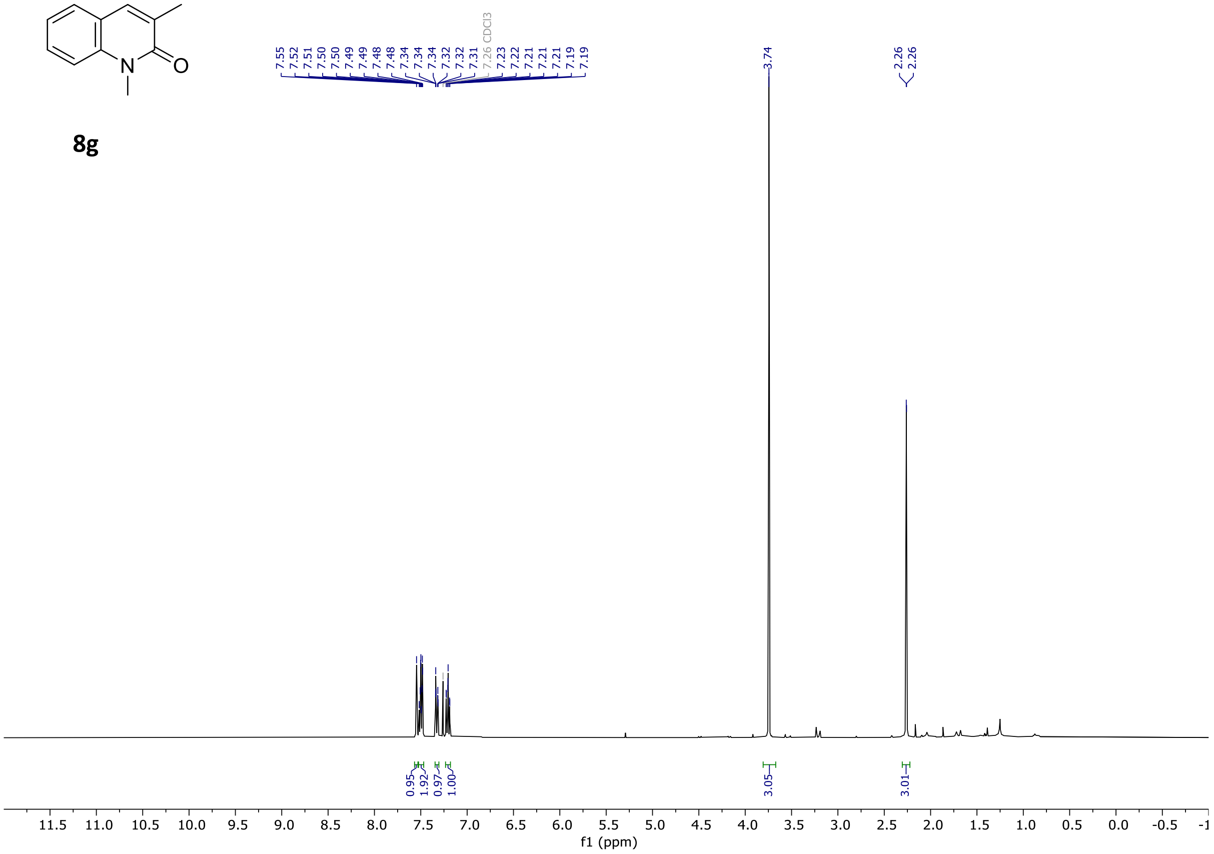

8g

7.55
7.52
7.51
7.50
7.50
7.49
7.49
7.48
7.34
7.34
7.32
7.32
7.31
7.26 CDCl3
7.23
7.22
7.21
7.21
7.21
7.19
7.19
3.74
2.26
2.26
0.95
1.92
0.97
1.00
3.05
3.01
11.5 11.0 10.5 10.0 9.5 9.0 8.5 8.0 7.5 7.0 6.5 6.0 5.5 5.0 4.5 4.0 3.5 3.0 2.5 2.0 1.5 1.0 0.5 0.0 -0.5
f1 (ppm)

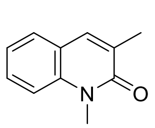

8g

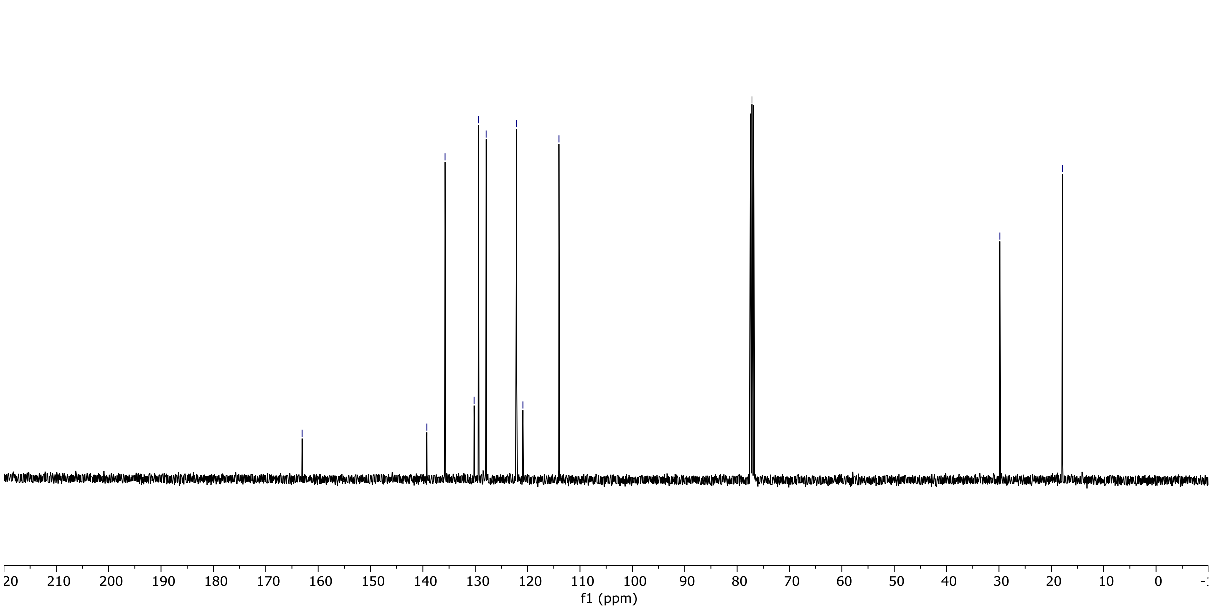
163.06
139.24
135.77
130.22
129.39
127.92
122.09
120.89
114.01
77.16 CDCl3
29.82
17.89
f1 (ppm)

Linopiridine

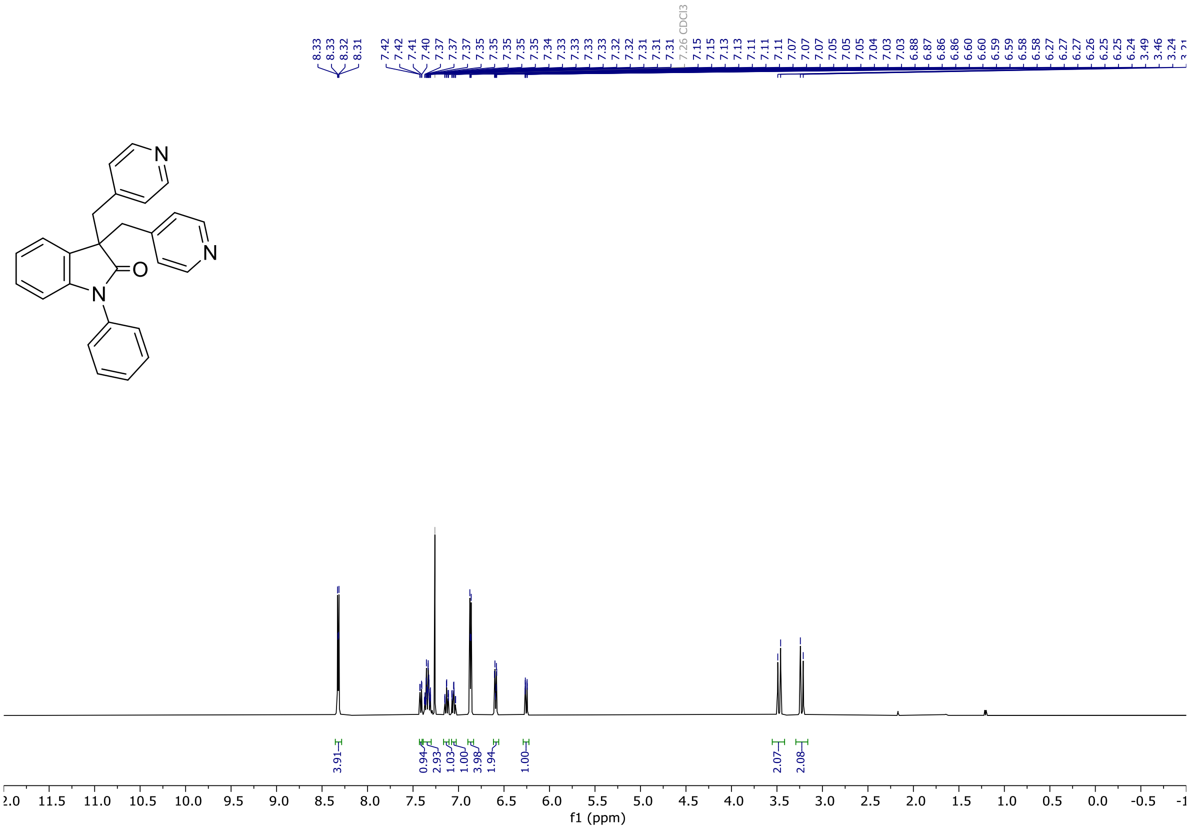

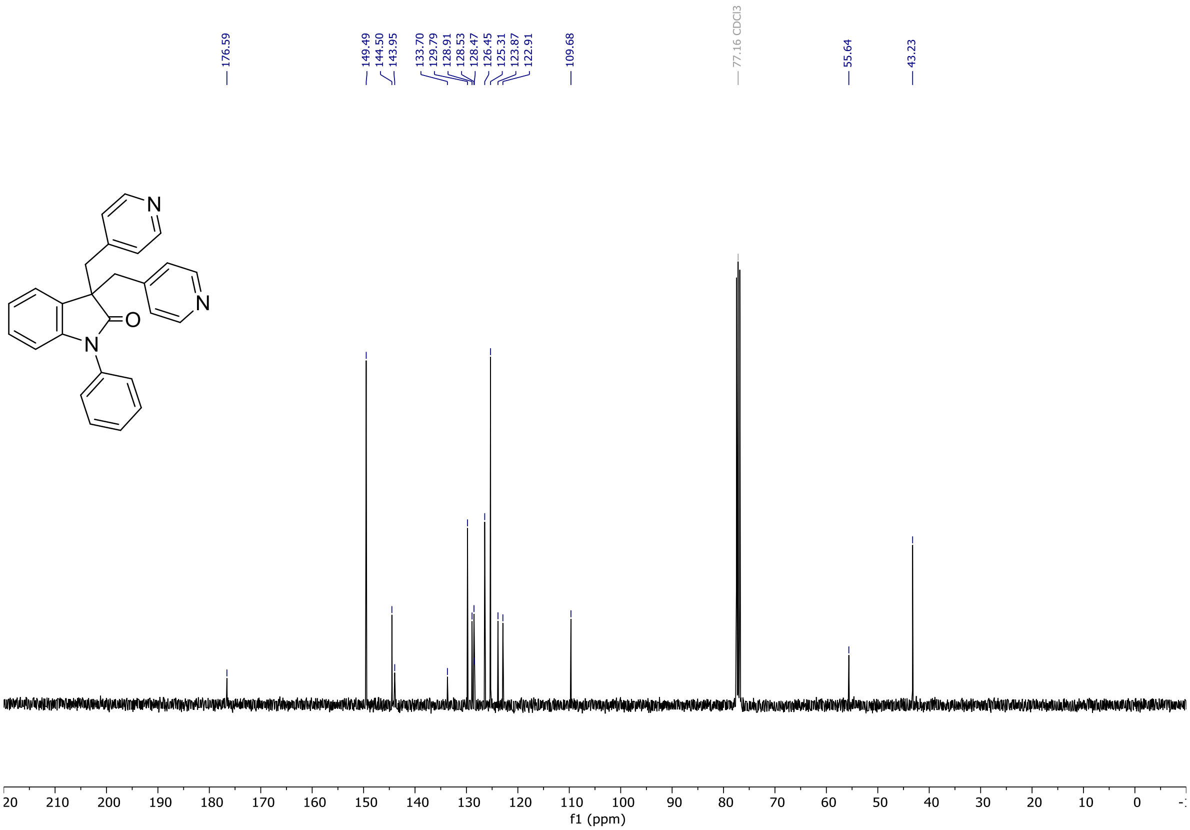

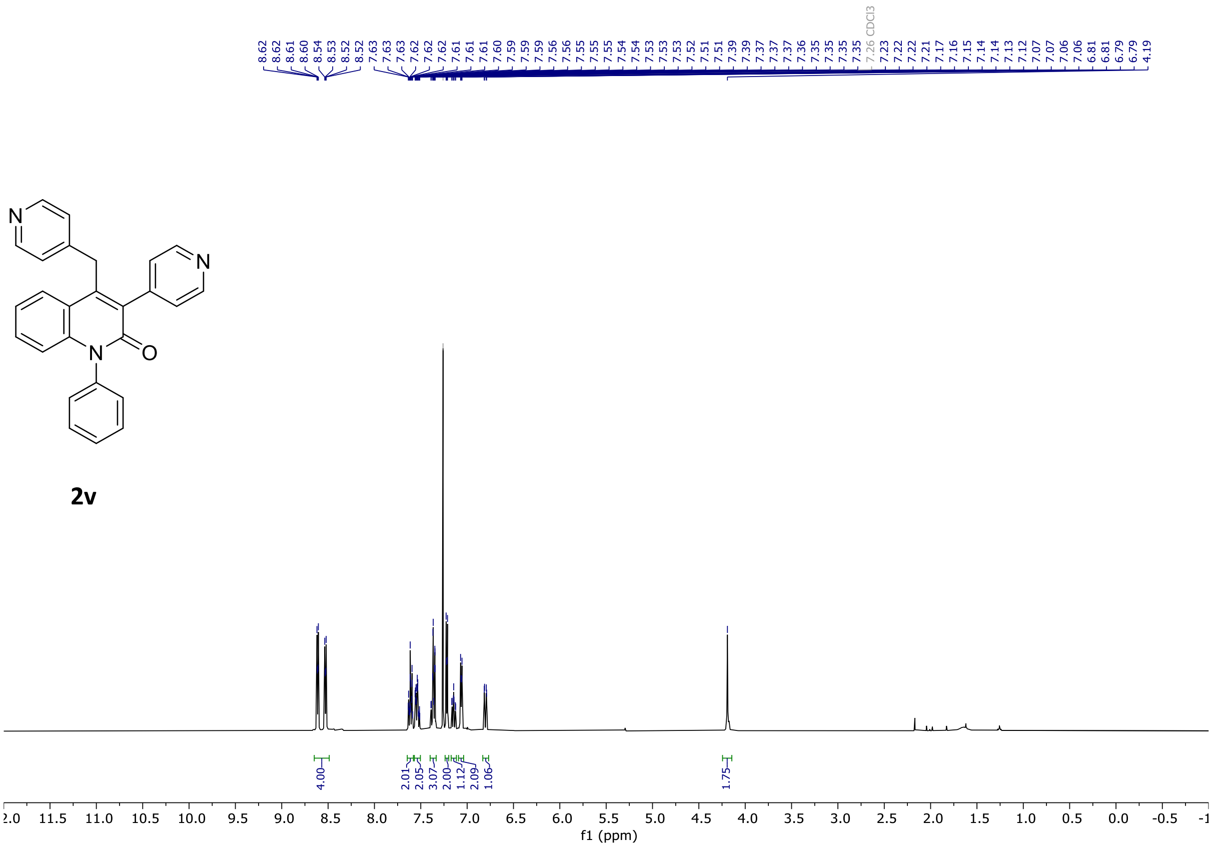

7.26 CDCl3
4.19
2v
f1 (ppm)

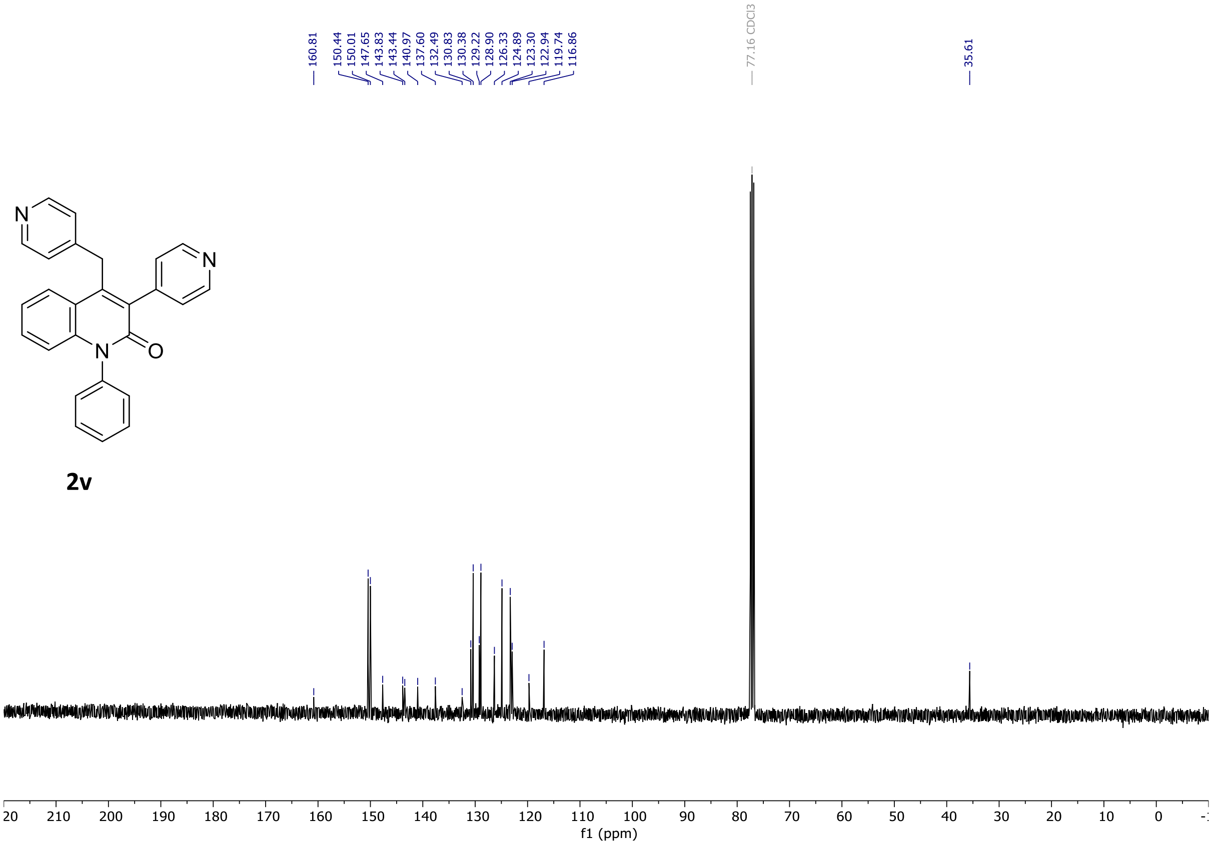

160.81
150.44
150.01
147.65
143.83
143.44
140.97
137.60
132.49
130.83
130.38
129.22
128.90
126.33
124.89
123.30
122.94
119.74
116.86
77.16 CDCl3
35.61
2v
f1 (ppm)

Doliracetam

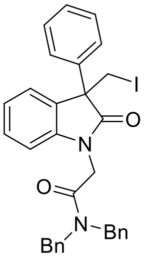

1wb

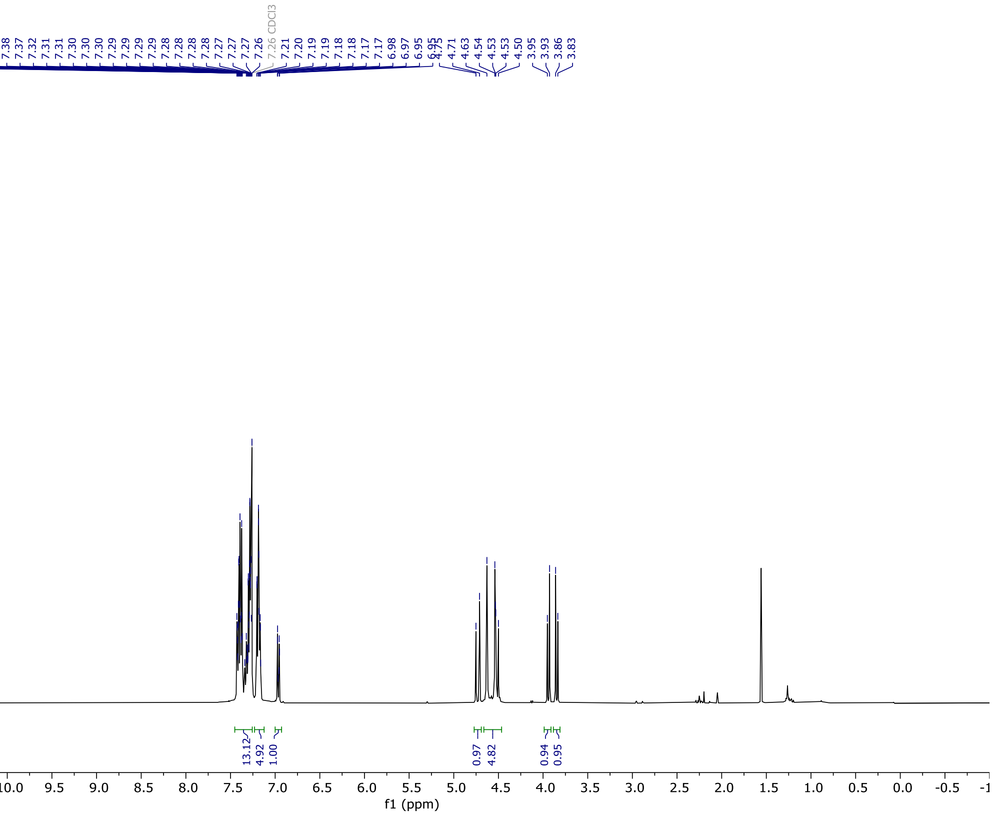

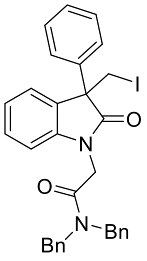

1wb

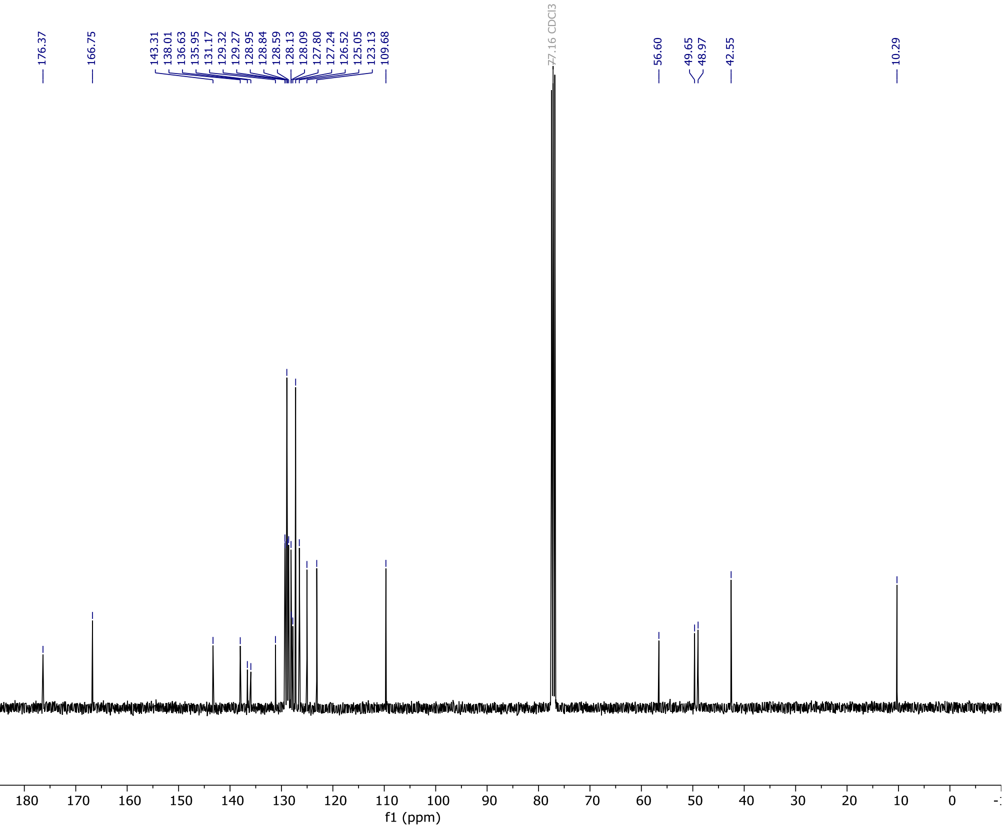

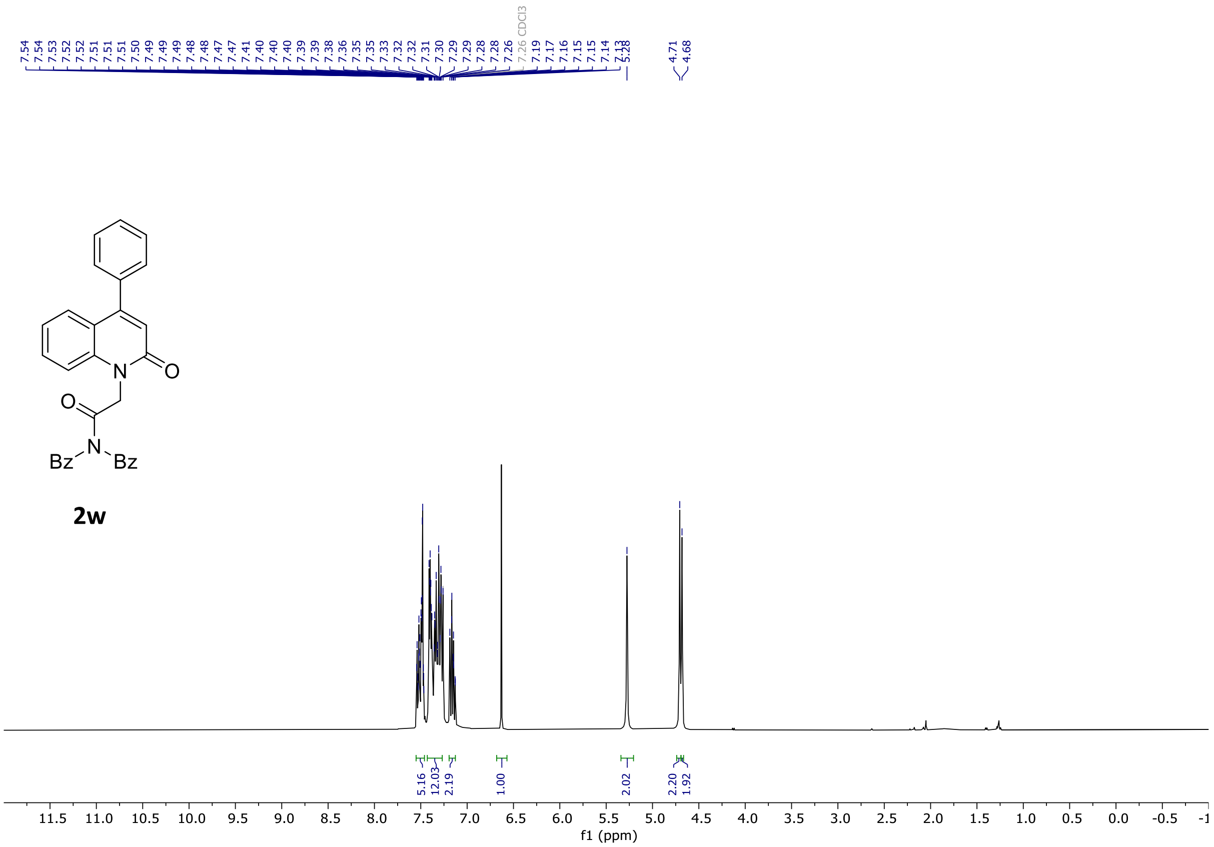
7.54
7.54
7.53
7.52
7.52
7.51
7.51
7.51
7.50
7.49
7.49
7.48
7.48
7.47
7.47
7.41
7.40
7.40
7.39
7.39
7.39
7.38
7.36
7.35
7.35
7.33
7.32
7.32
7.31
7.30
7.29
7.29
7.28
7.28
7.26
7.26 CDCl3
7.19
7.17
7.16
7.15
7.15
7.14
7.13
5.28
4.71
4.68
Bz
N
Bz
O
N
O
2w
5.16
12.03
2.19
1.00
2.02
2.20
1.92
11.5 11.0 10.5 10.0 9.5 9.0 8.5 8.0 7.5 7.0 6.5 6.0 5.5 5.0 4.5 4.0 3.5 3.0 2.5 2.0 1.5 1.0 0.5 0.0 -0.5
f1 (ppm)

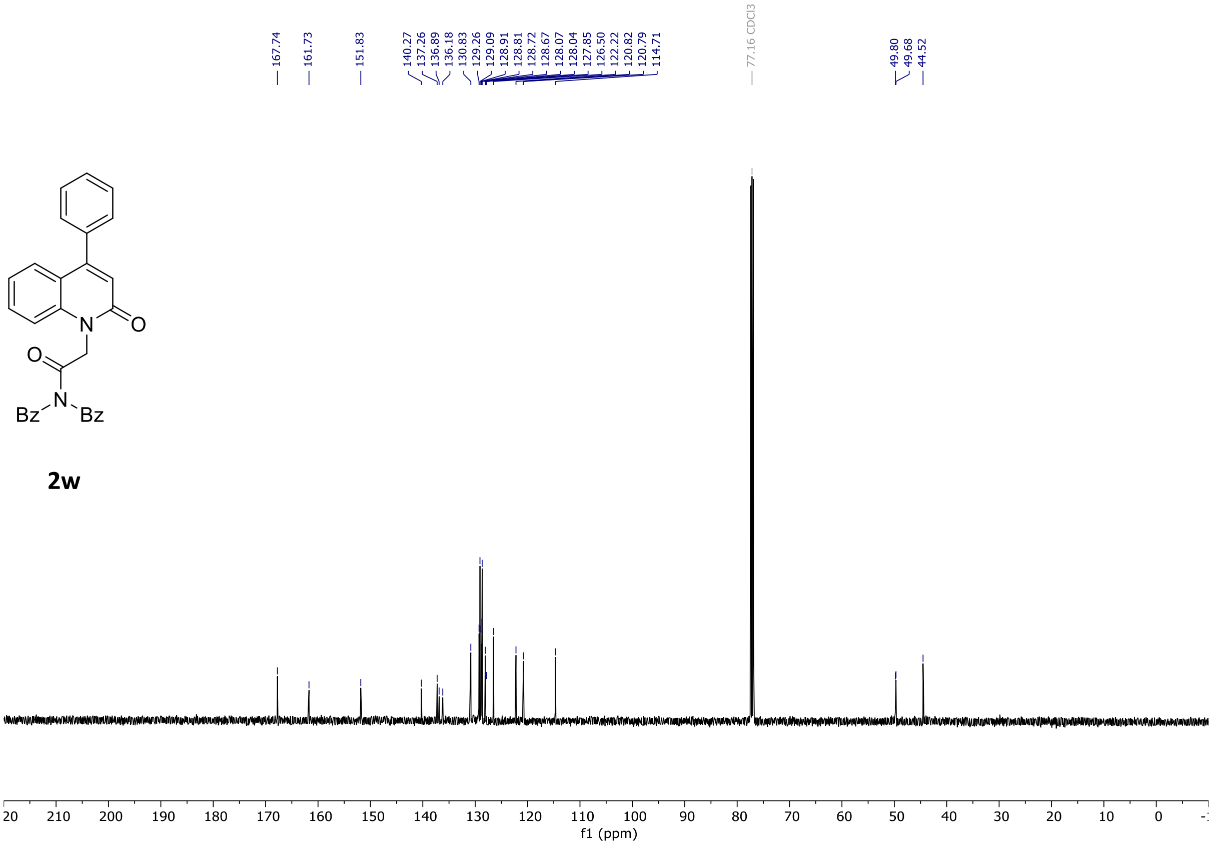
167.74
161.73
151.83
140.27
137.26
136.89
136.18
130.83
129.26
129.09
128.91
128.81
128.72
128.67
128.07
128.04
127.85
126.50
122.22
120.82
120.79
114.71
77.16 CDCl3
49.80
49.68
44.52
Bz
N
Bz
O
N
O
2w
210 200 190 180 170 160 150 140 130 120 110 100 90 80 70 60 50 40 30 20 10 0
f1 (ppm)

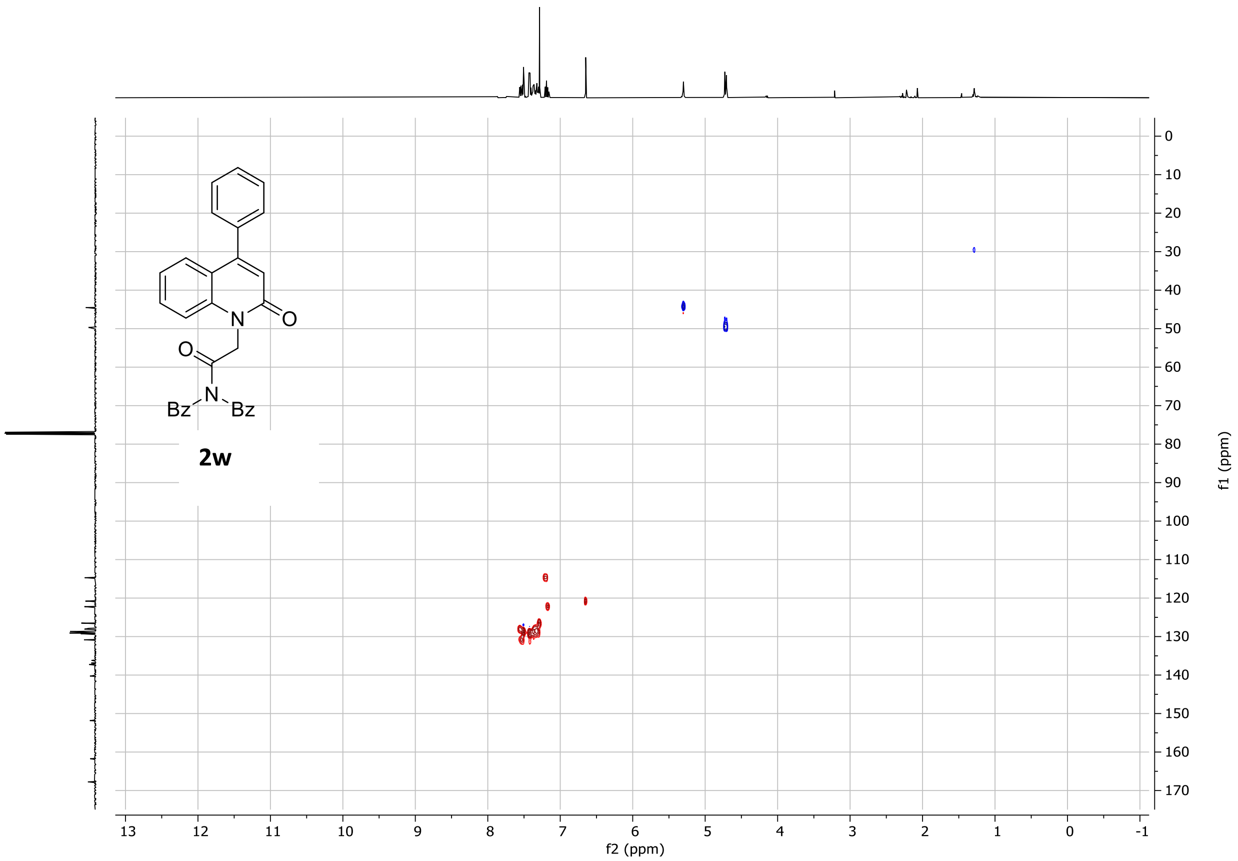

Bz
N
Bz
O
N
O
2w
f2 (ppm)
f1 (ppm)

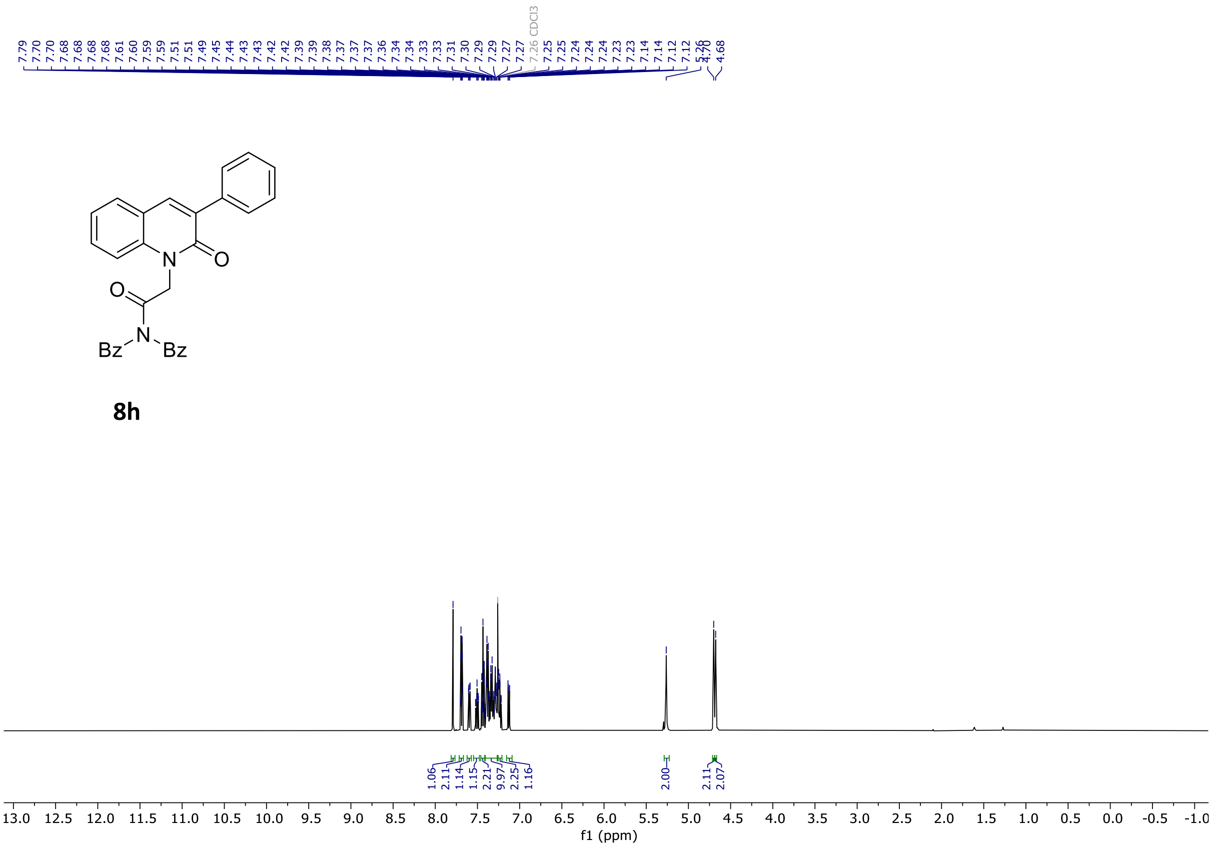
7.79 7.70 7.70 7.68 7.68 7.68 7.61 7.60 7.59 7.59 7.51 7.51 7.49 7.45 7.44 7.43 7.43 7.42 7.42 7.39 7.39 7.38 7.37 7.37 7.37 7.36 7.34 7.34 7.33 7.33 7.31 7.30 7.29 7.29 7.27 7.27
7.26 CDCl3
7.25 7.25 7.24 7.24 7.24 7.23 7.23 7.14 7.14 7.12 7.12 5.26 4.70 4.68
N
O
O
N
Bz
Bz
8h
1.06 2.11 1.14 1.15 2.21 9.97 2.25 1.16
2.00
2.11 2.07
13.0 12.5 12.0 11.5 11.0 10.5 10.0 9.5 9.0 8.5 8.0 7.5 7.0 6.5 6.0 5.5 5.0 4.5 4.0 3.5 3.0 2.5 2.0 1.5 1.0 0.5 0.0 -0.5 -1.0
f1 (ppm)

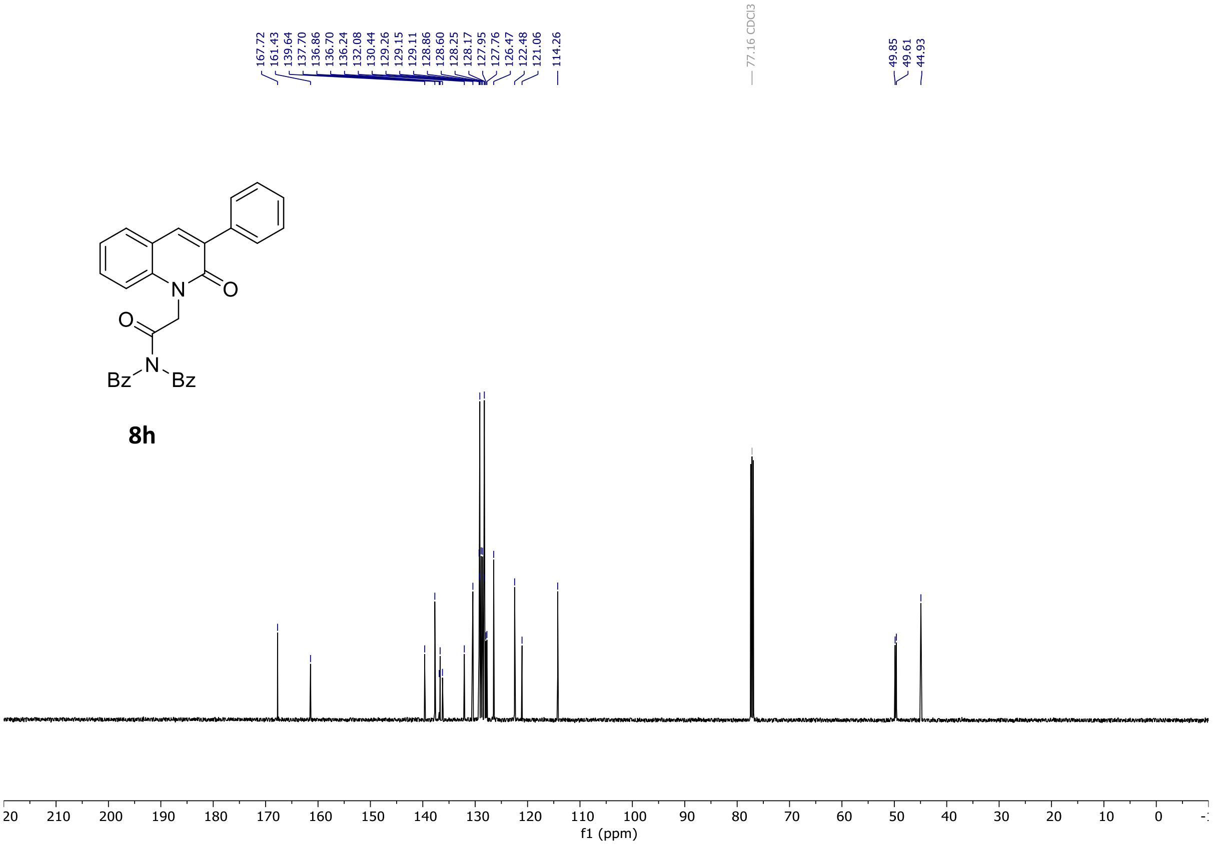
167.72 161.43 139.64 137.70 136.86 136.70 136.24 132.08 130.44 129.26 129.15 129.11 128.86 128.60 128.25 128.17 127.95 127.76 126.47 122.48 121.06 114.26
77.16 CDCl3
49.85 49.61 44.93
N
O
O
N
Bz
Bz
8h
20 210 200 190 180 170 160 150 140 130 120 110 100 90 80 70 60 50 40 30 20 10 0
f1 (ppm)

YWI92

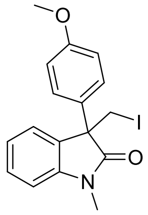

1xb

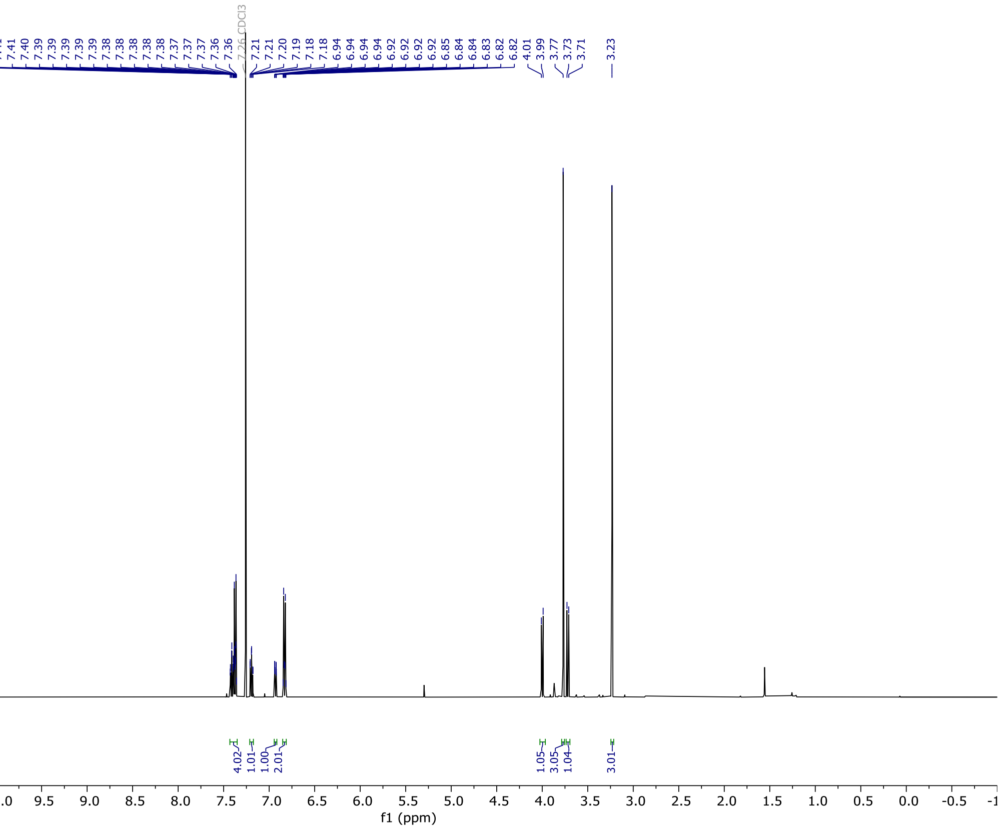

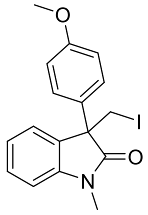

1xb

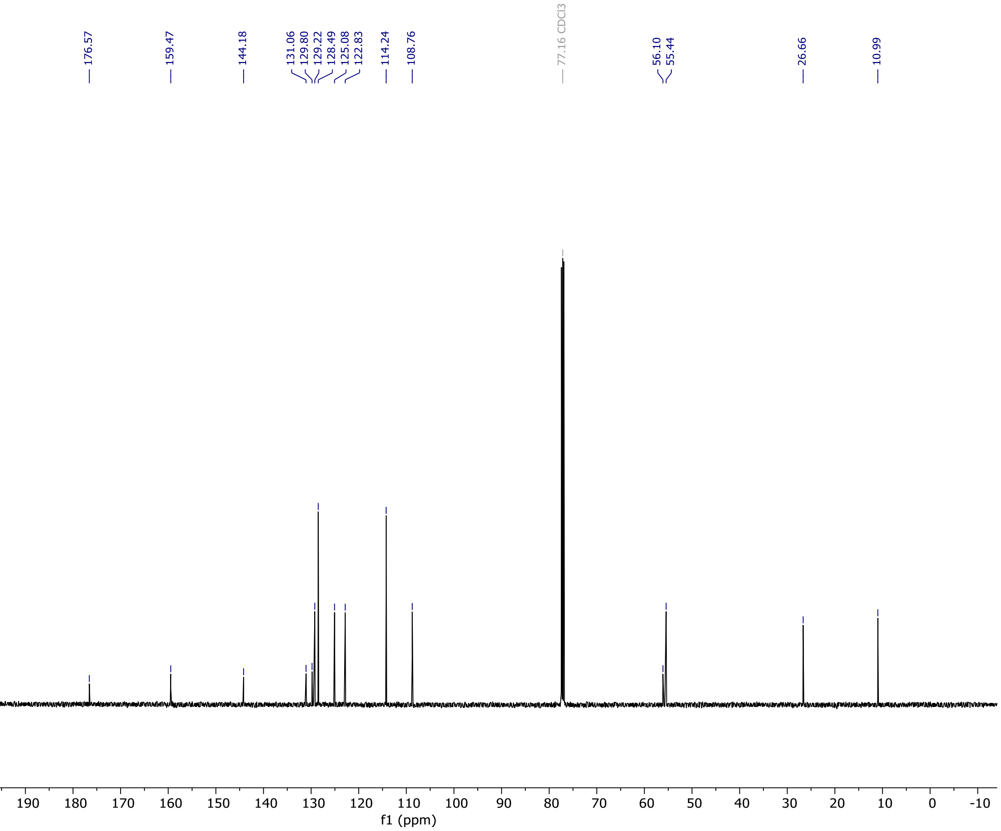

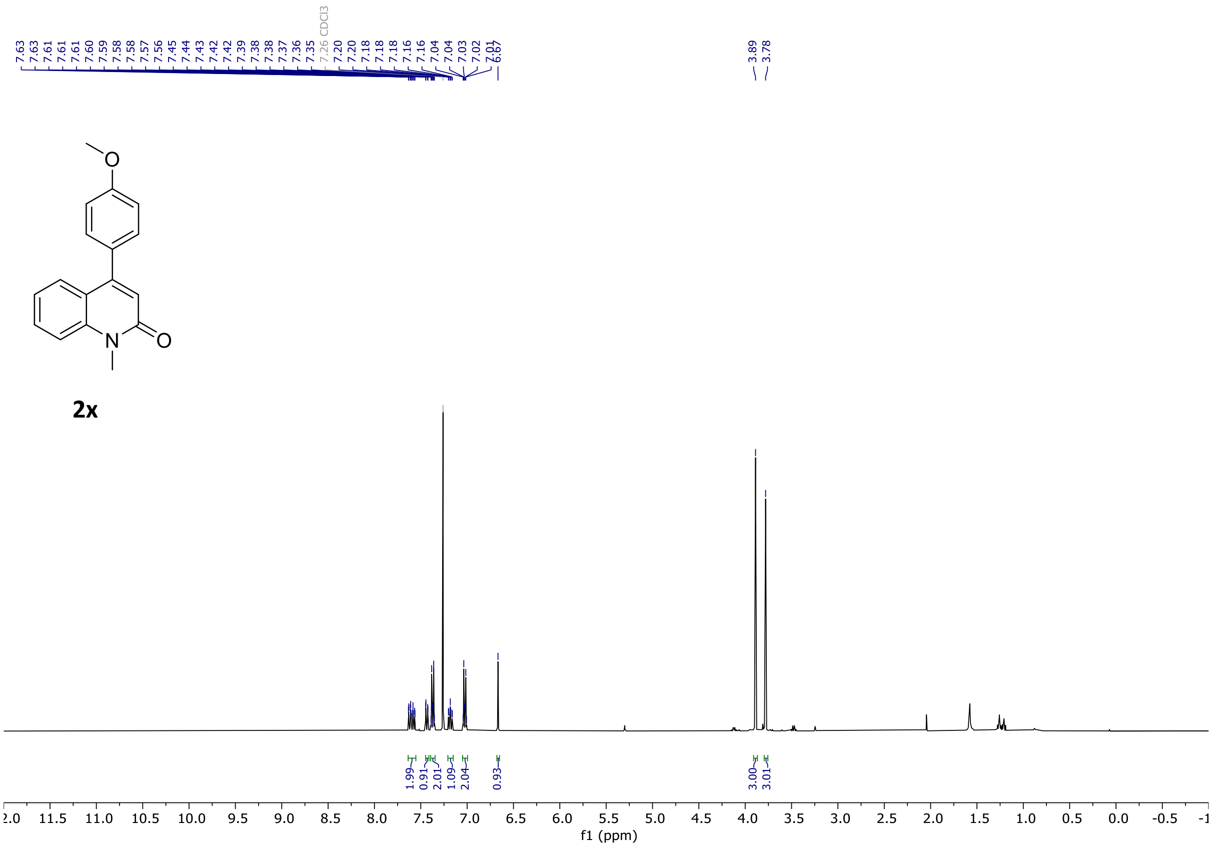

7.63
7.63
7.61
7.61
7.60
7.59
7.58
7.58
7.57
7.56
7.45
7.44
7.43
7.42
7.42
7.39
7.38
7.38
7.37
7.36
7.35
7.26 CDCl3
7.20
7.20
7.18
7.18
7.18
7.16
7.16
7.04
7.04
7.03
7.02
7.01
6.67
3.89
3.78
2x
1.99
0.91
2.01
1.09
2.04
0.93
3.00
3.01
f1 (ppm)

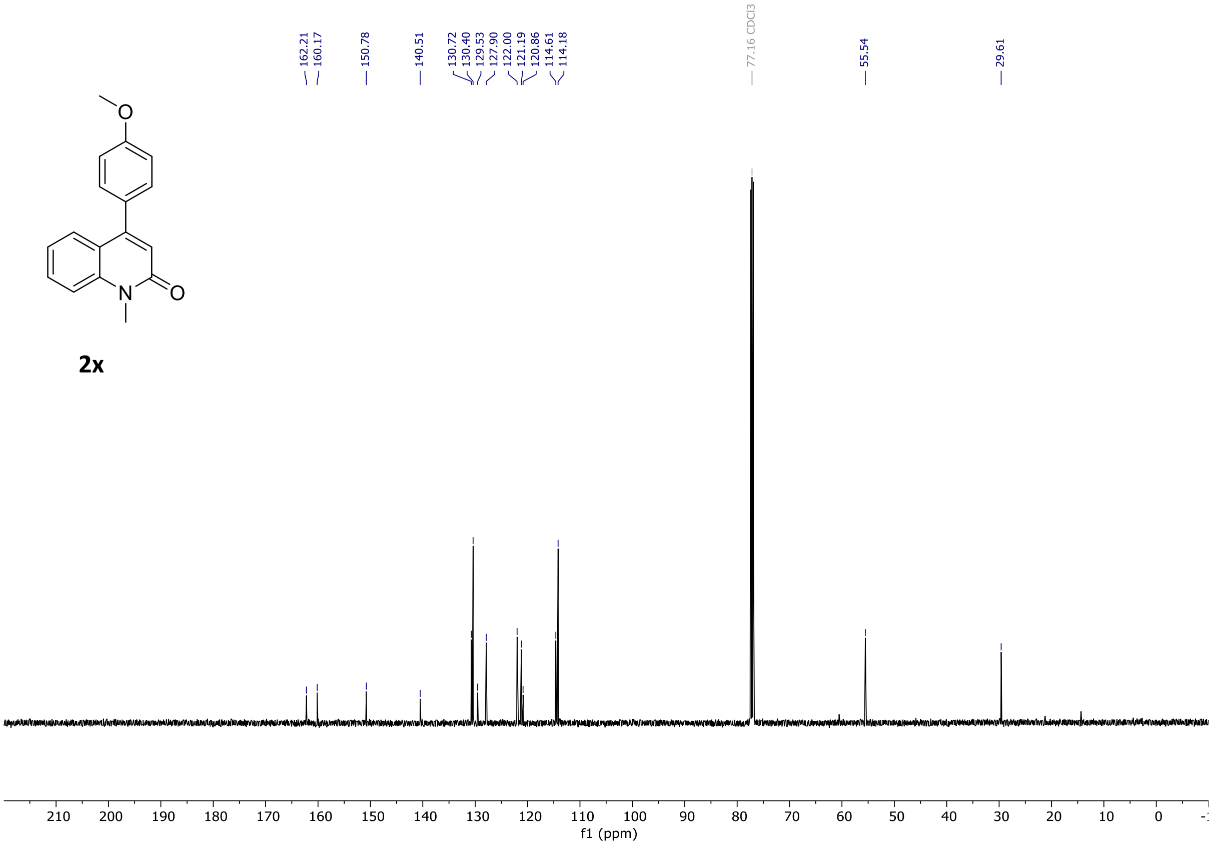

162.21
160.17
150.78
140.51
130.72
130.40
129.53
127.90
122.00
121.19
120.86
114.61
114.18
77.16 CDCl3
55.54
29.61
2x
f1 (ppm)

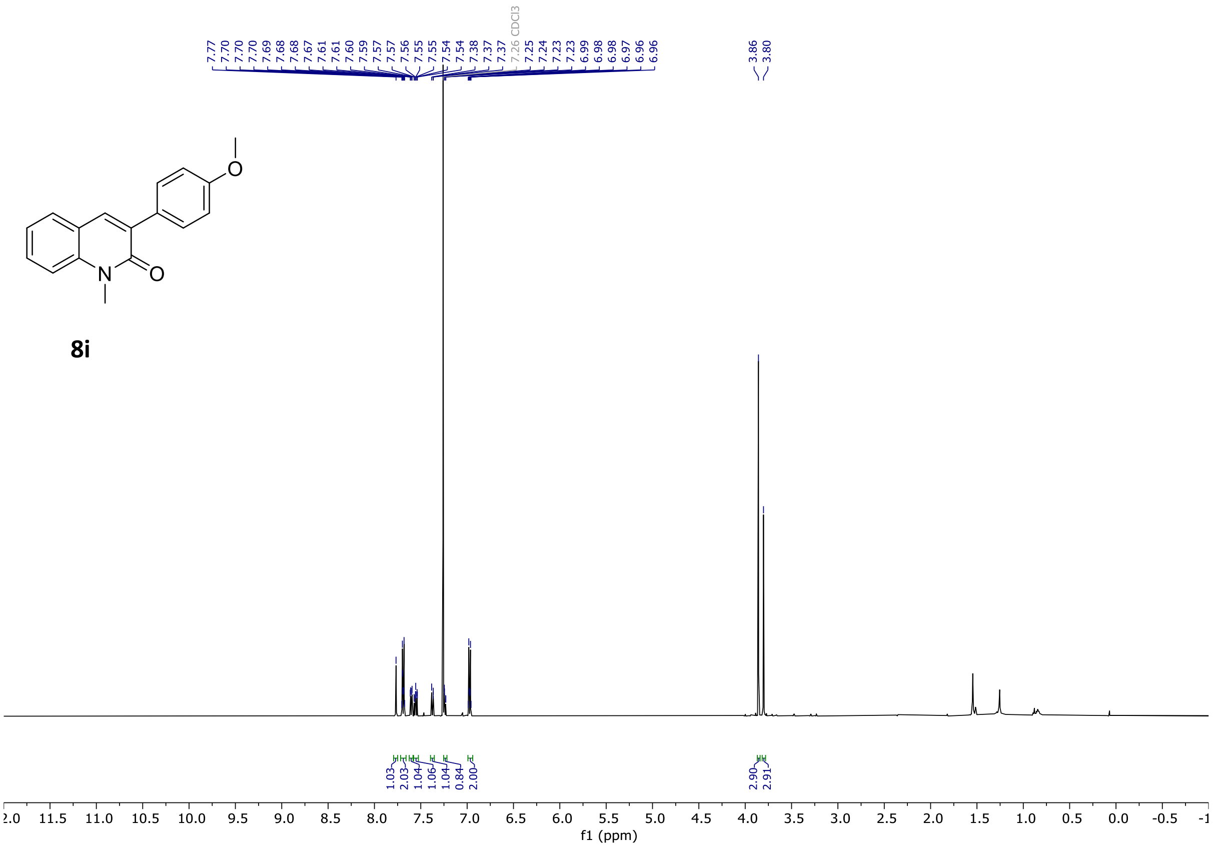
7.77 7.70 7.70 7.69 7.68 7.68 7.67 7.61 7.61 7.60 7.59 7.57 7.57 7.56 7.55 7.55 7.54 7.54 7.38 7.37 7.37 7.26 CDCl3 7.25 7.24 7.23 7.23 6.99 6.98 6.98 6.97 6.96 6.96
3.86 3.80
O
N
O
8i
1.03 2.03 1.04 1.06 1.04 0.84 2.00
2.90 2.91
f1 (ppm)

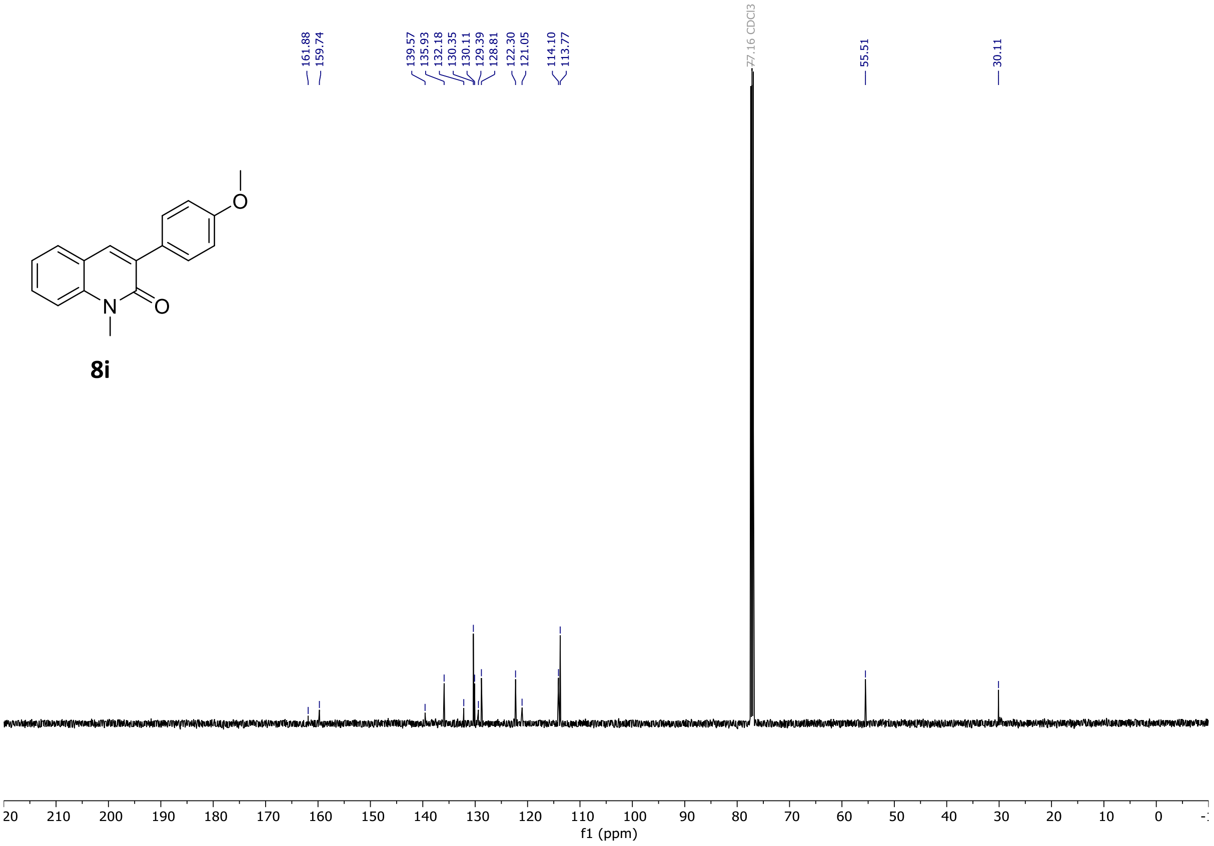
161.88 159.74
139.57 135.93 132.18 130.35 130.11 129.39 128.81 122.30 121.05 114.10 113.77
77.16 CDCl3
55.51
30.11
O
N
O
8i
f1 (ppm)

Tipifarnib

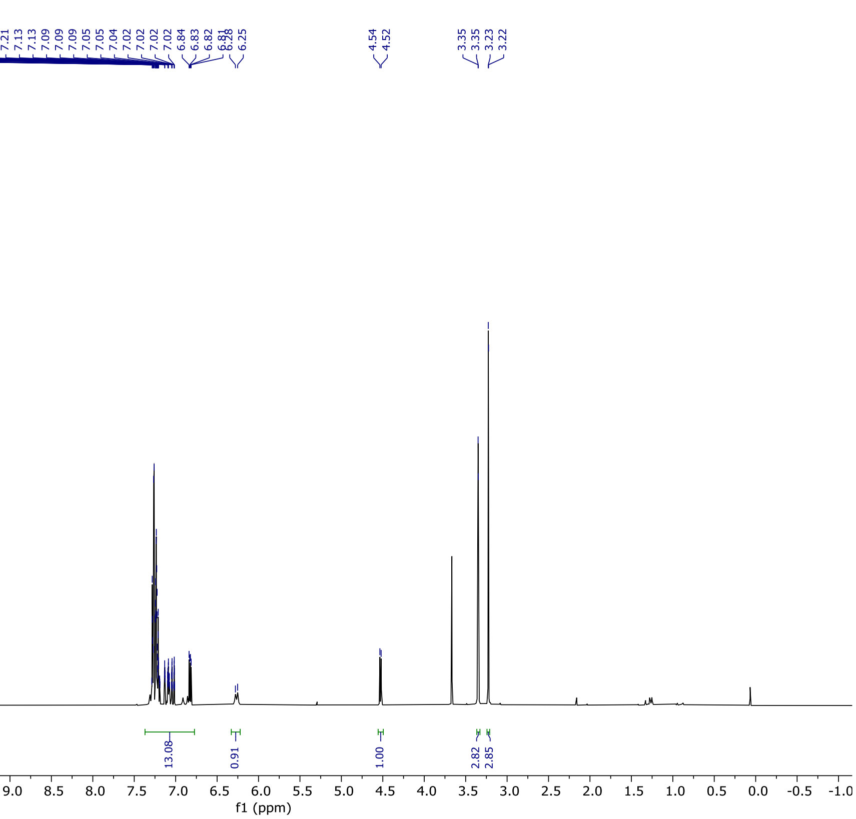

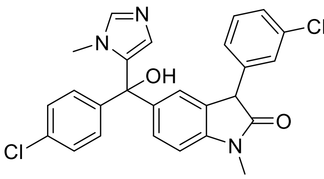

11

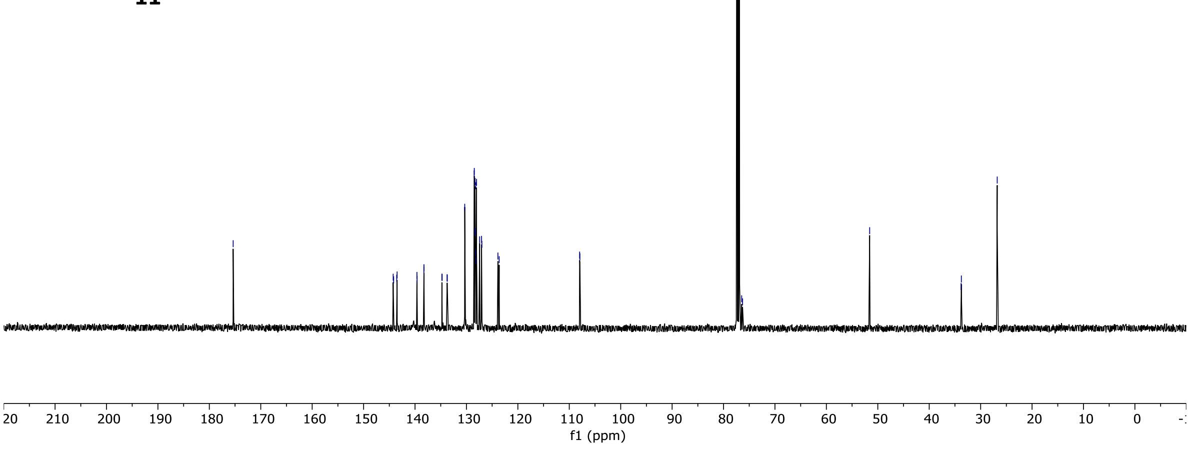

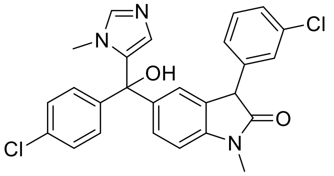

11

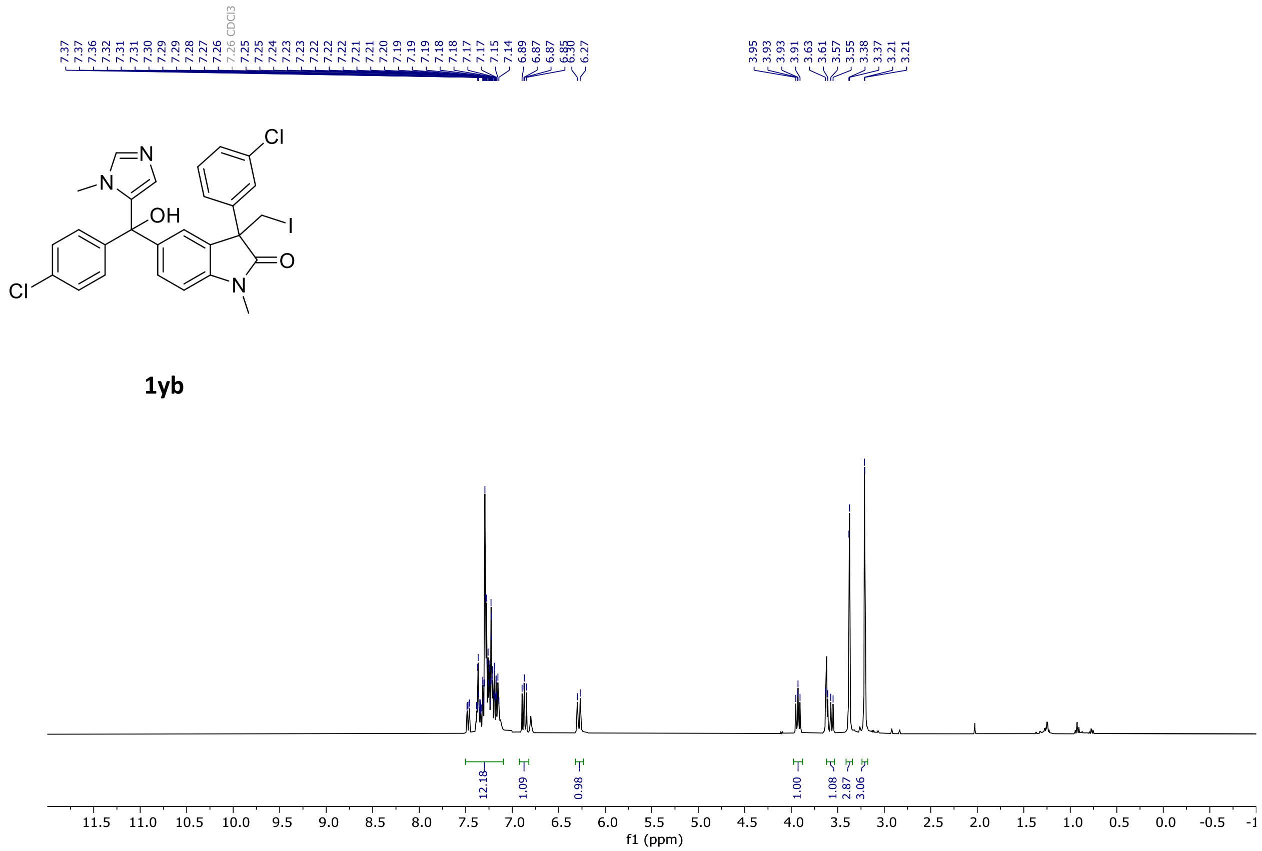

1yb

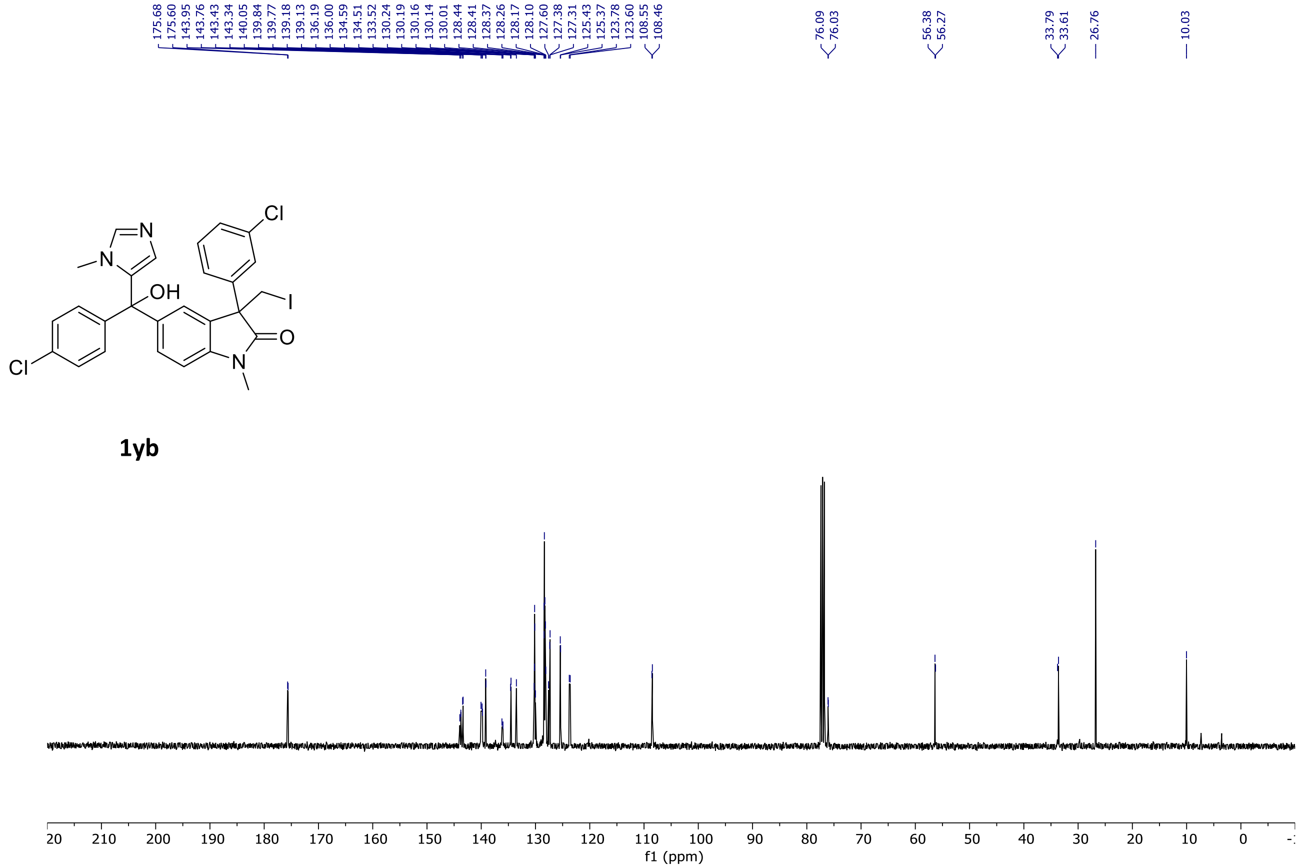

1yb

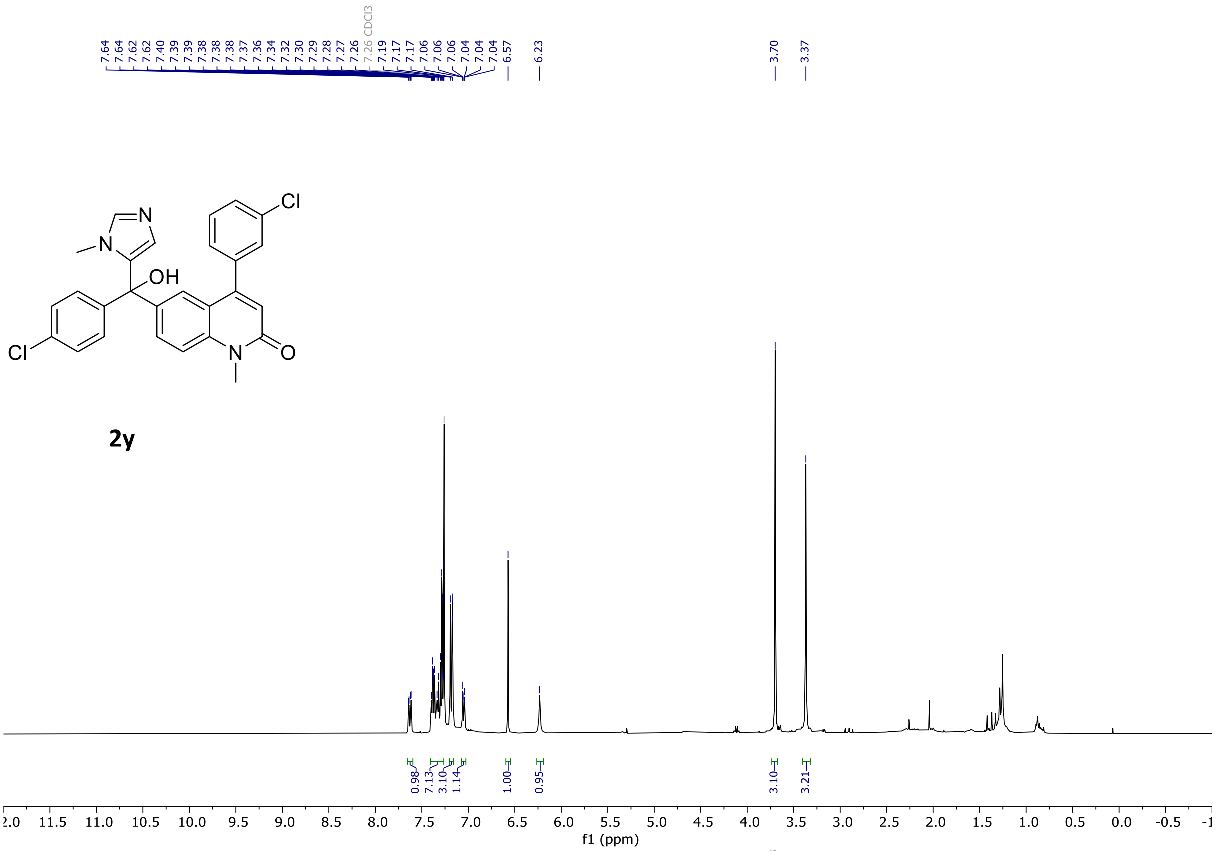
7.64
7.64
7.62
7.40
7.39
7.39
7.38
7.38
7.37
7.36
7.34
7.32
7.30
7.29
7.28
7.27
7.26 CDCl3
7.19
7.17
7.17
7.06
7.06
7.06
7.04
7.04
7.04
6.57
6.23
3.70
3.37
Cl
N
N
OH
Cl
N
O
2y
0.98
7.13
3.10
1.14
1.00
0.95
3.10
3.21
f1 (ppm)

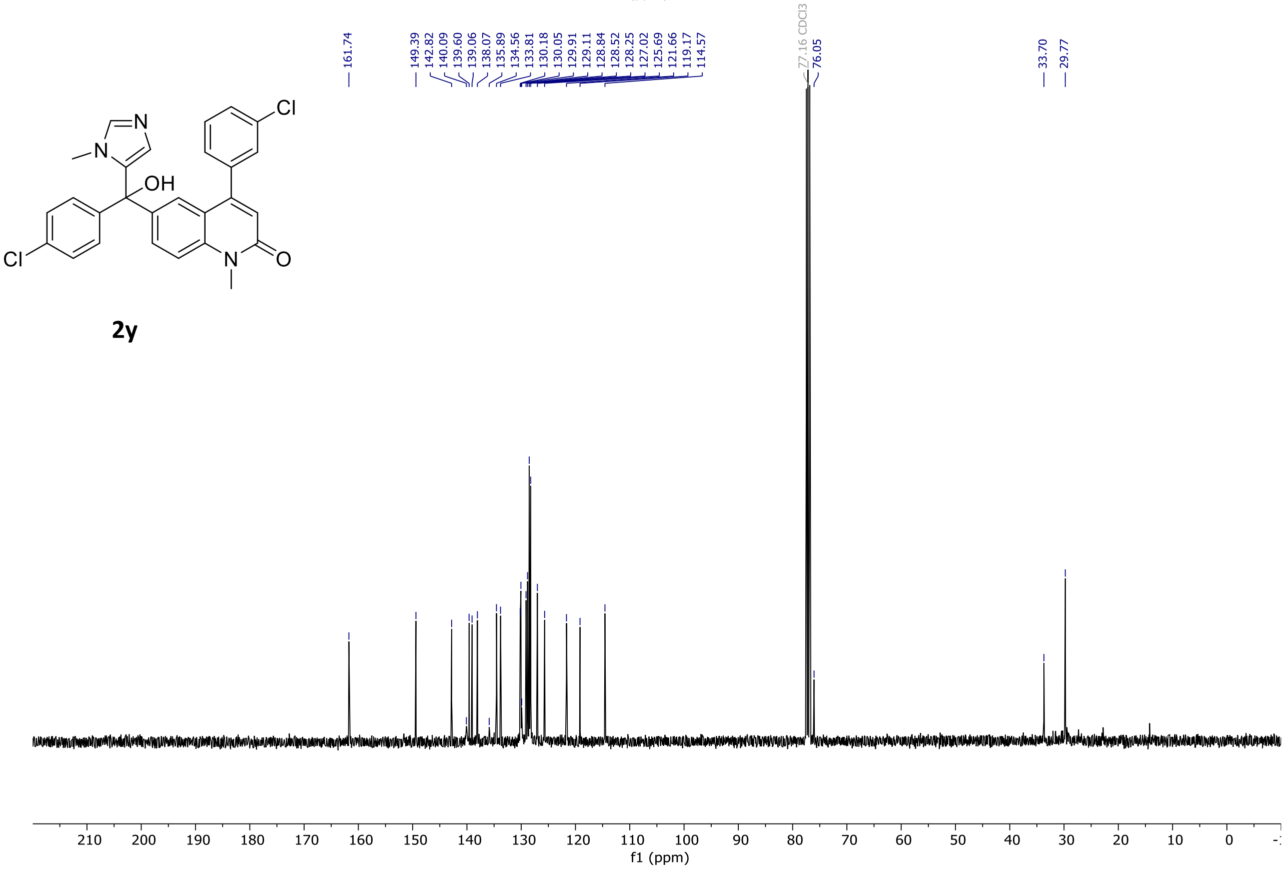
161.74
149.39
142.82
140.09
139.60
139.06
138.07
135.89
134.56
133.81
130.18
130.05
129.91
129.11
128.84
128.52
128.25
127.02
125.69
121.66
119.17
114.57
77.16 CDCl3
76.05
33.70
29.77
Cl
N
N
OH
Cl
N
O
2y
f1 (ppm)

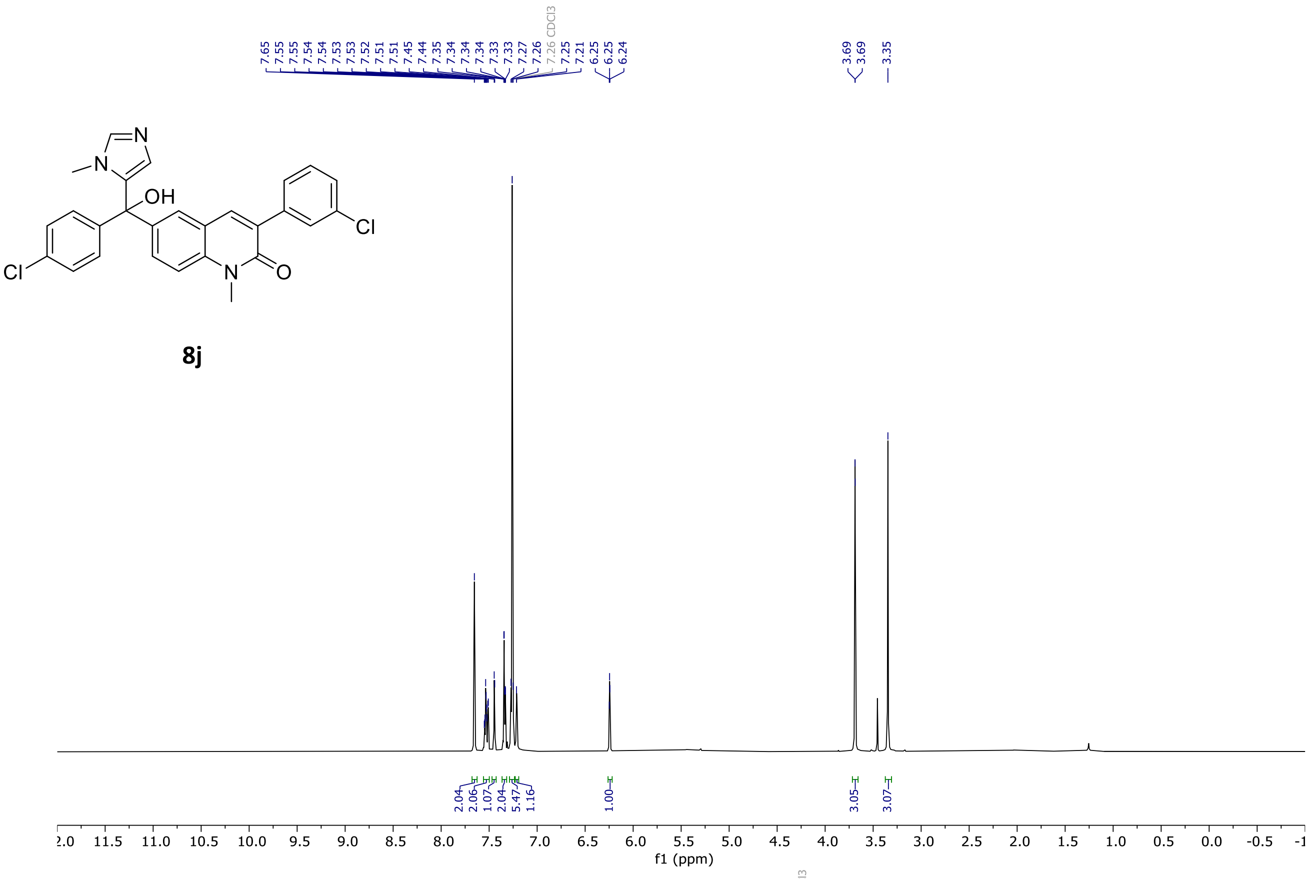
7.65
7.55
7.55
7.54
7.54
7.53
7.53
7.52
7.51
7.51
7.45
7.44
7.35
7.34
7.34
7.33
7.33
7.27
7.26
7.26 CDCl3
7.25
7.21
6.25
6.25
6.24
3.69
3.69
3.35
N
N
OH
Cl
Cl
N
O
8j
2.04
2.06
1.07
2.04
5.47
1.16
1.00
3.05
3.07
f1 (ppm)

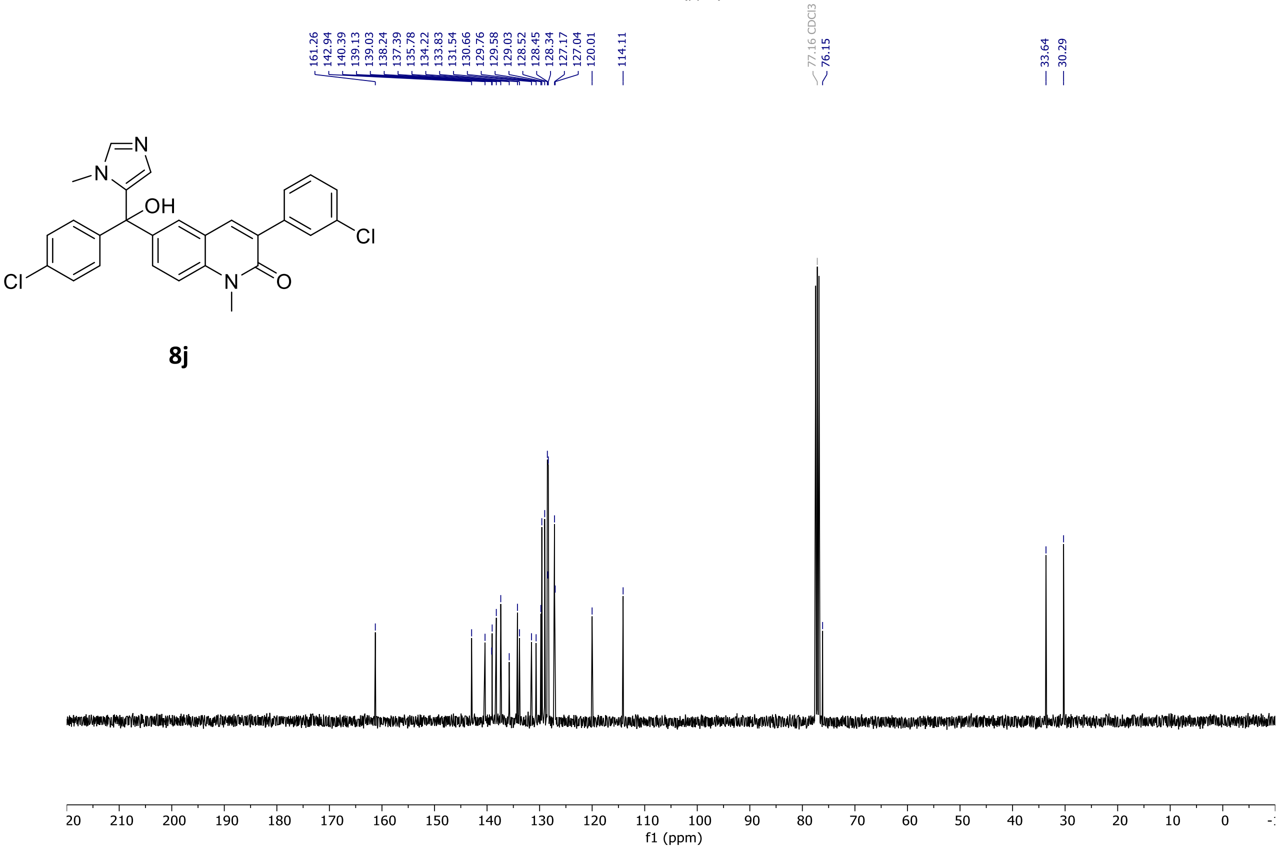
161.26
142.94
140.39
139.13
139.03
138.24
137.39
135.78
134.22
133.83
131.54
130.66
129.76
129.58
129.03
128.52
128.45
128.34
127.17
127.04
120.01
114.11
77.16 CDCl3
76.15
33.64
30.29
N
N
OH
Cl
Cl
N
O
8j
f1 (ppm)

Mechanistic experiments

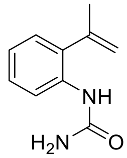

6

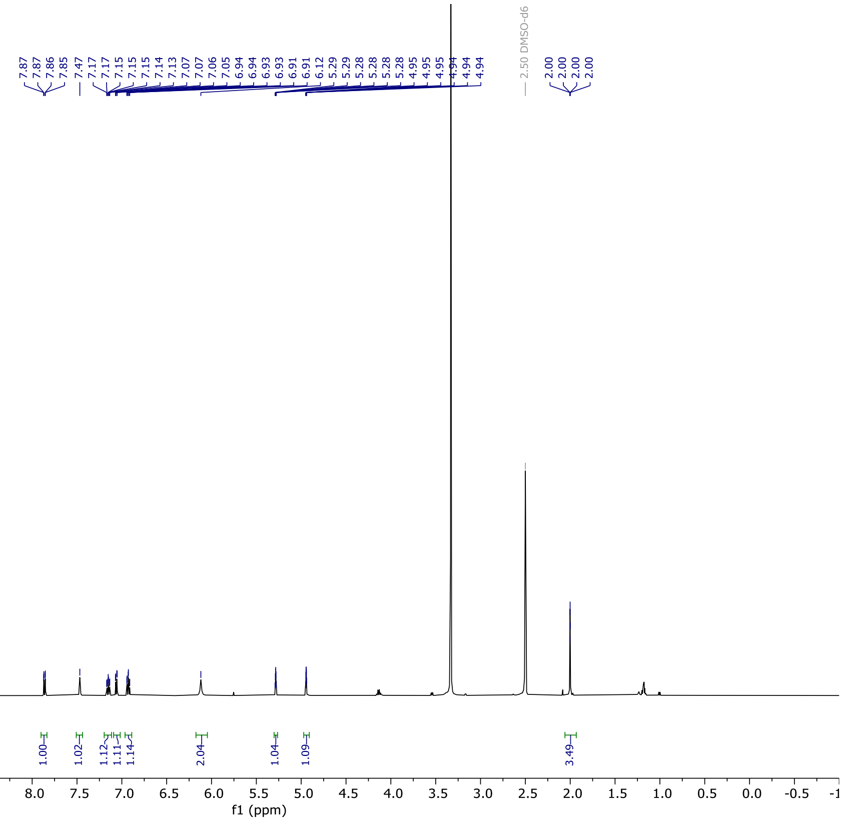

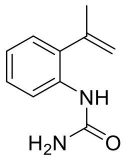

6

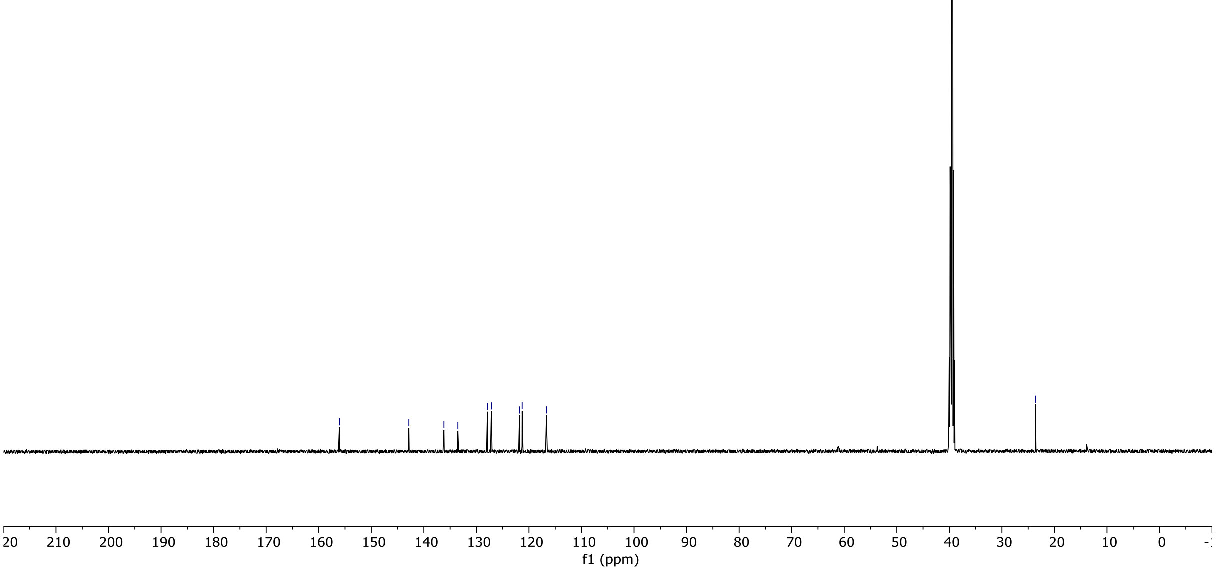

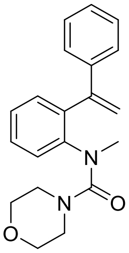

7

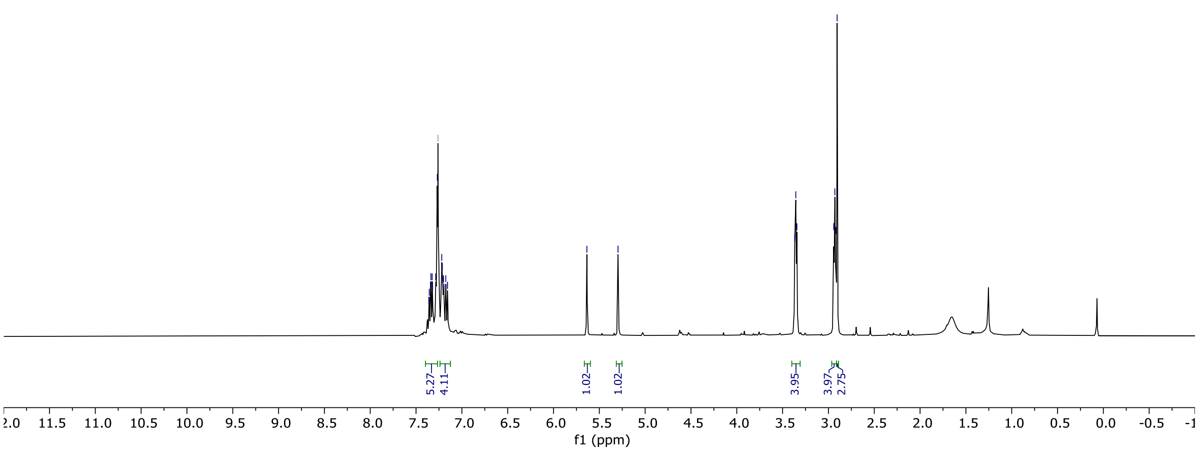

7.36
7.36
7.34
7.34
7.32
7.32
7.29
7.27
7.26 CDCl3
7.22
7.21
7.21
7.20
7.20
7.18
7.16
5.64
5.30
3.37
3.36
3.34
2.94
2.93
2.92
2.90
5.27
4.11
1.02
1.02
3.95
3.97
2.75
f1 (ppm)

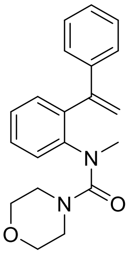

7

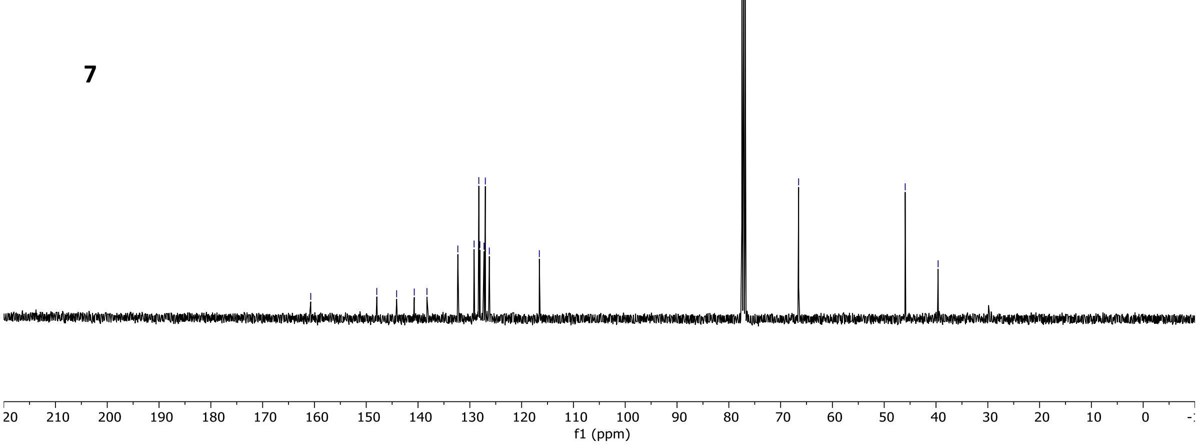

160.71
147.96
144.13
140.73
138.29
132.32
129.18
128.27
128.06
127.27
127.01
126.24
116.58
77.16 CDCl3
66.52
45.95
39.60
f1 (ppm)

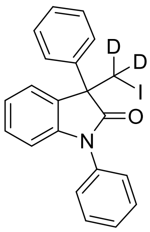
D
D
I
O
N

1mb-d2

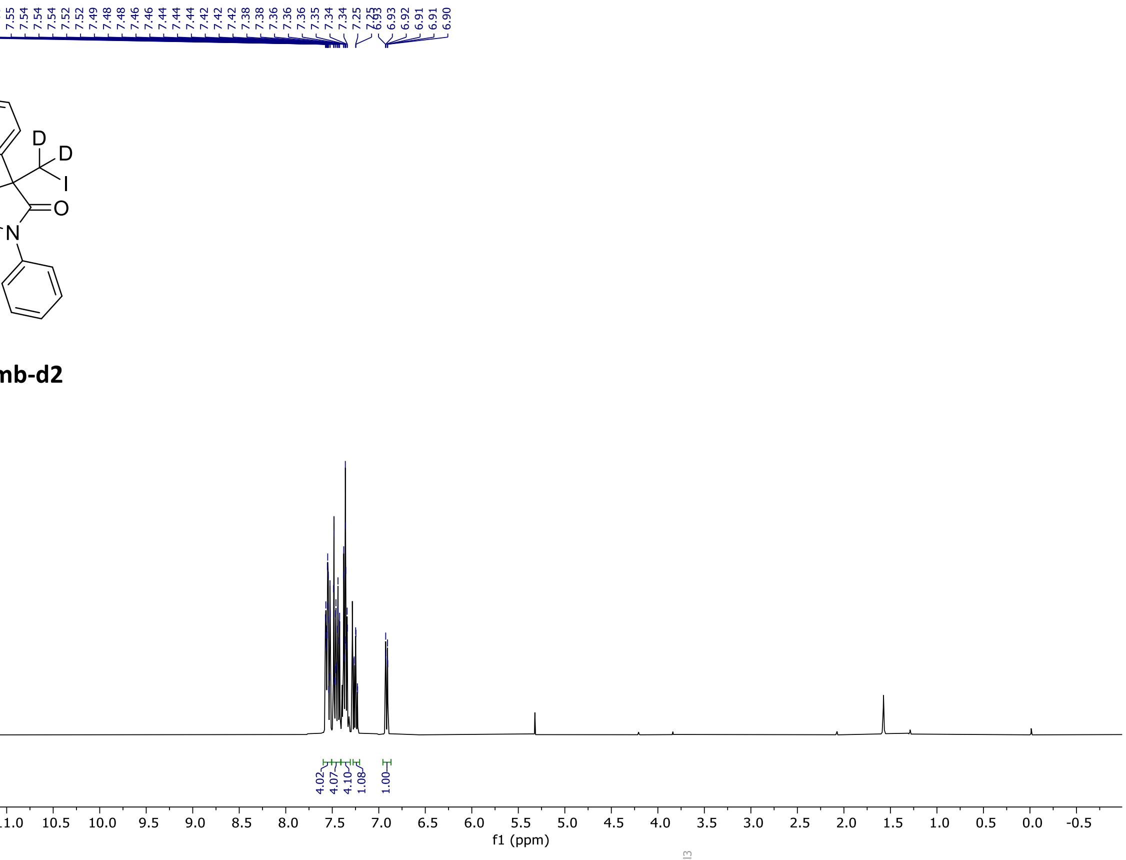
7.57 7.57 7.57 7.56 7.55 7.55 7.55 7.54 7.54 7.54 7.52 7.52 7.49 7.48 7.48 7.46 7.44 7.44 7.44 7.42 7.42 7.42 7.38 7.38 7.36 7.36 7.35 7.34 7.34 7.25 6.93 6.93 6.92 6.91 6.91 6.90
4.02 4.07 4.10 1.08 1.00
f1 (ppm)

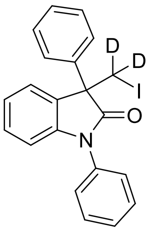
D
D
I
O
N

1mb-d2

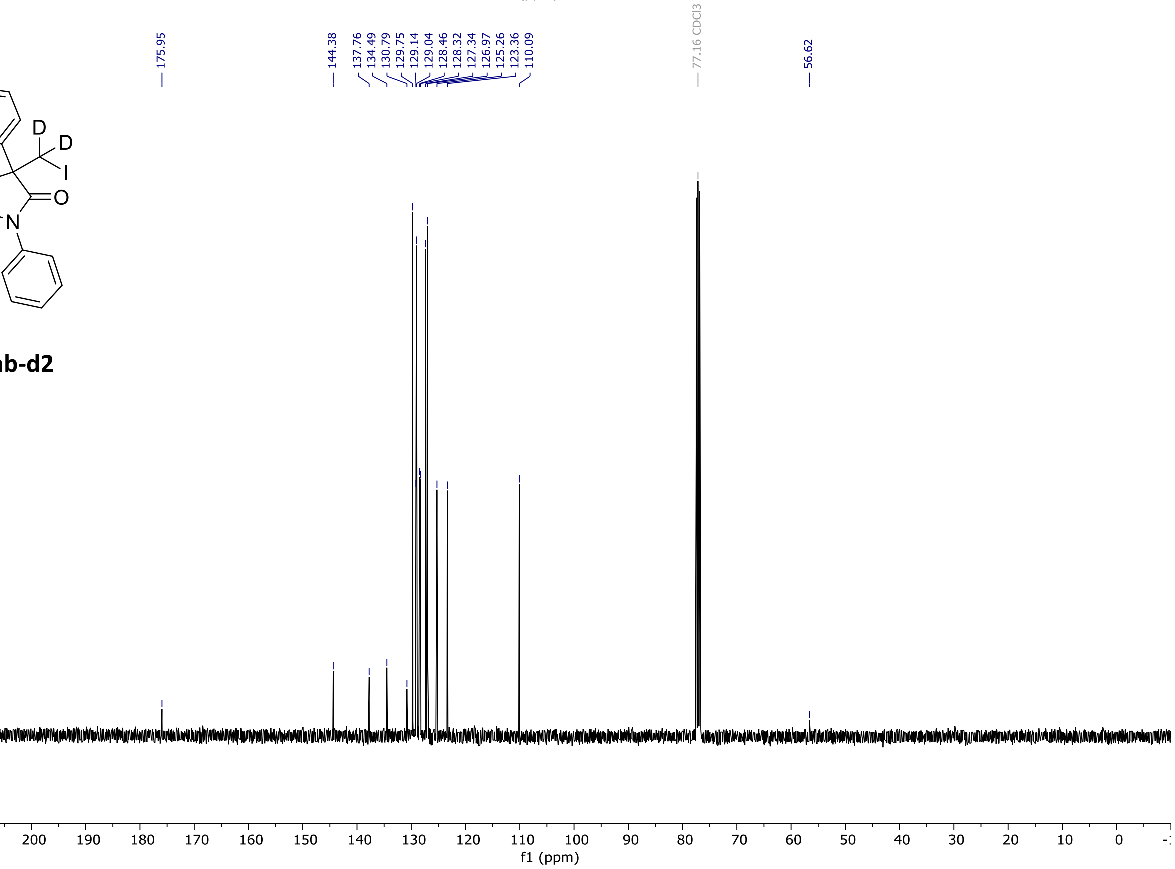
175.95
144.38 137.76 134.49 130.79 129.75 129.14 129.04 128.46 128.32 127.34 126.97 125.26 123.36 110.09
77.16 CDCl3
56.62
f1 (ppm)
